# Supplementary material for: Global Burden of Vitamin A Deficiency in 204 Countries and Territories from 1990–2019
Source: Nutrients. 2022 Feb 23;14(5):950. doi: 10.3390/nu14050950 (PMC8912822; doi:10.3390/nu14050950)

nutrients

Global burden of vitamin A deficiency in 204 countries and territories from 1990-2019

Supplemental figure

Supplemental Figure S1: The flowchart for VAD analysis in GBD. DALYs, disability adjusted life year. DHS, Demographic and Health Surveys. GBD, Global Burden of Disease. MICS, Multiple Indicator Cluster Survey. VAD, vitamin A deficiency. YLDs, years lived with disability.

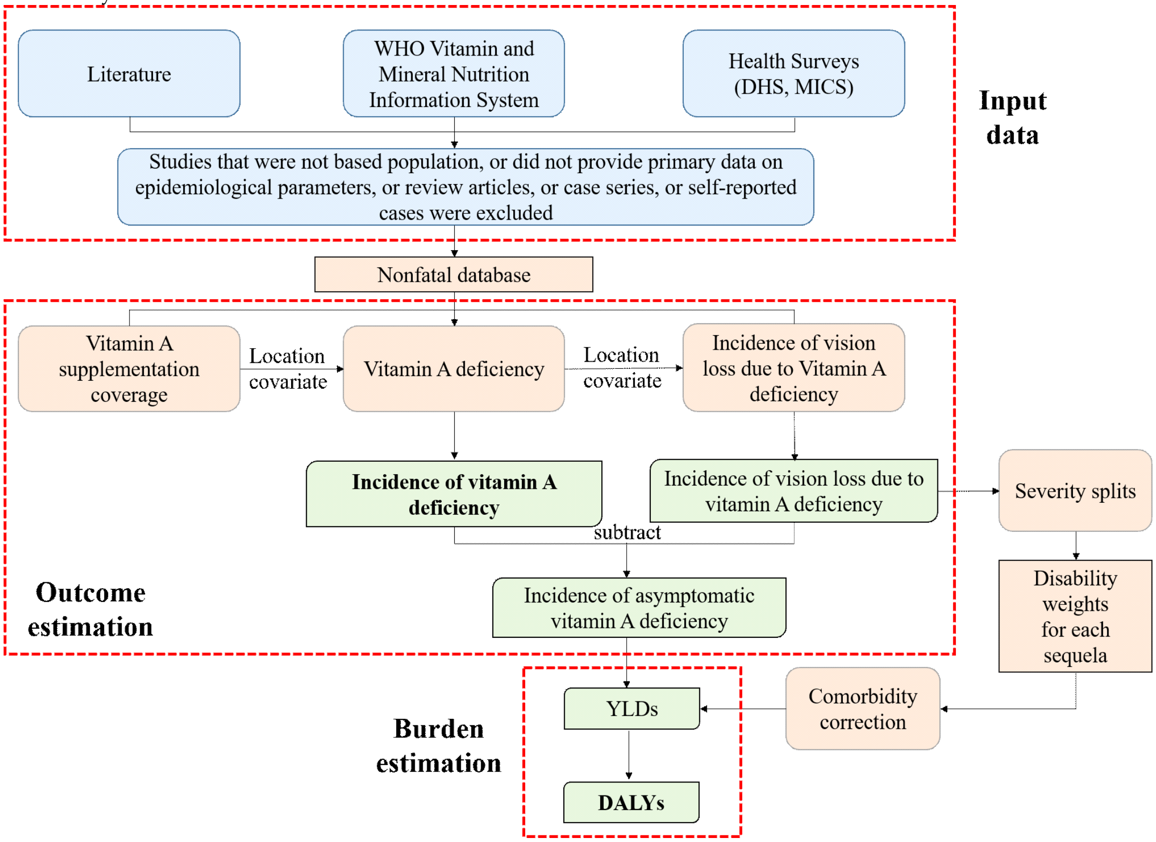

Supplemental Figure S2: The incidence number and DALY number of VAD between 1990 and 2019 among sexes. (A) Incidence number. (B) DALY number. DALY, disability adjusted life year. VAD, vitamin A deficiency.

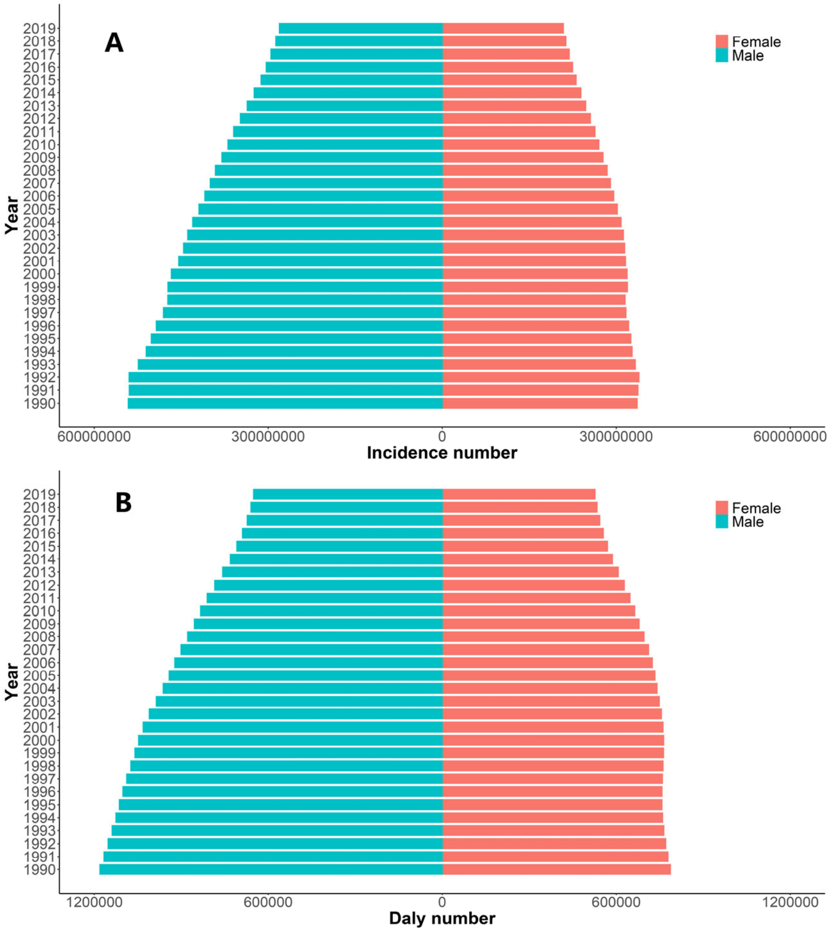

Supplemental Figure S3: The change trends of age-standardized rates of VAD among different SDI quintiles and sexes from 1990 to 2019. Age-standardized incidence rate of (A) both sexes; (B) female; (C) male in different SDI quintiles. Age-standardized DALY rate of (D) both sexes; (E) female; (F) male in different SDI quintiles. DALY, disability adjusted life year. SDI, socio-demographic index. VAD, vitamin A deficiency.

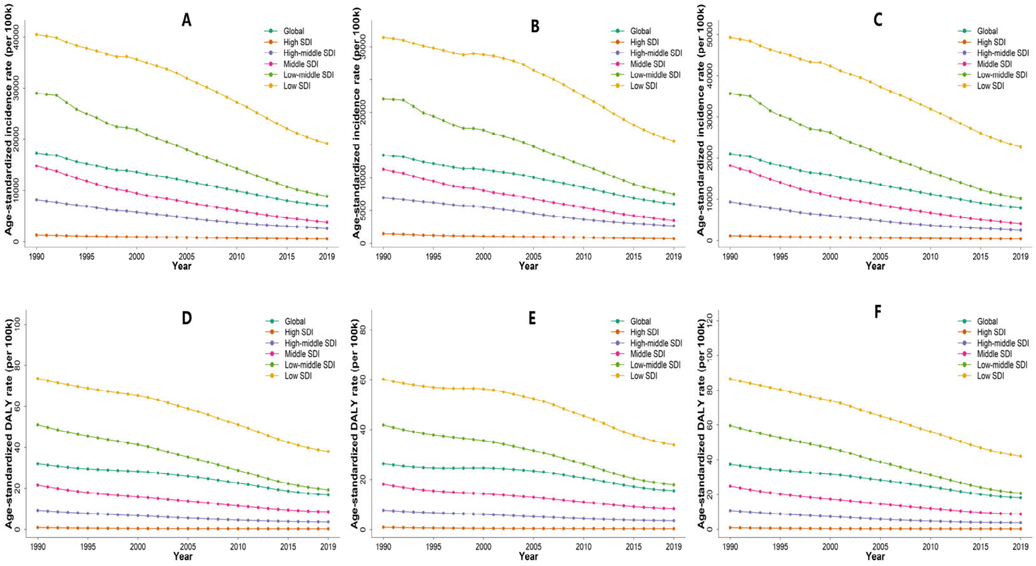

Supplemental Figure S4: The ratio of male to female VAD incidence among different age groups in 2019. (A) Global. (B) High SDI. (C) High-middle SDI. (D) Middle SDI. (E) Low-middle SDI. (F) Low SDI. SDI, socio-demographic index. VAD, vitamin A deficiency.

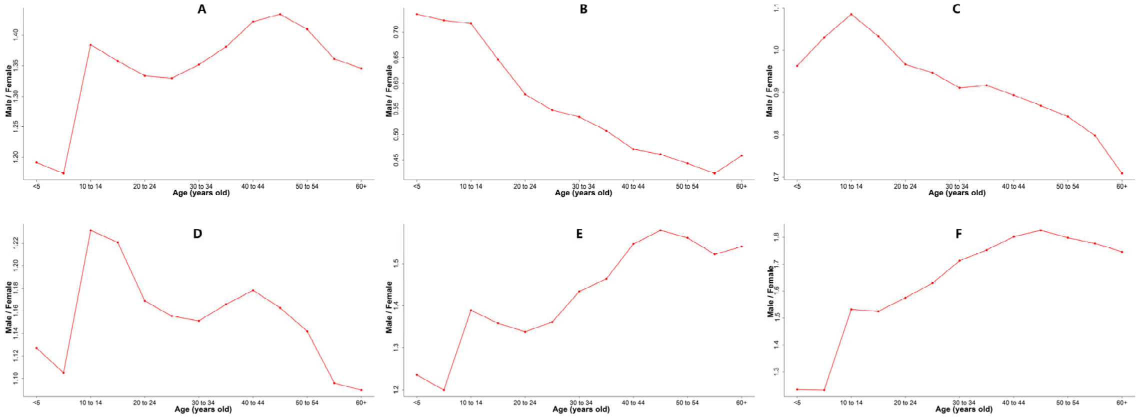

Supplemental Figure S5: Distribution of different ages in VAD incidence. (A) Global. (B) High SDI. (C) High-middle SDI. (D) Middle SDI. (E) Low-middle SDI. (F) Low SDI. SDI, socio-demographic index. VAD, vitamin A deficiency.

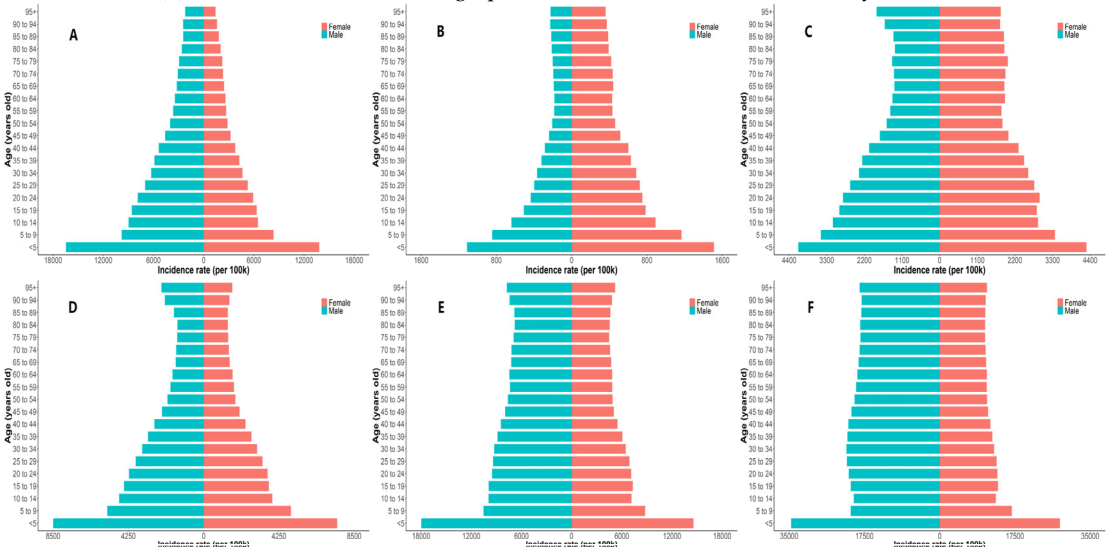

Supplemental Figure S6: The correlation between EAPC and age-standardized rates of VAD and SDI in 2019. (A) EAPC and age-standardized incidence rate. (B) EAPC and SDI in incidence. (C) EAPC and age-standardized DALY rate. (D) EAPC and SDI in DALYs. The circles represent countries that were available on SDI data. The size of circle is increased with the cases of VAD. The ϱ indices Pearson's correlation coefficient and *P* values were derived from Pearson's correlation analysis. DALY, disability adjusted life year. EAPC, estimated annual percentage change. SDI, socio-demographic index. VAD, vitamin A deficiency.

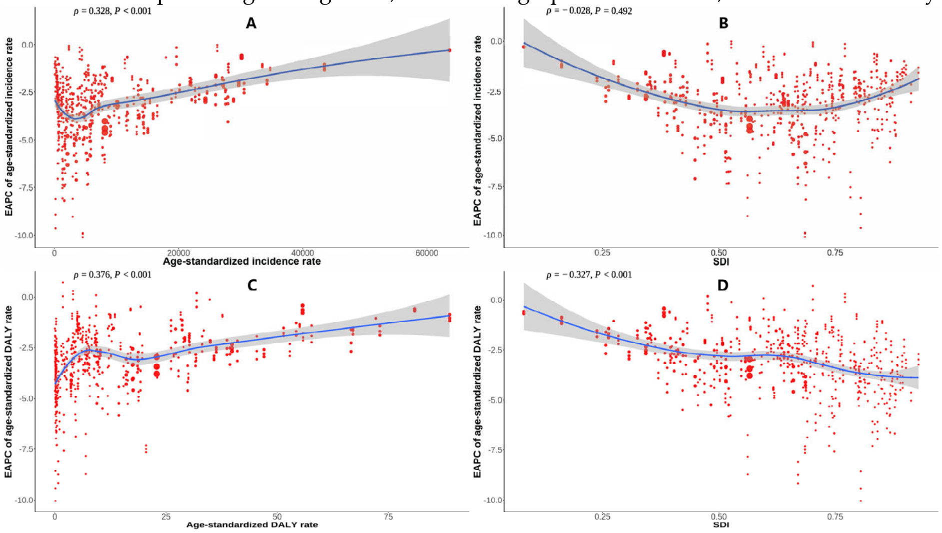

Supplemental Figure S7: The age-standardized rates of VAD per 100,000 population among regions based on SDI in 2019. (A) age-standardized incidence rate. (B) age-standardized DALY rate. DALY, disability adjusted life year. SDI, social development index. VAD, vitamin A deficiency.

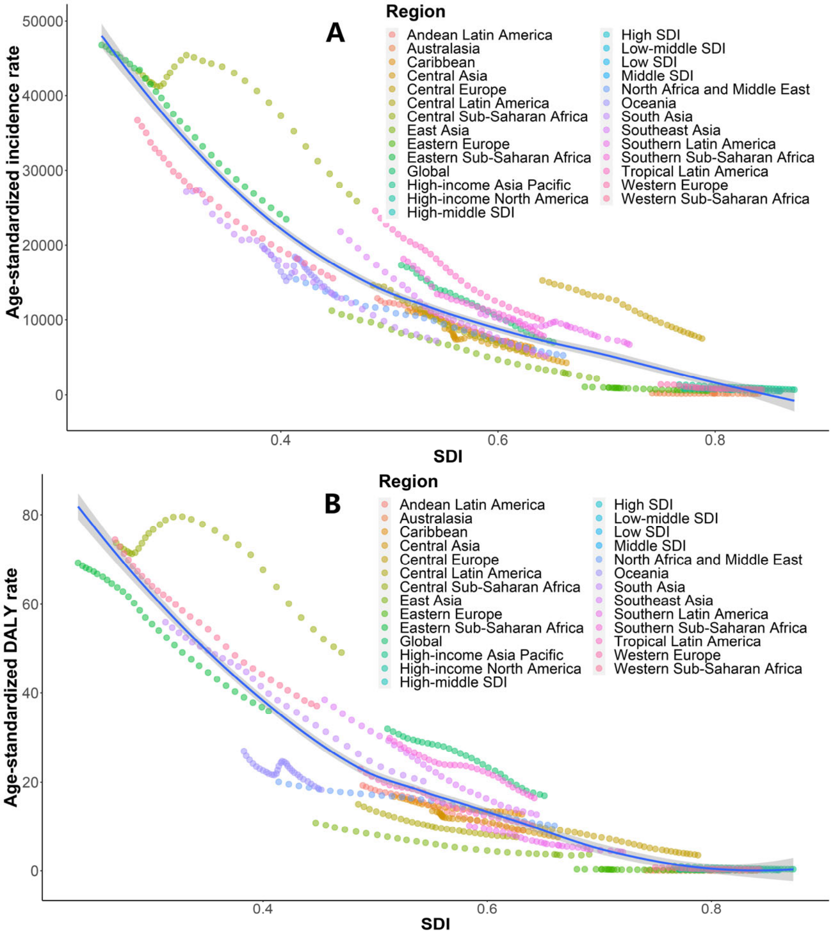

Supplemental Figure S8: The incidence and DALY rates of VAD in different age groups. (A) Incidence in 1990. (B) Incidence in 2019. (C) DALY rate in 1990. (D) DALY rate in 2019. DALY, disability adjusted life year. VAD, vitamin A deficiency.

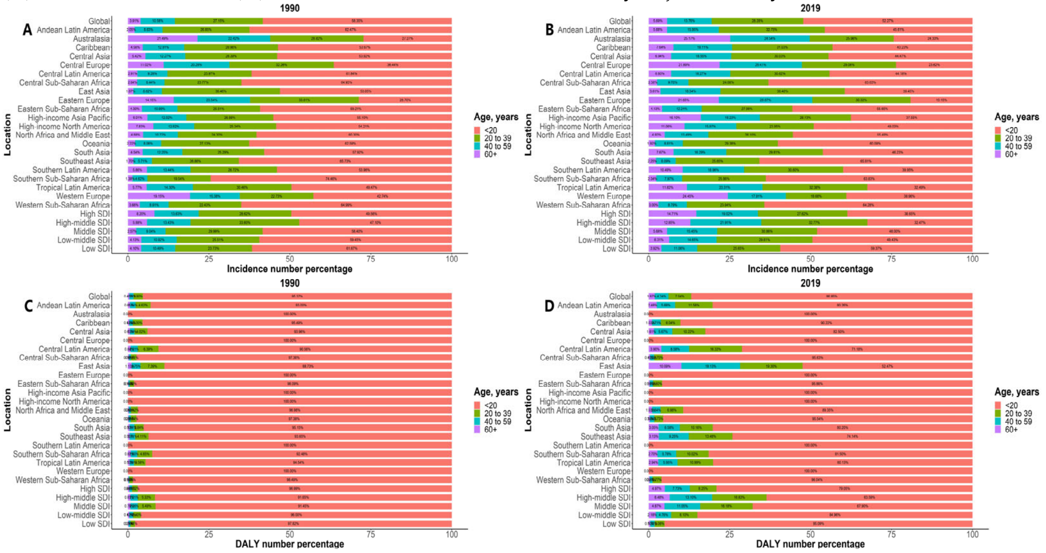

Supplemental Figure S9: The proportion of different ages in VAD incidence number for both sexes by years. (A) Global. (B) High SDI. (C) High-middle SDI. (D) Middle SDI. (E) Low-middle SDI. (F) Low SDI. SDI, socio-demographic index. VAD, vitamin A deficiency.

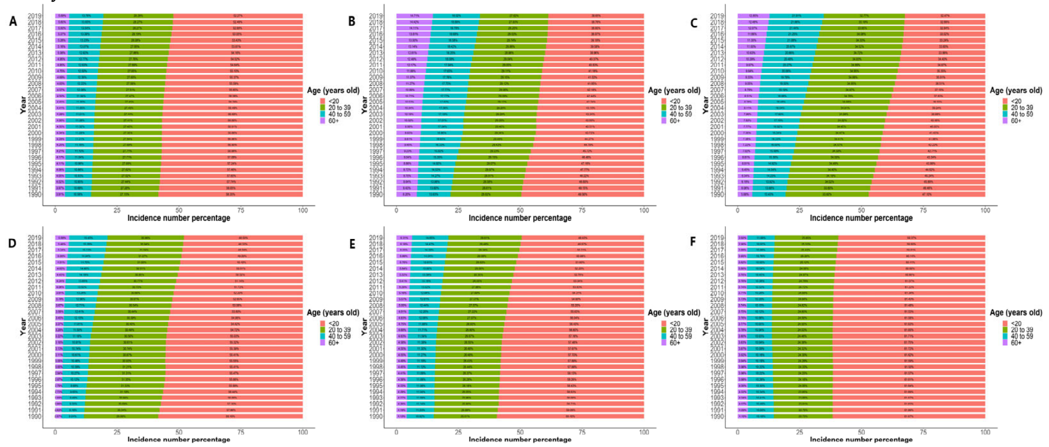

Supplemental Figure S10: The global EAPC of VAD for both sexes in 204 countries. (A) The EAPC of age-standardized incidence rate. (B) The EAPC of age-standardized DALY rate. DALY, disability adjusted life year. EAPC, estimated annual percentage change. VAD, vitamin A deficiency.

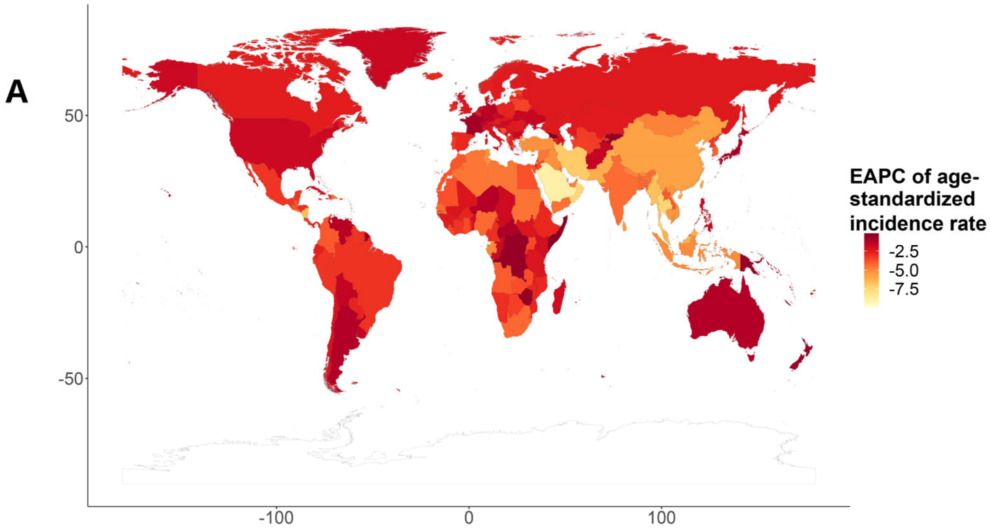

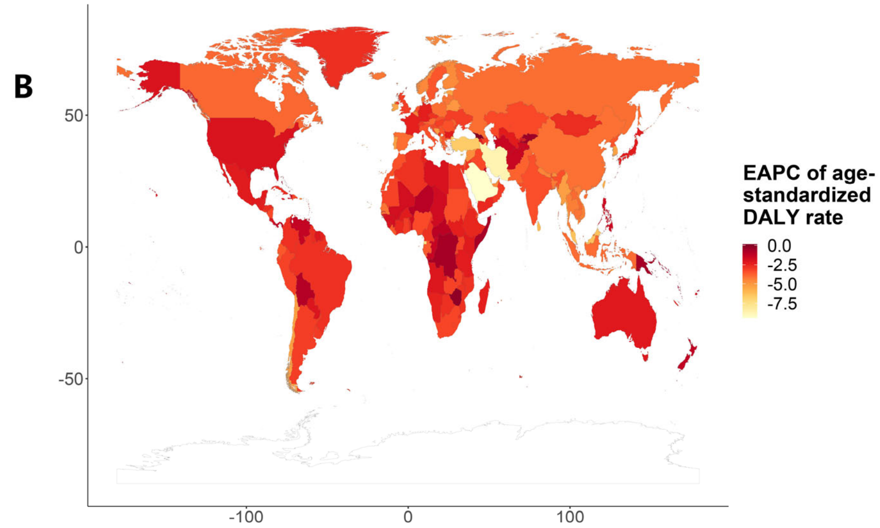

Supplemental Figure S11: The global EAPC of VAD for female in 204 countries. (A) The EAPC of age-standardized incidence rate. (B) The EAPC of age-standardized DALY rate. DALY, disability adjusted life year. EAPC, estimated annual percentage change. VAD, vitamin A deficiency.

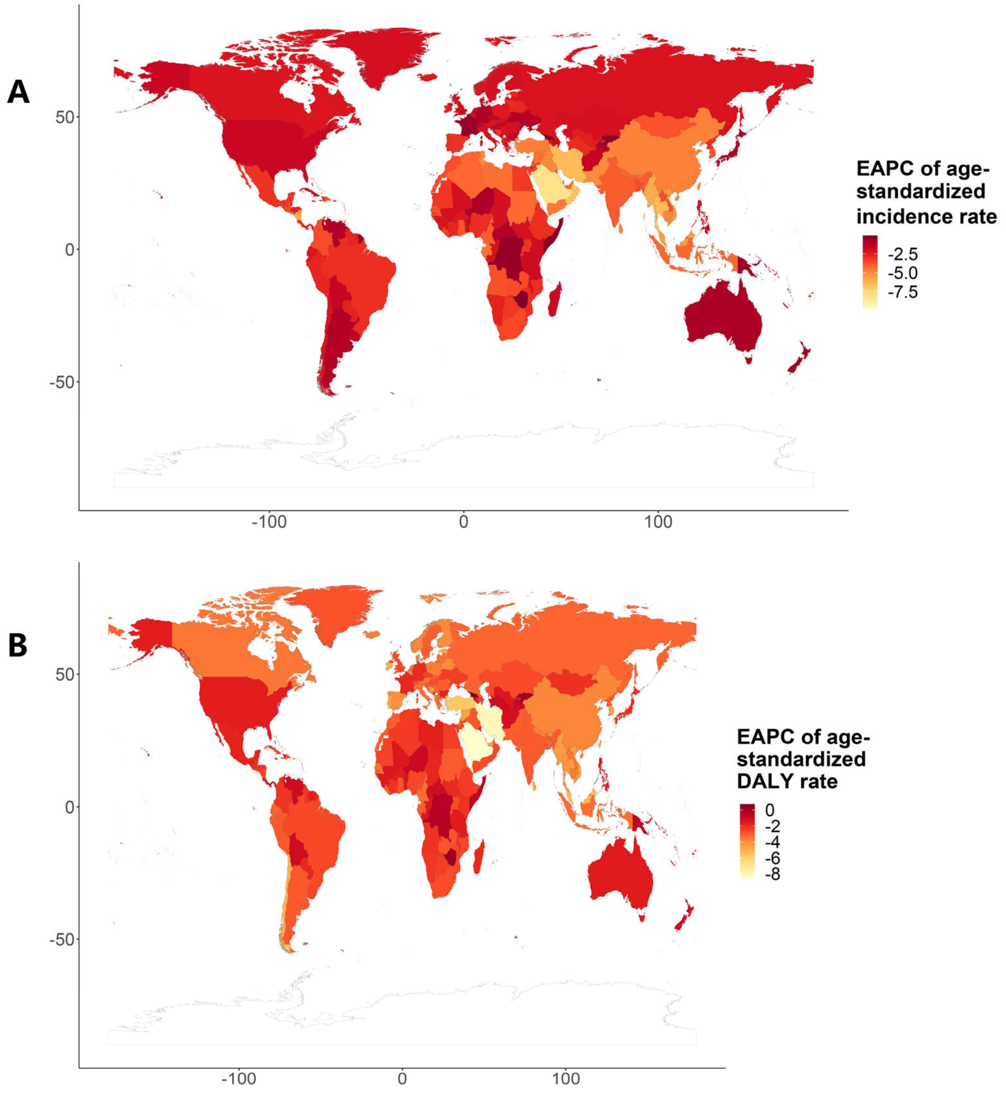

Supplemental Figure S12: The global EAPC of VAD for male in 204 countries. (A) The EAPC of age-standardized incidence rate. (B) The EAPC of age-standardized DALY rate. DALY, disability adjusted life year. EAPC, estimated annual percentage change. VAD, vitamin A deficiency.

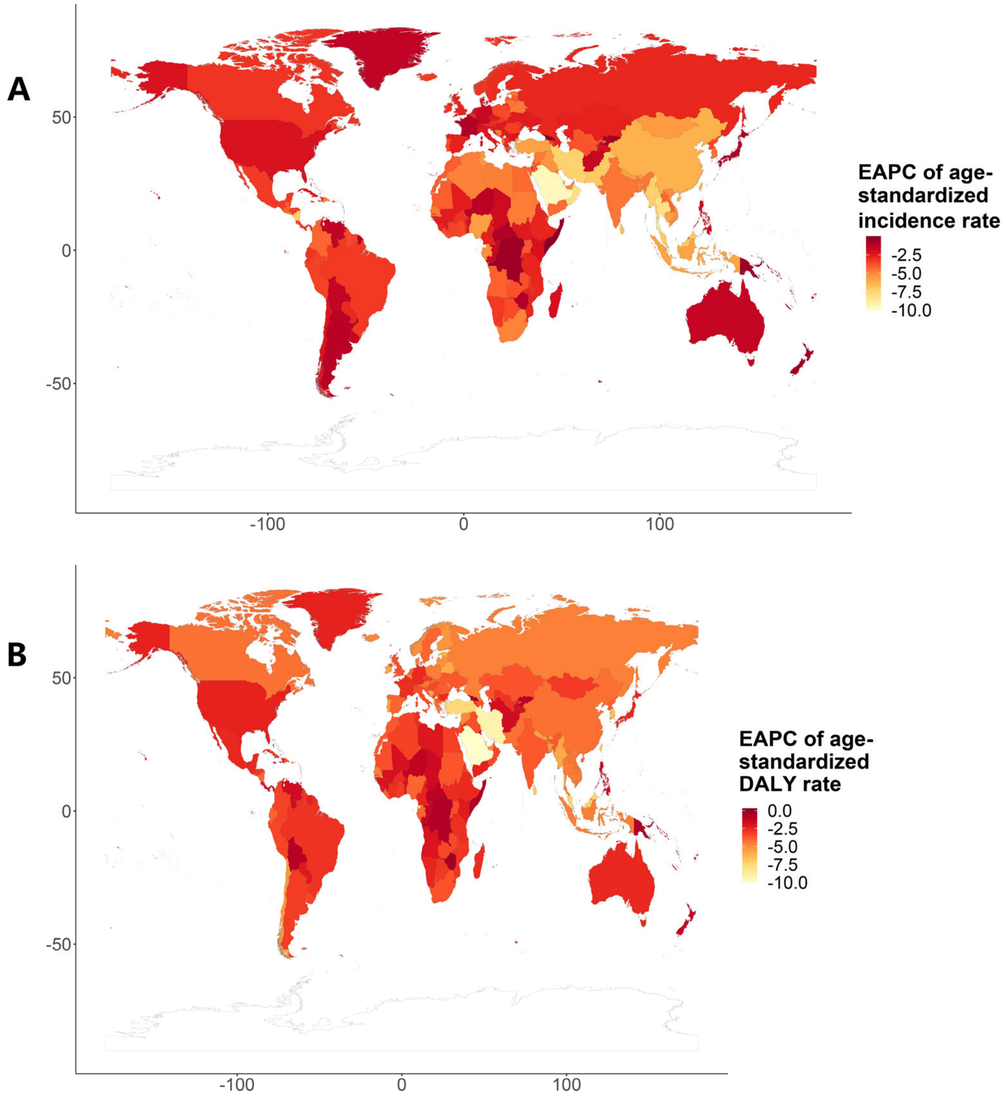

Supplemental Figure S13: The global burden of VAD for both sexes in 204 countries. (A) The age standardized incidence rate of VAD in 2019. (B) The age-standardized DALY rate of VAD in 2019. DALY, disability adjusted life year. VAD, vitamin A deficiency.

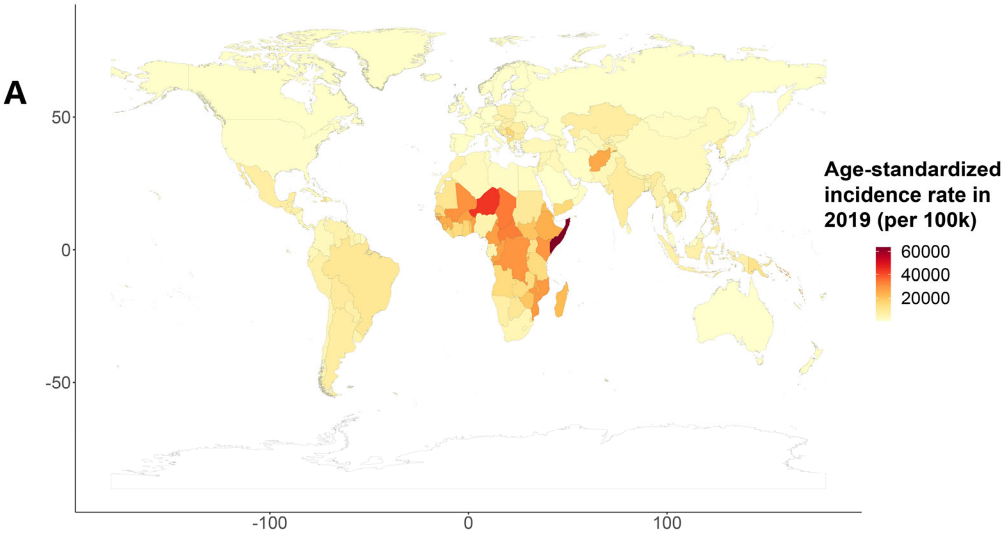

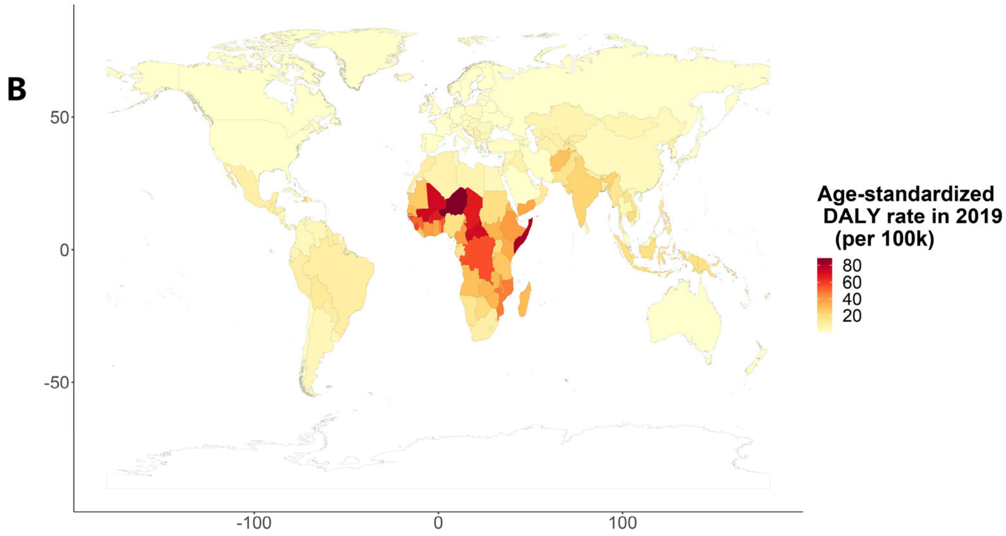

Supplemental Figure S14: The global burden of VAD for female in 204 countries. (A) The age standardized incidence rate of VAD in 2019. (B) The age-standardized DALY rate of VAD in 2019. DALY, disability adjusted life year. VAD, vitamin A deficiency.

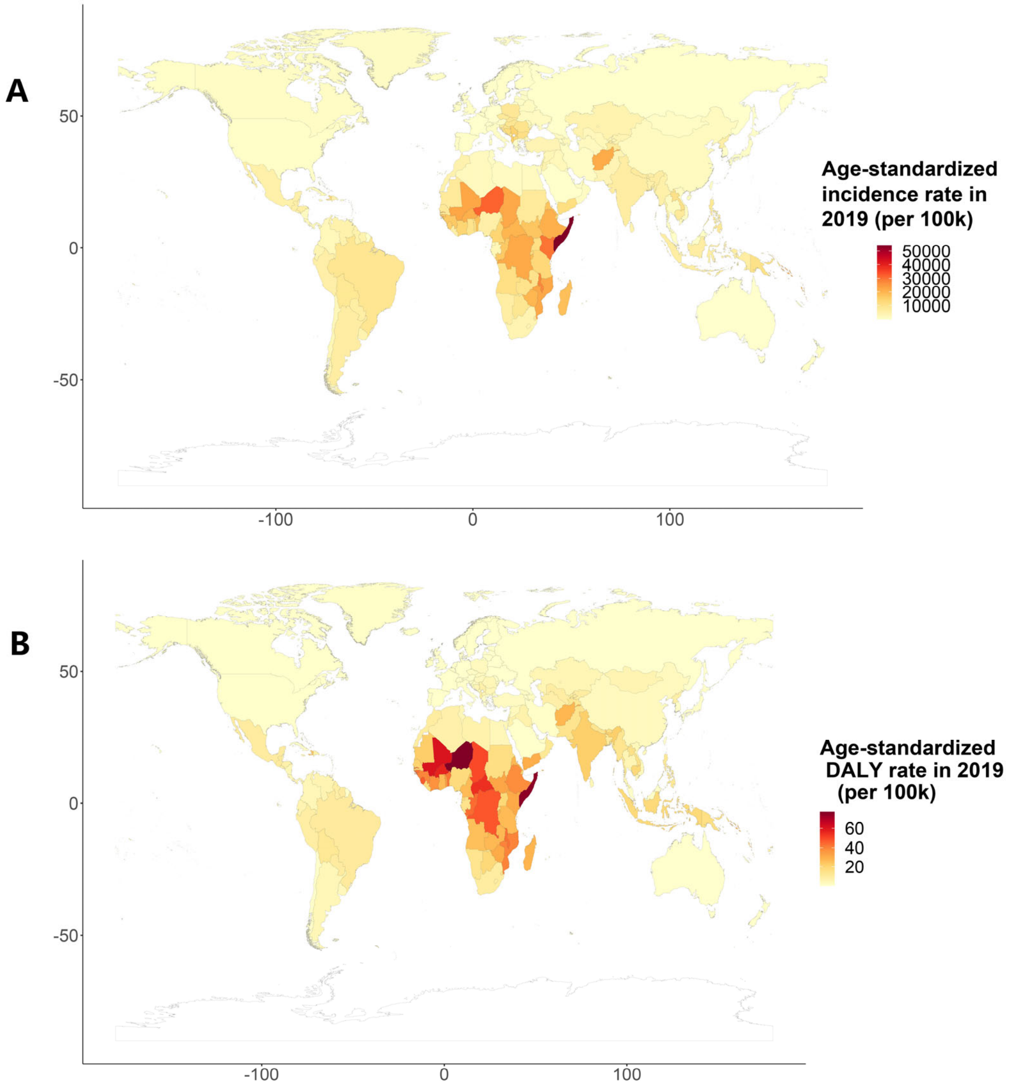

Supplemental Figure S15: The global burden of VAD for male in 204 countries. (A) The age standardized incidence rate of VAD in 2019. (B) The age-standardized DALY rate of VAD in 2019. DALY, disability adjusted life year. VAD, vitamin A deficiency.

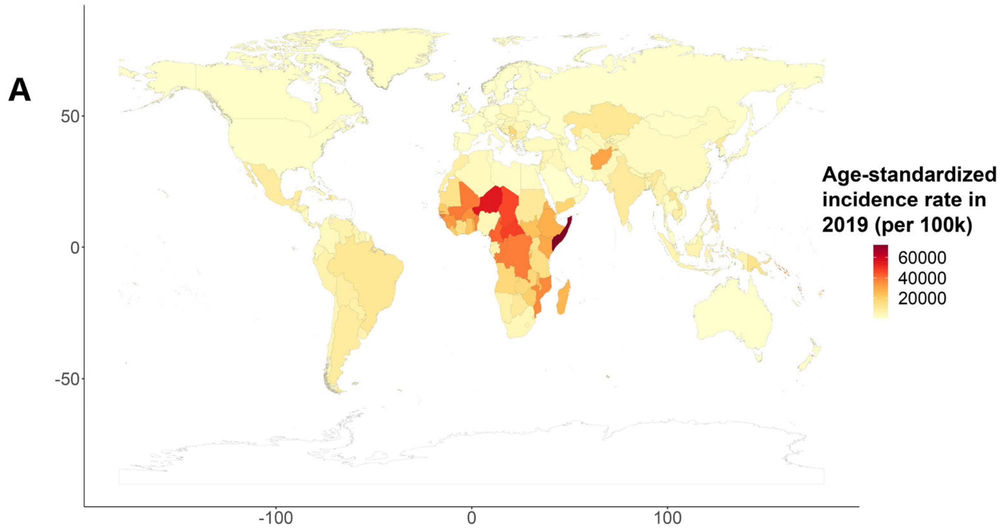

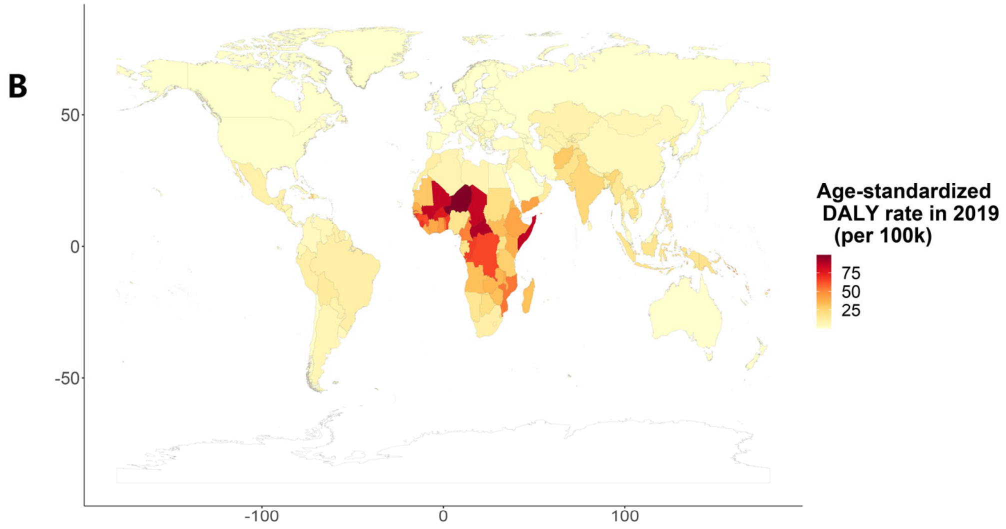

Supplemental Figure S16: The ratio of male to female DALY rate among different age groups in 2019. (A) Global. (B) High SDI. (C) High-middle SDI. (D) Middle SDI. (E) Low-middle SDI. (F) Low SDI. DALY, disability adjusted life year. SDI, socio-demographic index.

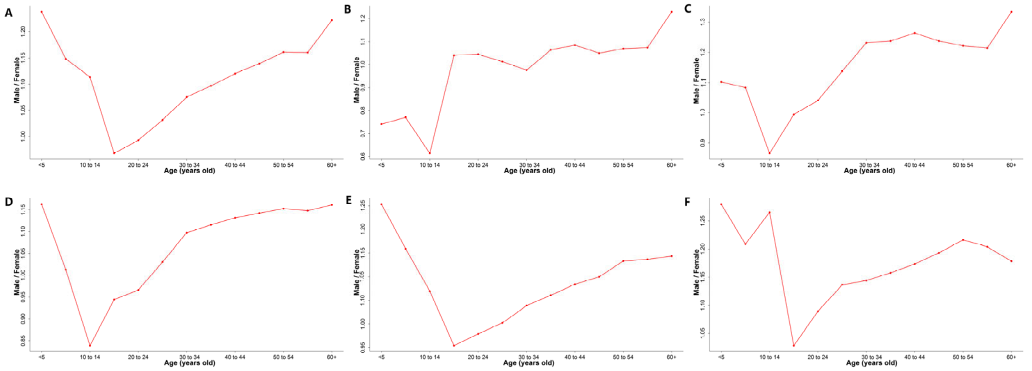

Supplemental Figure S17: Distribution of different ages in VAD DALY rate. (A) Global. (B) High SDI. (C) High-middle SDI. (D) Middle SDI. (E) Low-middle SDI. (F) Low SDI. DALY, disability adjusted life year. SDI, socio-demographic index. VAD, vitamin A deficiency.

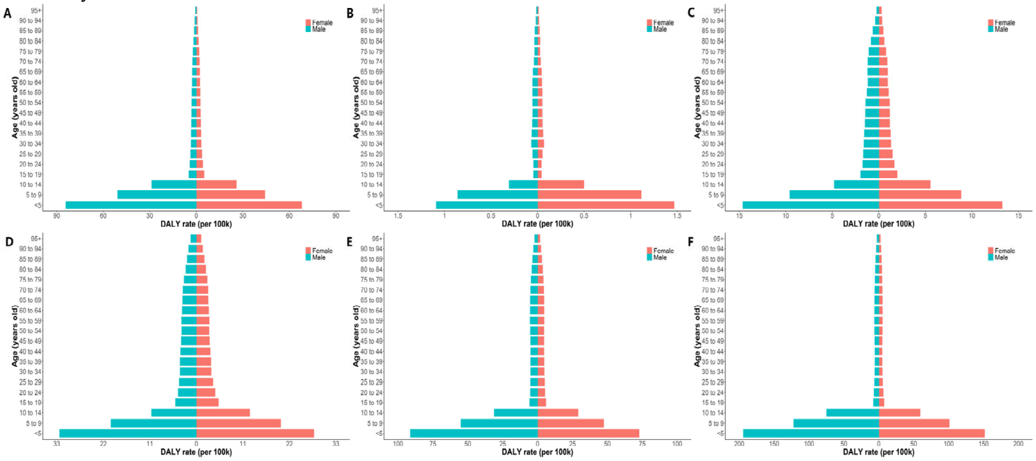

Supplemental Figure S18: The proportion of different ages in VAD DALY number for both sexes by years. (A) Global. (B) High SDI. (C) High-middle SDI. (D) Middle SDI. (E) Low-middle SDI. (F) Low SDI. DALY, disability adjusted life year. SDI, socio-demographic index. VAD, vitamin A deficiency.

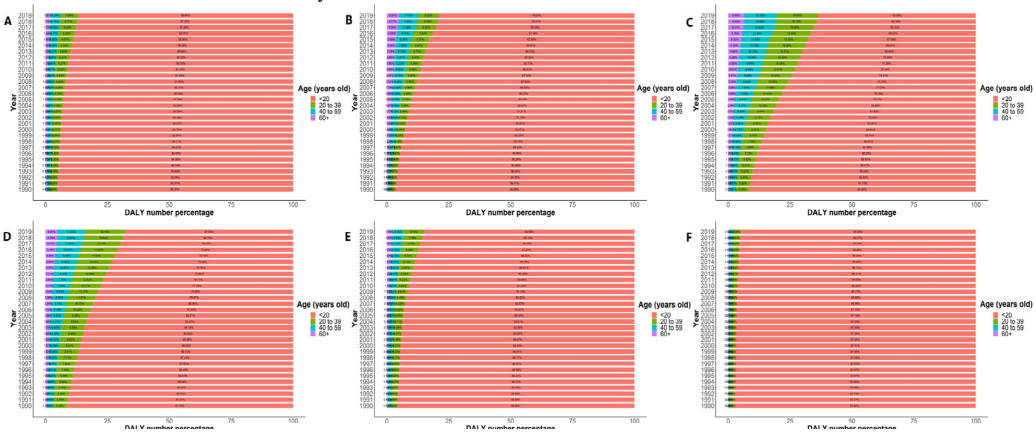

Supplement: Supplementary file 1 [file nutrients-14-00950-s001.zip › Supplemental figure.pdf]
