# Supplementary material for: Global Burden of Vitamin A Deficiency in 204 Countries and Territories from 1990–2019
Source: Nutrients. 2022 Feb 23;14(5):950. doi: 10.3390/nu14050950 (PMC8912822; doi:10.3390/nu14050950)
Supplement: Supplementary file 1 [file nutrients-14-00950-s001.zip › Supplemental table.pdf]

nutrients

MDPI

Global burden of vitamin A deficiency in 204 countries and territories from 1990-2019

Supplemental Tables

Supplemental Table S1: The top three and the bottom three regions of VAD incidence or DALY.

sex	Top three regions			Bottom three regions		
			**2019 Age-standardized rates (per 100,000 people)**			
			**Age-standardized incidence rate**			
**both**	Central Sub-Saharan Africa (25905.22)	Eastern Sub-Saharan Africa (23500.02)	Western Sub-Saharan Africa (15570.91)	Australasia (148.85)	High-income North America (485.64)	Eastern Europe (530.88)
**female**	Eastern Sub-Saharan Africa (20731.43)	Central Sub-Saharan Africa (18608.15)	Western Sub-Saharan Africa (11458.81)	Australasia (187.25)	Western Europe (657.56)	Eastern Europe (677.48)
**male**	Central Sub-Saharan Africa (33242.35)	Eastern Sub-Saharan Africa (26294.39)	Western Sub-Saharan Africa (19857.65)	Australasia (110.28)	High-income North America (255.41)	Eastern Europe (376.55)
			**Age-standardized DALY rate**			
**both**	Central Sub-Saharan Africa (49.08)	Western Sub-Saharan Africa (37.04)	Eastern Sub-Saharan Africa (35.90)	Australasia (0.05)	Eastern Europe (0.11)	High-income North America (0.15)

**female**	Central Sub-Saharan Africa (41.43)	Eastern Sub-Saharan Africa (33.71)	Western Sub-Saharan Africa (31.46)	Australasia (0.05)	Eastern Europe (0.11)	High-income North America (0.19)
**male**	Central Sub-Saharan Africa (56.68)	Western Sub-Saharan Africa (42.57)	Eastern Sub-Saharan Africa (38.04)	Australasia (0.06)	Eastern Europe (0.10)	High-income North America (0.11)
			**1990-2019 increase times**			
			**Incidence (cases)**			
**both**	Central Sub-Saharan Africa (0.42)	Oceania (0.31)	Eastern Sub-Saharan Africa (0.09)	East Asia (−0.79)	Southeast Asia (-0.68)	South Asia (-0.57)
**female**	Central Sub-Saharan Africa (0.41)	Oceania (0.35)	Eastern Sub-Saharan Africa (0.11)	East Asia (−0.70)	Southeast Asia (−0.63)	South Asia (−0.51)
**male**	Central Sub-Saharan Africa (0.42)	Oceania (0.29)	Eastern Sub-Saharan Africa (0.07)	East Asia (−0.83)	Southeast Asia (−0.72)	Eastern Europe (−0.63)
			**DALY (years)**			
**both**	Central Sub-Saharan Africa (0.41)	Oceania (0.25)	Western Sub-Saharan Africa (0.08)	High-income Asia Pacific (−0.79)	Eastern Europe (−0.79)	Central Europe (−0.78)
**female**	Central Sub-Saharan Africa (0.45)	Oceania (0.29)	Western Sub-Saharan Africa (0.19)	High-income Asia Pacific (−0.77)	Central Europe (−0.77)	Eastern Europe (−0.75)
**male**	Central Sub-Saharan Africa (0.39)	Oceania (0.23)	Western Sub-Saharan Africa (0.01)	High-income Asia Pacific (−0.82)	Eastern Europe (−0.81)	Central Europe (−0.80)
			**EAPC**			
			**Incidence**			
**both**	Oceania (−1.01)	Australasia (−1.07)	Central Sub-Saharan Africa (−1.37)	East Asia (−5.61)	Southeast Asia (−4.64)	South Asia (−4.53)
**female**	Oceania (−0.96)	Australasia (−0.99)	Central Sub-Saharan Africa (−1.29)	East Asia (−4.71)	Southeast Asia (−4.15)	South Asia (−4.08)

**male**	Oceania (−1.04)	Australasia (−1.20)	Central Sub-Saharan Africa (−1.42)	East Asia (−6.18)	Southeast Asia (−5.06)	South Asia (−4.79)
			**DALY**			
**both**	Oceania (−0.81)	Caribbean (−0.89)	Central Sub-Saharan Africa (−1.07)	Eastern Europe (−4.36)	East Asia (−4.07)	Central Europe (−3.83)
**female**	Caribbean (−0.63)	Oceania (−0.72)	Central Sub-Saharan Africa (−0.91)	East Asia (−3.77)	Eastern Europe (−3.62)	Central Europe (−3.46)
**male**	Oceania (−0.87)	Caribbean (−1.12)	Central Sub-Saharan Africa (−1.18)	Eastern Europe (−5.00)	East Asia (−4.26)	Southeast Asia (−4.25)

Abbreviations: DALY, disability adjusted life years; EAPC, estimated annual percentage change; VAD, vitamin A deficiency.

Supplemental Table S2: The top three and the bottom three countries of VAD incidence or DALY.

sex	Top three countries			Bottom three countries		
			**2019 Age-standardized rates (per 100,000 people)**			
			**Age-standardized incidence rate**			
**both**	Somalia (63640.11)	Niger (43501.51)	Micronesia (Federated States of) (34768.53)	Australia (77.34)	France (172.51)	Saudi Arabia (190.04)
**female**	Somalia (54280.58)	Niger (31675.96)	Kenya (29768.57)	Australia (102.23)	France (173.41)	Russian Federation (229.62)
**male**	Somalia (72502.08)	Niger (55451.41)	Central African Republic (47754.69)	Australia (52.46)	Saudi Arabia (148.00)	Russian Federation (167.84)
			**Age-standardized DALY rate**			
**both**	Niger (88.75)	Somalia (80.92)	Mali (73.09)	Australia (0.01)	Russian Federation (0.04)	France (0.05)
**female**	Niger (77.67)	Somalia (75.56)	Mali (60.62)	Australia (0.01)	Russian Federation (0.03)	France (0.05)
**male**	Niger (99.54)	Central African Republic (89.85)	Somalia (86.01)	Australia (0.01)	Russian Federation (0.04)	France (0.06)
			**1990-2019 increase times**			
			**Incidence (cases)**			
**both**	Somalia (1.65)	Afghanistan (1.09)	Niger (0.99)	Saudi Arabia (-0.89)	Thailand (-0.87)	Mauritius (-0.83)
**female**	Somalia (1.68)	Niger (1.04)	Afghanistan (1.01)	Saudi Arabia (-0.87)	Thailand (-0.84)	Iran (Islamic Republic of) (-0.80)
**male**	Somalia (1.63)	Afghanistan (1.14)	Niger (0.96)	Saudi Arabia (-0.92)	Thailand (-0.89)	Mauritius (-0.87)
**DALY (years)**						
**both**	Somalia (1.38)	Afghanistan (1.22)	Niger (1.13)	Saudi Arabia (-0.96)	Iran (Islamic Republic of) (-0.94)	Taiwan (Province of China) (-0.90)
**female**	Somalia (1.50)	Afghanistan (1.34)	Niger (1.19)	Saudi Arabia (-0.95)	Iran (Islamic Republic of) (-0.93)	Republic of Korea (-0.87)

**male**	Somalia (1.29)	Afghanistan (1.13)	Niger (1.08)	Saudi Arabia (-0.97)	Iran (Islamic Republic of) (-0.95)	Republic of Korea (-0.93)
			**EAPC**			
			**Incidence**			
**both**	Kiribati (-0.14)	Georgia (-0.18)	Somalia (-0.30)	Equatorial Guinea (-9.90)	Saudi Arabia (-8.89)	Maldives (-8.07)
**female**	Georgia (-0.07)	Zimbabwe (-0.14)	Kyrgyzstan (-0.28)	Equatorial Guinea (-9.88)	Saudi Arabia (-8.08)	Maldives (-6.93)
**male**	Kiribati (-0.02)	Northern Mariana Islands (-0.14)	Georgia (-0.28)	Equatorial Guinea (-10.09)	Saudi Arabia (-9.63)	Maldives (-9.01)
			**DALY**			
**both**	Northern Mariana Islands (0.14)	Georgia (0.04)	Kiribati (-0.12)	Saudi Arabia (-9.41)	Iran (Islamic Republic of) (-8.73)	Maldives (-7.82)
**female**	Georgia (0.71)	Zimbabwe (0.19)	Kyrgyzstan (0.09)	Saudi Arabia (-8.56)	Iran (Islamic Republic of) (-8.09)	Equatorial Guinea (-7.31)
**male**	Northern Mariana Islands (0.30)	Kiribati (-0.12)	Samoa (-0.47)	Saudi Arabia (-10.04)	Iran (Islamic Republic of) (-9.17)	Maldives (-8.71)

Abbreviations: DALY, disability adjusted life-years; EAPC, estimated annual percentage change; VAD, vitamin A deficiency.

Supplemental Table S3: The incident cases and age-standardized incidence rate of VAD in 1990 and 2019, and its temporal trends from 1990 to 2019.

Nation	Sex	Incident Cases No. (thousands) (95% UI)		Change in absolute number (%)	Age-standardized incidence rate per 100,000 No. (95% UI)		1990-2019 EAPC No. (95% CI)
		1990	2019		1990	2019	
Afghanistan	both	4775.28 (4362.40,5217.51)	9976.08 (8681.04,11360.87)	108.91%	41738.50 (37517.62,46253.42)	26013.77 (22250.86,30088.73)	-1.64 (-2.13,-1.10)
Albania	both	876.83 (777.62,986.21)	304.74 (267.79,347.91)	-65.25%	27225.10 (23823.90,31079.80)	11731.87 (10138.25,13478.61)	-3.49 (-3.80,-3.26)
Algeria	both	2732.04 (2269.73,3238.70)	1104.51 (936.74,1321.37)	-59.57%	10831.30 (8778.67,13077.83)	2690.40 (2223.70,3276.27)	-4.59 (-4.81,-4.40)
American Samoa	both	5.26 (4.37,6.28)	3.18 (2.60,3.87)	-39.54%	11586.42 (9465.90,14131.92)	6562.33 (5256.22,8099.88)	-1.87 (-2.09,-1.59)
Andorra	both	0.32 (0.26,0.38)	0.27 (0.23,0.31)	-15.84%	641.20 (519.84,790.42)	365.05 (300.66,437.47)	-1.62 (-1.83,-1.34)
Angola	both	4813.69 (4400.51,5213.16)	4301.08 (3589.60,5075.60)	-10.65%	46810.03 (42173.80,51407.81)	14328.40 (11671.06,17269.88)	-4.05 (-4.42,-3.58)

Antigua and Barbuda	both	2.50 (2.10,2.99)	1.65 (1.41,1.93)	-34.02%	4345.83 (3579.53,5253.23)	1945.29 (1628.89,2294.08)	-2.83 (-2.96,-2.75)
Argentina	both	3776.97 (3300.63,4316.20)	3372.88 (2896.43,3904.03)	-10.70%	12043.09 (10373.15,13995.08)	7788.85 (6532.28,9183.88)	-1.20 (-1.37,-0.95)
Armenia	both	35.69 (30.52,41.71)	15.06 (12.82,17.44)	-57.82%	1027.40 (851.43,1217.64)	494.58 (414.77,579.73)	-2.77 (-3.09,-2.46)
Australia	both	27.85 (23.95,32.02)	22.92 (19.72,26.69)	-17.70%	134.73 (115.03,157.42)	77.34 (66.04,90.92)	-1.19 (-1.52,-0.70)
Austria	both	85.45 (72.10,102.37)	43.88 (37.50,51.57)	-48.65%	1237.09 (1010.65,1509.17)	565.09 (470.38,691.19)	-2.62 (-2.94,-2.25)
Azerbaijan	both	316.96 (263.14,383.10)	259.78 (219.53,309.08)	-18.04%	4384.91 (3553.61,5436.97)	2574.20 (2141.14,3099.16)	-2.22 (-2.85,-1.63)
Bahamas	both	6.69 (5.60,8.02)	5.22 (4.45,6.01)	-21.93%	2697.29 (2218.62,3284.83)	1434.22 (1203.77,1682.36)	-2.73 (-3.00,-2.48)
Bahrain	both	24.53 (20.34,29.68)	20.75 (17.26,24.96)	-15.41%	4847.01 (3969.50,5928.03)	1459.86 (1201.00,1773.07)	-3.83 (-4.01,-3.66)
Bangladesh	both	20271.82 (17751.52,22730.56)	7439.74 (6423.43,8662.02)	-63.30%	18570.55 (16035.56,21140.18)	4761.95 (4024.38,5647.55)	-4.30 (-4.56,-3.93)
Barbados	both	8.33 (7.03,9.72)	5.71 (4.90,6.55)	-31.45%	3516.08 (2937.04,4153.17)	2031.76 (1719.50,2371.30)	-1.87 (-1.95,-1.80)
Belarus	both	346.81 (296.51,401.61)	121.02 (104.09,141.09)	-65.11%	3435.08 (2895.74,4034.36)	1286.97 (1099.85,1503.09)	-3.56 (-3.80,-3.38)
Belgium	both	107.38 (90.55,128.18)	52.93 (44.77,62.87)	-50.71%	1212.22 (983.73,1497.45)	536.09 (435.82,656.65)	-2.52 (-2.84,-2.15)

Belize	both	23.13 (19.54,27.08)	18.86 (15.99,21.92)	-18.49%	12520.73 (10368.10,14922.13)	4673.41 (3900.08,5506.83)	-3.27 (-3.45,-3.07)
Benin	both	2493.77 (2314.52,2668.33)	3371.32 (2945.63,3825.93)	35.19%	52554.19 (48209.51,56473.67)	27262.28 (23522.25,31358.09)	-2.10 (-2.21,-1.95)
Bermuda	both	1.40 (1.18,1.65)	0.73 (0.63,0.84)	-47.97%	2489.94 (2084.85,2968.59)	1210.93 (1037.32,1416.25)	-2.74 (-2.94,-2.61)
Bhutan	both	153.63 (132.74,174.56)	43.65 (36.94,50.89)	-71.58%	24935.21 (21140.62,28794.53)	5858.63 (4894.76,6905.90)	-5.02 (-5.08,-4.96)
Bolivia (Plurinational State of)	both	881.62 (745.06,1022.13)	960.65 (800.87,1136.80)	8.96%	14311.62 (11955.53,16904.73)	8475.21 (6977.25,10156.55)	-1.60 (-1.85,-1.34)
Bosnia and Herzegovina	both	987.05 (868.09,1105.04)	281.44 (244.98,319.64)	-71.49%	22565.98 (19612.39,25555.57)	8900.14 (7662.50,10266.41)	-3.79 (-4.16,-3.53)
Botswana	both	337.03 (292.02,381.97)	210.16 (175.95,248.60)	-37.64%	26955.48 (23021.64,30718.98)	9632.49 (7920.46,11592.91)	-3.46 (-3.55,-3.38)
Brazil	both	36406.98 (32783.51,40573.16)	21512.08 (18678.12,24696.58)	-40.91%	24855.26 (22230.93,27862.46)	10128.64 (8668.30,11793.54)	-3.15 (-3.26,-3.08)

Brunei Darussalam	both	6.37 (5.22,7.86)	4.44 (3.68,5.35)	-30.34%	2599.10 (2094.28,3299.53)	1112.34 (898.59,1358.55)	-2.95 (-3.02,-2.89)
Bulgaria	both	1164.43 (1016.65,1320.09)	549.14 (478.29,627.28)	-52.84%	14270.98 (12305.35,16448.25)	8299.09 (7160.68,9591.05)	-1.77 (-2.00,-1.55)
Burkina Faso	both	5542.14 (5200.39,5852.94)	6155.17 (5423.17,6998.54)	11.06%	59092.24 (55188.90,62800.70)	27594.06 (23930.23,31725.92)	-2.84 (-2.96,-2.76)
Burundi	both	2021.56 (1798.07,2251.80)	2725.35 (2361.71,3096.37)	34.81%	35621.45 (31235.57,40430.62)	22039.35 (18730.43,25405.87)	-1.99 (-2.24,-1.83)
Cape Verde	both	100.08 (87.00,113.39)	28.18 (23.30,33.91)	-71.84%	29867.69 (25413.07,34527.94)	5248.87 (4294.08,6489.23)	-6.06 (-6.39,-5.78)
Cambodia	both	3661.45 (3285.28,4068.16)	1163.97 (953.06,1409.01)	-68.21%	36548.11 (32378.61,41153.50)	7755.02 (6235.50,9471.09)	-5.38 (-5.50,-5.27)
Cameroon	both	7123.99 (6885.39,7386.50)	7937.38 (6856.36,8973.85)	11.42%	69494.50 (66902.38,72335.40)	27949.82 (23794.23,31961.96)	-3.10 (-3.26,-2.90)
Canada	both	441.21 (367.55,525.97)	262.51 (219.20,316.68)	-40.50%	1921.75 (1552.31,2321.51)	906.47 (739.05,1120.87)	-2.63 (-2.90,-2.38)

Central African Republic	both	1244.76 (1139.67,1346.31)	1759.90 (1571.16,1956.61)	41.39%	45887.48 (41619.73,50082.84)	33496.12 (29500.63,37726.42)	-1.08 (-1.16,-1.01)
Chad	both	3570.26 (3374.90,3759.26)	5550.08 (4869.85,6230.29)	55.45%	60512.81 (56794.85,64216.93)	34260.53 (29897.54,38895.56)	-1.90 (-1.98,-1.78)
Chile	both	997.33 (825.20,1199.05)	642.57 (539.20,757.47)	-35.57%	8303.49 (6752.51,10076.08)	4106.52 (3404.17,4908.80)	-2.25 (-2.39,-2.03)
China	both	122360.77 (103119.20,145575.77)	25263.60 (21390.09,30021.08)	-79.35%	11237.97 (9346.97,13367.94)	2113.34 (1764.06,2553.64)	-5.72 (-5.94,-5.51)
Colombia	both	2750.18 (2331.28,3203.71)	1391.26 (1200.98,1610.77)	-49.41%	8519.54 (7139.49,10048.34)	2925.54 (2459.16,3459.79)	-3.98 (-4.13,-3.85)
Comoros	both	187.87 (170.08,207.58)	141.70 (123.11,160.14)	-24.57%	39801.50 (35484.90,44664.23)	19533.69 (16776.57,22517.14)	-2.59 (-2.77,-2.45)
Congo	both	1165.24 (1074.65,1249.51)	1376.53 (1213.40,1560.45)	18.13%	48889.86 (44674.27,52797.65)	27043.51 (23493.53,30947.28)	-1.84 (-2.12,-1.49)
Cook Islands	both	1.69 (1.40,2.02)	0.57 (0.46,0.69)	-66.37%	9689.90 (7875.33,11787.25)	3858.09 (3068.39,4773.77)	-2.88 (-3.02,-2.70)
Costa Rica	both	224.81 (191.77,265.93)	131.70 (112.87,154.66)	-41.42%	7560.87 (6330.33,9123.12)	2864.12 (2411.40,3417.05)	-3.29 (-3.38,-3.21)

Cote d'Ivoire	both	4565.88 (4119.27,5016.61)	3608.17 (3026.53,4270.28)	-20.98%	38363.96 (34398.72,42372.03)	14240.01 (11642.12,17152.56)	-3.18 (-3.32,-3.00)
Croatia	both	560.44 (488.93,635.20)	300.27 (262.45,341.86)	-46.42%	12052.21 (10446.18,13804.12)	7409.32 (6414.41,8556.06)	-1.91 (-2.16,-1.73)
Cuba	both	429.13 (360.96,501.58)	252.11 (217.89,291.47)	-41.25%	4099.56 (3401.63,4918.89)	2264.94 (1936.66,2660.79)	-2.09 (-2.36,-1.87)
Cyprus	both	17.28 (14.13,21.19)	7.54 (6.35,9.00)	-56.40%	2447.85 (1939.17,3068.31)	645.65 (524.87,791.75)	-4.56 (-5.26,-3.85)
Czechia	both	989.54 (854.89,1145.55)	560.78 (488.73,639.53)	-43.33%	10118.43 (8647.97,11876.24)	5505.39 (4722.66,6331.71)	-1.88 (-2.09,-1.66)
Democratic People's Republic of Korea	both	3373.08 (2870.86,3890.97)	1577.94 (1317.94,1880.80)	-53.22%	17477.55 (14717.58,20450.60)	7105.38 (5858.88,8577.58)	-3.33 (-3.63,-3.07)
Democratic Republic of the Congo	both	16062.68 (14452.32,17695.16)	26160.53 (23199.02,29447.96)	62.87%	42167.42 (37452.40,46894.05)	30146.05 (26304.29,34484.43)	-0.64 (-1.14,0.05)
Denmark	both	38.37 (32.14,45.97)	22.15 (18.93,26.20)	-42.28%	835.93 (680.51,1036.57)	439.59 (359.82,533.72)	-2.02 (-2.27,-1.73)

Djibouti	both	214.93 (193.32,235.71)	202.05 (172.96,232.89)	-5.99%	43788.00 (38861.36,48563.49)	16576.49 (14006.60,19322.85)	-3.54 (-3.78,-3.37)
Dominica	both	3.83 (3.20,4.52)	1.39 (1.19,1.62)	-63.69%	5493.24 (4464.19,6650.06)	2129.11 (1789.02,2528.77)	-3.57 (-3.91,-3.26)
Dominican Re-public	both	1031.70 (908.36,1172.11)	517.32 (442.40,600.01)	-49.86%	14482.28 (12489.04,16636.52)	4835.14 (4044.74,5695.19)	-4.36 (-4.76,-4.07)
Ecuador	both	754.80 (630.43,900.66)	583.17 (476.11,712.59)	-22.74%	8176.85 (6743.18,9826.89)	3770.76 (3036.99,4652.23)	-2.72 (-3.09,-2.41)
Egypt	both	4262.83 (3690.66,4964.00)	1909.49 (1600.08,2270.53)	-55.21%	7674.90 (6504.18,9108.35)	1925.18 (1590.42,2320.36)	-3.85 (-4.17,-3.38)
El Salvador	both	1070.97 (931.21,1220.94)	355.17 (299.21,415.23)	-66.84%	20665.16 (17765.92,23933.94)	5828.65 (4813.20,6986.56)	-4.66 (-5.00,-4.41)
Equatorial Guinea	both	250.24 (234.94,265.84)	63.63 (51.86,78.55)	-74.57%	58996.33 (54861.20,63142.25)	4574.18 (3671.03,5768.95)	-9.90 (-10.55,-9.45)
Eritrea	both	1569.10 (1434.45,1685.11)	1505.04 (1298.81,1719.88)	-4.08%	51808.31 (46756.78,56195.97)	22128.39 (18798.03,25563.98)	-2.88 (-2.96,-2.79)
Estonia	both	29.08 (24.80,33.39)	9.27 (8.04,10.65)	-68.11%	1921.52 (1594.25,2244.59)	713.55 (606.50,827.21)	-3.48 (-3.60,-3.37)

Eswatini	both	207.98 (175.36,237.00)	94.46 (77.51,113.65)	-54.58%	26597.52 (22241.61,30659.07)	8688.37 (7026.23,10730.00)	-3.73 (-3.82,-3.60)
Ethiopia	both	28888.09 (27068.22,30895.89)	26813.28 (23411.56,30832.69)	-7.18%	55371.92 (51310.24,59745.89)	24329.13 (20909.75,28388.42)	-2.90 (-3.31,-2.44)
Fiji	both	127.03 (106.32,149.53)	62.84 (50.34,77.73)	-50.53%	17805.56 (14697.02,21255.37)	7759.07 (6053.25,9738.16)	-2.61 (-2.78,-2.40)
Finland	both	49.47 (41.42,59.81)	25.10 (21.47,29.56)	-49.26%	1108.26 (890.36,1390.37)	527.55 (434.14,645.28)	-2.45 (-2.70,-2.17)
France	both	139.37 (118.83,165.87)	101.60 (86.30,119.65)	-27.10%	259.59 (214.88,316.19)	172.51 (141.66,210.35)	-0.88 (-1.09,-0.60)
Gabon	both	207.40 (178.34,238.78)	78.19 (64.96,95.23)	-62.30%	21581.20 (18230.11,25152.45)	4611.67 (3770.01,5688.78)	-4.99 (-5.11,-4.83)
Gambia	both	522.65 (487.36,557.18)	499.98 (431.09,579.59)	-4.34%	53208.58 (49111.21,56989.16)	22716.80 (19313.06,26722.24)	-2.93 (-2.96,-2.90)
Georgia	both	143.44 (121.66,167.63)	82.67 (70.75,96.39)	-42.36%	2669.14 (2215.21,3174.29)	2335.21 (1971.62,2798.31)	-0.18 (-0.85,0.56)
Germany	both	373.04 (318.04,436.56)	249.90 (214.17,290.69)	-33.01%	509.56 (424.59,606.81)	334.98 (282.36,399.44)	-1.37 (-1.46,-1.28)
Ghana	both	6913.37 (6355.04,7456.23)	4824.13 (4035.05,5629.56)	-30.22%	47086.90 (42907.88,51165.73)	15915.73 (13097.57,18795.72)	-3.71 (-3.95,-3.49)

Greece	both	164.25 (136.71,198.19)	68.05 (58.53,80.48)	-58.57%	1791.29 (1445.31,2242.77)	762.96 (630.88,932.89)	-2.64 (-3.15,-2.11)
Greenland	both	0.71 (0.59,0.85)	0.48 (0.40,0.57)	-32.89%	1363.51 (1110.18,1646.06)	977.16 (797.95,1206.44)	-1.79 (-2.25,-1.41)
Grenada	both	9.78 (8.22,11.50)	3.58 (3.05,4.21)	-63.34%	12004.06 (9864.51,14311.21)	3614.87 (3008.89,4298.71)	-3.64 (-4.06,-3.19)
Guam	both	7.69 (6.31,9.36)	4.49 (3.64,5.44)	-41.63%	6082.49 (4921.61,7508.03)	3113.35 (2469.01,3874.46)	-2.60 (-2.82,-2.38)
Guatemala	both	1545.56 (1356.30,1756.91)	1051.75 (897.13,1243.23)	-31.95%	19030.28 (16481.51,21961.74)	5880.07 (4913.62,7117.03)	-3.95 (-4.17,-3.73)
Guinea	both	3113.29 (2902.02,3326.44)	3036.29 (2640.70,3462.09)	-2.47%	51724.96 (47844.88,55617.58)	24602.80 (20937.38,28472.22)	-2.46 (-2.54,-2.36)
Guinea-Bissau	both	567.13 (534.74,598.52)	510.80 (445.63,581.89)	-9.93%	57411.84 (53698.83,60960.37)	27544.15 (23660.00,31740.50)	-2.43 (-2.48,-2.36)
Guyana	both	99.80 (84.57,117.07)	37.10 (31.54,43.37)	-62.83%	13281.65 (11114.42,15793.08)	4953.90 (4161.42,5881.25)	-3.26 (-3.34,-3.13)
Haiti	both	1663.67 (1453.62,1879.27)	1668.65 (1444.60,1925.68)	0.30%	26475.93 (22772.06,30289.15)	13512.71 (11514.77,15853.40)	-2.41 (-2.47,-2.36)

Honduras	both	687.02 (589.73,791.47)	585.32 (501.16,682.24)	-14.80%	14344.21 (12092.59,16940.56)	5901.58 (4946.86,7014.21)	-3.06 (-3.28,-2.87)
Hungary	both	1144.81 (994.80,1293.56)	619.02 (540.30,700.15)	-45.93%	11607.90 (9936.98,13385.80)	6651.65 (5727.19,7631.89)	-1.90 (-1.97,-1.85)
Iceland	both	2.81 (2.30,3.45)	1.52 (1.28,1.80)	-46.10%	1218.22 (969.58,1536.36)	500.95 (414.12,608.70)	-2.91 (-3.16,-2.63)
India	both	250998.24 (222317.51,278623.10)	110835.61 (94078.89,130591.37)	-55.84%	29243.89 (25640.64,32812.48)	8129.88 (6844.02,9719.21)	-4.39 (-4.68,-4.00)
Indonesia	both	48858.71 (43522.08,54657.97)	13341.45 (11204.02,15633.10)	-72.69%	27720.45 (24314.18,31288.30)	5770.57 (4762.10,6863.66)	-5.07 (-5.15,-4.96)
Iran (Islamic Republic of)	both	6380.16 (5501.50,7379.38)	1105.55 (942.03,1334.90)	-82.67%	11013.23 (9319.16,12973.80)	1344.70 (1118.86,1651.79)	-7.05 (-7.67,-6.52)
Iraq	both	2498.65 (2126.11,2909.93)	1337.63 (1114.03,1602.38)	-46.47%	14157.60 (11755.15,16889.98)	3209.16 (2619.10,3937.86)	-5.16 (-5.51,-4.76)
Ireland	both	42.12 (34.74,50.82)	20.63 (17.08,24.45)	-51.03%	1297.52 (1037.55,1609.76)	479.65 (383.67,580.43)	-2.93 (-3.31,-2.42)
Israel	both	413.07 (349.50,496.71)	341.46 (271.01,427.28)	-17.34%	9766.40 (8128.09,11842.75)	4414.10 (3439.74,5554.15)	-2.57 (-2.95,-2.11)

Italy	both	1837.45 (1616.01,2117.77)	971.79 (840.32,1123.30)	-47.11%	2924.52 (2492.36,3445.67)	1415.51 (1206.43,1660.52)	-2.20 (-2.65,-1.70)
Jamaica	both	145.31 (123.31,170.82)	67.63 (58.40,77.98)	-53.46%	6429.07 (5306.24,7675.66)	2504.46 (2122.73,2927.25)	-3.25 (-3.45,-3.09)
Japan	both	1114.78 (940.28,1335.53)	732.25 (627.73,848.74)	-34.31%	1020.90 (839.54,1260.79)	712.49 (592.15,851.59)	-1.02 (-1.13,-0.87)
Jordan	both	537.72 (458.78,622.74)	575.23 (487.42,683.78)	6.98%	14577.81 (12277.11,17103.79)	5045.49 (4187.36,6131.25)	-3.70 (-4.07,-3.29)
Kazakhstan	both	2532.27 (2164.48,2923.26)	1351.55 (1134.77,1601.01)	-46.63%	15992.83 (13361.33,18737.95)	7611.97 (6200.30,9212.28)	-2.52 (-2.59,-2.45)
Kenya	both	12874.27 (11937.88,13753.01)	14836.61 (13161.36,16772.92)	15.24%	55426.80 (50875.72,59654.14)	29712.62 (26085.15,33973.70)	-2.02 (-2.19,-1.82)
Kiribati	both	21.92 (19.41,24.73)	28.65 (24.82,32.75)	30.70%	31237.24 (27298.53,35627.58)	26227.13 (22408.78,30289.12)	-0.14 (-0.33,0.09)
Kuwait	both	38.96 (32.00,46.83)	32.54 (27.57,39.14)	-16.49%	2226.26 (1809.88,2713.22)	745.67 (619.93,912.18)	-4.28 (-4.57,-4.05)
Kyrgyzstan	both	262.90 (217.18,315.07)	285.56 (239.94,336.78)	8.62%	5983.72 (4827.70,7260.36)	4423.49 (3637.70,5355.66)	-0.57 (-0.82,-0.27)

Lao People's Democratic Republic	both	1490.14 (1351.12,1616.53)	792.53 (667.70,921.53)	-46.82%	36868.53 (33077.50,40334.17)	11596.34 (9592.97,13603.04)	-4.00 (-4.33,-3.66)
Latvia	both	45.19 (39.00,52.38)	14.39 (12.45,16.66)	-68.16%	1764.69 (1497.95,2073.51)	757.10 (642.82,891.09)	-2.90 (-3.03,-2.79)
Lebanon	both	229.24 (194.02,270.88)	85.32 (72.50,101.03)	-62.78%	7051.48 (5831.48,8495.46)	1682.65 (1399.46,2043.83)	-4.78 (-4.95,-4.59)
Lesotho	both	665.34 (601.54,731.35)	303.76 (261.85,355.94)	-54.35%	38818.57 (34746.42,43118.08)	15405.14 (13011.94,18346.03)	-2.98 (-3.08,-2.83)
Liberia	both	681.75 (615.65,745.31)	640.23 (526.49,761.23)	-6.09%	35237.77 (31333.29,39053.75)	13461.13 (10787.56,16479.80)	-3.69 (-4.06,-3.38)
Libya	both	325.73 (269.67,384.40)	121.05 (101.64,144.47)	-62.84%	7697.01 (6236.35,9246.15)	1829.16 (1515.35,2213.54)	-4.60 (-5.34,-3.89)
Lithuania	both	65.12 (55.40,76.34)	18.91 (16.41,21.82)	-70.96%	1833.96 (1526.99,2195.02)	681.81 (582.95,800.97)	-3.53 (-3.63,-3.47)
Luxembourg	both	2.56 (2.14,3.07)	2.22 (1.86,2.61)	-13.13%	751.09 (612.98,935.92)	408.17 (331.07,494.71)	-1.95 (-2.19,-1.69)
Madagascar	both	4644.95 (4181.88,5142.59)	5945.04 (5192.21,6751.07)	27.99%	38273.00 (33882.49,42871.88)	21864.71 (18803.93,25124.57)	-1.95 (-2.22,-1.69)
Malawi	both	5104.26 (4777.40,5447.28)	4866.23 (4255.50,5529.77)	-4.66%	52642.85 (48715.22,56769.39)	25987.25 (22397.78,29970.98)	-2.63 (-2.89,-2.38)

Malaysia	both	622.78 (515.47,750.70)	156.27 (129.45,188.10)	-74.91%	3706.16 (3020.66,4519.82)	546.71 (445.28,669.52)	-6.23 (-6.84,-5.58)
Maldives	both	67.23 (58.52,76.47)	13.22 (10.72,15.82)	-80.34%	31634.51 (27138.83,36344.85)	2948.44 (2340.18,3559.24)	-8.07 (-8.58,-7.59)
Mali	both	4980.77 (4686.09,5260.93)	6550.94 (5680.12,7399.01)	31.52%	58645.98 (54848.22,62269.59)	30561.22 (26304.47,35008.25)	-2.34 (-2.40,-2.30)
Malta	both	8.05 (6.60,9.98)	2.89 (2.47,3.46)	-64.07%	2420.77 (1936.29,3078.98)	759.61 (627.82,942.10)	-3.75 (-4.10,-3.34)
Marshall Islands	both	21.50 (19.54,23.38)	12.09 (10.46,13.90)	-43.76%	47649.82 (42740.66,52278.68)	22663.67 (19353.74,26326.44)	-2.44 (-2.49,-2.38)
Mauritania	both	653.34 (575.71,727.04)	404.04 (336.00,485.77)	-38.16%	32713.43 (28684.76,36674.26)	10418.29 (8536.49,12756.34)	-3.81 (-3.88,-3.71)
Mauritius	both	79.71 (66.57,94.64)	13.49 (11.14,16.27)	-83.08%	7952.65 (6549.99,9554.50)	1311.46 (1059.56,1609.53)	-6.00 (-6.36,-5.61)
Mexico	both	14604.05 (12592.39,16686.06)	8293.91 (6967.59,9727.16)	-43.21%	18098.54 (15498.68,20835.04)	7181.88 (5990.60,8558.13)	-3.23 (-3.32,-3.15)
Micronesia (Federated States of)	both	53.16 (49.08,57.08)	34.70 (30.93,38.43)	-34.72%	51663.11 (47261.08,55869.48)	34768.53 (30793.63,38842.13)	-1.26 (-1.33,-1.19)
Monaco	both	0.13 (0.12,0.16)	0.10 (0.08,0.11)	-27.39%	497.97 (414.34,609.64)	301.60 (249.02,367.06)	-1.47 (-1.71,-1.17)

Mongolia	both	112.37 (91.28,135.90)	41.90 (35.06,50.01)	-62.72%	5219.20 (4143.16,6446.50)	1242.08 (1020.41,1511.34)	-4.84 (-5.10,-4.63)
Montenegro	both	57.38 (49.46,65.97)	37.83 (33.13,43.01)	-34.08%	9551.54 (8116.63,11129.91)	6333.41 (5458.87,7256.33)	-2.00 (-2.64,-1.50)
Morocco	both	5074.28 (4390.58,5824.56)	2140.59 (1798.00,2534.39)	-57.81%	20349.67 (17273.51,23793.99)	6117.33 (5055.53,7318.74)	-3.96 (-4.15,-3.70)
Mozambique	both	8287.29 (7829.86,8732.12)	8598.57 (7570.06,9772.11)	3.76%	62889.25 (58775.02,66874.12)	28865.43 (24964.81,33338.42)	-2.90 (-3.08,-2.73)
Myanmar	both	12906.24 (11508.97,14399.64)	2536.98 (2068.91,3082.47)	-80.34%	33564.63 (29582.98,37834.30)	5309.21 (4283.05,6548.45)	-6.73 (-6.99,-6.55)
Namibia	both	209.75 (177.59,244.92)	148.58 (123.40,178.17)	-29.16%	15549.28 (12924.99,18449.26)	6569.44 (5370.48,7995.87)	-3.02 (-3.22,-2.71)
Nauru	both	1.69 (1.42,2.00)	1.12 (0.92,1.33)	-33.75%	17234.45 (14349.92,20668.14)	11349.06 (9161.41,13719.79)	-0.96 (-1.57,-0.11)
Nepal	both	4310.40 (3843.65,4859.69)	1703.36 (1450.80,1981.05)	-60.48%	22064.43 (19326.35,25177.77)	5693.31 (4776.57,6728.39)	-4.84 (-5.02,-4.69)
Netherlands	both	121.02 (101.14,144.45)	71.47 (60.43,84.66)	-40.94%	902.11 (728.80,1114.56)	481.15 (395.01,594.34)	-1.81 (-2.07,-1.49)
New Zealand	both	23.34 (19.83,27.51)	21.56 (18.16,25.17)	-7.65%	741.94 (620.11,895.66)	541.36 (444.73,650.73)	-0.73 (-0.92,-0.44)

Nicaragua	both	415.99 (353.69,484.49)	119.16 (101.80,140.18)	-71.35%	10557.36 (8799.71,12562.76)	1750.25 (1455.03,2099.56)	-6.74 (-7.33,-6.29)
Niger	both	5052.87 (4794.86,5318.85)	10054.06 (9095.25,11010.93)	98.98%	63679.09 (60043.29,67480.10)	43501.51 (38967.65,48083.42)	-1.22 (-1.28,-1.12)
Nigeria	both	15849.74 (13899.11,17835.86)	11240.75 (9455.16,13180.07)	-29.08%	17500.20 (15142.12,20062.77)	5128.40 (4206.23,6114.31)	-4.35 (-5.00,-3.73)
Niue	both	0.28 (0.23,0.33)	0.08 (0.06,0.10)	-71.99%	13632.06 (11012.60,16441.52)	5712.04 (4530.45,7092.96)	-3.03 (-3.13,-2.94)
North Macedonia	both	496.66 (439.48,556.52)	265.63 (234.48,301.92)	-46.52%	25539.89 (22249.31,29042.24)	12794.14 (11158.74,14829.76)	-2.75 (-2.99,-2.58)
Northern Mariana Islands	both	2.36 (1.98,2.85)	1.43 (1.16,1.76)	-39.66%	5523.04 (4544.63,6759.48)	4083.04 (3267.32,5089.13)	-0.38 (-0.61,-0.04)
Norway	both	34.37 (28.53,41.56)	18.64 (15.77,22.45)	-45.76%	908.63 (723.71,1131.83)	399.47 (324.37,499.28)	-2.71 (-2.98,-2.38)
Oman	both	259.46 (216.36,308.66)	59.23 (48.45,74.11)	-77.17%	13315.45 (10911.76,16069.30)	1300.80 (1050.09,1633.51)	-7.54 (-8.23,-6.81)

Pakistan	both	23561.27 (20896.06,26306.62)	7910.72 (6641.71,9387.38)	-66.42%	20976.55 (18328.11,23917.07)	3521.68 (2883.57,4256.64)	-6.13 (-6.51,-5.79)
Palau	both	1.57 (1.30,1.90)	0.77 (0.64,0.94)	-50.84%	11098.67 (9032.52,13708.84)	5160.58 (4154.47,6343.55)	-2.27 (-2.41,-2.10)
Palestine	both	876.75 (788.83,962.85)	266.72 (222.10,324.43)	-69.58%	42555.10 (37960.59,47276.60)	5435.63 (4444.95,6784.06)	-6.82 (-7.25,-6.30)
Panama	both	151.49 (129.14,177.07)	114.55 (97.31,133.40)	-24.38%	6425.75 (5339.84,7730.69)	2811.88 (2339.63,3337.04)	-2.87 (-3.14,-2.61)
Papua New Guinea	both	676.57 (572.99,781.14)	1116.85 (938.50,1325.62)	65.07%	16770.27 (14040.01,19627.26)	11735.76 (9736.37,14094.30)	-0.65 (-0.98,-0.20)
Paraguay	both	603.93 (514.02,704.83)	421.01 (356.20,497.84)	-30.29%	15515.88 (12995.86,18286.38)	6379.22 (5318.97,7614.22)	-2.80 (-2.90,-2.65)
Peru	both	3090.53 (2704.19,3509.25)	1996.09 (1695.17,2321.16)	-35.41%	14628.81 (12655.96,16754.45)	6087.75 (5098.07,7231.62)	-3.19 (-3.40,-3.01)
Philippines	both	11470.12 (10026.19,13033.14)	9410.61 (7882.35,11178.71)	-17.96%	18912.99 (16316.16,21842.89)	9309.22 (7668.95,11135.15)	-1.80 (-2.13,-1.33)

Poland	both	4907.17 (4312.74,5609.63)	2198.54 (1895.06,2522.72)	-55.20%	13436.32 (11586.07,15560.32)	5933.51 (5066.37,6864.50)	-2.82 (-2.85,-2.79)
Portugal	both	281.09 (231.90,335.36)	93.64 (79.27,108.36)	-66.69%	3123.19 (2496.56,3881.91)	1015.76 (829.01,1219.85)	-3.36 (-3.79,-2.84)
Puerto Rico	both	97.40 (82.77,115.18)	39.62 (34.25,45.57)	-59.32%	2862.48 (2384.62,3460.16)	1204.48 (1024.81,1419.38)	-3.20 (-3.31,-3.11)
Qatar	both	16.33 (13.18,20.16)	20.59 (16.45,26.08)	26.05%	3681.26 (2955.68,4576.46)	725.76 (575.06,927.36)	-5.24 (-5.52,-4.96)
Republic of Korea	both	905.25 (732.66,1099.30)	281.66 (241.22,330.92)	-68.89%	2229.59 (1767.28,2746.11)	620.71 (521.64,750.32)	-4.18 (-4.38,-3.95)
Republic of Moldova	both	155.33 (133.10,180.22)	67.37 (57.75,77.70)	-56.63%	3592.49 (3015.95,4221.53)	1857.99 (1571.52,2177.42)	-2.39 (-2.77,-2.01)
Romania	both	3202.41 (2826.10,3629.40)	1317.90 (1151.97,1499.12)	-58.85%	14416.55 (12485.07,16617.84)	7124.73 (6167.45,8178.26)	-2.55 (-2.73,-2.44)
Russian Federation	both	677.53 (583.08,787.27)	304.60 (262.63,351.58)	-55.04%	430.48 (360.67,513.97)	199.50 (169.93,233.04)	-2.47 (-2.59,-2.39)
Rwanda	both	2457.87 (2202.89,2763.32)	1872.25 (1610.52,2151.67)	-23.83%	33886.51 (29955.11,38408.29)	14592.39 (12290.56,17073.35)	-3.12 (-3.60,-2.69)

Saint Kitts and Nevis	both	2.11 (1.73,2.52)	1.10 (0.92,1.29)	-47.99%	5395.75 (4355.17,6550.94)	1914.57 (1601.52,2266.93)	-3.44 (-3.66,-3.24)
Saint Lucia	both	9.77 (8.25,11.39)	5.02 (4.36,5.77)	-48.61%	7373.42 (6092.32,8725.54)	3007.21 (2581.26,3490.05)	-2.84 (-3.09,-2.57)
Saint Vincent and the Grenadines	both	12.48 (10.56,14.64)	4.31 (3.68,5.00)	-65.44%	11757.70 (9765.67,14066.14)	3982.82 (3363.28,4724.69)	-3.69 (-3.94,-3.48)
Samoa	both	36.14 (30.90,42.05)	34.09 (28.64,40.08)	-5.67%	23699.67 (19976.97,27726.20)	17957.84 (14963.12,21397.02)	-0.68 (-0.85,-0.48)
San Marino	both	0.17 (0.14,0.21)	0.13 (0.11,0.15)	-24.52%	806.09 (654.47,1007.40)	443.71 (366.66,532.09)	-1.84 (-2.24,-1.38)
Sao Tome and Principe	both	56.43 (51.77,60.51)	26.28 (21.93,31.43)	-53.44%	47964.79 (43503.51,51762.27)	13261.50 (10897.33,16156.53)	-4.56 (-4.68,-4.46)
Saudi Arabia	both	630.81 (513.24,796.58)	66.90 (56.04,78.99)	-89.39%	3938.50 (3123.99,5166.26)	190.04 (156.49,229.04)	-8.89 (-9.73,-7.77)
Senegal	both	4153.44 (3964.06,4347.79)	2127.53 (1775.91,2587.14)	-48.78%	55613.66 (52913.84,58495.64)	14427.53 (11765.99,17808.84)	-4.34 (-4.81,-3.81)
Serbia	both	2680.00 (2411.03,2954.33)	1333.72 (1190.44,1491.13)	-50.23%	29608.87 (26346.09,32995.87)	15871.77 (13945.39,17944.52)	-2.36 (-2.64,-2.15)

Seychelles	both	4.79 (3.91,5.77)	1.17 (0.97,1.42)	-75.56%	7290.95 (5850.61,8946.31)	1370.17 (1121.33,1707.77)	-5.35 (-5.95,-4.69)
Sierra Leone	both	1822.19 (1698.96,1934.25)	1884.54 (1620.24,2184.07)	3.42%	51178.08 (47341.09,54712.22)	23318.25 (19733.15,27130.19)	-2.63 (-2.76,-2.48)
Singapore	both	53.22 (44.04,63.37)	32.84 (27.76,38.56)	-38.29%	1904.62 (1556.03,2332.80)	665.95 (552.46,796.38)	-3.64 (-3.73,-3.56)
Slovakia	both	650.17 (566.69,739.16)	317.62 (277.25,363.46)	-51.15%	12906.67 (11039.81,14864.13)	6072.27 (5178.87,7025.26)	-2.53 (-2.63,-2.44)
Slovenia	both	156.60 (135.51,179.21)	96.66 (84.10,110.00)	-38.28%	8314.01 (7057.10,9661.67)	4867.49 (4198.27,5622.75)	-1.86 (-1.95,-1.81)
Solomon Islands	both	139.01 (124.50,153.08)	171.56 (147.47,195.43)	23.42%	42483.37 (37675.48,47221.51)	27989.22 (23742.88,32287.78)	-1.16 (-1.32,-0.93)
Somalia	both	4912.21 (4641.49,5160.23)	13022.65 (12234.85,13758.47)	165.11%	68419.71 (64136.36,72391.35)	63640.11 (59279.85,67657.56)	-0.30 (-0.35,-0.28)
South Africa	both	4497.31 (3854.44,5188.03)	2013.63 (1658.92,2423.86)	-55.23%	13497.22 (11455.44,15717.15)	4066.68 (3287.26,4986.81)	-4.30 (-4.42,-4.21)
South Sudan	both	2272.33 (2026.82,2511.90)	1917.04 (1655.24,2218.51)	-15.64%	38239.96 (33691.41,42712.08)	20173.61 (17093.99,23709.22)	-2.35 (-2.55,-2.18)
Spain	both	642.58 (544.33,750.36)	274.10 (238.97,320.23)	-57.34%	1827.61 (1507.51,2187.46)	667.86 (566.16,800.20)	-2.89 (-3.33,-2.40)

Sri Lanka	both	2731.81 (2373.93,3127.23)	655.63 (553.14,789.31)	-76.00%	17121.08 (14723.18,19848.21)	3499.45 (2892.14,4281.62)	-5.69 (-5.89,-5.52)
Sudan	both	7410.19 (6682.41,8221.31)	3681.59 (3088.14,4399.98)	-50.32%	36394.37 (32214.54,40852.56)	9067.55 (7431.24,11071.48)	-4.54 (-4.81,-4.19)
Suriname	both	37.34 (31.61,43.74)	23.89 (20.21,27.63)	-36.01%	10037.90 (8321.20,11955.45)	4317.26 (3567.13,5090.38)	-3.17 (-3.36,-3.04)
Sweden	both	86.40 (72.87,103.32)	45.02 (38.25,53.32)	-47.89%	1137.60 (931.37,1419.86)	507.84 (415.44,622.63)	-2.52 (-2.94,-2.04)
Switzerland	both	32.85 (27.67,39.21)	24.71 (21.11,29.11)	-24.78%	535.46 (435.30,658.35)	322.82 (264.39,396.45)	-1.64 (-1.85,-1.42)
Syrian Arab Republic	both	1830.52 (1526.77,2164.00)	529.82 (446.33,624.31)	-71.06%	14119.53 (11651.75,17057.54)	3722.36 (3067.32,4442.22)	-4.62 (-4.90,-4.38)
Taiwan (Province of China)	both	596.23 (481.74,727.84)	122.02 (102.64,145.88)	-79.53%	3253.15 (2589.96,4026.64)	647.07 (531.99,785.23)	-5.51 (-5.82,-5.25)
Tajikistan	both	709.22 (608.12,822.72)	766.52 (646.82,893.41)	8.08%	13136.70 (11104.85,15449.22)	8084.77 (6676.17,9556.33)	-1.69 (-2.31,-1.11)
Thailand	both	8028.82 (6964.97,9153.46)	1040.55 (895.29,1226.26)	-87.04%	15181.10 (13069.42,17425.37)	1594.68 (1341.93,1918.04)	-7.44 (-7.95,-6.87)

Timor-Leste	both	273.40 (245.23,300.83)	100.36 (82.41,123.10)	-63.29%	36026.69 (31926.88,40171.66)	8083.78 (6551.79,10002.27)	-6.00 (-6.51,-5.63)
Togo	both	1668.35 (1539.03,1803.82)	1443.36 (1228.06,1668.98)	-13.49%	46624.37 (42655.95,50759.67)	18885.65 (15809.46,22054.03)	-2.83 (-2.94,-2.67)
Tokelau	both	0.38 (0.33,0.44)	0.13 (0.10,0.15)	-67.33%	24927.47 (20985.29,28770.76)	10190.53 (8207.30,12598.05)	-3.02 (-3.18,-2.85)
Tonga	both	20.60 (17.60,24.02)	11.40 (9.35,13.64)	-44.68%	22923.69 (19285.14,27024.53)	12528.03 (10191.31,15246.97)	-1.73 (-1.91,-1.48)
Trinidad and Tobago	both	54.82 (45.76,65.11)	26.25 (22.84,30.11)	-52.12%	4752.07 (3899.92,5745.81)	1982.99 (1691.94,2315.83)	-3.48 (-3.75,-3.29)
Tunisia	both	691.74 (578.21,820.71)	202.04 (173.83,233.62)	-70.79%	8202.75 (6753.36,10020.21)	1754.96 (1468.02,2071.49)	-5.23 (-5.57,-4.93)
Turkey	both	8301.12 (7435.59,9192.89)	2574.87 (2219.00,2951.85)	-68.98%	13891.16 (12333.73,15509.10)	3184.30 (2713.45,3724.76)	-5.20 (-5.40,-5.05)
Turkmenistan	both	210.16 (175.82,252.53)	113.81 (95.83,135.39)	-45.85%	5700.14 (4653.31,7018.33)	2273.88 (1881.51,2740.36)	-3.44 (-3.78,-3.01)
Tuvalu	both	2.50 (2.21,2.83)	1.56 (1.32,1.84)	-37.73%	29113.62 (25597.73,33303.10)	14979.88 (12572.78,17994.06)	-1.88 (-2.02,-1.69)

Uganda	both	5658.48 (5067.05,6280.42)	4903.51 (4227.63,5671.22)	-13.34%	31699.60 (27839.90,35685.77)	11785.58 (10055.06,13818.26)	-3.60 (-3.80,-3.40)
Ukraine	both	1083.44 (932.72,1254.52)	589.96 (510.48,688.59)	-45.55%	2138.51 (1807.21,2517.91)	1357.39 (1158.01,1590.47)	-1.67 (-1.85,-1.54)
United Arab Emirates	both	60.35 (49.59,72.88)	66.51 (52.58,84.09)	10.21%	3233.20 (2620.11,3937.19)	729.84 (578.07,919.20)	-4.80 (-5.38,-4.29)
United Kingdom	both	661.16 (552.62,788.44)	350.03 (296.87,416.25)	-47.06%	1297.05 (1045.11,1600.56)	598.87 (485.27,737.25)	-2.40 (-2.73,-2.01)
United Republic of Tanzania	both	7859.86 (7052.81,8714.09)	8066.02 (7004.44,9196.33)	2.62%	29513.24 (26147.24,33084.72)	13929.63 (11912.06,16214.66)	-2.36 (-2.56,-2.10)
United States Virgin Islands	both	3.25 (2.75,3.80)	1.05 (0.90,1.22)	-67.71%	3211.74 (2669.66,3806.31)	1070.23 (903.61,1256.16)	-4.01 (-4.33,-3.76)
United States of America	both	1560.81 (1319.55,1810.10)	1255.48 (1046.54,1492.77)	-19.56%	690.86 (574.02,816.65)	440.08 (357.09,541.27)	-1.80 (-2.10,-1.52)
Uruguay	both	230.05 (189.00,274.38)	148.78 (124.25,177.42)	-35.33%	8558.62 (6883.71,10362.04)	5121.55 (4190.82,6222.50)	-1.58 (-1.78,-1.29)
Uzbekistan	both	1688.14 (1367.89,2031.21)	992.65 (843.69,1180.41)	-41.20%	8103.98 (6441.04,9928.09)	2979.09 (2484.75,3597.28)	-3.37 (-3.54,-3.16)

Vanuatu	both	59.45 (53.22,65.52)	76.22 (65.95,86.42)	28.21%	41096.99 (36427.90,45759.29)	28080.41 (24054.27,32084.44)	-1.18 (-1.28,-1.06)
Venezuela (Bolivarian Republic of)	both	1637.63 (1403.48,1889.15)	1500.27 (1274.20,1738.99)	-8.39%	8781.57 (7390.27,10405.98)	5461.03 (4549.23,6476.00)	-1.31 (-1.67,-0.90)
Viet Nam	both	5671.53 (4750.43,6662.52)	1319.98 (1022.96,1742.15)	-76.73%	9302.63 (7714.63,11115.89)	1674.85 (1280.32,2240.90)	-5.28 (-5.52,-4.96)
Yemen	both	6164.77 (5622.50,6737.43)	4727.58 (3968.59,5544.23)	-23.31%	44607.55 (40223.61,49336.31)	15059.25 (12423.07,17887.40)	-4.49 (-4.87,-4.22)
Zambia	both	3113.40 (2820.84,3434.26)	2543.62 (2172.32,2976.66)	-18.30%	38626.28 (34505.56,43029.17)	13680.37 (11466.73,16283.86)	-3.76 (-4.27,-3.24)
Zimbabwe	both	2959.79 (2609.88,3317.29)	2882.55 (2483.77,3296.38)	-2.61%	29228.50 (25410.97,33119.76)	19669.56 (16646.11,22884.43)	-0.61 (-0.90,-0.17)
Afghanistan	male	2795.87 (2450.70,3161.69)	5989.14 (4879.52,7182.56)	114.21%	49428.14 (42176.54,57025.67)	30358.71 (24238.10,37178.78)	-1.68 (-2.15,-1.16)

Albania	male	408.60 (329.11,494.28)	101.46 (79.32,128.82)	-75.17%	25351.67 (20067.71,31135.03)	8519.10 (6450.19,11010.89)	-4.47 (-4.86,-4.20)
Algeria	male	1710.70 (1307.52,2172.02)	648.13 (493.63,837.23)	-62.11%	13353.20 (9906.53,17327.97)	3100.83 (2277.76,4125.29)	-4.84 (-5.05,-4.67)
American Samoa	male	3.19 (2.40,4.18)	1.94 (1.43,2.60)	-39.25%	13700.56 (10036.16,18067.34)	7948.49 (5716.70,10809.80)	-1.75 (-2.00,-1.44)
Andorra	male	0.16 (0.12,0.21)	0.13 (0.10,0.16)	-20.19%	608.82 (429.37,828.16)	339.06 (247.04,462.58)	-1.80 (-2.00,-1.57)
Angola	male	3060.95 (2721.46,3382.44)	2583.82 (1979.18,3318.62)	-15.59%	58944.18 (51362.72,66260.34)	17590.09 (13019.16,23295.38)	-4.17 (-4.54,-3.71)
Antigua and Barbuda	male	1.17 (0.86,1.52)	0.69 (0.53,0.89)	-40.71%	4223.68 (2970.18,5636.08)	1699.82 (1243.65,2209.62)	-3.24 (-3.41,-3.13)
Argentina	male	2192.84 (1775.67,2657.28)	1932.67 (1529.49,2386.80)	-11.86%	14210.82 (11293.99,17570.70)	9085.53 (6921.29,11510.19)	-1.23 (-1.43,-0.97)
Armenia	male	17.08 (12.92,22.07)	5.90 (4.54,7.57)	-65.45%	1012.91 (749.71,1352.38)	407.10 (305.45,543.13)	-3.53 (-3.94,-3.20)
Australia	male	9.86 (7.57,12.48)	7.65 (6.07,9.62)	-22.38%	98.04 (74.22,126.55)	52.46 (41.29,65.94)	-1.56 (-1.89,-1.09)
Austria	male	44.81 (33.81,59.36)	21.75 (16.66,28.76)	-51.47%	1326.28 (957.35,1821.50)	560.84 (408.52,780.94)	-2.99 (-3.32,-2.63)

Azerbaijan	male	183.13 (135.19,242.79)	141.36 (107.52,180.90)	-22.81%	5212.70 (3714.33,7108.94)	2814.87 (2096.31,3723.54)	-2.60 (-3.33,-1.93)
Bahamas	male	3.07 (2.27,4.12)	2.13 (1.63,2.74)	-30.59%	2539.91 (1815.80,3476.88)	1226.75 (904.69,1620.00)	-3.13 (-3.45,-2.85)
Bahrain	male	14.73 (11.10,19.44)	12.50 (9.24,16.59)	-15.11%	5026.39 (3712.47,6762.29)	1410.65 (1044.08,1867.69)	-4.06 (-4.24,-3.88)
Bangladesh	male	11173.97 (9100.99,13453.57)	3282.78 (2511.84,4157.86)	-70.62%	19991.75 (16217.53,24231.52)	4262.08 (3154.91,5694.72)	-4.94 (-5.21,-4.53)
Barbados	male	3.87 (2.89,4.97)	2.43 (1.88,3.11)	-37.11%	3400.88 (2477.70,4511.11)	1827.38 (1378.42,2373.79)	-2.11 (-2.22,-2.03)
Belarus	male	143.71 (109.80,186.04)	35.91 (27.41,47.83)	-75.01%	3100.20 (2298.40,4091.33)	866.71 (640.86,1187.32)	-4.66 (-4.91,-4.49)
Belgium	male	55.98 (42.29,76.03)	25.25 (19.43,33.43)	-54.90%	1273.49 (917.01,1801.16)	513.59 (371.46,714.00)	-2.94 (-3.26,-2.58)
Belize	male	12.87 (9.83,16.21)	9.28 (7.03,11.85)	-27.90%	13819.42 (10312.94,17969.71)	4665.23 (3427.23,6094.02)	-3.58 (-3.79,-3.35)
Benin	male	1698.68 (1558.57,1814.81)	2267.53 (1885.69,2688.86)	33.49%	73065.05 (66596.96,78711.05)	37035.88 (30279.35,44513.90)	-2.23 (-2.30,-2.13)
Bermuda	male	0.61 (0.46,0.79)	0.29 (0.22,0.37)	-52.75%	2244.65 (1653.20,3000.65)	1012.88 (763.84,1318.08)	-3.02 (-3.25,-2.86)
Bhutan	male	93.51 (75.42,113.18)	22.81 (17.74,29.08)	-75.61%	28658.40 (22272.48,35262.55)	5840.32 (4393.74,7616.27)	-5.47 (-5.54,-5.42)

Bolivia (Plurinational State of)	male	414.03 (309.01,534.01)	460.80 (337.48,610.76)	11.30%	13823.80 (10173.42,18320.31)	8338.84 (6005.47,11238.44)	-1.57 (-1.92,-1.25)
Bosnia and Herzegovina	male	390.89 (308.77,481.51)	79.28 (61.65,101.09)	-79.72%	18736.13 (14458.57,23338.45)	5716.32 (4320.29,7434.20)	-4.79 (-5.20,-4.47)
Botswana	male	187.86 (152.02,227.05)	113.42 (84.17,147.51)	-39.62%	31389.09 (24588.56,38477.93)	10534.65 (7736.75,13924.69)	-3.71 (-3.81,-3.63)
Brazil	male	20195.77 (17187.80,23771.49)	11577.19 (9342.25,14459.05)	-42.68%	27812.45 (23377.38,33081.87)	11088.71 (8649.08,14055.46)	-3.28 (-3.42,-3.17)
Brunei Darussalam	male	3.16 (2.20,4.47)	2.16 (1.59,2.90)	-31.69%	2458.07 (1650.24,3559.37)	1043.17 (741.83,1422.16)	-3.11 (-3.19,-3.02)
Bulgaria	male	408.50 (319.66,524.06)	161.76 (127.33,205.17)	-60.40%	11013.61 (8355.29,14458.28)	5652.21 (4277.99,7284.13)	-2.26 (-2.57,-1.98)
Burkina Faso	male	3611.74 (3373.09,3801.78)	3815.58 (3144.18,4526.28)	5.64%	79343.93 (73273.53,83855.37)	34743.61 (28045.93,42184.87)	-3.12 (-3.27,-3.01)
Burundi	male	1173.42 (1001.02,1351.83)	1575.24 (1276.77,1883.33)	34.24%	42238.83 (35493.13,49754.65)	25430.43 (20152.35,31073.97)	-2.13 (-2.39,-1.97)

Cape Verde	male	72.59 (61.28,84.03)	18.20 (13.94,23.85)	-74.92%	45179.32 (37240.59,53137.88)	6638.37 (4923.92,8864.76)	-6.72 (-7.14,-6.36)
Cambodia	male	2015.39 (1697.84,2341.14)	588.33 (421.15,792.23)	-70.81%	42166.53 (34937.51,49784.05)	7935.34 (5515.41,10906.98)	-5.81 (-5.94,-5.70)
Cameroon	male	4727.61 (4620.80,4817.31)	6008.18 (5047.61,6961.18)	27.09%	92312.44 (89901.75,94293.42)	42090.70 (34841.19,49509.22)	-2.74 (-2.94,-2.53)
Canada	male	184.37 (130.57,255.24)	94.56 (68.18,131.08)	-48.71%	1638.20 (1107.02,2305.49)	684.87 (459.30,982.16)	-3.18 (-3.46,-2.94)
Central African Republic	male	860.77 (777.64,941.06)	1234.28 (1069.02,1400.69)	43.39%	64335.55 (57373.22,71002.81)	47754.69 (40535.68,55034.11)	-1.03 (-1.09,-0.98)
Chad	male	2327.11 (2182.47,2439.04)	3747.74 (3177.00,4287.86)	61.05%	80950.35 (75176.45,85135.62)	46629.53 (38674.43,54438.84)	-1.85 (-1.89,-1.77)
Chile	male	578.25 (430.80,760.62)	373.68 (282.55,480.34)	-35.38%	9808.16 (7177.90,13068.38)	4853.49 (3626.67,6395.66)	-2.28 (-2.44,-2.03)
China	male	83311.88 (65672.22,103746.92)	13850.74 (10509.29,17803.15)	-83.37%	14803.36 (11480.24,18541.05)	2264.67 (1670.28,2975.34)	-6.31 (-6.45,-6.18)
Colombia	male	1495.81 (1158.16,1868.55)	694.33 (541.45,890.16)	-53.58%	9377.37 (7001.92,12055.02)	2992.19 (2240.70,3936.87)	-4.24 (-4.38,-4.09)

Comoros	male	115.78 (100.50,133.09)	91.20 (74.69,109.77)	-21.23%	49250.34 (42040.55,57858.32)	24875.94 (20019.81,30331.15)	-2.52 (-2.71,-2.37)
Congo	male	773.66 (698.13,843.06)	967.53 (809.06,1131.48)	25.06%	65899.75 (58816.39,72361.41)	38140.16 (31561.81,45242.14)	-1.72 (-1.95,-1.42)
Cook Islands	male	1.03 (0.77,1.33)	0.32 (0.24,0.43)	-68.70%	11459.57 (8369.53,15193.39)	4564.65 (3212.39,6175.83)	-2.79 (-2.96,-2.57)
Costa Rica	male	123.67 (95.92,155.50)	65.21 (50.81,83.62)	-47.27%	8296.35 (6262.38,10721.66)	2930.24 (2236.16,3906.67)	-3.52 (-3.62,-3.43)
Cote d'Ivoire	male	2618.16 (2271.29,2972.06)	1893.76 (1441.93,2434.73)	-27.67%	43231.09 (36934.09,49626.30)	14540.48 (10639.99,19329.71)	-3.51 (-3.64,-3.34)
Croatia	male	195.90 (152.48,248.28)	88.15 (68.61,112.63)	-55.00%	9308.63 (7066.07,12071.14)	5023.71 (3765.47,6571.73)	-2.46 (-2.77,-2.23)
Cuba	male	222.25 (167.34,282.38)	121.64 (95.54,155.68)	-45.27%	4252.85 (3122.74,5567.71)	2225.00 (1705.44,2924.86)	-2.34 (-2.64,-2.08)
Cyprus	male	9.98 (7.32,13.53)	3.85 (2.95,5.12)	-61.48%	2785.40 (1958.86,3916.82)	661.30 (478.78,924.12)	-5.01 (-5.75,-4.26)
Czechia	male	324.47 (248.66,417.79)	155.54 (122.70,200.57)	-52.06%	7385.73 (5539.14,9701.68)	3481.66 (2655.46,4578.22)	-2.38 (-2.63,-2.15)
Democratic People's Republic of Korea	male	2120.64 (1671.91,2573.49)	947.31 (722.97,1220.34)	-55.33%	23015.70 (17993.56,28349.40)	8454.50 (6298.74,10993.49)	-3.65 (-3.93,-3.42)

Democratic Republic of the Congo	male	10226.52 (8891.19,11546.61)	16805.32 (13968.87,19742.37)	64.33%	54104.60 (46316.41,62192.14)	38353.83 (31390.94,45904.64)	-0.70 (-1.16,-0.08)
Denmark	male	19.39 (14.46,25.69)	10.66 (8.05,13.88)	-45.03%	844.94 (599.12,1186.91)	419.91 (297.74,572.03)	-2.33 (-2.57,-2.05)
Djibouti	male	142.00 (122.58,159.90)	128.40 (103.00,158.14)	-9.57%	53549.08 (45576.86,61173.63)	19591.48 (15204.72,24537.22)	-3.72 (-3.99,-3.54)
Dominica	male	1.91 (1.42,2.54)	0.62 (0.46,0.79)	-67.69%	5485.98 (3956.41,7433.90)	1877.05 (1371.37,2479.54)	-4.04 (-4.43,-3.68)
Dominican Republic	male	559.54 (457.27,683.04)	252.57 (194.55,322.85)	-54.86%	16043.91 (12793.90,20017.92)	4701.77 (3491.33,6142.41)	-4.85 (-5.29,-4.55)
Ecuador	male	387.20 (291.53,510.68)	256.49 (179.66,352.19)	-33.76%	8480.06 (6197.87,11500.87)	3404.03 (2342.50,4784.62)	-3.16 (-3.50,-2.84)
Egypt	male	2418.43 (1923.34,3069.78)	948.61 (717.78,1248.88)	-60.78%	8543.10 (6503.01,11119.31)	1848.66 (1366.51,2523.38)	-4.33 (-4.67,-3.86)
El Salvador	male	621.54 (502.25,753.61)	181.42 (139.74,232.71)	-70.81%	24444.15 (19286.60,30437.01)	6324.23 (4672.50,8331.55)	-5.04 (-5.39,-4.78)
Equatorial Guinea	male	154.05 (142.39,163.90)	42.54 (30.79,57.16)	-72.39%	75282.22 (68989.82,80605.11)	5605.09 (3970.49,7692.92)	-10.09 (-10.80,-9.62)

Eritrea	male	942.63 (840.74,1034.25)	927.51 (742.45,1124.00)	-1.60%	61974.47 (54259.85,68996.34)	26862.85 (20939.49,32972.13)	-2.86 (-2.94,-2.77)
Estonia	male	10.85 (8.25,14.04)	2.49 (1.92,3.18)	-77.09%	1577.60 (1147.77,2100.87)	433.59 (323.60,570.88)	-4.58 (-4.82,-4.38)
Eswatini	male	121.31 (95.64,145.84)	51.23 (36.77,68.32)	-57.77%	32637.38 (24976.15,39928.70)	9623.11 (6789.21,13123.19)	-4.11 (-4.22,-3.97)
Ethiopia	male	16309.65 (14813.06,17954.32)	15612.79 (12809.84,18762.16)	-4.27%	61910.95 (55354.65,68899.24)	27823.10 (22280.83,34568.73)	-2.82 (-3.26,-2.37)
Fiji	male	90.84 (71.12,112.65)	47.07 (35.09,62.05)	-48.18%	25165.63 (19352.81,31721.86)	11598.57 (8440.78,15590.78)	-2.35 (-2.51,-2.13)
Finland	male	25.15 (18.55,34.19)	12.05 (9.16,15.74)	-52.08%	1143.74 (805.06,1600.53)	505.34 (361.91,694.99)	-2.84 (-3.09,-2.58)
France	male	70.95 (54.95,93.44)	49.77 (38.51,64.20)	-29.84%	268.12 (198.04,370.51)	171.60 (125.95,234.24)	-1.08 (-1.27,-0.84)
Gabon	male	142.23 (116.02,169.95)	48.79 (37.34,64.21)	-65.70%	29691.17 (23627.72,36038.13)	5922.09 (4422.01,8057.97)	-5.24 (-5.36,-5.09)
Gambia	male	346.61 (318.17,372.46)	322.06 (259.65,388.86)	-7.08%	70475.17 (63565.61,76074.44)	29437.86 (23279.23,36427.20)	-3.03 (-3.10,-2.96)
Georgia	male	74.47 (57.46,96.70)	42.45 (33.02,54.35)	-42.99%	2943.97 (2199.43,3932.98)	2509.10 (1896.60,3314.90)	-0.28 (-1.04,0.55)

Germany	male	204.14 (160.34,257.24)	130.65 (102.41,166.23)	-36.00%	566.56 (426.58,731.94)	346.48 (264.73,449.13)	-1.64 (-1.73,-1.56)
Ghana	male	4698.65 (4228.75,5128.54)	3323.32 (2633.85,4100.08)	-29.27%	63968.86 (57140.57,70407.36)	22256.13 (17258.34,27730.84)	-3.61 (-3.80,-3.45)
Greece	male	88.08 (65.15,121.11)	34.31 (26.81,45.07)	-61.05%	1928.79 (1361.34,2825.19)	780.34 (579.66,1083.42)	-2.91 (-3.46,-2.35)
Greenland	male	0.19 (0.14,0.28)	0.15 (0.11,0.21)	-19.55%	664.77 (457.80,1020.83)	607.54 (410.68,894.30)	-1.41 (-2.27,-0.67)
Grenada	male	5.34 (4.02,6.92)	1.77 (1.36,2.27)	-66.90%	13298.34 (9644.73,17719.10)	3514.69 (2635.48,4609.16)	-4.06 (-4.52,-3.56)
Guam	male	4.62 (3.38,6.15)	2.59 (1.87,3.48)	-43.84%	6847.87 (5001.92,9297.37)	3492.26 (2449.55,4787.32)	-2.57 (-2.77,-2.37)
Guatemala	male	853.07 (704.54,1032.12)	522.33 (400.10,685.65)	-38.77%	21144.12 (16824.39,26093.84)	5991.74 (4435.11,8145.22)	-4.21 (-4.45,-3.97)
Guinea	male	2141.57 (1968.35,2292.13)	2021.67 (1658.40,2403.65)	-5.60%	72519.06 (65878.64,78189.22)	33671.41 (26861.77,40962.22)	-2.56 (-2.71,-2.41)
Guinea-Bissau	male	383.60 (361.19,402.85)	346.91 (289.32,410.74)	-9.56%	79800.07 (74726.95,84102.21)	38143.85 (30995.80,45927.25)	-2.50 (-2.56,-2.42)
Guyana	male	56.36 (43.17,72.60)	18.43 (14.18,23.75)	-67.31%	15226.61 (11536.19,20004.33)	5010.85 (3738.87,6594.72)	-3.69 (-3.79,-3.53)

Haiti	male	910.64 (746.05,1089.46)	871.23 (691.41,1071.67)	-4.33%	29824.35 (23840.57,36411.14)	14516.80 (11144.56,18335.76)	-2.64 (-2.73,-2.57)
Honduras	male	378.77 (302.37,473.60)	287.92 (221.85,371.54)	-23.99%	15889.21 (12210.34,20441.08)	5969.33 (4390.76,7939.38)	-3.36 (-3.61,-3.13)
Hungary	male	369.40 (284.42,465.61)	167.10 (129.28,214.12)	-54.76%	8461.39 (6345.58,11021.92)	4225.43 (3168.60,5618.37)	-2.43 (-2.53,-2.37)
Iceland	male	1.48 (1.06,2.03)	0.73 (0.54,0.96)	-50.48%	1264.90 (854.56,1791.19)	473.98 (332.86,658.90)	-3.36 (-3.61,-3.10)
India	male	166330.81 (142277.23,190990.24)	68601.37 (54100.21,85441.28)	-58.76%	37068.55 (31099.11,43379.79)	9687.52 (7489.88,12330.58)	-4.60 (-4.89,-4.22)
Indonesia	male	27459.62 (22496.33,32447.32)	6016.76 (4387.97,7954.45)	-78.09%	31606.09 (25449.66,37730.47)	5317.56 (3775.64,7225.83)	-5.84 (-5.96,-5.69)
Iran (Islamic Republic of)	male	4145.88 (3384.03,5057.04)	649.47 (504.54,845.22)	-84.33%	13937.00 (11066.17,17510.46)	1538.91 (1144.88,2048.90)	-7.37 (-8.02,-6.83)
Iraq	male	1495.09 (1178.34,1872.92)	769.14 (571.61,1019.09)	-48.56%	16576.14 (12382.97,21363.54)	3600.24 (2590.15,4907.99)	-5.31 (-5.74,-4.84)
Ireland	male	21.11 (15.43,29.42)	9.53 (7.02,12.69)	-54.85%	1295.05 (899.30,1875.43)	445.16 (311.25,616.72)	-3.24 (-3.65,-2.75)

Israel	male	244.57 (185.36,314.90)	188.31 (132.27,262.04)	-23.00%	11589.79 (8529.10,15358.89)	4852.60 (3297.03,6896.46)	-2.89 (-3.30,-2.41)
Italy	male	1007.12 (826.19,1225.33)	517.55 (409.57,657.55)	-48.61%	3280.93 (2584.76,4125.30)	1526.45 (1178.22,1975.15)	-2.36 (-2.83,-1.83)
Jamaica	male	70.40 (53.21,93.58)	28.96 (22.55,37.14)	-58.87%	6375.04 (4696.61,8741.83)	2185.87 (1661.76,2879.76)	-3.72 (-3.95,-3.53)
Japan	male	470.18 (342.54,650.86)	318.08 (245.80,412.63)	-32.35%	884.83 (620.51,1271.42)	644.18 (470.82,865.59)	-1.01 (-1.15,-0.83)
Jordan	male	331.58 (263.60,409.14)	339.59 (261.18,440.04)	2.42%	17119.19 (13376.55,21735.42)	5543.75 (4162.74,7372.44)	-3.97 (-4.37,-3.52)
Kazakhstan	male	1870.56 (1525.32,2239.42)	968.33 (767.70,1221.25)	-48.23%	23967.77 (19236.23,29246.34)	11018.80 (8458.80,13969.06)	-2.69 (-2.78,-2.60)
Kenya	male	6623.52 (5895.53,7333.18)	7372.00 (6077.61,8715.12)	11.30%	57553.93 (50385.19,64320.57)	29656.79 (23779.76,35780.04)	-2.13 (-2.28,-1.93)
Kiribati	male	13.36 (11.26,15.54)	17.77 (14.37,21.17)	33.04%	38226.47 (31927.75,45208.16)	33008.88 (26161.57,39780.60)	-0.02 (-0.20,0.22)
Kuwait	male	21.84 (16.25,29.20)	16.00 (11.95,21.37)	-26.73%	2177.98 (1589.39,2932.19)	683.85 (500.55,922.58)	-4.54 (-4.86,-4.29)
Kyrgyzstan	male	151.03 (112.40,198.80)	155.79 (116.81,200.26)	3.15%	7048.48 (5119.87,9502.00)	4886.60 (3580.19,6479.63)	-0.79 (-1.05,-0.50)

Lao People's Democratic Republic	male	847.22 (738.75,948.84)	432.31 (329.80,539.25)	-48.97%	42642.28 (36674.93,48220.29)	12560.02 (9301.49,16131.49)	-4.24 (-4.59,-3.89)
Latvia	male	16.27 (12.26,21.20)	3.69 (2.77,4.86)	-77.33%	1407.70 (1032.73,1881.13)	451.59 (331.13,604.89)	-3.99 (-4.19,-3.84)
Lebanon	male	123.25 (95.05,160.09)	41.02 (31.51,53.83)	-66.71%	7498.02 (5569.97,10083.20)	1637.47 (1203.31,2213.58)	-5.06 (-5.27,-4.84)
Lesotho	male	393.92 (337.54,446.06)	177.01 (139.13,218.22)	-55.06%	46959.77 (39671.96,53678.17)	18157.10 (14060.33,22808.16)	-3.07 (-3.17,-2.94)
Liberia	male	551.66 (489.50,610.21)	508.03 (404.29,622.65)	-7.91%	58162.55 (50628.92,65209.75)	21171.24 (16294.13,26780.74)	-3.83 (-4.10,-3.62)
Libya	male	186.65 (139.43,239.01)	65.61 (49.59,86.47)	-64.85%	8448.30 (6180.98,11022.40)	1918.51 (1418.01,2579.51)	-4.78 (-5.54,-4.08)
Lithuania	male	24.07 (18.10,31.22)	4.82 (3.69,6.08)	-80.00%	1476.68 (1080.12,1971.00)	403.64 (303.22,525.51)	-4.65 (-4.81,-4.52)
Luxembourg	male	1.28 (0.94,1.75)	1.06 (0.80,1.37)	-17.15%	756.83 (530.91,1062.78)	384.04 (275.07,520.20)	-2.31 (-2.54,-2.05)
Madagascar	male	2821.81 (2428.47,3224.16)	3540.21 (2896.86,4248.37)	25.46%	46406.85 (39047.99,53664.19)	25767.58 (20614.10,31719.26)	-2.10 (-2.35,-1.86)
Malawi	male	2686.81 (2424.36,2948.59)	2232.28 (1825.50,2752.12)	-16.92%	56664.73 (50247.61,63073.42)	24560.27 (19524.13,30889.17)	-3.15 (-3.39,-2.95)

Malaysia	male	309.98 (225.08,423.46)	63.14 (45.90,87.40)	-79.63%	3661.41 (2630.54,5095.96)	427.91 (300.30,608.64)	-7.04 (-7.67,-6.41)
Maldives	male	39.14 (32.03,46.41)	7.00 (4.95,9.41)	-82.12%	36399.07 (29229.78,43590.58)	2604.90 (1824.97,3543.49)	-9.01 (-9.51,-8.56)
Mali	male	3274.40 (3083.83,3467.62)	4151.84 (3427.24,4879.15)	26.80%	77986.37 (72785.69,82858.30)	38781.33 (31120.00,46392.77)	-2.54 (-2.64,-2.46)
Malta	male	4.59 (3.32,6.30)	1.49 (1.14,1.98)	-67.42%	2762.18 (1894.08,3949.86)	775.41 (558.32,1088.59)	-4.20 (-4.58,-3.78)
Marshall Islands	male	12.87 (11.17,14.40)	7.58 (6.22,9.09)	-41.07%	55509.12 (47082.08,62925.34)	27704.97 (22146.61,33822.15)	-2.25 (-2.31,-2.17)
Mauritania	male	445.54 (381.36,508.82)	257.59 (196.30,331.68)	-42.19%	44648.44 (37802.66,51638.59)	13417.39 (9839.69,17725.50)	-4.10 (-4.17,-4.01)
Mauritius	male	42.08 (31.37,56.58)	5.66 (4.04,7.84)	-86.56%	8459.59 (6184.74,11601.27)	1124.26 (781.86,1607.95)	-6.70 (-7.11,-6.25)
Mexico	male	9171.13 (7465.95,11091.18)	5097.48 (3935.10,6379.85)	-44.42%	23049.52 (18452.81,28200.53)	9004.35 (6868.31,11450.82)	-3.31 (-3.40,-3.22)
Micronesia (Federated States of)	male	32.07 (28.89,35.27)	21.20 (17.89,24.31)	-33.90%	60386.74 (53464.51,67237.13)	41438.89 (34766.92,48261.34)	-1.20 (-1.24,-1.15)
Monaco	male	0.06 (0.05,0.08)	0.04 (0.03,0.06)	-29.36%	463.52 (338.95,638.10)	267.02 (195.67,367.83)	-1.73 (-1.97,-1.46)

Mongolia	male	73.40 (55.18,95.84)	22.33 (16.90,29.23)	-69.58%	6820.73 (4973.84,9025.59)	1339.19 (984.29,1800.34)	-5.53 (-5.84,-5.28)
Montenegro	male	20.02 (15.05,25.42)	10.75 (8.24,13.65)	-46.29%	7113.20 (5192.97,9232.77)	4021.20 (3001.09,5255.62)	-2.73 (-3.50,-2.12)
Morocco	male	3266.39 (2690.33,3955.74)	1344.07 (1047.82,1687.57)	-58.85%	26110.86 (20745.99,32408.34)	7536.38 (5760.53,9752.08)	-4.11 (-4.32,-3.84)
Mozambique	male	4853.00 (4523.82,5139.61)	5210.68 (4337.55,6153.03)	7.37%	76868.41 (70468.91,82264.51)	36007.45 (29101.33,43652.99)	-2.85 (-3.04,-2.69)
Myanmar	male	7399.33 (6267.87,8632.77)	1220.87 (859.73,1704.23)	-83.50%	39010.46 (32565.99,46307.71)	5292.29 (3676.50,7505.60)	-7.34 (-7.65,-7.13)
Namibia	male	118.96 (92.57,148.92)	76.79 (56.44,101.03)	-35.45%	18052.04 (13654.52,22983.18)	7032.90 (5064.00,9515.03)	-3.28 (-3.48,-3.00)
Nauru	male	1.04 (0.80,1.31)	0.70 (0.53,0.91)	-32.00%	20662.14 (15532.94,26623.70)	14112.49 (10340.43,18422.22)	-0.79 (-1.45,0.13)
Nepal	male	2119.68 (1751.64,2538.93)	616.25 (470.04,793.23)	-70.93%	21567.47 (17139.28,26359.51)	4282.17 (3208.14,5672.30)	-5.77 (-5.95,-5.64)
Netherlands	male	61.66 (45.81,81.73)	35.34 (26.99,47.40)	-42.69%	918.71 (642.07,1257.41)	475.02 (342.24,662.79)	-2.01 (-2.25,-1.71)
New Zealand	male	8.68 (6.53,11.47)	8.22 (6.27,10.93)	-5.23%	564.89 (409.04,772.29)	432.39 (312.64,594.85)	-0.68 (-0.91,-0.35)

Nicaragua	male	239.60 (186.41,302.82)	57.21 (43.76,75.17)	-76.12%	12417.09 (9383.72,15967.17)	1730.65 (1269.98,2415.59)	-7.35 (-8.00,-6.85)
Niger	male	3257.45 (3082.02,3405.38)	6384.76 (5571.32,7182.16)	96.01%	82826.28 (77714.72,86821.40)	55451.41 (47393.95,63263.21)	-1.33 (-1.43,-1.20)
Nigeria	male	9846.30 (8182.31,11643.64)	4824.02 (3576.56,6368.67)	-51.01%	21655.05 (17544.90,26158.57)	4550.88 (3216.17,6207.00)	-5.69 (-6.18,-5.26)
Niue	male	0.18 (0.13,0.22)	0.05 (0.04,0.06)	-72.48%	16646.52 (12309.03,21494.11)	6934.50 (4994.16,9448.67)	-3.04 (-3.14,-2.93)
North Macedonia	male	275.23 (224.60,325.06)	134.95 (108.43,164.72)	-50.97%	28315.35 (22697.27,33883.95)	13001.20 (10205.98,16143.14)	-3.12 (-3.39,-2.93)
Northern Mariana Islands	male	1.36 (1.00,1.82)	0.85 (0.62,1.15)	-37.30%	6127.39 (4461.44,8327.65)	4780.34 (3365.88,6587.17)	-0.14 (-0.38,0.22)
Norway	male	17.82 (13.25,23.69)	8.98 (6.78,12.08)	-49.59%	941.54 (662.06,1322.41)	378.58 (266.27,526.04)	-3.16 (-3.42,-2.87)
Oman	male	166.80 (129.73,212.38)	36.56 (26.76,50.18)	-78.08%	14705.52 (11127.00,19113.28)	1245.37 (902.49,1717.46)	-8.05 (-8.77,-7.30)

Pakistan	male	14739.68 (12449.33,17381.53)	3675.43 (2764.16,4971.08)	-75.06%	24988.54 (20630.79,30097.66)	3158.79 (2275.43,4398.17)	-7.10 (-7.60,-6.65)
Palau	male	0.98 (0.73,1.26)	0.50 (0.37,0.65)	-49.10%	13418.24 (9811.31,17645.12)	6077.40 (4449.00,7986.39)	-2.28 (-2.44,-2.06)
Palestine	male	585.56 (509.03,653.71)	159.81 (121.79,209.32)	-72.71%	56223.71 (48253.14,63366.80)	6379.02 (4707.49,8596.73)	-7.30 (-7.80,-6.73)
Panama	male	84.04 (65.89,106.35)	58.68 (45.03,75.01)	-30.17%	7035.43 (5331.11,9237.44)	2852.78 (2116.45,3773.04)	-3.15 (-3.47,-2.85)
Papua New Guinea	male	387.88 (300.05,477.82)	634.78 (482.31,821.59)	63.66%	18540.09 (14089.02,23251.41)	12943.64 (9630.96,17218.26)	-0.63 (-0.98,-0.14)
Paraguay	male	333.34 (259.38,424.19)	228.40 (174.80,293.83)	-31.48%	16948.83 (12875.44,22128.00)	6837.72 (5116.17,9067.65)	-2.87 (-2.99,-2.72)
Peru	male	1722.15 (1394.74,2113.49)	1060.71 (822.99,1337.58)	-38.41%	16554.08 (13166.11,20574.81)	6469.03 (4872.56,8317.05)	-3.52 (-3.76,-3.32)
Philippines	male	6507.89 (5284.95,7993.76)	5405.89 (4104.65,6923.22)	-16.93%	21274.22 (16910.44,26809.45)	10529.36 (7850.56,13585.64)	-1.75 (-2.10,-1.23)

Poland	male	1700.83 (1310.39,2176.04)	584.10 (438.94,748.07)	-65.66%	10216.06 (7677.74,13388.93)	3626.48 (2655.17,4752.07)	-3.63 (-3.70,-3.58)
Portugal	male	159.21 (120.15,210.58)	47.99 (36.99,61.24)	-69.86%	3616.95 (2606.39,4996.77)	1079.41 (794.91,1455.32)	-3.71 (-4.19,-3.16)
Puerto Rico	male	43.69 (32.58,57.45)	15.15 (11.67,19.58)	-65.31%	2670.98 (1915.52,3606.86)	992.37 (746.66,1314.04)	-3.66 (-3.79,-3.55)
Qatar	male	10.82 (8.00,14.49)	14.17 (10.22,19.36)	30.98%	3652.48 (2634.25,4934.13)	667.46 (470.98,927.51)	-5.48 (-5.77,-5.18)
Republic of Korea	male	425.81 (305.46,582.38)	121.75 (91.23,159.90)	-71.41%	2098.55 (1457.84,2941.91)	539.09 (395.81,739.83)	-4.57 (-4.71,-4.40)
Republic of Moldova	male	65.97 (49.98,84.37)	21.93 (16.68,28.31)	-66.76%	3275.81 (2433.06,4286.91)	1330.20 (990.82,1759.61)	-3.28 (-3.67,-2.92)
Romania	male	1142.70 (869.84,1433.78)	355.04 (276.79,454.16)	-68.93%	11183.91 (8220.37,14353.92)	4429.60 (3324.64,5849.41)	-3.42 (-3.64,-3.27)
Russian Federation	male	284.23 (217.24,364.30)	114.03 (88.36,147.66)	-59.88%	401.90 (299.51,535.19)	167.84 (128.60,218.78)	-2.83 (-2.94,-2.73)
Rwanda	male	1415.04 (1192.02,1646.76)	1045.78 (825.97,1278.51)	-26.10%	39823.63 (32917.60,47302.51)	16536.43 (12695.92,20826.41)	-3.28 (-3.76,-2.86)

Saint Kitts and Nevis	male	1.07 (0.78,1.40)	0.48 (0.37,0.63)	-54.71%	5566.60 (3978.01,7434.96)	1700.79 (1258.75,2282.06)	-3.94 (-4.21,-3.70)
Saint Lucia	male	4.76 (3.55,6.16)	2.26 (1.73,2.90)	-52.46%	7382.25 (5352.20,9784.22)	2758.68 (2068.04,3575.28)	-3.10 (-3.38,-2.81)
Saint Vincent and the Grenadines	male	6.91 (5.30,8.81)	2.09 (1.61,2.69)	-69.80%	13050.98 (9758.45,17013.95)	3815.72 (2893.80,5071.85)	-4.23 (-4.53,-4.00)
Samoa	male	24.10 (19.47,29.23)	22.36 (17.25,27.99)	-7.24%	30021.42 (23895.64,36996.95)	22880.32 (17422.88,28901.49)	-0.65 (-0.82,-0.45)
San Marino	male	0.08 (0.06,0.11)	0.06 (0.05,0.08)	-29.51%	798.63 (554.51,1150.13)	424.07 (308.44,576.72)	-2.08 (-2.52,-1.62)
Sao Tome and Principe	male	40.98 (37.25,44.21)	18.68 (14.68,23.37)	-54.42%	69854.55 (62632.24,75835.67)	18688.44 (14327.85,23931.98)	-4.69 (-4.79,-4.61)
Saudi Arabia	male	351.81 (250.43,508.59)	29.42 (21.87,39.04)	-91.64%	4023.34 (2745.31,5993.53)	148.00 (106.88,202.42)	-9.63 (-10.42,-8.53)
Senegal	male	3161.46 (3037.33,3261.64)	1552.69 (1202.96,1983.66)	-50.89%	86672.96 (83014.26,89461.48)	20931.33 (15777.27,27228.68)	-4.65 (-5.20,-4.06)

Serbia	male	1414.41 (1193.47,1627.27)	711.82 (592.40,851.45)	-49.67%	32375.33 (26883.25,37882.95)	17233.21 (13995.37,20782.45)	-2.42 (-2.74,-2.16)
Seychelles	male	2.54 (1.87,3.40)	0.52 (0.37,0.71)	-79.72%	7787.71 (5570.61,10566.73)	1154.40 (807.86,1630.36)	-6.10 (-6.76,-5.38)
Sierra Leone	male	1265.76 (1171.48,1349.54)	1262.50 (1022.99,1515.14)	-0.26%	72679.77 (66493.77,77770.44)	31458.19 (24809.55,38310.90)	-2.87 (-2.97,-2.75)
Singapore	male	24.13 (17.87,32.65)	13.96 (10.66,18.50)	-42.14%	1727.60 (1237.03,2416.49)	562.90 (414.90,762.44)	-4.03 (-4.15,-3.94)
Slovakia	male	235.02 (176.73,299.07)	90.76 (68.37,116.63)	-61.38%	10178.22 (7388.34,13183.07)	3923.84 (2917.08,5219.95)	-3.27 (-3.37,-3.18)
Slovenia	male	49.78 (37.35,64.03)	25.49 (19.95,32.73)	-48.79%	5852.70 (4262.49,7694.56)	2926.12 (2243.55,3875.34)	-2.46 (-2.60,-2.38)
Solomon Islands	male	82.77 (71.45,94.52)	104.20 (84.05,124.63)	25.89%	49032.94 (41576.49,56363.48)	33421.14 (26527.57,40515.86)	-1.02 (-1.18,-0.79)
Somalia	male	2887.57 (2681.85,3063.36)	7595.61 (6950.32,8164.79)	163.05%	77680.01 (71182.25,83125.73)	72502.08 (65646.71,78683.16)	-0.29 (-0.33,-0.27)
South Africa	male	2527.92 (2037.51,3101.32)	983.10 (689.70,1350.64)	-61.11%	15736.27 (12358.12,19481.72)	4060.16 (2756.36,5743.95)	-4.91 (-5.07,-4.80)

South Sudan	male	1427.11 (1227.91,1641.77)	1130.78 (909.40,1377.99)	-20.76%	45271.00 (38257.93,52830.36)	23506.34 (18488.39,29472.26)	-2.45 (-2.68,-2.26)
Spain	male	292.30 (220.17,380.97)	139.15 (110.15,177.93)	-52.39%	1655.60 (1216.96,2266.20)	680.75 (518.98,901.89)	-2.73 (-3.03,-2.41)
Sri Lanka	male	1448.25 (1177.48,1786.82)	234.89 (167.14,325.76)	-83.78%	18624.82 (14789.33,23155.02)	2708.99 (1872.80,3867.46)	-6.75 (-6.97,-6.55)
Sudan	male	4529.68 (3914.76,5198.07)	2162.05 (1656.72,2839.83)	-52.27%	44110.29 (37387.62,51490.46)	10498.33 (7816.79,14107.95)	-4.72 (-5.00,-4.36)
Suriname	male	20.69 (15.87,26.58)	11.78 (9.01,15.20)	-43.05%	11082.48 (8281.50,14453.41)	4345.95 (3219.99,5719.73)	-3.52 (-3.74,-3.39)
Sweden	male	45.91 (34.28,62.34)	22.23 (16.90,29.08)	-51.59%	1208.20 (848.12,1706.33)	493.33 (357.62,680.71)	-2.96 (-3.39,-2.50)
Switzerland	male	15.72 (11.64,20.79)	11.38 (8.69,14.92)	-27.64%	512.75 (358.99,718.50)	295.01 (212.25,408.42)	-1.95 (-2.13,-1.77)
Syrian Arab Republic	male	1072.91 (805.69,1384.42)	289.69 (221.80,372.55)	-73.00%	16166.09 (11762.87,21422.73)	4179.59 (3063.25,5507.23)	-4.71 (-5.01,-4.46)
Taiwan (Province of China)	male	364.09 (263.70,482.00)	56.69 (41.31,76.48)	-84.43%	3887.98 (2705.52,5249.28)	612.79 (431.54,856.09)	-6.23 (-6.64,-5.86)

Tajikistan	male	419.69 (332.09,521.43)	421.86 (329.83,533.36)	0.52%	15601.32 (11953.56,19832.13)	8765.72 (6656.93,11423.93)	-2.10 (-2.80,-1.50)
Thailand	male	4835.05 (3911.36,5895.21)	513.72 (395.96,669.10)	-89.38%	18233.30 (14637.65,22298.71)	1605.85 (1192.80,2163.35)	-8.16 (-8.64,-7.70)
Timor-Leste	male	152.96 (129.12,176.86)	52.82 (37.40,72.34)	-65.47%	39341.50 (32483.54,46144.93)	8432.05 (5797.48,11831.47)	-6.18 (-6.71,-5.79)
Togo	male	1206.01 (1089.65,1311.91)	1011.51 (810.49,1223.09)	-16.13%	68855.72 (61409.40,75378.73)	26750.77 (21003.64,32936.55)	-3.03 (-3.13,-2.90)
Tokelau	male	0.24 (0.19,0.29)	0.08 (0.06,0.10)	-66.62%	31175.93 (24304.25,38413.90)	12609.94 (9165.77,16830.48)	-3.05 (-3.22,-2.87)
Tonga	male	13.03 (10.40,16.05)	7.23 (5.42,9.24)	-44.55%	28713.82 (22362.82,35937.69)	15920.23 (11629.81,20744.08)	-1.64 (-1.84,-1.38)
Trinidad and Tobago	male	27.85 (20.86,36.48)	11.92 (9.28,15.17)	-57.20%	4850.73 (3523.71,6573.24)	1820.72 (1374.62,2374.25)	-3.95 (-4.28,-3.72)
Tunisia	male	398.46 (303.34,519.82)	107.00 (84.06,134.20)	-73.15%	9346.75 (6846.38,12622.93)	1870.64 (1421.08,2401.02)	-5.46 (-5.79,-5.19)

Turkey	male	3941.49 (3278.91,4695.14)	1010.26 (773.62,1264.66)	-74.37%	13096.29 (10642.13,15757.12)	2492.74 (1877.30,3215.07)	-5.81 (-5.99,-5.68)
Turkmenistan	male	123.76 (94.98,160.89)	62.23 (47.46,80.47)	-49.72%	6813.30 (5028.05,9072.62)	2442.17 (1827.41,3268.90)	-3.86 (-4.22,-3.39)
Tuvalu	male	1.56 (1.31,1.85)	1.03 (0.81,1.29)	-33.89%	37420.90 (30902.54,44648.16)	18955.95 (14516.68,24022.58)	-1.92 (-2.06,-1.72)
Uganda	male	3245.40 (2744.84,3750.41)	2576.29 (2034.75,3275.49)	-20.62%	36376.92 (29950.97,43106.90)	12353.74 (9426.54,16028.11)	-3.94 (-4.17,-3.75)
Ukraine	male	403.14 (307.27,525.55)	169.46 (131.53,220.54)	-57.96%	1764.59 (1310.77,2378.22)	894.32 (662.79,1188.30)	-2.53 (-2.67,-2.42)
United Arab Emirates	male	33.16 (24.63,44.87)	38.20 (26.32,54.92)	15.19%	2745.88 (2010.88,3754.65)	574.86 (391.39,834.52)	-4.89 (-5.53,-4.28)
United Kingdom	male	347.14 (259.17,462.16)	172.29 (132.34,228.50)	-50.37%	1385.09 (979.85,1917.03)	589.87 (425.52,820.57)	-2.78 (-3.12,-2.41)
United Republic of Tanzania	male	4520.39 (3848.11,5198.32)	4152.98 (3305.87,5081.37)	-8.13%	34681.15 (28862.92,40840.61)	14557.77 (11325.89,18246.94)	-2.81 (-3.00,-2.58)
United States Virgin Islands	male	1.47 (1.11,1.89)	0.39 (0.30,0.49)	-73.62%	3025.79 (2216.25,3983.29)	855.69 (649.44,1113.37)	-4.62 (-5.00,-4.31)

United States of America	male	344.88 (252.65,457.14)	284.43 (203.39,405.21)	-17.53%	323.12 (224.74,451.47)	209.00 (140.88,309.13)	-2.16 (-2.92,-1.48)
Uruguay	male	133.55 (99.83,172.10)	84.76 (64.39,110.86)	-36.53%	10195.06 (7413.29,13429.24)	6030.44 (4439.78,8106.89)	-1.67 (-1.89,-1.34)
Uzbekistan	male	1014.51 (740.87,1329.13)	514.03 (389.11,675.86)	-49.33%	9855.44 (7005.20,13101.73)	3098.93 (2268.92,4197.71)	-3.96 (-4.14,-3.72)
Vanuatu	male	37.58 (32.25,42.39)	49.54 (41.01,58.21)	31.84%	50650.54 (42672.95,57812.83)	36154.24 (29558.51,42946.08)	-1.00 (-1.11,-0.86)
Venezuela (Bolivarian Republic of)	male	966.94 (777.79,1203.69)	840.67 (656.45,1046.31)	-13.06%	10427.71 (8120.91,13228.75)	6227.29 (4704.21,7944.21)	-1.49 (-1.85,-1.05)
Viet Nam	male	2842.34 (2093.32,3686.19)	646.39 (414.86,972.03)	-77.26%	9398.50 (6758.70,12445.62)	1617.41 (1015.79,2486.96)	-5.22 (-5.51,-4.81)
Yemen	male	3703.10 (3239.20,4187.41)	2928.09 (2298.83,3648.79)	-20.93%	52553.72 (45016.97,60370.66)	18416.56 (13954.13,23369.90)	-4.39 (-4.77,-4.12)
Zambia	male	1972.68 (1709.98,2233.76)	1610.47 (1266.71,1988.39)	-18.36%	49533.82 (42148.71,57088.01)	17302.40 (13325.97,21809.25)	-3.83 (-4.35,-3.31)

Zimbabwe	male	1641.61 (1352.99,1943.33)	1387.87 (1085.22,1731.10)	-15.46%	33361.10 (26968.04,40343.10)	19851.16 (15050.53,24998.73)	-1.05 (-1.36,-0.61)
Afghanistan	female	1979.42 (1742.23,2245.38)	3986.94 (3353.35,4647.51)	101.42%	34238.28 (29444.45,39519.21)	21419.02 (17728.64,25443.35)	-1.61 (-2.11,-1.06)
Albania	female	468.23 (405.65,535.99)	203.28 (174.73,231.92)	-56.59%	29227.64 (24936.26,33987.21)	15056.41 (12781.84,17480.31)	-2.77 (-3.02,-2.58)
Algeria	female	1021.34 (831.35,1250.92)	456.38 (375.12,561.61)	-55.32%	8237.17 (6553.35,10318.29)	2268.47 (1823.11,2885.23)	-4.21 (-4.44,-3.98)
American Samoa	female	2.07 (1.67,2.57)	1.24 (0.99,1.56)	-39.98%	9350.25 (7366.43,11830.20)	5138.50 (4007.83,6630.93)	-2.03 (-2.21,-1.80)
Andorra	female	0.16 (0.12,0.19)	0.14 (0.11,0.16)	-11.37%	678.03 (525.75,844.82)	392.71 (312.58,489.58)	-1.45 (-1.68,-1.15)
Angola	female	1752.73 (1532.12,1987.93)	1717.26 (1367.92,2089.59)	-2.02%	34483.92 (29559.09,39645.56)	11230.89 (8741.01,13990.01)	-3.78 (-4.16,-3.28)
Antigua and Barbuda	female	1.33 (1.10,1.61)	0.96 (0.79,1.15)	-28.15%	4462.76 (3559.97,5475.43)	2183.95 (1770.04,2654.08)	-2.49 (-2.60,-2.42)
Argentina	female	1584.12 (1342.02,1855.11)	1440.20 (1208.38,1709.03)	-9.09%	9912.02 (8207.93,11796.64)	6513.48 (5331.88,7870.35)	-1.15 (-1.31,-0.93)

Armenia	female	18.61 (15.40,22.21)	9.15 (7.64,10.89)	-50.82%	1041.73 (835.98,1280.50)	580.80 (471.45,701.67)	-2.14 (-2.44,-1.86)
Australia	female	17.99 (14.74,21.48)	15.27 (12.67,18.48)	-15.14%	172.05 (140.01,206.70)	102.23 (83.79,123.96)	-0.99 (-1.36,-0.47)
Austria	female	40.64 (33.82,49.60)	22.13 (18.37,26.36)	-45.55%	1148.01 (928.01,1447.23)	569.44 (453.64,711.88)	-2.21 (-2.53,-1.84)
Azerbaijan	female	133.83 (109.46,162.12)	118.41 (99.31,141.58)	-11.52%	3570.76 (2868.80,4398.04)	2325.37 (1912.59,2857.37)	-1.74 (-2.24,-1.25)
Bahamas	female	3.62 (2.94,4.47)	3.09 (2.57,3.70)	-14.57%	2851.29 (2290.01,3608.98)	1632.38 (1336.34,1988.24)	-2.41 (-2.66,-2.20)
Bahrain	female	9.80 (7.90,12.20)	8.24 (6.78,9.91)	-15.85%	4604.47 (3639.45,5807.90)	1537.24 (1239.10,1880.13)	-3.50 (-3.67,-3.33)
Bangladesh	female	9097.84 (7746.73,10666.07)	4156.95 (3476.92,4949.81)	-54.31%	17083.25 (14253.94,20370.78)	5244.37 (4301.93,6351.77)	-3.65 (-3.91,-3.27)
Barbados	female	4.47 (3.66,5.29)	3.28 (2.75,3.85)	-26.55%	3627.30 (2913.39,4401.11)	2228.71 (1836.20,2718.08)	-1.66 (-1.74,-1.60)
Belarus	female	203.10 (171.57,238.79)	85.11 (70.55,101.66)	-58.10%	3754.85 (3113.06,4502.18)	1689.91 (1390.09,2045.57)	-2.88 (-3.16,-2.65)
Belgium	female	51.40 (42.61,62.83)	27.68 (22.64,34.04)	-46.14%	1150.01 (911.60,1448.13)	558.85 (437.79,719.83)	-2.09 (-2.40,-1.69)
Belize	female	10.26 (8.47,12.51)	9.58 (7.91,11.38)	-6.69%	11191.72 (9025.10,13818.49)	4681.49 (3808.72,5640.18)	-2.92 (-3.07,-2.77)
Benin	female	795.09 (676.08,915.99)	1103.78 (894.31,1323.78)	38.82%	32922.59 (27554.09,38452.28)	17753.38 (13909.94,21828.73)	-1.86 (-2.06,-1.59)
Bermuda	female	0.79 (0.65,0.95)	0.44 (0.37,0.52)	-44.29%	2728.83 (2208.60,3370.26)	1403.83 (1160.11,1666.66)	-2.53 (-2.70,-2.42)

Bhutan	female	60.12 (50.47,70.99)	20.85 (17.51,25.13)	-65.33%	20745.30 (16942.31,24642.04)	5878.48 (4837.36,7233.30)	-4.43 (-4.53,-4.35)
Bolivia (Plurinational State of)	female	467.59 (388.41,555.86)	499.85 (414.48,602.14)	6.90%	14792.89 (11999.50,17890.72)	8613.30 (6997.51,10543.03)	-1.63 (-1.79,-1.42)
Bosnia and Herzegovina	female	596.16 (517.59,677.80)	202.17 (174.43,235.43)	-66.09%	26524.33 (22610.03,30474.29)	12077.57 (10370.39,14171.14)	-3.20 (-3.52,-2.98)
Botswana	female	149.18 (124.80,175.00)	96.74 (78.72,117.73)	-35.15%	22864.86 (18992.42,27325.38)	8741.84 (6985.27,10924.31)	-3.19 (-3.28,-3.08)
Brazil	female	16211.21 (14207.62,18422.38)	9934.90 (8448.17,11624.95)	-38.72%	21935.84 (18931.38,25192.49)	9187.47 (7735.77,10941.80)	-3.00 (-3.07,-2.95)
Brunei Darussalam	female	3.21 (2.52,4.02)	2.28 (1.84,2.84)	-29.01%	2757.28 (2149.04,3532.78)	1189.56 (939.97,1509.30)	-2.79 (-2.89,-2.68)
Bulgaria	female	755.93 (652.91,870.93)	387.38 (327.79,452.40)	-48.75%	17535.14 (14742.75,20377.53)	10981.85 (9200.63,12872.37)	-1.48 (-1.67,-1.28)
Burkina Faso	female	1930.40 (1687.60,2186.66)	2339.59 (1954.35,2805.41)	21.20%	40103.39 (34517.80,45943.41)	20743.03 (17008.74,25399.01)	-2.38 (-2.54,-2.24)

Burundi	female	848.14 (727.80,978.05)	1150.12 (968.48,1349.11)	35.60%	29299.65 (24641.73,34274.33)	18726.91 (15487.74,22433.79)	-1.83 (-2.10,-1.64)
Cape Verde	female	27.49 (22.46,33.22)	9.98 (8.09,12.19)	-63.71%	15676.50 (12546.01,19162.39)	3804.49 (2972.27,4768.62)	-4.88 (-5.01,-4.77)
Cambodia	female	1646.06 (1422.85,1875.49)	575.64 (459.21,708.85)	-65.03%	31313.01 (26604.02,36190.92)	7575.27 (5923.26,9486.05)	-4.89 (-5.02,-4.78)
Cameroon	female	2396.38 (2177.24,2628.81)	1929.21 (1560.12,2358.70)	-19.49%	47299.34 (42634.16,52173.21)	13878.29 (10983.90,17341.77)	-4.03 (-4.14,-3.87)
Canada	female	256.84 (208.78,318.46)	167.95 (136.30,206.18)	-34.61%	2207.01 (1755.39,2763.37)	1128.09 (880.25,1416.02)	-2.26 (-2.53,-1.98)
Central African Republic	female	383.98 (329.83,438.76)	525.63 (439.33,617.34)	36.89%	27871.59 (23632.03,32457.16)	19728.46 (16196.17,23540.99)	-1.16 (-1.35,-0.98)
Chad	female	1243.15 (1082.06,1397.97)	1802.34 (1486.03,2179.12)	44.98%	41203.81 (35385.70,46981.65)	22163.08 (17982.82,27437.12)	-2.06 (-2.24,-1.82)
Chile	female	419.09 (336.63,519.48)	268.89 (222.73,324.27)	-35.84%	6823.19 (5377.22,8585.68)	3363.37 (2708.14,4162.61)	-2.22 (-2.34,-2.02)
China	female	39048.89 (32446.38,46258.38)	11412.86 (9450.87,13788.70)	-70.77%	7414.74 (6087.77,8856.71)	1951.84 (1602.45,2378.77)	-4.79 (-5.23,-4.39)

Colombia	female	1254.37 (1025.59,1498.41)	696.93 (580.97,826.06)	-44.44%	7678.91 (6147.36,9364.27)	2860.05 (2361.67,3485.66)	-3.70 (-3.85,-3.57)
Comoros	female	72.09 (61.90,82.75)	50.51 (42.62,59.79)	-29.94%	30485.67 (25630.49,35463.90)	14124.29 (11704.42,17204.82)	-2.75 (-2.91,-2.62)
Congo	female	391.58 (337.81,449.36)	409.00 (336.87,489.99)	4.45%	32449.52 (27536.09,37731.05)	16138.71 (12913.54,19735.98)	-2.13 (-2.51,-1.67)
Cook Islands	female	0.65 (0.52,0.81)	0.24 (0.20,0.30)	-62.67%	7781.62 (6099.90,9855.07)	3184.42 (2471.07,4036.31)	-2.91 (-3.00,-2.80)
Costa Rica	female	101.14 (84.22,122.50)	66.48 (55.89,78.83)	-34.27%	6818.88 (5508.11,8452.04)	2800.77 (2299.95,3426.36)	-3.02 (-3.10,-2.95)
Cote d'Ivoire	female	1947.72 (1687.14,2196.41)	1714.41 (1402.05,2078.86)	-11.98%	33314.50 (28639.36,37871.46)	13920.56 (11056.79,17264.84)	-2.79 (-2.95,-2.59)
Croatia	female	364.54 (309.92,424.15)	212.12 (179.58,248.86)	-41.81%	14795.25 (12424.63,17366.94)	9808.17 (8219.37,11569.50)	-1.59 (-1.79,-1.43)
Cuba	female	206.88 (171.70,249.06)	130.47 (107.85,157.06)	-36.94%	3942.91 (3208.07,4823.27)	2305.74 (1878.58,2812.60)	-1.83 (-2.06,-1.64)
Cyprus	female	7.30 (5.85,9.20)	3.69 (3.01,4.59)	-49.46%	2097.39 (1605.53,2695.78)	630.01 (501.61,816.38)	-4.00 (-4.62,-3.34)
Czechia	female	665.07 (557.21,776.51)	405.23 (342.13,470.54)	-39.07%	12846.21 (10602.09,15275.34)	7579.56 (6323.55,8894.67)	-1.59 (-1.79,-1.39)

Democratic People's Republic of Korea	female	1252.43 (1025.85,1498.47)	630.63 (521.17,766.92)	-49.65%	12344.59 (9938.12,14916.65)	5691.10 (4615.14,7008.81)	-2.95 (-3.32,-2.64)
Democratic Republic of the Congo	female	5836.16 (4993.64,6704.33)	9355.21 (7970.97,11033.40)	60.30%	30441.11 (25509.96,35548.40)	21849.12 (18137.63,26349.85)	-0.56 (-1.14,0.24)
Denmark	female	18.98 (15.48,22.88)	11.49 (9.31,14.09)	-39.47%	826.73 (652.99,1033.10)	459.76 (353.28,587.20)	-1.71 (-1.97,-1.38)
Djibouti	female	72.93 (62.99,83.62)	73.64 (61.36,87.52)	0.98%	32300.90 (27481.73,37661.21)	13146.49 (10612.70,15921.57)	-3.19 (-3.40,-3.04)
Dominica	female	1.92 (1.59,2.34)	0.78 (0.64,0.93)	-59.73%	5500.81 (4395.00,6817.88)	2394.83 (1934.67,2928.02)	-3.13 (-3.41,-2.88)
Dominican Republic	female	472.16 (398.92,549.72)	264.75 (218.51,311.90)	-43.93%	12983.00 (10778.20,15466.45)	4971.33 (4023.64,5996.24)	-3.83 (-4.21,-3.55)
Ecuador	female	367.60 (297.88,442.05)	326.68 (264.46,404.68)	-11.13%	7875.94 (6307.36,9603.99)	4138.73 (3296.99,5198.90)	-2.31 (-2.70,-1.99)
Egypt	female	1844.40 (1546.58,2168.73)	960.88 (794.77,1159.64)	-47.90%	6760.24 (5531.25,8102.05)	2006.92 (1624.69,2495.28)	-3.29 (-3.61,-2.83)

El Salvador	female	449.44 (381.61,523.74)	173.75 (142.95,207.95)	-61.34%	17018.61 (14145.34,20118.55)	5375.01 (4316.56,6548.74)	-4.16 (-4.48,-3.92)
Equatorial Guinea	female	96.18 (86.26,106.78)	21.09 (16.85,26.38)	-78.07%	43697.47 (38483.21,49216.59)	3318.96 (2607.48,4228.16)	-9.88 (-10.50,-9.46)
Eritrea	female	626.47 (553.79,702.26)	577.53 (483.21,676.74)	-7.81%	41468.48 (35989.22,47047.02)	17269.64 (14082.83,20478.90)	-2.93 (-3.01,-2.84)
Estonia	female	18.23 (15.07,21.60)	6.79 (5.66,8.09)	-62.76%	2250.36 (1832.86,2717.59)	991.17 (808.67,1188.59)	-2.86 (-2.94,-2.79)
Eswatini	female	86.67 (70.89,103.81)	43.23 (34.91,52.68)	-50.12%	21087.95 (16993.68,25529.15)	7770.53 (6159.33,9632.06)	-3.28 (-3.37,-3.16)
Ethiopia	female	12578.44 (11348.80,13824.75)	11200.49 (9354.90,13170.17)	-10.95%	48784.58 (43551.96,54344.96)	20767.11 (17007.97,24915.28)	-3.00 (-3.39,-2.53)
Fiji	female	36.19 (29.54,43.79)	15.77 (12.82,19.42)	-56.42%	10187.08 (8120.00,12611.84)	3759.68 (2966.49,4761.20)	-3.37 (-3.66,-3.11)
Finland	female	24.32 (20.10,30.06)	13.05 (10.70,15.79)	-46.34%	1072.52 (846.09,1368.40)	550.34 (428.48,700.47)	-2.05 (-2.31,-1.73)
France	female	68.42 (56.48,83.33)	51.82 (42.73,63.12)	-24.25%	251.04 (201.59,318.27)	173.41 (138.33,220.79)	-0.66 (-0.90,-0.35)

Gabon	female	65.16 (53.34,78.79)	29.40 (23.60,35.79)	-54.88%	13517.27 (10718.87,16683.99)	3383.70 (2666.33,4208.67)	-4.44 (-4.58,-4.25)
Gambia	female	176.04 (153.28,199.89)	177.92 (146.20,214.56)	1.07%	36157.86 (31216.23,41572.22)	16201.82 (13102.36,19764.83)	-2.72 (-2.78,-2.66)
Georgia	female	68.97 (57.33,82.74)	40.22 (33.75,47.87)	-41.68%	2406.71 (1954.91,2948.36)	2161.96 (1762.10,2632.84)	-0.07 (-0.64,0.55)
Germany	female	168.90 (141.40,201.19)	119.25 (100.22,140.77)	-29.40%	451.95 (367.33,551.82)	322.96 (263.92,389.21)	-1.05 (-1.15,-0.95)
Ghana	female	2214.72 (1901.66,2548.88)	1500.80 (1214.66,1852.69)	-32.24%	30409.89 (25475.52,35379.76)	9831.97 (7845.64,12412.68)	-3.85 (-4.19,-3.53)
Greece	female	76.17 (62.25,92.96)	33.75 (27.85,40.55)	-55.69%	1653.42 (1301.05,2102.34)	745.79 (591.74,926.46)	-2.35 (-2.82,-1.82)
Greenland	female	0.53 (0.42,0.65)	0.33 (0.27,0.40)	-37.68%	2187.22 (1720.35,2767.78)	1383.27 (1100.41,1733.26)	-2.01 (-2.26,-1.80)
Grenada	female	4.44 (3.64,5.26)	1.82 (1.52,2.17)	-59.06%	10707.11 (8575.99,12888.80)	3722.03 (3070.03,4545.07)	-3.19 (-3.55,-2.78)
Guam	female	3.08 (2.47,3.87)	1.90 (1.53,2.33)	-38.31%	5206.68 (4077.33,6661.82)	2703.14 (2112.76,3377.50)	-2.61 (-2.87,-2.39)
Guatemala	female	692.49 (588.74,806.26)	529.42 (443.76,634.91)	-23.55%	16951.16 (14072.36,20047.83)	5772.17 (4711.09,7076.71)	-3.66 (-3.85,-3.48)

Guinea	female	971.72 (831.99,1126.21)	1014.62 (834.17,1221.06)	4.41%	31808.65 (26764.34,37419.46)	16070.97 (12912.18,19668.09)	-2.20 (-2.28,-2.10)
Guinea-Bissau	female	183.53 (160.32,207.04)	163.89 (135.47,196.55)	-10.70%	36282.56 (31205.07,41407.85)	17406.21 (14058.37,21335.92)	-2.31 (-2.40,-2.18)
Guyana	female	43.43 (35.62,52.49)	18.67 (15.51,22.27)	-57.02%	11376.11 (9142.16,13995.66)	4897.36 (3986.33,5941.05)	-2.77 (-2.83,-2.67)
Haiti	female	753.03 (647.61,871.18)	797.42 (658.55,942.93)	5.89%	23315.42 (19643.92,27670.25)	12558.67 (10229.20,15078.79)	-2.14 (-2.19,-2.10)
Honduras	female	308.25 (259.19,365.52)	297.40 (249.12,353.28)	-3.52%	12809.89 (10561.17,15497.27)	5836.57 (4783.66,7051.42)	-2.72 (-2.90,-2.58)
Hungary	female	775.41 (661.78,892.83)	451.92 (385.45,523.27)	-41.72%	14694.42 (12333.73,17182.61)	9046.02 (7544.78,10491.49)	-1.62 (-1.68,-1.56)
Iceland	female	1.34 (1.08,1.65)	0.78 (0.64,0.97)	-41.26%	1170.03 (904.02,1501.52)	529.14 (412.48,677.29)	-2.45 (-2.70,-2.14)
India	female	84667.43 (72836.03,97310.30)	42234.24 (34907.21,50754.61)	-50.12%	20750.92 (17599.61,24219.17)	6468.78 (5238.27,7913.84)	-4.00 (-4.31,-3.57)

Indonesia	female	21399.09 (18878.78,24044.73)	7324.69 (5972.34,8903.73)	-65.77%	23824.12 (20599.52,27239.99)	6237.33 (5026.88,7687.60)	-4.25 (-4.38,-4.07)
Iran (Islamic Republic of)	female	2234.28 (1834.09,2662.97)	456.08 (373.43,549.94)	-79.59%	7967.15 (6439.84,9741.42)	1143.27 (913.43,1410.71)	-6.53 (-7.11,-6.05)
Iraq	female	1003.56 (819.85,1211.52)	568.49 (459.70,707.01)	-43.35%	11628.08 (9231.18,14401.37)	2796.46 (2229.49,3484.98)	-4.93 (-5.18,-4.65)
Ireland	female	21.01 (16.47,26.36)	11.10 (8.94,13.52)	-47.18%	1300.05 (984.13,1690.07)	514.06 (399.86,646.75)	-2.63 (-2.99,-2.12)
Israel	female	168.50 (135.59,207.62)	153.16 (118.29,196.38)	-9.11%	7938.09 (6237.94,10005.85)	3967.59 (2973.12,5199.77)	-2.15 (-2.50,-1.72)
Italy	female	830.33 (711.67,963.23)	454.24 (384.79,538.16)	-45.29%	2570.62 (2141.50,3073.00)	1304.09 (1094.23,1570.65)	-2.02 (-2.44,-1.55)
Jamaica	female	74.90 (61.73,91.19)	38.67 (31.98,46.29)	-48.37%	6481.81 (5216.80,8076.50)	2822.85 (2288.13,3433.42)	-2.85 (-3.01,-2.71)
Japan	female	644.60 (535.79,784.60)	414.17 (346.76,489.89)	-35.75%	1158.05 (934.68,1452.02)	781.88 (631.92,958.02)	-1.03 (-1.15,-0.87)
Jordan	female	206.14 (169.68,247.91)	235.64 (191.53,290.96)	14.31%	11782.77 (9580.45,14387.08)	4476.82 (3517.50,5626.35)	-3.32 (-3.66,-2.96)
Kazakhstan	female	661.71 (553.77,784.64)	383.22 (311.46,467.73)	-42.09%	8148.65 (6644.76,9901.88)	4272.87 (3397.65,5371.57)	-2.06 (-2.13,-1.96)
Kenya	female	6250.76 (5676.82,6844.96)	7464.61 (6420.33,8564.46)	19.42%	53329.32 (47814.45,59180.84)	29768.57 (25405.63,34572.59)	-1.92 (-2.11,-1.70)

Kiribati	female	8.56 (7.21,10.04)	10.88 (9.11,12.94)	27.04%	24260.88 (20102.37,28836.13)	19564.93 (15992.26,23561.10)	-0.33 (-0.50,-0.13)
Kuwait	female	17.12 (13.70,21.19)	16.54 (13.36,20.32)	-3.43%	2288.92 (1799.11,2852.97)	815.25 (647.68,1008.11)	-4.01 (-4.27,-3.81)
Kyrgyzstan	female	111.87 (92.28,136.45)	129.77 (108.23,156.93)	16.00%	4929.93 (3948.74,6103.82)	3958.18 (3206.88,4891.63)	-0.28 (-0.54,0.03)
Lao People's Democratic Republic	female	642.92 (568.17,720.56)	360.22 (300.26,430.44)	-43.97%	31265.79 (27334.11,35390.06)	10615.69 (8712.36,13027.06)	-3.72 (-4.00,-3.40)
Latvia	female	28.91 (24.32,34.11)	10.70 (8.99,12.71)	-63.00%	2102.62 (1738.90,2530.92)	1050.38 (860.98,1263.16)	-2.34 (-2.48,-2.23)
Lebanon	female	105.99 (86.83,127.39)	44.30 (36.61,54.00)	-58.21%	6590.60 (5243.79,8078.97)	1727.94 (1380.28,2145.96)	-4.47 (-4.62,-4.31)
Lesotho	female	271.43 (234.58,308.99)	126.75 (105.07,153.07)	-53.30%	30915.99 (26311.27,35746.01)	12646.03 (10339.35,15555.73)	-2.87 (-2.99,-2.69)
Liberia	female	130.09 (105.66,158.75)	132.20 (104.57,168.28)	1.62%	13365.02 (10655.54,16698.36)	5679.74 (4363.05,7356.90)	-3.44 (-4.22,-2.77)
Libya	female	139.08 (111.56,170.90)	55.44 (45.69,66.58)	-60.14%	6885.11 (5357.38,8706.70)	1733.63 (1397.13,2120.58)	-4.40 (-5.11,-3.72)
Lithuania	female	41.04 (34.16,49.23)	14.09 (11.83,16.69)	-65.67%	2176.45 (1771.97,2656.49)	947.43 (779.68,1150.35)	-2.97 (-3.09,-2.88)
Luxembourg	female	1.28 (1.06,1.56)	1.16 (0.96,1.42)	-9.12%	745.27 (595.04,941.75)	433.28 (346.56,550.51)	-1.60 (-1.82,-1.32)

Madagascar	female	1823.14 (1561.21,2088.13)	2404.82 (2014.05,2833.36)	31.91%	30188.85 (25323.66,34994.67)	17943.65 (14840.90,21450.70)	-1.75 (-2.02,-1.46)
Malawi	female	2417.46 (2174.31,2641.55)	2633.95 (2264.75,3028.92)	8.96%	48764.12 (43351.91,53927.74)	27349.17 (23070.85,32155.75)	-2.12 (-2.43,-1.80)
Malaysia	female	312.79 (250.50,386.71)	93.13 (74.32,117.56)	-70.23%	3752.00 (2959.17,4677.27)	674.96 (529.51,859.11)	-5.54 (-6.12,-4.89)
Maldives	female	28.09 (23.33,32.98)	6.22 (4.97,7.74)	-77.85%	26707.78 (21861.93,31860.81)	3468.80 (2721.88,4408.01)	-6.93 (-7.47,-6.42)
Mali	female	1706.37 (1495.26,1917.64)	2399.10 (1989.90,2845.27)	40.60%	39935.06 (34331.08,45223.78)	22468.31 (18265.44,27082.32)	-2.03 (-2.11,-1.95)
Malta	female	3.47 (2.78,4.25)	1.40 (1.17,1.70)	-59.64%	2075.71 (1614.41,2610.24)	743.13 (596.12,927.65)	-3.19 (-3.53,-2.79)
Marshall Islands	female	8.63 (7.49,9.79)	4.51 (3.76,5.30)	-47.76%	39397.32 (33562.79,45111.44)	17382.68 (14268.66,20711.42)	-2.75 (-2.78,-2.70)
Mauritania	female	207.80 (174.79,243.25)	146.45 (115.99,179.81)	-29.52%	20922.80 (17403.26,24875.97)	7516.88 (5819.23,9445.47)	-3.21 (-3.32,-3.06)
Mauritius	female	37.64 (30.89,45.84)	7.84 (6.27,9.59)	-79.18%	7437.72 (6006.34,9211.54)	1499.08 (1180.86,1870.98)	-5.36 (-5.65,-5.04)

Mexico	female	5432.92 (4594.97,6284.93)	3196.43 (2668.24,3793.01)	-41.17%	13272.48 (11158.98,15531.29)	5405.78 (4381.89,6533.86)	-3.11 (-3.19,-3.02)
Micronesia (Federated States of)	female	21.08 (18.83,23.44)	13.50 (11.65,15.33)	-35.97%	42417.72 (37228.93,47564.32)	27763.61 (23648.08,31866.98)	-1.35 (-1.46,-1.25)
Monaco	female	0.07 (0.06,0.09)	0.05 (0.05,0.07)	-25.79%	531.53 (428.91,654.46)	335.61 (268.88,417.70)	-1.24 (-1.50,-0.92)
Mongolia	female	38.96 (31.28,47.98)	19.57 (15.86,24.07)	-49.78%	3603.64 (2837.75,4522.91)	1145.42 (904.07,1436.30)	-3.82 (-3.97,-3.69)
Montenegro	female	37.37 (31.97,43.79)	27.08 (23.12,31.39)	-27.55%	12056.37 (10048.21,14337.95)	8694.54 (7320.32,10241.99)	-1.59 (-2.15,-1.15)
Morocco	female	1807.89 (1541.30,2124.10)	796.52 (657.91,949.70)	-55.94%	14612.95 (12212.24,17604.36)	4673.60 (3790.60,5726.46)	-3.72 (-3.91,-3.47)
Mozambique	female	3434.30 (3090.66,3771.18)	3387.89 (2886.93,3946.19)	-1.35%	50047.08 (44304.10,55459.08)	22189.86 (18615.05,26243.78)	-2.97 (-3.15,-2.82)
Myanmar	female	5506.91 (4722.35,6384.02)	1316.11 (1029.13,1611.23)	-76.10%	28160.13 (23824.34,33040.88)	5325.32 (4097.83,6628.11)	-6.02 (-6.22,-5.88)
Namibia	female	90.79 (74.60,110.23)	71.79 (57.89,88.15)	-20.93%	13137.13 (10587.67,16209.96)	6125.33 (4851.13,7671.21)	-2.70 (-2.96,-2.36)

Nauru	female	0.65 (0.52,0.80)	0.41 (0.33,0.51)	-36.52%	13640.64 (10699.23,17061.71)	8492.26 (6640.48,10624.00)	-1.24 (-1.76,-0.51)
Nepal	female	2190.72 (1912.96,2490.12)	1087.11 (913.92,1310.14)	-50.38%	22562.64 (19470.67,26275.86)	6989.18 (5738.14,8545.29)	-4.18 (-4.39,-3.99)
Netherlands	female	59.36 (47.62,73.47)	36.13 (29.44,43.58)	-39.14%	885.14 (680.60,1124.53)	487.39 (381.77,615.76)	-1.61 (-1.88,-1.24)
New Zealand	female	14.67 (11.93,17.86)	13.33 (11.10,15.96)	-9.08%	919.83 (727.73,1146.15)	647.20 (523.04,796.42)	-0.78 (-0.97,-0.53)
Nicaragua	female	176.39 (147.00,209.63)	61.95 (51.92,73.85)	-64.88%	8746.42 (7147.92,10598.22)	1769.76 (1452.00,2156.15)	-6.05 (-6.56,-5.66)
Niger	female	1795.42 (1606.69,2006.39)	3669.30 (3146.17,4220.40)	104.37%	45012.24 (39697.17,50973.95)	31675.96 (26792.42,36871.30)	-1.03 (-1.10,-0.93)
Nigeria	female	6003.44 (5067.95,6993.72)	6416.73 (5202.80,7773.62)	6.88%	13426.16 (11084.18,15872.65)	5665.11 (4538.17,7000.38)	-2.94 (-3.78,-2.01)
Niue	female	0.11 (0.09,0.13)	0.03 (0.02,0.04)	-71.18%	10445.57 (8245.12,12754.14)	4433.32 (3467.05,5737.14)	-3.00 (-3.10,-2.93)
North Macedonia	female	221.43 (191.89,254.44)	130.68 (112.53,150.99)	-40.98%	22674.23 (19325.28,26510.25)	12574.98 (10710.41,14670.13)	-2.31 (-2.51,-2.16)

Northern Mariana Islands	female	1.00 (0.79,1.23)	0.57 (0.47,0.70)	-42.85%	4881.62 (3792.24,6137.46)	3337.50 (2678.66,4107.14)	-0.76 (-0.96,-0.48)
Norway	female	16.55 (13.46,20.45)	9.66 (8.00,11.96)	-41.63%	874.79 (683.17,1131.51)	421.38 (335.10,538.04)	-2.24 (-2.54,-1.86)
Oman	female	92.66 (74.99,112.99)	22.68 (18.18,27.48)	-75.53%	11404.63 (9006.81,14278.99)	1399.58 (1091.09,1726.58)	-6.75 (-7.39,-6.08)
Pakistan	female	8821.59 (7568.20,10160.53)	4235.30 (3381.97,5208.54)	-51.99%	16585.48 (14100.58,19304.45)	3903.39 (3056.92,4919.74)	-4.99 (-5.19,-4.83)
Palau	female	0.60 (0.48,0.74)	0.28 (0.23,0.34)	-53.71%	8624.28 (6847.42,10721.50)	4034.10 (3228.27,5101.81)	-2.38 (-2.47,-2.28)
Palestine	female	291.18 (250.31,336.22)	106.90 (86.68,129.81)	-63.29%	28438.38 (24003.02,33391.89)	4450.37 (3507.93,5513.28)	-6.01 (-6.34,-5.56)
Panama	female	67.46 (56.71,81.21)	55.87 (46.19,66.76)	-17.17%	5799.97 (4761.22,7120.39)	2769.89 (2237.42,3400.01)	-2.55 (-2.76,-2.34)
Papua New Guinea	female	288.70 (237.21,349.02)	482.07 (394.14,589.31)	66.98%	14855.14 (12006.24,18136.72)	10440.79 (8439.50,12971.82)	-0.68 (-0.97,-0.29)
Paraguay	female	270.59 (222.34,329.53)	192.60 (158.99,230.44)	-28.82%	14054.27 (11263.64,17420.00)	5903.53 (4757.69,7199.89)	-2.71 (-2.81,-2.56)

Peru	female	1368.38 (1186.31,1582.49)	935.39 (784.80,1100.12)	-31.64%	12715.59 (10865.85,14834.80)	5698.42 (4682.39,6790.43)	-2.80 (-2.94,-2.62)
Philippines	female	4962.23 (4247.23,5780.20)	4004.72 (3240.81,4964.17)	-19.30%	16505.46 (13965.34,19480.96)	8034.05 (6409.01,10053.78)	-1.90 (-2.19,-1.47)
Poland	female	3206.34 (2744.32,3730.65)	1614.44 (1361.41,1899.88)	-49.65%	16648.23 (13963.69,19685.98)	8242.65 (6898.18,9815.54)	-2.39 (-2.42,-2.34)
Portugal	female	121.88 (99.39,148.50)	45.65 (38.47,53.60)	-62.54%	2641.18 (2079.26,3338.98)	954.71 (771.53,1165.31)	-2.93 (-3.31,-2.45)
Puerto Rico	female	53.71 (43.94,64.88)	24.47 (20.59,28.88)	-54.44%	3046.45 (2454.54,3752.46)	1405.60 (1172.03,1705.79)	-2.85 (-2.95,-2.78)
Qatar	female	5.51 (4.41,6.83)	6.42 (5.21,7.76)	16.36%	3736.83 (2928.44,4717.87)	891.31 (711.33,1089.78)	-4.55 (-4.83,-4.24)
Republic of Korea	female	479.44 (383.43,581.35)	159.90 (132.34,192.43)	-66.65%	2365.51 (1857.30,2931.32)	707.86 (574.99,867.26)	-3.82 (-4.08,-3.54)
Republic of Moldova	female	89.36 (74.47,107.00)	45.44 (38.01,54.33)	-49.15%	3894.61 (3209.26,4725.20)	2373.61 (1950.20,2875.77)	-1.77 (-2.17,-1.37)
Romania	female	2059.71 (1772.12,2384.48)	962.86 (824.16,1126.39)	-53.25%	17662.21 (14937.46,20827.11)	9851.69 (8278.41,11690.90)	-2.07 (-2.22,-1.96)

Russian Federation	female	393.30 (329.55,470.98)	190.57 (157.98,227.49)	-51.55%	457.87 (378.34,559.39)	229.62 (187.91,275.02)	-2.21 (-2.31,-2.13)
Rwanda	female	1042.83 (893.15,1201.54)	826.47 (682.91,987.31)	-20.75%	28193.08 (23687.17,32862.85)	12717.53 (10347.67,15571.62)	-2.92 (-3.39,-2.48)
Saint Kitts and Nevis	female	1.04 (0.84,1.29)	0.61 (0.51,0.74)	-41.13%	5226.87 (4116.76,6609.59)	2131.17 (1720.64,2617.41)	-2.97 (-3.14,-2.82)
Saint Lucia	female	5.01 (4.12,6.10)	2.76 (2.35,3.25)	-44.95%	7364.88 (5906.38,9189.94)	3257.58 (2704.93,3900.70)	-2.59 (-2.82,-2.36)
Saint Vincent and the Grenadines	female	5.58 (4.58,6.74)	2.23 (1.87,2.66)	-60.05%	10446.39 (8353.61,12817.11)	4157.24 (3415.38,5048.67)	-3.09 (-3.27,-2.92)
Samoa	female	12.03 (9.75,14.49)	11.73 (9.37,14.24)	-2.53%	16631.76 (13248.12,20246.75)	12705.16 (9956.62,15636.93)	-0.68 (-0.85,-0.48)
San Marino	female	0.09 (0.07,0.10)	0.07 (0.06,0.08)	-19.60%	813.63 (642.04,1025.36)	462.29 (371.45,580.04)	-1.62 (-1.99,-1.19)
Sao Tome and Principe	female	15.45 (13.02,18.08)	7.59 (6.09,9.30)	-50.85%	26286.22 (21790.11,31114.31)	7786.97 (6065.85,9743.05)	-4.29 (-4.52,-4.08)
Saudi Arabia	female	279.01 (224.52,353.18)	37.48 (30.50,45.03)	-86.57%	3833.27 (3005.26,4923.69)	247.82 (199.58,305.14)	-8.08 (-8.97,-6.92)

Senegal	female	991.98 (843.87,1155.70)	574.85 (467.39,706.76)	-42.05%	26235.55 (21831.98,31278.03)	7814.31 (6214.31,9800.03)	-3.72 (-4.00,-3.34)
Serbia	female	1265.59 (1120.60,1429.51)	621.91 (536.22,714.43)	-50.86%	26890.87 (23579.24,30891.47)	14466.56 (12456.98,16712.09)	-2.31 (-2.53,-2.14)
Seychelles	female	2.24 (1.81,2.73)	0.65 (0.53,0.82)	-70.84%	6784.09 (5361.34,8439.67)	1615.08 (1259.57,2052.11)	-4.60 (-5.13,-4.02)
Sierra Leone	female	556.43 (472.94,633.67)	622.04 (508.06,747.75)	11.79%	30819.34 (25653.47,35576.97)	15365.35 (12359.31,18766.08)	-2.26 (-2.48,-1.99)
Singapore	female	29.09 (23.53,35.94)	18.88 (15.61,23.01)	-35.09%	2087.54 (1645.19,2633.04)	774.52 (625.10,958.99)	-3.28 (-3.42,-3.14)
Slovakia	female	415.16 (355.60,484.75)	226.85 (193.23,267.80)	-45.36%	15627.73 (13159.26,18581.86)	8246.95 (6925.42,9811.73)	-2.10 (-2.21,-2.00)
Slovenia	female	106.83 (90.39,125.42)	71.17 (60.95,82.38)	-33.38%	10781.49 (8937.80,12824.95)	6899.84 (5827.24,8067.28)	-1.52 (-1.58,-1.48)
Solomon Islands	female	56.25 (48.37,64.58)	67.37 (55.84,80.19)	19.77%	35501.04 (29972.74,41420.73)	22321.01 (18216.54,26928.80)	-1.34 (-1.51,-1.13)
Somalia	female	2024.64 (1867.91,2190.56)	5427.05 (4888.45,5937.63)	168.05%	58377.26 (53054.12,63870.71)	54280.58 (48351.29,59958.17)	-0.30 (-0.35,-0.26)

South Africa	female	1969.39 (1659.81,2335.39)	1030.53 (834.89,1248.55)	-47.67%	11369.44 (9427.18,13750.98)	4073.15 (3225.71,5041.71)	-3.61 (-3.70,-3.53)
South Sudan	female	845.23 (722.48,979.28)	786.25 (651.07,929.16)	-6.98%	30398.47 (25448.70,35628.05)	16804.96 (13562.68,20165.97)	-2.11 (-2.26,-1.97)
Spain	female	350.28 (288.44,426.63)	134.95 (112.84,159.63)	-61.47%	2000.65 (1590.94,2475.61)	654.86 (532.18,788.88)	-3.05 (-3.62,-2.38)
Sri Lanka	female	1283.56 (1086.07,1493.10)	420.74 (344.47,514.43)	-67.22%	15599.87 (12993.93,18434.92)	4260.10 (3449.63,5277.18)	-4.84 (-5.17,-4.56)
Sudan	female	2880.51 (2454.48,3334.33)	1519.55 (1225.79,1878.68)	-47.25%	28553.81 (23881.47,33475.39)	7602.51 (5975.21,9559.54)	-4.27 (-4.53,-3.94)
Suriname	female	16.65 (13.74,20.06)	12.11 (10.04,14.35)	-27.26%	8967.59 (7225.64,11001.30)	4288.52 (3463.18,5218.47)	-2.75 (-2.91,-2.65)
Sweden	female	40.49 (33.25,49.81)	22.80 (18.34,27.88)	-43.69%	1065.24 (836.17,1361.94)	522.98 (405.19,673.17)	-2.05 (-2.46,-1.58)
Switzerland	female	17.13 (13.85,20.73)	13.33 (10.94,16.11)	-22.16%	558.81 (436.55,701.27)	351.53 (277.10,440.49)	-1.36 (-1.58,-1.09)
Syrian Arab Republic	female	757.61 (622.00,913.59)	240.13 (196.06,291.69)	-68.30%	11981.09 (9585.13,14780.81)	3291.41 (2642.94,4077.23)	-4.44 (-4.72,-4.19)
Taiwan (Province of China)	female	232.14 (185.10,289.24)	65.33 (53.38,79.23)	-71.86%	2579.03 (2013.03,3252.92)	681.87 (542.13,850.44)	-4.66 (-4.84,-4.53)

Tajikistan	female	289.54 (239.00,345.94)	344.66 (282.47,417.98)	19.04%	10662.22 (8539.75,13111.08)	7381.76 (5927.59,9096.08)	-1.14 (-1.65,-0.59)
Thailand	female	3193.77 (2726.43,3755.41)	526.83 (441.33,625.12)	-83.50%	12140.47 (10247.93,14306.02)	1583.70 (1303.62,1940.07)	-6.51 (-7.09,-5.84)
Timor-Leste	female	120.44 (105.26,135.97)	47.54 (37.50,59.37)	-60.53%	32476.62 (27829.82,37095.00)	7725.81 (5974.73,9789.98)	-5.78 (-6.27,-5.43)
Togo	female	462.35 (386.54,547.59)	431.86 (354.05,528.66)	-6.59%	25317.33 (20634.53,30493.23)	11190.98 (8959.08,13903.24)	-2.40 (-2.58,-2.13)
Tokelau	female	0.15 (0.12,0.18)	0.05 (0.04,0.06)	-68.48%	18736.95 (15196.91,22717.57)	7622.45 (5909.92,9542.33)	-3.07 (-3.21,-2.93)
Tonga	female	7.57 (6.27,9.09)	4.17 (3.32,5.17)	-44.92%	16972.18 (13805.49,20708.77)	9102.29 (7090.26,11600.56)	-1.85 (-2.01,-1.64)
Trinidad and Tobago	female	26.97 (21.95,32.96)	14.33 (11.98,16.98)	-46.88%	4652.26 (3706.81,5837.10)	2149.25 (1762.18,2598.02)	-3.02 (-3.25,-2.86)
Tunisia	female	293.28 (239.39,354.11)	95.04 (78.82,113.26)	-67.59%	7028.04 (5622.05,8623.78)	1638.91 (1324.59,1992.36)	-4.93 (-5.29,-4.61)

Turkey	female	4359.63 (3824.21,4905.86)	1564.61 (1303.08,1845.63)	-64.11%	14717.23 (12770.78,16631.28)	3905.69 (3170.89,4721.57)	-4.73 (-4.95,-4.55)
Turkmenistan	female	86.40 (70.42,107.25)	51.58 (42.60,61.79)	-40.30%	4591.85 (3653.25,5794.79)	2092.93 (1680.25,2567.67)	-2.90 (-3.20,-2.51)
Tuvalu	female	0.94 (0.80,1.11)	0.53 (0.43,0.64)	-44.05%	21186.17 (17718.90,25081.55)	10575.84 (8438.20,13128.36)	-2.03 (-2.16,-1.87)
Uganda	female	2413.08 (2115.69,2744.67)	2327.21 (1932.37,2731.53)	-3.56%	27169.77 (23256.24,31290.84)	11228.04 (9132.42,13418.53)	-3.19 (-3.40,-2.99)
Ukraine	female	680.30 (573.06,809.07)	420.50 (351.18,500.22)	-38.19%	2490.01 (2047.37,3015.55)	1792.39 (1455.94,2168.58)	-1.20 (-1.43,-1.03)
United Arab Emirates	female	27.18 (22.35,32.97)	28.31 (22.68,34.80)	4.14%	4084.58 (3326.34,5009.57)	1114.76 (888.50,1377.43)	-4.22 (-4.66,-3.83)
United Kingdom	female	314.02 (256.07,386.55)	177.75 (147.77,215.40)	-43.40%	1209.63 (953.86,1537.04)	607.96 (481.95,778.47)	-1.98 (-2.31,-1.58)
United Republic of Tanzania	female	3339.46 (2875.67,3807.14)	3913.05 (3263.17,4621.57)	17.18%	24613.29 (20886.98,28427.96)	13332.89 (10944.62,15989.94)	-1.82 (-2.05,-1.53)
United States Virgin Islands	female	1.78 (1.47,2.15)	0.66 (0.55,0.78)	-62.83%	3388.54 (2752.06,4165.46)	1271.28 (1049.32,1534.81)	-3.59 (-3.85,-3.38)

United States of America	female	1215.93 (1010.16,1444.27)	971.05 (791.30,1184.64)	-20.14%	1057.98 (865.31,1287.58)	671.76 (531.23,849.18)	-1.66 (-1.80,-1.51)
Uruguay	female	96.50 (78.54,116.73)	64.01 (52.94,77.24)	-33.67%	6951.36 (5547.25,8658.58)	4237.90 (3426.37,5237.67)	-1.44 (-1.63,-1.19)
Uzbekistan	female	673.63 (540.80,837.04)	478.62 (390.04,578.25)	-28.95%	6354.01 (5010.99,8074.32)	2857.36 (2280.95,3545.72)	-2.61 (-2.78,-2.39)
Vanuatu	female	21.87 (18.63,25.47)	26.68 (22.11,31.68)	21.99%	31047.90 (26005.86,36485.94)	19812.57 (16181.80,23851.10)	-1.46 (-1.56,-1.35)
Venezuela (Bolivarian Republic of)	female	670.70 (564.91,792.28)	659.59 (555.11,785.02)	-1.66%	7133.20 (5914.10,8618.73)	4710.60 (3872.81,5730.61)	-1.08 (-1.42,-0.69)
Viet Nam	female	2829.19 (2299.12,3417.07)	673.60 (501.17,920.14)	-76.19%	9209.96 (7360.76,11401.65)	1733.30 (1257.17,2406.44)	-5.34 (-5.52,-5.07)
Yemen	female	2461.67 (2161.68,2763.86)	1799.49 (1457.43,2194.34)	-26.90%	36291.01 (31098.33,41353.06)	11609.73 (9212.93,14352.35)	-4.61 (-5.01,-4.35)
Zambia	female	1140.73 (991.31,1305.70)	933.16 (768.76,1147.14)	-18.20%	28165.86 (24072.91,32600.45)	10138.64 (8206.35,12753.91)	-3.66 (-4.15,-3.13)

Zimbabwe	female	1318.17 (1118.19,1529.95)	1494.68 (1263.20,1766.20)	13.39%	25261.07 (21084.77,29929.72)	19498.86 (16046.67,23316.23)	-0.14 (-0.47,0.33)

Abbreviations: CI, confidence interval; EAPC, estimated annual percentage change; UI, uncertainty interval; VAD, vitamin A deficiency.

Supplemental Table S4: The DALY and age-standardized DALY rate of VAD in 1990 and 2019, and its temporal trends from 1990 to 2019.

**Nation**	**Sex**	**DALY No. (thousands) (95% UI)**		**Change in absolute number (%)**	**Age-standardized DALY rate per 100,000 No. (95% UI)**		**1990-2019 EAPC No. (95% CI)**
		**1990**	**2019**		**1990**	**2019**	
Afghanistan	both	7878.40 (5034.01,11556.67)	17488.54 (10887.01,25832.76)	121.98%	43.77 (27.84,64.15)	28.63 (17.96,42.33)	-1.40 (-1.80,-0.93)
Albania	both	922.47 (552.25,1445.09)	129.70 (73.24,216.02)	-85.94%	23.30 (13.90,36.38)	7.86 (4.44,13.21)	-4.38 (-4.80,-4.08)
Algeria	both	6062.64 (3639.33,9157.97)	3077.76 (1916.51,4711.79)	-49.23%	17.29 (10.46,25.90)	7.34 (4.57,11.21)	-2.83 (-3.01,-2.68)
American Samoa	both	6.14 (3.42,10.47)	2.63 (1.38,4.60)	-57.12%	8.68 (4.82,14.77)	4.83 (2.51,8.46)	-1.65 (-1.84,-1.39)
Andorra	both	0.11 (0.04,0.23)	0.05 (0.02,0.11)	-58.92%	0.38 (0.14,0.77)	0.14 (0.05,0.33)	-3.08 (-3.58,-2.53)

Angola	both	9044.14 (5986.55,12946.78)	12919.30 (7987.07,19468.99)	42.85%	56.73 (37.02,81.28)	29.21 (18.48,42.74)	-2.08 (-2.35,-1.71)
Antigua and Barbuda	both	5.33 (3.32,8.03)	3.83 (2.40,5.65)	-28.12%	8.24 (5.14,12.37)	5.43 (3.40,8.14)	-1.51 (-1.66,-1.41)
Argentina	both	4675.42 (2723.71,7220.61)	1982.48 (1102.37,3390.37)	-57.60%	13.39 (7.81,20.67)	5.53 (3.08,9.35)	-3.07 (-3.24,-2.89)
Armenia	both	23.65 (12.64,40.37)	6.80 (3.33,12.01)	-71.23%	0.64 (0.34,1.10)	0.32 (0.16,0.57)	-2.19 (-2.61,-1.67)
Australia	both	2.64 (1.23,5.26)	1.39 (0.61,2.80)	-47.24%	0.02 (0.01,0.04)	0.01 (0.00,0.02)	-2.15 (-2.44,-1.73)
Austria	both	35.19 (14.66,69.35)	9.50 (3.38,21.83)	-72.99%	0.76 (0.32,1.48)	0.21 (0.08,0.49)	-3.99 (-4.47,-3.42)
Azerbaijan	both	353.66 (193.40,591.54)	143.95 (76.50,239.44)	-59.30%	4.06 (2.23,6.74)	1.79 (0.96,2.95)	-3.12 (-3.95,-2.25)
Bahamas	both	17.11 (10.47,25.72)	16.21 (10.13,24.40)	-5.26%	6.21 (3.83,9.29)	5.05 (3.21,7.61)	-0.82 (-0.97,-0.70)
Bahrain	both	28.06 (16.06,46.86)	9.43 (4.69,16.65)	-66.38%	4.74 (2.67,8.00)	1.20 (0.60,2.12)	-4.48 (-4.62,-4.36)
Bangladesh	both	60545.62 (38165.81,89138.65)	14216.01 (8788.53,21750.16)	-76.52%	35.37 (22.58,52.06)	9.45 (5.79,14.62)	-4.29 (-4.46,-4.05)
Barbados	both	11.91 (7.45,18.52)	8.04 (5.11,12.33)	-32.43%	5.18 (3.24,8.11)	3.89 (2.42,5.90)	-0.95 (-1.09,-0.83)
Belarus	both	99.81 (47.32,192.64)	16.02 (6.61,31.51)	-83.95%	1.20 (0.58,2.25)	0.28 (0.12,0.55)	-4.95 (-5.22,-4.61)
Belgium	both	46.97 (20.09,95.37)	12.35 (4.37,27.28)	-73.71%	0.76 (0.32,1.57)	0.19 (0.07,0.41)	-4.45 (-4.87,-3.98)

Belize	both	47.66 (28.44,73.34)	34.76 (21.52,53.00)	-27.07%	17.47 (10.56,26.71)	8.28 (5.11,12.63)	-2.38 (-2.61,-2.19)
Benin	both	7887.95 (5226.64,11461.76)	10911.29 (6974.31,16305.72)	38.33%	90.51 (59.77,131.66)	54.27 (34.87,80.59)	-1.70 (-1.97,-1.37)
Bermuda	both	0.75 (0.40,1.33)	0.18 (0.08,0.34)	-76.45%	1.77 (0.94,3.16)	0.59 (0.26,1.15)	-3.99 (-4.18,-3.84)
Bhutan	both	560.09 (338.71,826.76)	111.22 (67.00,164.85)	-80.14%	62.13 (37.62,91.96)	16.40 (9.89,24.36)	-4.74 (-4.91,-4.62)
Bolivia (Plurinational State of)	both	1652.44 (1040.36,2437.00)	1719.11 (1075.25,2592.86)	4.03%	18.02 (11.34,26.45)	12.49 (7.85,18.67)	-0.95 (-1.14,-0.72)
Bosnia and Herzegovina	both	600.53 (338.10,983.96)	73.48 (39.51,127.04)	-87.76%	15.89 (8.97,25.99)	4.59 (2.50,7.92)	-5.15 (-5.68,-4.72)
Botswana	both	856.29 (556.54,1269.61)	477.56 (303.85,718.22)	-44.23%	45.22 (29.57,66.12)	19.65 (12.57,29.67)	-2.75 (-2.85,-2.64)
Brazil	both	41320.59 (25904.64,61162.27)	18072.25 (11056.57,26646.48)	-56.26%	23.19 (14.64,34.28)	10.22 (6.20,15.16)	-2.78 (-2.86,-2.72)
Brunei Darussalam	both	8.14 (4.43,13.70)	2.72 (1.32,4.79)	-66.59%	2.49 (1.37,4.11)	0.83 (0.40,1.45)	-3.66 (-3.92,-3.38)
Bulgaria	both	516.46 (271.67,871.29)	148.87 (78.44,258.78)	-71.18%	8.89 (4.72,14.92)	4.43 (2.31,7.64)	-2.27 (-2.51,-2.04)
Burkina Faso	both	20310.24 (13427.50,29362.31)	24172.62 (15361.56,34729.55)	19.02%	121.06 (80.23,175.95)	66.62 (42.11,96.08)	-2.27 (-2.44,-2.16)

Burundi	both	4538.76 (2862.08,6788.88)	5736.73 (3583.75,8777.45)	26.39%	47.98 (30.34,71.30)	30.27 (19.01,45.93)	-2.03 (-2.30,-1.86)
Cape Verde	both	264.79 (166.31,395.16)	66.22 (40.80,102.08)	-74.99%	48.90 (31.04,72.78)	11.91 (7.38,18.41)	-4.87 (-5.14,-4.65)
Cambodia	both	11591.49 (7462.16,17288.25)	3391.05 (2005.21,5377.46)	-70.75%	66.63 (42.70,99.37)	19.26 (11.42,30.49)	-4.17 (-4.21,-4.11)
Cameroon	both	15507.68 (10335.32,22329.96)	16302.82 (10154.84,25201.88)	5.13%	91.11 (60.89,131.48)	39.37 (24.53,60.73)	-2.83 (-2.89,-2.73)
Canada	both	120.68 (56.91,231.06)	38.55 (14.58,78.54)	-68.06%	0.61 (0.29,1.16)	0.19 (0.07,0.38)	-3.93 (-4.46,-3.42)
Central African Republic	both	4272.52 (2779.58,6233.19)	5630.61 (3633.50,8243.13)	31.79%	95.57 (62.37,139.70)	72.16 (46.90,105.76)	-1.06 (-1.21,-0.94)
Chad	both	11353.54 (7442.49,16213.56)	19433.42 (12871.56,28343.86)	71.17%	108.52 (70.77,154.64)	67.03 (44.68,97.16)	-1.64 (-1.76,-1.49)
Chile	both	309.13 (134.80,607.51)	52.86 (18.56,126.82)	-82.90%	2.19 (0.96,4.30)	0.43 (0.16,1.00)	-5.25 (-5.52,-4.89)
China	both	123307.32 (78817.68,179189.43)	40319.25 (25940.47,61110.38)	-67.30%	10.68 (6.85,15.59)	3.69 (2.34,5.55)	-4.10 (-4.54,-3.75)

Colombia	both	3605.43 (2211.60,5595.07)	1882.43 (1152.51,2829.01)	-47.79%	9.22 (5.75,14.26)	4.31 (2.64,6.49)	-2.77 (-3.03,-2.54)
Comoros	both	437.61 (269.09,655.87)	211.82 (130.40,324.30)	-51.60%	59.31 (37.27,88.93)	26.73 (16.46,40.84)	-2.91 (-3.10,-2.76)
Congo	both	3140.49 (2051.52,4612.65)	3673.53 (2332.08,5458.18)	16.97%	84.88 (55.74,123.73)	55.06 (34.92,81.28)	-1.39 (-1.72,-1.01)
Cook Islands	both	2.87 (1.69,4.55)	0.96 (0.57,1.45)	-66.58%	12.78 (7.55,20.07)	6.19 (3.64,9.62)	-2.15 (-2.27,-2.00)
Costa Rica	both	269.69 (160.45,413.21)	170.53 (105.90,260.59)	-36.77%	7.24 (4.36,10.97)	4.08 (2.51,6.25)	-2.00 (-2.22,-1.79)
Cote d'Ivoire	both	17182.82 (11524.48,24658.84)	14312.41 (9045.70,21125.26)	-16.71%	83.69 (56.40,120.09)	38.70 (24.49,57.46)	-2.54 (-2.70,-2.31)
Croatia	both	166.32 (81.12,285.85)	43.68 (21.25,77.15)	-73.74%	5.10 (2.56,8.62)	2.19 (1.09,3.86)	-3.29 (-3.50,-3.14)
Cuba	both	275.75 (136.66,470.98)	89.19 (42.62,166.19)	-67.66%	3.15 (1.55,5.42)	1.44 (0.70,2.66)	-2.68 (-2.79,-2.54)
Cyprus	both	12.29 (5.58,23.22)	1.90 (0.71,4.36)	-84.54%	1.81 (0.82,3.39)	0.26 (0.10,0.59)	-6.58 (-7.32,-5.94)
Czechia	both	367.36 (195.64,610.37)	113.13 (56.39,202.48)	-69.20%	5.08 (2.71,8.52)	1.94 (0.97,3.45)	-2.82 (-3.25,-2.38)
Democratic People's Republic of Korea	both	5635.82 (3371.62,8543.07)	1531.15 (857.32,2535.96)	-72.83%	22.60 (13.41,34.51)	8.82 (4.92,14.70)	-3.55 (-4.06,-3.14)

Democratic Republic of the Congo	both	48965.97 (31911.42,69056.27)	71779.78 (46476.22,103707.37)	46.59%	75.85 (48.96,106.89)	55.72 (36.28,80.23)	-0.64 (-1.12,0.00)
Denmark	both	18.57 (7.65,38.06)	5.84 (2.12,12.91)	-68.58%	0.63 (0.26,1.31)	0.18 (0.07,0.40)	-4.13 (-4.57,-3.69)
Djibouti	both	458.52 (290.61,674.68)	336.82 (201.76,505.90)	-26.54%	59.35 (37.23,87.12)	23.02 (13.79,34.22)	-3.50 (-3.75,-3.32)
Dominica	both	6.11 (3.83,9.45)	2.94 (1.84,4.45)	-51.94%	7.43 (4.68,11.40)	5.09 (3.15,7.74)	-1.34 (-1.61,-1.11)
Dominican Republic	both	1494.15 (912.80,2253.74)	735.04 (444.35,1124.80)	-50.81%	15.97 (9.85,24.02)	6.77 (4.08,10.35)	-3.24 (-3.43,-3.11)
Ecuador	both	1798.68 (1085.98,2819.69)	937.00 (585.61,1458.08)	-47.91%	13.72 (8.37,21.44)	5.28 (3.33,8.24)	-3.63 (-3.84,-3.47)
Egypt	both	8383.76 (5231.97,12640.74)	4195.99 (2572.18,6517.51)	-49.95%	10.95 (6.85,16.56)	3.84 (2.35,5.95)	-2.81 (-3.05,-2.43)
El Salvador	both	1328.64 (803.40,2041.32)	471.63 (288.58,720.62)	-64.50%	18.91 (11.45,29.08)	7.76 (4.77,11.86)	-3.02 (-3.36,-2.70)
Equatorial Guinea	both	908.50 (632.79,1297.76)	334.25 (206.72,490.15)	-63.21%	133.55 (93.00,188.52)	20.51 (12.90,30.29)	-7.49 (-8.06,-7.10)
Eritrea	both	3841.92 (2562.22,5533.90)	2830.35 (1737.33,4307.15)	-26.33%	78.00 (52.34,112.33)	31.76 (19.59,48.31)	-3.02 (-3.08,-2.96)
Estonia	both	7.80 (3.65,14.07)	1.00 (0.41,1.93)	-87.20%	0.64 (0.30,1.16)	0.14 (0.06,0.26)	-5.37 (-5.61,-5.14)
Eswatini	both	513.35 (324.92,779.75)	227.73 (145.20,339.31)	-55.64%	39.50 (25.07,59.41)	17.03 (11.03,25.25)	-2.77 (-2.84,-2.67)

Ethiopia	both	72098.96 (49181.15,101948.47)	62932.82 (41381.78,91811.66)	-12.71%	81.26 (55.60,114.65)	39.83 (26.28,57.96)	-2.53 (-2.85,-2.19)
Fiji	both	217.85 (134.20,335.16)	118.26 (70.32,197.38)	-45.72%	22.74 (14.07,34.78)	12.79 (7.60,21.36)	-1.62 (-1.74,-1.44)
Finland	both	26.38 (10.72,55.61)	5.61 (1.76,13.39)	-78.74%	0.81 (0.33,1.72)	0.20 (0.06,0.47)	-4.66 (-5.14,-4.18)
France	both	50.62 (18.80,113.58)	20.84 (8.08,44.28)	-58.84%	0.13 (0.05,0.28)	0.05 (0.02,0.11)	-2.40 (-2.64,-2.12)
Gabon	both	693.56 (435.07,1025.09)	303.21 (194.84,450.27)	-56.28%	48.99 (31.17,71.51)	16.05 (10.34,23.71)	-3.65 (-3.73,-3.53)
Gambia	both	1901.58 (1281.78,2725.76)	1685.52 (1108.09,2489.78)	-11.36%	113.22 (76.86,161.30)	52.84 (34.72,77.71)	-2.68 (-2.72,-2.65)
Georgia	both	98.66 (49.26,169.31)	46.32 (23.15,81.50)	-53.05%	2.08 (1.05,3.55)	1.82 (0.92,3.20)	0.04 (-0.54,0.80)
Germany	both	90.01 (37.16,181.49)	37.97 (15.99,79.37)	-57.82%	0.20 (0.08,0.40)	0.09 (0.04,0.20)	-2.35 (-2.53,-2.14)
Ghana	both	20738.89 (13730.06,29630.91)	13873.99 (8539.60,20597.31)	-33.10%	85.40 (56.30,122.82)	35.79 (22.12,52.76)	-3.04 (-3.34,-2.73)
Greece	both	75.26 (29.98,163.38)	17.74 (6.47,39.41)	-76.43%	1.18 (0.47,2.50)	0.37 (0.14,0.81)	-3.69 (-4.42,-2.96)
Greenland	both	0.51 (0.26,0.91)	0.21 (0.09,0.40)	-59.68%	1.00 (0.51,1.78)	0.51 (0.22,0.97)	-2.73 (-3.06,-2.41)
Grenada	both	18.49 (11.32,28.25)	6.09 (3.88,9.14)	-67.07%	17.10 (10.53,25.94)	7.17 (4.52,10.81)	-2.57 (-3.03,-2.10)
Guam	both	8.93 (4.89,15.34)	4.91 (2.50,8.09)	-44.96%	5.87 (3.18,10.09)	3.08 (1.57,5.09)	-2.10 (-2.32,-1.82)

Guatemala	both	2600.63 (1643.55,3866.07)	1822.73 (1149.44,2772.74)	-29.91%	20.53 (13.08,30.36)	9.26 (5.84,14.01)	-2.37 (-2.74,-1.99)
Guinea	both	9208.58 (6118.15,13347.55)	10196.65 (6502.35,14791.27)	10.73%	89.99 (59.76,131.16)	51.43 (33.01,74.77)	-1.78 (-1.83,-1.71)
Guinea-Bissau	both	1717.60 (1138.96,2449.01)	1528.67 (963.45,2256.94)	-11.00%	102.10 (67.67,146.56)	55.81 (35.62,82.65)	-2.00 (-2.05,-1.94)
Guyana	both	201.85 (121.23,313.15)	69.20 (41.11,103.02)	-65.72%	20.73 (12.54,32.14)	9.29 (5.52,13.85)	-2.62 (-2.75,-2.45)
Haiti	both	3819.61 (2388.19,5517.30)	3869.28 (2454.06,5857.92)	1.30%	40.50 (25.31,58.44)	26.12 (16.68,39.39)	-1.43 (-1.47,-1.37)
Honduras	both	1169.25 (740.06,1801.09)	773.14 (476.41,1190.55)	-33.88%	15.74 (9.98,23.87)	7.02 (4.38,10.78)	-2.66 (-2.88,-2.46)
Hungary	both	474.85 (253.06,842.85)	126.93 (64.42,232.10)	-73.27%	6.82 (3.76,11.72)	2.73 (1.38,4.98)	-3.06 (-3.30,-2.87)
Iceland	both	1.16 (0.48,2.52)	0.34 (0.12,0.72)	-71.06%	0.53 (0.22,1.13)	0.15 (0.05,0.31)	-4.25 (-4.75,-3.76)
India	both	689823.55 (467103.45,988266.94)	297500.77 (193958.30,434216.30)	-56.87%	60.56 (41.16,86.72)	22.90 (14.86,33.34)	-3.44 (-3.79,-3.04)
Indonesia	both	122711.99 (81033.01,182071.21)	42527.24 (27016.04,62814.43)	-65.34%	54.37 (36.05,80.08)	17.60 (11.24,25.68)	-4.01 (-4.13,-3.93)
Iran (Islamic Republic of)	both	9548.75 (5821.18,14855.10)	540.19 (283.98,986.02)	-94.34%	10.32 (6.32,16.04)	0.76 (0.40,1.38)	-8.73 (-9.25,-8.32)

Iraq	both	4949.46 (3053.72,7594.91)	3353.45 (2049.52,5099.02)	-32.25%	18.11 (11.34,27.25)	7.32 (4.50,11.05)	-3.32 (-3.53,-3.14)
Ireland	both	22.01 (9.01,43.42)	4.50 (1.73,9.45)	-79.56%	0.71 (0.29,1.41)	0.13 (0.05,0.28)	-5.43 (-5.91,-4.93)
Israel	both	364.46 (172.63,648.42)	204.19 (89.77,410.56)	-43.97%	6.86 (3.29,12.21)	2.21 (0.97,4.44)	-3.68 (-4.03,-3.28)
Italy	both	332.21 (148.35,614.16)	95.99 (37.44,200.58)	-71.10%	1.11 (0.50,2.08)	0.37 (0.15,0.76)	-3.46 (-3.97,-2.88)
Jamaica	both	273.85 (166.37,420.22)	137.44 (86.04,210.81)	-49.81%	9.83 (6.06,15.00)	5.77 (3.61,8.89)	-1.82 (-2.07,-1.61)
Japan	both	585.21 (287.50,1063.63)	192.54 (88.33,351.92)	-67.10%	0.76 (0.38,1.38)	0.36 (0.17,0.68)	-2.28 (-2.41,-2.11)
Jordan	both	884.56 (549.59,1333.59)	881.85 (538.02,1335.38)	-0.31%	16.19 (10.12,24.08)	7.13 (4.40,10.71)	-2.90 (-3.16,-2.67)
Kazakhstan	both	3235.39 (1953.63,5006.81)	1232.97 (689.09,2085.86)	-61.89%	17.68 (10.73,27.44)	6.78 (3.81,11.51)	-3.32 (-3.54,-3.02)
Kenya	both	25265.34 (16654.81,36105.87)	22486.03 (14572.41,33571.31)	-11.00%	64.28 (42.78,91.39)	34.95 (22.70,51.89)	-2.20 (-2.46,-1.95)
Kiribati	both	43.57 (27.78,65.72)	50.71 (31.41,76.56)	16.39%	41.40 (26.18,62.60)	34.82 (21.59,52.42)	-0.12 (-0.29,0.11)
Kuwait	both	130.20 (82.11,195.19)	197.20 (117.12,304.77)	51.46%	6.78 (4.33,10.08)	5.02 (3.05,7.74)	-1.10 (-1.16,-1.05)
Kyrgyzstan	both	634.15 (389.47,983.61)	661.25 (414.64,1010.24)	4.27%	11.51 (7.19,17.60)	9.27 (5.85,14.09)	-0.46 (-0.62,-0.23)
Lao People's Democratic Republic	both	3213.91 (2060.16,4726.52)	1215.61 (714.34,1926.20)	-62.18%	49.04 (31.40,71.95)	15.44 (9.10,24.49)	-3.97 (-4.25,-3.65)

Latvia	both	12.26 (6.00,22.33)	1.86 (0.82,3.52)	-84.83%	0.61 (0.30,1.11)	0.18 (0.08,0.33)	-4.16 (-4.25,-4.07)
Lebanon	both	296.69 (160.85,487.75)	52.22 (25.37,101.76)	-82.40%	6.58 (3.58,10.87)	1.05 (0.51,2.05)	-6.14 (-6.37,-5.88)
Lesotho	both	1465.12 (947.43,2209.01)	672.97 (427.88,1010.26)	-54.07%	57.02 (37.13,85.05)	30.14 (19.21,45.05)	-2.01 (-2.11,-1.85)
Liberia	both	2206.15 (1442.16,3235.27)	1742.71 (1077.27,2688.86)	-21.01%	70.00 (45.61,101.88)	27.80 (17.41,42.84)	-3.63 (-4.03,-3.31)
Libya	both	790.58 (498.00,1215.45)	418.14 (261.01,627.24)	-47.11%	13.22 (8.55,19.93)	7.04 (4.43,10.40)	-1.90 (-2.29,-1.54)
Lithuania	both	16.98 (8.12,32.14)	2.38 (1.01,4.49)	-85.99%	0.59 (0.28,1.11)	0.16 (0.07,0.31)	-4.24 (-4.36,-4.04)
Luxembourg	both	1.15 (0.50,2.28)	0.47 (0.17,1.05)	-58.90%	0.50 (0.21,0.99)	0.14 (0.05,0.31)	-4.12 (-4.69,-3.53)
Madagascar	both	11438.80 (7445.57,16361.23)	11299.86 (7130.36,16651.53)	-1.21%	58.18 (37.73,83.02)	30.27 (19.10,44.44)	-2.38 (-2.67,-2.09)
Malawi	both	12919.13 (8638.04,18291.55)	10794.67 (6723.82,15954.09)	-16.44%	82.40 (55.13,117.57)	41.04 (25.84,60.23)	-2.67 (-2.93,-2.45)
Malaysia	both	867.34 (475.28,1371.40)	125.55 (63.53,230.50)	-85.52%	3.68 (2.03,5.82)	0.48 (0.24,0.87)	-6.34 (-6.85,-5.71)
Maldives	both	246.54 (158.37,354.58)	28.46 (16.34,46.33)	-88.45%	63.84 (41.11,92.29)	6.96 (3.99,11.27)	-7.82 (-8.47,-7.29)
Mali	both	17027.94 (11436.09,23575.03)	26432.76 (17098.67,38164.85)	55.23%	117.42 (79.09,163.57)	73.09 (46.88,105.53)	-1.70 (-1.75,-1.67)

Malta	both	4.88 (2.16,9.78)	0.76 (0.29,1.60)	-84.46%	1.65 (0.73,3.29)	0.35 (0.13,0.73)	-4.89 (-5.26,-4.43)
Marshall Islands	both	43.85 (28.53,63.83)	18.16 (11.40,27.94)	-58.59%	56.93 (37.09,82.70)	28.91 (18.13,44.38)	-2.22 (-2.28,-2.17)
Mauritania	both	2179.35 (1434.55,3150.53)	1394.12 (846.25,2103.93)	-36.03%	65.80 (43.65,95.19)	26.12 (16.20,38.90)	-3.16 (-3.22,-3.11)
Mauritius	both	157.52 (95.46,244.50)	37.01 (23.46,56.47)	-76.51%	14.25 (8.55,22.12)	4.31 (2.63,6.65)	-3.71 (-4.07,-3.30)
Mexico	both	20183.52 (13077.28,29239.92)	11070.43 (7357.13,16184.13)	-45.15%	18.25 (11.94,26.25)	9.45 (6.31,13.81)	-2.24 (-2.38,-2.09)
Micronesia (Federated States of)	both	93.68 (60.67,135.39)	34.98 (21.77,53.17)	-62.66%	57.31 (37.34,82.88)	33.50 (20.78,50.52)	-1.66 (-1.74,-1.56)
Monaco	both	0.03 (0.01,0.08)	0.02 (0.01,0.04)	-50.17%	0.28 (0.10,0.63)	0.10 (0.04,0.24)	-3.19 (-3.64,-2.70)
Mongolia	both	349.34 (223.51,525.30)	211.37 (131.05,318.52)	-39.49%	12.40 (7.97,18.52)	6.04 (3.75,9.09)	-2.66 (-2.85,-2.53)
Montenegro	both	27.07 (14.86,47.11)	10.12 (5.41,18.41)	-62.61%	4.90 (2.67,8.58)	2.75 (1.48,4.98)	-2.76 (-3.45,-2.25)
Morocco	both	9535.47 (5878.64,14628.31)	3352.89 (1997.41,4962.45)	-64.84%	28.19 (17.41,43.16)	10.02 (5.91,14.94)	-3.48 (-3.62,-3.31)
Mozambique	both	21750.83 (14291.38,30649.78)	22856.59 (14520.40,34029.96)	5.08%	100.49 (65.88,141.77)	47.03 (30.02,70.04)	-2.81 (-3.03,-2.64)

Myanmar	both	34701.05 (22437.57,52664.26)	7410.30 (4450.24,11714.72)	-78.65%	61.83 (39.75,93.86)	14.39 (8.68,22.82)	-5.23 (-5.51,-5.02)
Namibia	both	572.11 (353.14,855.90)	409.15 (252.04,613.43)	-28.48%	28.97 (18.18,42.95)	14.77 (9.16,22.17)	-2.44 (-2.75,-2.10)
Nauru	both	3.30 (1.96,5.18)	1.82 (1.05,2.95)	-44.94%	20.58 (12.30,32.11)	13.51 (7.95,21.77)	-0.90 (-1.51,-0.05)
Nepal	both	11090.75 (7013.26,16635.05)	3320.82 (2026.94,5144.17)	-70.06%	35.80 (22.67,53.29)	10.70 (6.57,16.52)	-4.52 (-4.81,-4.33)
Netherlands	both	41.03 (15.73,91.21)	12.92 (4.72,31.16)	-68.51%	0.44 (0.17,0.97)	0.14 (0.05,0.34)	-3.56 (-3.95,-3.13)
New Zealand	both	13.59 (6.55,26.15)	8.22 (3.79,15.65)	-39.46%	0.49 (0.24,0.95)	0.27 (0.13,0.52)	-1.23 (-1.52,-0.81)
Nicaragua	both	534.12 (325.62,842.21)	254.56 (152.32,398.76)	-52.34%	9.23 (5.72,14.09)	3.77 (2.30,5.89)	-3.34 (-3.80,-2.97)
Niger	both	17764.85 (11634.97,25440.79)	37771.26 (24401.88,54540.75)	112.62%	123.61 (81.87,176.79)	88.75 (57.91,128.32)	-1.05 (-1.10,-0.98)
Nigeria	both	60647.24 (40044.03,87760.05)	52694.21 (34742.08,76155.54)	-13.11%	43.32 (28.87,62.84)	17.47 (11.66,25.37)	-3.36 (-3.77,-2.97)
Niue	both	0.47 (0.27,0.76)	0.11 (0.06,0.17)	-77.03%	16.74 (9.71,26.54)	7.70 (4.44,12.44)	-2.65 (-2.73,-2.57)
North Macedonia	both	304.12 (179.73,476.73)	75.60 (40.55,121.63)	-75.14%	16.49 (9.81,25.83)	6.38 (3.45,10.28)	-3.60 (-3.80,-3.46)

Northern Mariana Islands	both	2.37 (1.26,3.94)	0.98 (0.51,1.76)	-58.52%	5.20 (2.76,8.72)	4.09 (2.13,7.36)	0.14 (-0.21,0.65)
Norway	both	17.43 (7.52,34.99)	4.90 (1.89,10.58)	-71.91%	0.63 (0.27,1.27)	0.16 (0.06,0.33)	-4.62 (-5.18,-4.05)
Oman	both	953.56 (615.55,1451.14)	532.15 (333.33,788.30)	-44.19%	33.12 (21.50,49.97)	12.14 (7.71,18.04)	-3.03 (-3.61,-2.45)
Pakistan	both	84668.72 (55902.19,123245.66)	42649.04 (27687.54,62154.94)	-49.63%	48.59 (32.12,71.10)	16.06 (10.44,23.37)	-3.83 (-4.22,-3.49)
Palau	both	2.24 (1.34,3.50)	0.96 (0.58,1.48)	-57.37%	13.68 (8.20,21.47)	7.63 (4.55,12.04)	-1.61 (-1.75,-1.42)
Palestine	both	1334.48 (814.73,2018.94)	582.21 (357.43,876.37)	-56.37%	38.43 (23.25,58.10)	9.74 (6.04,14.48)	-4.38 (-4.72,-3.97)
Panama	both	201.24 (120.99,306.26)	204.76 (127.35,311.20)	1.75%	7.39 (4.50,11.10)	5.02 (3.12,7.60)	-1.35 (-1.51,-1.16)
Papua New Guinea	both	1441.34 (898.06,2129.93)	2230.98 (1326.96,3392.84)	54.78%	24.22 (15.10,35.67)	17.03 (10.13,25.73)	-0.61 (-0.84,-0.27)
Paraguay	both	948.05 (601.12,1451.59)	625.62 (383.15,936.59)	-34.01%	16.92 (10.93,25.66)	9.04 (5.58,13.49)	-2.03 (-2.10,-1.95)
Peru	both	6329.91 (4116.83,9207.66)	3174.50 (2031.35,4785.02)	-49.85%	22.30 (14.60,32.33)	9.72 (6.22,14.63)	-3.18 (-3.44,-2.97)
Philippines	both	19926.75 (12357.97,30098.59)	16143.85 (9670.26,25137.23)	-18.98%	22.68 (14.11,34.21)	13.16 (8.01,20.50)	-1.55 (-1.76,-1.23)

Poland	both	2807.88 (1526.77,4666.04)	536.01 (263.70,935.52)	-80.91%	8.70 (4.78,14.40)	2.66 (1.31,4.67)	-4.06 (-4.20,-3.96)
Portugal	both	180.36 (79.75,361.89)	22.45 (8.75,50.42)	-87.55%	2.73 (1.20,5.47)	0.50 (0.20,1.12)	-5.30 (-5.88,-4.66)
Puerto Rico	both	69.84 (34.01,123.27)	10.65 (5.16,19.43)	-84.76%	2.05 (1.00,3.63)	0.63 (0.31,1.14)	-4.38 (-4.62,-4.19)
Qatar	both	12.74 (6.74,21.47)	5.54 (2.51,10.62)	-56.49%	2.83 (1.49,4.77)	0.38 (0.17,0.72)	-6.62 (-6.88,-6.38)
Republic of Korea	both	724.83 (382.74,1186.98)	77.17 (35.84,139.77)	-89.35%	1.86 (0.99,3.04)	0.33 (0.16,0.60)	-5.36 (-5.89,-4.76)
Republic of Moldova	both	96.55 (51.38,169.14)	14.03 (6.92,25.48)	-85.47%	2.25 (1.21,3.94)	0.73 (0.36,1.30)	-3.86 (-4.29,-3.31)
Romania	both	1926.86 (1111.11,3031.61)	399.81 (214.16,642.66)	-79.25%	10.39 (5.98,16.36)	4.00 (2.15,6.48)	-3.47 (-3.76,-3.25)
Russian Federation	both	151.04 (78.24,266.46)	36.88 (16.89,70.59)	-75.59%	0.13 (0.07,0.22)	0.04 (0.02,0.08)	-4.06 (-4.36,-3.74)
Rwanda	both	4631.02 (2953.03,6852.55)	2633.73 (1588.95,3987.48)	-43.13%	38.80 (24.64,57.06)	16.43 (10.12,24.93)	-3.26 (-3.85,-2.71)
Saint Kitts and Nevis	both	4.02 (2.48,6.39)	2.53 (1.58,3.84)	-37.10%	8.54 (5.29,13.56)	5.20 (3.24,7.90)	-1.60 (-1.87,-1.33)
Saint Lucia	both	19.27 (11.71,30.09)	8.30 (5.09,12.56)	-56.96%	11.01 (6.70,16.96)	6.32 (3.78,9.67)	-1.71 (-2.00,-1.42)
Saint Vincent and the Grenadines	both	21.37 (12.93,33.30)	7.07 (4.44,10.59)	-66.92%	15.36 (9.28,23.99)	7.55 (4.71,11.39)	-2.42 (-2.73,-2.17)
Samoa	both	54.01 (32.58,83.09)	46.69 (28.07,75.33)	-13.54%	24.74 (15.11,37.71)	19.30 (11.69,31.05)	-0.48 (-0.64,-0.28)
San Marino	both	0.06 (0.02,0.14)	0.03 (0.01,0.06)	-57.51%	0.42 (0.16,0.93)	0.15 (0.05,0.34)	-3.30 (-3.91,-2.68)

Sao Tome and Principe	both	183.86 (115.75,272.67)	74.76 (41.32,121.18)	-59.34%	93.37 (59.24,137.96)	30.03 (16.84,48.50)	-3.97 (-4.09,-3.84)
Saudi Arabia	both	884.25 (470.89,1563.94)	32.17 (13.50,62.87)	-96.36%	3.59 (1.90,6.37)	0.14 (0.06,0.27)	-9.41 (-10.30,-8.21)
Senegal	both	13572.64 (9011.95,19308.13)	6125.21 (3764.15,9371.31)	-54.87%	106.70 (71.32,151.10)	29.86 (18.45,45.55)	-4.18 (-4.53,-3.75)
Serbia	both	1365.77 (804.27,2220.26)	376.05 (188.17,651.85)	-72.47%	19.20 (11.35,31.31)	7.67 (3.89,13.18)	-3.51 (-3.74,-3.35)
Seychelles	both	8.59 (5.03,13.35)	3.11 (1.89,4.85)	-63.79%	10.53 (6.19,16.35)	3.66 (2.20,5.78)	-3.19 (-3.67,-2.66)
Sierra Leone	both	5848.57 (3906.74,8265.63)	6723.20 (4399.57,9758.45)	14.95%	102.92 (68.89,144.08)	57.73 (37.69,83.64)	-1.93 (-2.07,-1.75)
Singapore	both	32.99 (16.07,64.95)	7.92 (3.36,15.62)	-76.01%	1.47 (0.72,2.88)	0.28 (0.12,0.55)	-5.66 (-5.94,-5.40)
Slovakia	both	315.54 (167.55,536.21)	72.34 (36.34,128.56)	-77.07%	7.09 (3.77,11.86)	2.47 (1.24,4.40)	-3.47 (-3.65,-3.30)
Slovenia	both	53.90 (28.01,92.24)	16.88 (8.02,30.96)	-68.68%	3.94 (2.06,6.69)	1.58 (0.76,2.92)	-3.33 (-3.53,-3.19)
Solomon Islands	both	305.40 (199.43,450.47)	342.80 (210.51,519.77)	12.25%	54.62 (35.74,80.49)	37.68 (23.04,57.27)	-0.92 (-1.09,-0.67)
Somalia	both	11402.26 (7584.25,16417.35)	27110.71 (17450.51,39502.20)	137.77%	94.45 (63.06,135.50)	80.92 (52.54,118.45)	-0.65 (-0.71,-0.60)
South Africa	both	10407.49 (6775.51,15082.30)	5237.85 (3447.32,7631.17)	-49.67%	23.54 (15.35,34.08)	9.71 (6.37,14.17)	-3.24 (-3.42,-3.08)

South Sudan	both	5756.72 (3752.89,8550.64)	4736.10 (3081.66,6964.92)	-17.73%	62.65 (40.84,92.88)	33.13 (21.44,48.46)	-2.35 (-2.54,-2.20)
Spain	both	251.71 (110.26,488.08)	59.75 (21.68,130.47)	-76.26%	1.04 (0.47,2.05)	0.27 (0.10,0.60)	-4.05 (-4.58,-3.48)
Sri Lanka	both	4704.44 (2765.09,7262.82)	874.81 (512.13,1399.85)	-81.40%	24.60 (14.46,37.84)	4.70 (2.72,7.51)	-6.08 (-6.31,-5.91)
Sudan	both	17337.32 (10908.76,25165.79)	9776.25 (5951.43,14693.17)	-43.61%	53.03 (33.62,77.17)	18.59 (11.35,27.84)	-3.41 (-3.67,-3.08)
Suriname	both	71.36 (43.00,109.01)	49.51 (30.87,73.33)	-30.62%	16.02 (9.74,24.21)	9.34 (5.81,13.89)	-2.06 (-2.17,-1.98)
Sweden	both	32.24 (13.92,64.92)	10.85 (3.97,22.94)	-66.36%	0.59 (0.25,1.22)	0.18 (0.07,0.37)	-3.60 (-4.15,-2.96)
Switzerland	both	11.58 (4.60,24.43)	5.07 (1.87,10.99)	-56.24%	0.29 (0.11,0.61)	0.11 (0.04,0.24)	-2.99 (-3.42,-2.51)
Syrian Arab Republic	both	3225.65 (1873.71,5385.13)	460.40 (236.73,808.35)	-85.73%	14.64 (8.48,24.67)	3.65 (1.88,6.52)	-4.78 (-5.13,-4.49)
Taiwan (Province of China)	both	332.26 (164.67,589.30)	33.05 (13.34,64.87)	-90.05%	1.81 (0.90,3.19)	0.32 (0.13,0.63)	-5.71 (-6.17,-5.31)
Tajikistan	both	1445.44 (891.41,2154.18)	1346.18 (841.54,1985.33)	-6.87%	18.23 (11.29,27.02)	12.19 (7.68,17.86)	-1.45 (-1.87,-1.06)
Thailand	both	11683.21 (7388.74,17448.10)	2503.94 (1480.63,4089.74)	-78.57%	20.54 (13.03,30.63)	5.19 (3.03,8.52)	-4.39 (-4.73,-3.99)
Timor-Leste	both	810.72 (493.77,1205.18)	322.89 (198.23,490.90)	-60.17%	63.78 (38.44,95.14)	18.82 (11.58,28.45)	-4.92 (-5.40,-4.59)

Togo	both	6649.90 (4377.97,9850.78)	4903.37 (3161.26,7391.59)	-26.26%	105.75 (69.67,156.45)	45.41 (29.27,68.05)	-2.67 (-2.75,-2.55)
Tokelau	both	0.77 (0.47,1.20)	0.21 (0.12,0.33)	-72.75%	30.17 (18.59,47.32)	12.63 (7.42,19.91)	-2.89 (-3.00,-2.73)
Tonga	both	28.50 (17.16,44.44)	14.50 (8.52,23.27)	-49.14%	21.35 (12.99,32.94)	11.92 (7.07,19.09)	-1.55 (-1.71,-1.32)
Trinidad and Tobago	both	109.50 (66.35,169.53)	57.65 (35.69,86.98)	-47.35%	8.02 (4.91,12.25)	5.22 (3.25,7.93)	-1.77 (-1.96,-1.63)
Tunisia	both	1039.52 (660.66,1584.24)	489.96 (305.15,743.84)	-52.87%	9.94 (6.39,14.80)	4.66 (2.92,7.10)	-2.47 (-2.72,-2.22)
Turkey	both	10218.73 (6263.10,16110.77)	1060.24 (540.72,1860.05)	-89.62%	13.51 (8.24,21.30)	2.03 (1.04,3.51)	-6.67 (-6.89,-6.51)
Turkmenistan	both	577.65 (372.96,860.11)	413.73 (265.01,618.54)	-28.38%	11.67 (7.50,17.25)	7.81 (5.00,11.63)	-1.41 (-1.50,-1.29)
Tuvalu	both	4.46 (2.74,6.75)	2.21 (1.32,3.47)	-50.41%	37.22 (22.93,56.20)	19.10 (11.42,30.03)	-1.82 (-1.97,-1.60)
Uganda	both	13698.94 (8924.58,20302.24)	11983.71 (7671.25,18016.62)	-12.52%	45.37 (29.94,67.34)	18.75 (12.06,28.28)	-3.16 (-3.34,-2.99)
Ukraine	both	241.19 (117.83,433.55)	62.22 (25.40,117.16)	-74.20%	0.62 (0.31,1.11)	0.27 (0.11,0.51)	-3.09 (-3.25,-2.95)
United Arab Emirates	both	57.25 (29.69,98.57)	22.73 (10.81,41.03)	-60.30%	2.67 (1.39,4.54)	0.58 (0.28,1.02)	-4.71 (-5.29,-4.15)
United Kingdom	both	363.72 (191.13,623.50)	138.01 (67.95,255.78)	-62.06%	0.95 (0.50,1.63)	0.34 (0.17,0.63)	-3.06 (-3.48,-2.55)
United Republic of Tanzania	both	23251.22 (15254.07,33122.90)	21971.71 (14105.11,32070.07)	-5.50%	55.55 (36.51,79.02)	27.14 (17.61,39.73)	-2.25 (-2.40,-2.08)

United States Virgin Islands	both	2.97 (1.60,5.16)	0.52 (0.26,0.93)	-82.40%	2.69 (1.44,4.66)	0.76 (0.38,1.34)	-4.61 (-5.12,-4.20)
United States of America	both	510.59 (274.52,895.97)	298.20 (144.14,542.26)	-41.60%	0.26 (0.14,0.46)	0.15 (0.07,0.26)	-1.96 (-2.19,-1.73)
Uruguay	both	287.41 (144.72,492.75)	98.85 (48.52,181.55)	-65.61%	10.31 (5.18,17.77)	4.18 (2.05,7.72)	-3.02 (-3.15,-2.85)
Uzbekistan	both	5168.16 (3127.62,7865.17)	3178.28 (1990.52,4706.16)	-38.50%	17.53 (10.78,26.43)	8.95 (5.63,13.20)	-2.27 (-2.33,-2.18)
Vanuatu	both	94.74 (60.09,141.69)	108.04 (65.61,163.99)	14.05%	39.22 (24.84,58.59)	28.30 (17.22,42.84)	-0.89 (-1.00,-0.73)
Venezuela (Bolivarian Republic of)	both	1985.70 (1212.76,3062.75)	1393.32 (852.34,2109.15)	-29.83%	8.52 (5.23,12.96)	5.43 (3.30,8.17)	-1.32 (-1.54,-1.06)
Viet Nam	both	13678.82 (8491.06,21110.60)	2777.38 (1679.51,4378.65)	-79.70%	14.97 (9.36,23.10)	3.60 (2.15,5.69)	-4.44 (-4.68,-4.09)
Yemen	both	17625.38 (11850.12,25278.82)	15568.53 (10106.65,23335.68)	-11.67%	67.21 (44.97,96.53)	36.30 (23.67,53.57)	-2.60 (-2.97,-2.34)
Zambia	both	9678.65 (6454.68,13861.11)	7871.04 (5013.57,11757.70)	-18.68%	75.30 (49.83,107.73)	31.64 (20.59,46.93)	-3.18 (-3.67,-2.69)

Zimbabwe	both	6512.27 (4074.55,9614.73)	6303.51 (4018.12,9570.99)	-3.21%	40.68 (25.68,60.44)	31.89 (20.67,48.25)	-0.18 (-0.47,0.21)
Afghanistan	male	4370.52 (2747.65,6776.40)	9291.69 (5438.99,14554.02)	112.60%	47.16 (29.41,73.60)	29.40 (17.20,45.89)	-1.62 (-2.01,-1.18)
Albania	male	505.11 (261.22,852.16)	62.63 (29.44,115.06)	-87.60%	24.36 (12.62,41.10)	7.24 (3.40,13.38)	-4.88 (-5.32,-4.57)
Algeria	male	3546.20 (1914.73,5972.12)	1622.05 (964.16,2546.73)	-54.26%	19.72 (11.01,32.82)	7.58 (4.52,11.92)	-3.25 (-3.46,-3.10)
American Samoa	male	3.92 (1.88,7.47)	1.71 (0.81,3.21)	-56.42%	10.71 (5.08,20.32)	6.01 (2.79,11.32)	-1.61 (-1.80,-1.34)
Andorra	male	0.06 (0.01,0.16)	0.02 (0.00,0.07)	-60.98%	0.38 (0.10,1.01)	0.14 (0.03,0.39)	-3.36 (-3.84,-2.87)
Angola	male	5047.45 (3203.27,7481.64)	7281.18 (4131.16,11875.34)	44.25%	62.59 (39.74,91.86)	32.91 (19.36,51.84)	-2.01 (-2.25,-1.66)
Antigua and Barbuda	male	2.59 (1.48,4.17)	1.82 (1.11,2.80)	-29.77%	8.09 (4.62,12.97)	5.12 (3.06,7.93)	-1.65 (-1.81,-1.53)
Argentina	male	2780.45 (1549.44,4476.36)	1159.67 (522.06,2289.45)	-58.29%	15.75 (8.75,25.37)	6.36 (2.86,12.44)	-3.16 (-3.31,-3.00)
Armenia	male	12.76 (5.56,24.49)	3.06 (1.12,6.44)	-76.04%	0.67 (0.30,1.29)	0.27 (0.10,0.56)	-3.15 (-3.56,-2.70)
Australia	male	1.51 (0.52,3.58)	0.72 (0.22,1.78)	-52.02%	0.02 (0.01,0.05)	0.01 (0.00,0.02)	-2.63 (-2.93,-2.27)
Austria	male	19.93 (6.10,48.83)	4.96 (1.05,14.44)	-75.12%	0.83 (0.25,2.03)	0.22 (0.05,0.64)	-4.44 (-4.91,-3.92)
Azerbaijan	male	219.93 (101.35,405.55)	82.53 (33.87,156.84)	-62.48%	4.89 (2.23,9.08)	1.92 (0.80,3.63)	-3.69 (-4.62,-2.79)

Bahamas	male	8.43 (4.79,13.42)	7.63 (4.57,11.92)	-9.50%	6.17 (3.61,9.77)	4.79 (2.89,7.47)	-0.94 (-1.12,-0.81)
Bahrain	male	14.79 (6.57,28.31)	4.32 (1.63,9.22)	-70.77%	4.83 (2.12,9.14)	1.09 (0.42,2.31)	-4.92 (-5.09,-4.79)
Bangladesh	male	36427.91 (21638.51,55474.55)	6468.47 (3618.66,10864.23)	-82.24%	40.93 (24.72,61.62)	8.53 (4.77,14.26)	-5.23 (-5.39,-4.99)
Barbados	male	5.72 (3.22,9.64)	3.75 (2.28,5.90)	-34.32%	4.96 (2.77,8.56)	3.56 (2.12,5.75)	-1.07 (-1.24,-0.93)
Belarus	male	56.68 (20.01,133.54)	7.36 (1.83,18.25)	-87.01%	1.34 (0.48,3.07)	0.25 (0.06,0.61)	-5.89 (-6.20,-5.57)
Belgium	male	27.17 (7.48,69.16)	6.37 (1.26,19.34)	-76.57%	0.86 (0.23,2.18)	0.19 (0.04,0.56)	-4.94 (-5.36,-4.49)
Belize	male	26.66 (14.59,45.41)	17.42 (10.11,27.43)	-34.67%	19.26 (10.72,32.43)	8.29 (4.82,13.23)	-2.67 (-2.93,-2.45)
Benin	male	5119.06 (3275.67,7622.95)	6738.77 (4034.41,10345.56)	31.64%	113.51 (73.20,169.62)	66.13 (39.81,101.19)	-1.91 (-2.16,-1.62)
Bermuda	male	0.36 (0.14,0.78)	0.08 (0.02,0.22)	-76.38%	1.70 (0.68,3.64)	0.57 (0.16,1.47)	-3.99 (-4.16,-3.85)
Bhutan	male	337.41 (195.59,520.73)	56.45 (30.13,94.65)	-83.27%	72.34 (41.75,111.39)	16.44 (8.73,27.47)	-5.25 (-5.41,-5.13)
Bolivia (Plurinational State of)	male	856.58 (487.66,1348.74)	900.37 (523.93,1488.98)	5.11%	18.56 (10.66,28.84)	12.95 (7.59,21.40)	-0.89 (-1.09,-0.64)
Bosnia and Herzegovina	male	313.71 (157.38,551.62)	32.65 (13.26,69.83)	-89.59%	16.27 (8.23,28.62)	3.98 (1.62,8.56)	-5.77 (-6.34,-5.33)
Botswana	male	478.71 (291.77,732.55)	254.03 (148.28,410.68)	-46.93%	51.32 (31.72,77.82)	20.88 (12.39,33.67)	-3.03 (-3.12,-2.93)

Brazil	male	21460.58 (12770.98,32758.68)	9094.03 (5362.25,14438.64)	-57.62%	23.90 (14.42,36.19)	10.22 (5.95,16.39)	-2.91 (-3.01,-2.83)
Brunei Darussalam	male	4.16 (1.79,8.42)	1.24 (0.43,2.80)	-70.15%	2.45 (1.06,4.91)	0.73 (0.25,1.63)	-4.15 (-4.43,-3.88)
Bulgaria	male	268.93 (115.86,547.41)	69.63 (27.53,142.52)	-74.11%	9.09 (3.94,18.68)	4.05 (1.61,8.27)	-2.70 (-3.01,-2.41)
Burkina Faso	male	13091.25 (8851.38,18918.34)	14099.62 (8305.24,21332.46)	7.70%	153.82 (103.52,221.62)	76.59 (45.53,115.70)	-2.69 (-2.87,-2.56)
Burundi	male	2561.21 (1529.62,3960.91)	3108.32 (1753.22,5008.42)	21.36%	53.83 (32.26,82.22)	32.79 (18.78,52.72)	-2.21 (-2.49,-2.00)
Cape Verde	male	181.39 (108.94,276.21)	35.83 (19.71,59.13)	-80.25%	66.37 (40.32,100.85)	12.74 (7.06,21.10)	-5.76 (-6.13,-5.48)
Cambodia	male	6799.48 (4200.31,10591.00)	1790.85 (940.29,3059.52)	-73.66%	76.89 (47.21,120.37)	19.92 (10.48,33.91)	-4.60 (-4.64,-4.56)
Cameroon	male	9478.47 (6078.86,13671.58)	10898.58 (6194.67,17247.44)	14.98%	108.86 (68.77,157.66)	51.63 (29.49,81.72)	-2.52 (-2.71,-2.30)
Canada	male	63.97 (23.16,135.58)	18.79 (4.85,46.77)	-70.62%	0.63 (0.23,1.34)	0.18 (0.05,0.45)	-4.31 (-4.82,-3.85)
Central African Republic	male	2663.85 (1701.40,4015.92)	3528.88 (2150.54,5281.05)	32.47%	117.92 (75.70,178.49)	89.85 (55.20,133.67)	-1.05 (-1.13,-0.99)

Chad	male	6922.56 (4473.77,10072.09)	12415.58 (7888.10,18139.80)	79.35%	131.70 (84.88,191.29)	84.29 (54.07,123.51)	-1.53 (-1.60,-1.44)
Chile	male	188.47 (61.10,443.97)	32.01 (7.50,91.36)	-83.02%	2.61 (0.85,6.15)	0.51 (0.12,1.40)	-5.32 (-5.59,-4.96)
China	male	80159.87 (49793.47,122986.34)	25020.49 (15739.20,38015.17)	-68.79%	13.30 (8.22,20.32)	4.42 (2.76,6.73)	-4.27 (-4.81,-3.80)
Colombia	male	1987.08 (1098.27,3350.97)	863.16 (524.28,1326.46)	-56.56%	10.03 (5.62,16.83)	3.97 (2.39,6.15)	-3.29 (-3.53,-3.10)
Comoros	male	261.52 (154.21,410.63)	126.16 (70.41,209.18)	-51.76%	69.93 (41.84,110.55)	31.41 (17.72,51.45)	-2.95 (-3.16,-2.79)
Congo	male	1844.84 (1157.70,2752.90)	2219.00 (1332.50,3483.58)	20.28%	99.53 (62.82,147.80)	66.34 (40.09,102.84)	-1.35 (-1.59,-1.05)
Cook Islands	male	1.70 (0.89,3.03)	0.52 (0.29,0.88)	-69.14%	14.47 (7.74,25.54)	6.74 (3.68,11.49)	-2.26 (-2.38,-2.09)
Costa Rica	male	139.42 (75.08,240.16)	78.46 (47.68,122.95)	-43.73%	7.35 (4.03,12.51)	3.78 (2.25,5.88)	-2.21 (-2.39,-2.02)
Cote d'Ivoire	male	10062.74 (6441.16,14602.79)	7689.45 (4377.41,12457.92)	-23.58%	95.75 (61.55,139.37)	40.39 (23.16,64.85)	-2.86 (-3.01,-2.66)
Croatia	male	84.33 (32.32,164.40)	21.17 (7.60,44.32)	-74.90%	5.07 (2.01,9.79)	2.08 (0.75,4.21)	-3.48 (-3.71,-3.32)
Cuba	male	155.57 (63.11,297.76)	49.40 (17.47,109.43)	-68.25%	3.47 (1.40,6.68)	1.55 (0.56,3.34)	-2.87 (-3.03,-2.71)
Cyprus	male	7.23 (2.49,16.80)	1.01 (0.21,3.07)	-86.10%	2.06 (0.73,4.68)	0.26 (0.05,0.79)	-6.97 (-7.75,-6.31)

Czechia	male	184.16 (78.03,359.63)	52.18 (19.53,108.88)	-71.67%	5.02 (2.09,9.62)	1.75 (0.65,3.65)	-3.15 (-3.55,-2.75)
Democratic People's Republic of Korea	male	3607.65 (1957.77,5979.64)	865.19 (424.51,1624.49)	-76.02%	28.14 (14.99,46.72)	9.81 (4.76,18.62)	-3.97 (-4.42,-3.62)
Democratic Republic of the Congo	male	29248.59 (18898.36,43410.89)	41722.73 (25471.56,61903.24)	42.65%	89.66 (57.37,132.00)	64.12 (39.37,94.29)	-0.80 (-1.24,-0.20)
Denmark	male	9.72 (2.71,25.78)	2.85 (0.64,8.61)	-70.73%	0.65 (0.18,1.73)	0.17 (0.04,0.53)	-4.50 (-4.95,-4.06)
Djibouti	male	290.23 (175.48,437.49)	191.71 (100.91,308.06)	-33.94%	70.79 (42.82,106.66)	24.61 (13.10,39.07)	-3.97 (-4.26,-3.77)
Dominica	male	2.98 (1.71,4.89)	1.39 (0.84,2.12)	-53.34%	7.15 (4.17,11.57)	4.65 (2.78,7.15)	-1.45 (-1.75,-1.19)
Dominican Republic	male	796.72 (464.18,1291.13)	357.51 (209.33,565.16)	-55.13%	17.00 (9.93,27.59)	6.50 (3.78,10.32)	-3.61 (-3.83,-3.45)
Ecuador	male	985.63 (545.40,1675.23)	447.51 (259.08,720.98)	-54.60%	14.84 (8.44,24.84)	5.02 (2.92,8.09)	-4.03 (-4.25,-3.85)
Egypt	male	4497.00 (2717.08,7273.67)	2048.85 (1156.05,3339.57)	-54.44%	11.37 (6.85,18.71)	3.64 (2.10,5.81)	-3.21 (-3.46,-2.84)
El Salvador	male	741.58 (418.23,1191.80)	234.90 (133.40,382.81)	-68.32%	20.91 (11.90,33.13)	7.84 (4.48,12.64)	-3.36 (-3.71,-3.06)
Equatorial Guinea	male	516.67 (344.93,751.03)	197.70 (119.01,299.86)	-61.74%	149.57 (101.85,217.52)	22.25 (13.83,33.34)	-7.65 (-8.24,-7.26)
Eritrea	male	2282.58 (1483.69,3342.21)	1626.16 (909.88,2640.37)	-28.76%	90.46 (58.57,132.56)	35.89 (20.24,57.87)	-3.14 (-3.22,-3.07)

Estonia	male	4.13 (1.48,8.79)	0.43 (0.12,1.12)	-89.49%	0.67 (0.24,1.42)	0.12 (0.03,0.30)	-6.20 (-6.48,-5.97)
Eswatini	male	303.41 (183.03,473.15)	129.27 (75.61,209.74)	-57.39%	46.67 (28.70,72.87)	19.08 (11.36,30.68)	-3.00 (-3.04,-2.94)
Ethiopia	male	40345.56 (26771.53,57866.68)	33698.17 (20700.98,51420.38)	-16.48%	88.15 (58.98,125.61)	41.67 (25.76,62.98)	-2.68 (-3.02,-2.33)
Fiji	male	146.97 (81.77,240.84)	83.37 (44.33,149.80)	-43.27%	29.72 (16.70,48.72)	17.59 (9.41,31.47)	-1.37 (-1.51,-1.16)
Finland	male	15.91 (3.96,43.66)	2.99 (0.51,9.80)	-81.20%	0.96 (0.24,2.67)	0.21 (0.04,0.64)	-5.18 (-5.71,-4.68)
France	male	29.46 (5.92,82.96)	11.38 (3.15,32.08)	-61.38%	0.14 (0.03,0.40)	0.06 (0.02,0.16)	-2.78 (-2.98,-2.54)
Gabon	male	440.94 (260.39,671.91)	167.10 (99.54,269.40)	-62.10%	61.60 (37.16,93.59)	17.89 (10.82,28.32)	-4.07 (-4.16,-3.95)
Gambia	male	1176.41 (786.30,1666.35)	993.39 (571.59,1536.33)	-15.56%	139.63 (93.08,198.96)	61.72 (35.93,95.56)	-2.92 (-2.99,-2.86)
Georgia	male	56.19 (22.99,108.37)	24.13 (9.42,50.86)	-57.05%	2.33 (0.97,4.49)	1.82 (0.71,3.75)	-0.50 (-1.13,0.28)
Germany	male	50.88 (14.03,123.26)	20.24 (6.21,52.48)	-60.22%	0.22 (0.06,0.53)	0.10 (0.03,0.26)	-2.66 (-2.87,-2.45)
Ghana	male	13343.18 (8498.65,19709.82)	8754.26 (5080.32,13923.06)	-34.39%	108.10 (68.59,159.66)	44.34 (25.84,70.29)	-3.10 (-3.32,-2.87)
Greece	male	44.09 (11.58,119.06)	9.31 (2.16,25.98)	-78.88%	1.33 (0.37,3.44)	0.38 (0.09,1.04)	-4.14 (-4.93,-3.42)
Greenland	male	0.16 (0.05,0.38)	0.08 (0.02,0.22)	-50.57%	0.62 (0.20,1.43)	0.39 (0.11,1.05)	-2.49 (-3.16,-1.91)
Grenada	male	10.35 (5.82,17.06)	3.12 (1.89,4.94)	-69.88%	18.94 (10.80,31.05)	7.15 (4.26,11.72)	-2.93 (-3.42,-2.42)

Guam	male	5.16 (2.27,9.89)	2.85 (1.16,5.63)	-44.68%	6.64 (2.95,12.98)	3.47 (1.41,6.85)	-2.14 (-2.33,-1.88)
Guatemala	male	1366.40 (804.12,2175.29)	824.23 (475.18,1345.02)	-39.68%	20.90 (12.55,32.88)	8.40 (4.89,13.61)	-2.66 (-3.09,-2.22)
Guinea	male	5935.64 (3857.11,8717.51)	6350.70 (3917.91,9701.23)	6.99%	114.24 (73.65,167.64)	63.10 (39.14,96.48)	-1.92 (-1.97,-1.85)
Guinea-Bissau	male	1083.27 (702.77,1576.00)	950.54 (557.51,1487.41)	-12.25%	127.61 (82.57,185.50)	68.52 (40.62,106.59)	-2.14 (-2.17,-2.10)
Guyana	male	115.61 (64.52,195.48)	36.04 (20.50,59.18)	-68.82%	23.78 (13.36,39.90)	9.64 (5.48,15.93)	-2.96 (-3.11,-2.77)
Haiti	male	2109.59 (1262.15,3204.95)	2036.80 (1167.51,3352.51)	-3.45%	44.98 (26.99,68.38)	27.41 (15.91,44.85)	-1.67 (-1.70,-1.64)
Honduras	male	650.71 (373.64,1083.34)	369.15 (197.17,614.61)	-43.27%	17.07 (10.00,27.82)	6.70 (3.66,11.03)	-3.05 (-3.31,-2.81)
Hungary	male	240.70 (112.41,469.47)	58.94 (22.58,130.30)	-75.52%	6.81 (3.18,13.00)	2.47 (0.95,5.48)	-3.42 (-3.62,-3.28)
Iceland	male	0.64 (0.18,1.72)	0.17 (0.03,0.51)	-74.08%	0.57 (0.16,1.55)	0.14 (0.03,0.42)	-4.79 (-5.28,-4.36)
India	male	418794.92 (268487.16,615828.18)	162256.22 (101295.40,246859.59)	-61.26%	70.27 (45.53,102.47)	24.13 (14.97,36.62)	-3.78 (-4.13,-3.39)
Indonesia	male	70981.02 (45002.25,107452.71)	20916.43 (13095.17,30933.55)	-70.53%	61.33 (38.99,91.97)	17.13 (10.70,25.25)	-4.59 (-4.73,-4.47)

Iran (Islamic Republic of)	male	6023.96 (3308.58,10108.92)	305.63 (127.65,647.42)	-94.93%	12.72 (7.04,21.26)	0.83 (0.35,1.75)	-9.17 (-9.74,-8.74)
Iraq	male	2896.56 (1596.76,4758.03)	1812.56 (1058.08,2935.54)	-37.42%	20.49 (11.77,33.16)	7.73 (4.57,12.53)	-3.62 (-3.87,-3.38)
Ireland	male	13.06 (3.74,33.08)	2.45 (0.69,6.48)	-81.23%	0.81 (0.23,2.02)	0.14 (0.04,0.37)	-5.78 (-6.26,-5.30)
Israel	male	229.55 (93.27,461.39)	122.14 (36.62,301.13)	-46.79%	8.41 (3.45,16.89)	2.58 (0.77,6.39)	-3.89 (-4.28,-3.49)
Italy	male	189.26 (58.06,437.00)	55.54 (14.75,139.87)	-70.65%	1.23 (0.38,2.86)	0.41 (0.11,1.02)	-3.53 (-4.01,-3.02)
Jamaica	male	139.47 (78.30,233.95)	65.60 (38.46,103.88)	-52.96%	10.03 (5.74,16.59)	5.46 (3.16,8.70)	-2.09 (-2.38,-1.85)
Japan	male	245.62 (82.97,550.94)	76.23 (23.74,190.74)	-68.96%	0.63 (0.22,1.40)	0.28 (0.09,0.69)	-2.60 (-2.71,-2.45)
Jordan	male	497.16 (276.10,800.54)	477.66 (272.11,752.43)	-3.92%	17.59 (9.94,27.72)	7.43 (4.32,11.63)	-3.07 (-3.36,-2.82)
Kazakhstan	male	2196.40 (1228.77,3585.60)	787.18 (370.41,1460.00)	-64.16%	23.66 (13.24,38.88)	8.44 (4.00,15.63)	-3.66 (-3.88,-3.42)
Kenya	male	14105.70 (9272.95,20432.79)	12552.06 (7837.14,19240.41)	-11.01%	71.18 (46.65,103.14)	38.54 (24.33,58.78)	-2.24 (-2.47,-2.03)
Kiribati	male	25.63 (15.02,41.04)	29.77 (16.59,47.29)	16.16%	47.21 (27.87,75.22)	39.64 (22.16,62.61)	-0.12 (-0.29,0.08)
Kuwait	male	70.47 (42.18,110.02)	104.58 (59.97,163.96)	48.41%	6.80 (4.19,10.52)	5.02 (2.98,7.84)	-1.16 (-1.26,-1.07)
Kyrgyzstan	male	361.63 (213.67,593.84)	336.16 (196.21,539.15)	-7.04%	12.95 (7.70,21.00)	9.32 (5.54,14.77)	-0.92 (-1.06,-0.74)

Lao People's Democratic Republic	male	1920.75 (1146.42,2941.78)	648.74 (342.51,1098.73)	-66.22%	57.44 (34.07,88.68)	16.19 (8.60,27.38)	-4.39 (-4.67,-4.09)
Latvia	male	6.26 (2.14,13.60)	0.77 (0.23,1.86)	-87.61%	0.61 (0.21,1.32)	0.14 (0.04,0.34)	-5.05 (-5.20,-4.94)
Lebanon	male	165.92 (76.87,298.34)	26.15 (8.63,62.12)	-84.24%	7.05 (3.23,12.89)	1.01 (0.33,2.39)	-6.53 (-6.77,-6.26)
Lesotho	male	854.66 (543.18,1319.25)	401.55 (233.85,641.14)	-53.02%	66.26 (42.52,101.42)	35.94 (21.12,56.99)	-1.91 (-2.01,-1.78)
Liberia	male	1651.20 (1050.75,2424.84)	1218.96 (687.57,2008.59)	-26.18%	103.47 (65.51,151.87)	37.82 (21.42,62.07)	-3.91 (-4.21,-3.67)
Libya	male	426.91 (249.67,698.63)	223.45 (131.91,345.59)	-47.66%	14.20 (8.47,22.81)	7.29 (4.33,11.37)	-2.07 (-2.49,-1.69)
Lithuania	male	8.74 (3.15,19.62)	0.98 (0.26,2.31)	-88.80%	0.59 (0.22,1.33)	0.13 (0.04,0.31)	-5.18 (-5.30,-5.04)
Luxembourg	male	0.61 (0.16,1.50)	0.24 (0.05,0.67)	-61.25%	0.52 (0.14,1.27)	0.14 (0.03,0.38)	-4.40 (-4.99,-3.84)
Madagascar	male	6712.21 (4023.95,9848.53)	6094.65 (3553.32,9420.58)	-9.20%	67.20 (40.57,99.08)	32.19 (18.77,49.40)	-2.74 (-3.02,-2.49)
Malawi	male	6960.06 (4545.39,9990.25)	5043.88 (2944.30,8098.15)	-27.53%	87.88 (57.25,127.11)	39.00 (22.91,62.29)	-3.16 (-3.42,-2.97)
Malaysia	male	506.23 (247.75,882.55)	56.59 (21.19,121.40)	-88.82%	4.16 (2.03,7.20)	0.42 (0.15,0.90)	-7.29 (-7.79,-6.67)
Maldives	male	149.80 (89.49,227.17)	14.49 (7.49,25.80)	-90.33%	75.57 (45.40,114.93)	6.68 (3.34,12.04)	-8.71 (-9.41,-8.14)

Mali	male	10569.60 (7017.71,14550.44)	15609.88 (9460.55,23260.55)	47.69%	143.47 (94.26,198.89)	85.23 (51.99,127.50)	-1.89 (-1.99,-1.82)
Malta	male	2.87 (0.90,7.45)	0.41 (0.09,1.10)	-85.86%	1.89 (0.60,4.93)	0.36 (0.08,0.96)	-5.29 (-5.64,-4.86)
Marshall Islands	male	25.45 (15.57,37.86)	11.06 (6.36,17.57)	-56.54%	64.28 (39.32,94.94)	34.31 (19.90,54.73)	-2.04 (-2.09,-1.97)
Mauritania	male	1399.91 (890.30,2074.70)	815.64 (447.82,1319.31)	-41.74%	82.52 (52.24,122.32)	30.13 (16.91,48.02)	-3.55 (-3.63,-3.48)
Mauritius	male	88.76 (48.17,152.78)	17.98 (10.47,29.59)	-79.75%	15.91 (8.69,27.56)	4.10 (2.27,7.09)	-4.34 (-4.75,-3.89)
Mexico	male	11413.54 (7348.61,16647.53)	5468.92 (3529.11,8246.82)	-52.08%	20.40 (12.99,29.54)	9.39 (6.09,14.14)	-2.61 (-2.78,-2.45)
Micronesia (Federated States of)	male	55.01 (33.74,82.52)	20.54 (12.00,32.90)	-62.67%	64.72 (39.90,96.67)	38.19 (22.42,61.25)	-1.66 (-1.77,-1.54)
Monaco	male	0.02 (0.00,0.05)	0.01 (0.00,0.02)	-54.10%	0.28 (0.06,0.85)	0.10 (0.02,0.27)	-3.41 (-3.87,-2.92)
Mongolia	male	213.50 (129.01,334.67)	112.87 (67.17,170.85)	-47.14%	14.88 (9.29,22.96)	6.47 (3.88,9.83)	-3.08 (-3.31,-2.93)
Montenegro	male	13.62 (5.77,26.98)	4.51 (1.81,9.23)	-66.87%	4.81 (2.05,9.62)	2.36 (0.96,4.80)	-3.36 (-4.14,-2.79)
Morocco	male	5615.71 (3168.30,9070.10)	1823.69 (1024.05,2950.11)	-67.53%	32.71 (18.67,52.68)	10.74 (6.01,17.59)	-3.80 (-3.96,-3.63)

Mozambique	male	12826.15 (8392.41,18332.41)	13121.54 (7918.06,20139.19)	2.30%	118.49 (77.41,168.27)	54.10 (32.67,83.71)	-2.96 (-3.16,-2.80)
Myanmar	male	21366.95 (13116.63,33167.44)	4003.00 (2114.52,6922.67)	-81.27%	75.22 (46.00,116.56)	15.29 (8.12,26.56)	-5.82 (-6.14,-5.58)
Namibia	male	326.58 (186.01,506.89)	221.08 (129.34,351.15)	-32.30%	33.13 (19.62,51.27)	16.16 (9.68,25.36)	-2.58 (-2.84,-2.27)
Nauru	male	1.97 (1.01,3.27)	1.11 (0.55,2.02)	-43.85%	23.84 (12.35,40.51)	15.88 (8.06,28.68)	-0.86 (-1.48,0.01)
Nepal	male	5708.06 (3346.65,9097.42)	1286.79 (694.01,2154.53)	-77.46%	35.53 (20.75,56.30)	8.30 (4.54,13.90)	-5.50 (-5.81,-5.28)
Netherlands	male	19.28 (4.12,56.93)	5.49 (1.09,18.55)	-71.51%	0.40 (0.08,1.19)	0.12 (0.02,0.39)	-3.94 (-4.35,-3.49)
New Zealand	male	7.90 (2.96,17.83)	4.72 (1.49,10.53)	-40.18%	0.56 (0.21,1.26)	0.31 (0.10,0.69)	-1.35 (-1.61,-0.97)
Nicaragua	male	301.78 (167.54,514.29)	108.77 (63.74,172.71)	-63.96%	10.18 (5.79,16.96)	3.27 (1.94,5.13)	-4.09 (-4.63,-3.66)
Niger	male	10322.80 (6727.62,14688.61)	21501.84 (13442.95,31734.62)	108.29%	142.43 (93.43,202.23)	99.54 (62.15,147.23)	-1.18 (-1.22,-1.13)
Nigeria	male	35202.66 (22109.44,51547.56)	25754.27 (15617.08,39625.60)	-26.84%	51.61 (32.37,75.43)	17.31 (10.67,26.17)	-4.05 (-4.43,-3.72)
Niue	male	0.29 (0.15,0.50)	0.06 (0.03,0.11)	-77.94%	19.73 (10.24,33.65)	8.86 (4.32,15.84)	-2.74 (-2.81,-2.66)
North Macedonia	male	178.58 (95.94,304.82)	43.16 (20.92,76.18)	-75.83%	18.82 (10.14,32.11)	7.06 (3.43,12.38)	-3.79 (-4.01,-3.64)

Northern Mariana Islands	male	1.34 (0.60,2.46)	0.59 (0.26,1.21)	-55.93%	5.88 (2.60,11.33)	4.80 (2.07,9.95)	0.30 (-0.05,0.82)
Norway	male	11.68 (3.95,27.13)	3.03 (0.84,8.24)	-74.06%	0.82 (0.28,1.92)	0.19 (0.05,0.49)	-5.04 (-5.60,-4.48)
Oman	male	561.09 (333.19,884.33)	320.65 (193.06,483.41)	-42.85%	37.56 (22.62,59.06)	12.29 (7.55,18.37)	-3.41 (-4.07,-2.73)
Pakistan	male	57191.62 (36956.13,83895.55)	24835.82 (15805.97,37255.51)	-56.57%	62.66 (40.41,91.48)	18.25 (11.66,27.06)	-4.29 (-4.75,-3.86)
Palau	male	1.34 (0.71,2.29)	0.57 (0.32,0.95)	-57.60%	15.98 (8.43,27.23)	8.78 (4.78,15.10)	-1.65 (-1.79,-1.45)
Palestine	male	834.91 (471.89,1337.83)	312.09 (182.65,506.81)	-62.62%	46.17 (25.90,73.95)	10.18 (6.02,16.20)	-4.93 (-5.35,-4.46)
Panama	male	101.51 (57.25,166.31)	92.43 (53.86,145.35)	-8.95%	7.37 (4.21,11.84)	4.47 (2.61,7.07)	-1.67 (-1.89,-1.43)
Papua New Guinea	male	813.24 (461.69,1313.31)	1243.35 (667.50,2054.44)	52.89%	26.11 (15.04,41.69)	18.14 (9.72,29.58)	-0.64 (-0.88,-0.30)
Paraguay	male	483.66 (267.63,807.07)	303.61 (174.70,478.50)	-37.23%	16.93 (9.73,27.56)	8.62 (5.00,13.65)	-2.17 (-2.24,-2.09)
Peru	male	3611.86 (2177.45,5544.82)	1754.14 (1051.51,2756.97)	-51.43%	25.14 (15.15,38.27)	10.53 (6.27,16.45)	-3.36 (-3.65,-3.16)
Philippines	male	11111.46 (6590.13,17309.35)	9112.73 (5143.82,14807.10)	-17.99%	24.59 (14.65,38.18)	14.42 (8.19,23.34)	-1.54 (-1.74,-1.24)
Poland	male	1397.10 (639.20,2567.26)	234.35 (90.67,506.88)	-83.23%	8.54 (3.92,15.63)	2.27 (0.89,4.89)	-4.56 (-4.65,-4.49)

Portugal	male	108.04 (34.46,268.70)	12.35 (2.96,31.33)	-88.56%	3.18 (1.03,7.56)	0.53 (0.13,1.34)	-5.65 (-6.27,-4.98)
Puerto Rico	male	34.57 (13.17,72.56)	4.81 (1.55,11.11)	-86.08%	2.00 (0.75,4.22)	0.56 (0.19,1.26)	-4.68 (-4.95,-4.48)
Qatar	male	6.29 (2.65,12.36)	2.25 (0.57,5.69)	-64.18%	2.71 (1.13,5.28)	0.30 (0.08,0.75)	-7.30 (-7.59,-7.04)
Republic of Korea	male	313.14 (126.65,622.87)	23.43 (6.55,60.85)	-92.52%	1.53 (0.64,3.04)	0.19 (0.05,0.49)	-6.68 (-7.28,-6.06)
Republic of Moldova	male	49.58 (20.48,103.30)	6.02 (2.02,12.60)	-87.85%	2.27 (0.96,4.66)	0.61 (0.22,1.26)	-4.62 (-5.11,-4.08)
Romania	male	964.54 (485.11,1689.62)	181.06 (75.29,356.75)	-81.23%	10.26 (5.20,17.91)	3.54 (1.47,7.01)	-3.92 (-4.25,-3.66)
Russian Federation	male	97.95 (40.91,202.88)	21.05 (7.10,50.30)	-78.51%	0.16 (0.07,0.33)	0.04 (0.02,0.10)	-4.62 (-4.98,-4.27)
Rwanda	male	2448.01 (1408.15,3756.50)	1254.87 (666.01,2140.68)	-48.74%	41.19 (23.60,63.88)	15.87 (8.72,26.61)	-3.69 (-4.28,-3.17)
Saint Kitts and Nevis	male	2.07 (1.12,3.51)	1.22 (0.74,1.92)	-40.86%	8.80 (4.80,14.73)	4.95 (2.91,7.82)	-1.84 (-2.16,-1.53)
Saint Lucia	male	9.80 (5.55,16.60)	4.07 (2.38,6.41)	-58.53%	11.27 (6.51,18.85)	6.07 (3.48,9.84)	-1.91 (-2.24,-1.60)
Saint Vincent and the Grenadines	male	11.76 (6.23,20.25)	3.53 (2.06,5.68)	-69.98%	16.79 (8.93,28.74)	7.40 (4.24,12.12)	-2.80 (-3.15,-2.52)
Samoa	male	33.70 (18.59,55.77)	29.00 (15.47,50.20)	-13.96%	29.47 (16.60,47.91)	23.11 (12.78,39.08)	-0.47 (-0.62,-0.27)
San Marino	male	0.03 (0.01,0.09)	0.01 (0.00,0.04)	-60.63%	0.44 (0.11,1.23)	0.14 (0.03,0.43)	-3.70 (-4.31,-3.10)
Sao Tome and Principe	male	131.02 (78.95,198.07)	52.42 (25.99,90.19)	-59.99%	130.93 (79.06,197.50)	41.33 (20.78,70.24)	-4.04 (-4.15,-3.91)

Saudi Arabia	male	565.15 (271.76,1137.30)	17.37 (4.80,40.68)	-96.93%	4.53 (2.17,9.03)	0.14 (0.04,0.33)	-10.04 (-10.92,-8.85)
Senegal	male	9702.97 (6487.23,13740.03)	4112.70 (2288.61,6817.35)	-57.61%	152.86 (101.06,217.07)	38.66 (21.54,63.56)	-4.61 (-5.06,-4.13)
Serbia	male	726.53 (360.57,1268.42)	207.24 (80.85,405.08)	-71.47%	21.39 (10.81,37.65)	8.07 (3.24,15.88)	-3.75 (-4.01,-3.56)
Seychelles	male	4.90 (2.62,8.38)	1.56 (0.88,2.64)	-68.22%	11.86 (6.37,20.22)	3.52 (1.95,6.01)	-3.75 (-4.30,-3.15)
Sierra Leone	male	3763.71 (2488.62,5306.49)	4029.04 (2520.95,6141.81)	7.05%	132.71 (87.30,187.18)	68.97 (43.42,105.63)	-2.24 (-2.36,-2.10)
Singapore	male	14.90 (5.00,35.27)	2.92 (0.70,7.67)	-80.40%	1.30 (0.41,3.07)	0.21 (0.05,0.54)	-6.43 (-6.75,-6.16)
Slovakia	male	163.01 (72.47,309.57)	32.99 (11.98,71.19)	-79.76%	7.21 (3.26,13.82)	2.20 (0.80,4.79)	-3.97 (-4.12,-3.84)
Slovenia	male	26.95 (10.83,54.00)	7.51 (2.57,16.97)	-72.12%	3.86 (1.61,7.73)	1.38 (0.48,3.11)	-3.77 (-3.97,-3.64)
Solomon Islands	male	184.64 (116.47,282.49)	209.81 (121.64,332.84)	13.63%	63.50 (39.72,97.79)	44.35 (25.62,70.58)	-0.89 (-1.04,-0.67)
Somalia	male	6488.63 (4174.89,9541.71)	14843.86 (9367.46,21906.37)	128.77%	101.23 (65.04,148.49)	86.01 (54.39,126.82)	-0.70 (-0.77,-0.65)
South Africa	male	5996.03 (3648.90,9314.16)	2733.20 (1689.70,4139.74)	-54.42%	27.30 (16.97,41.85)	10.20 (6.31,15.50)	-3.65 (-3.85,-3.50)

South Sudan	male	3313.77 (2086.00,5021.31)	2525.00 (1488.25,3981.11)	-23.80%	69.17 (43.77,105.10)	33.92 (20.21,53.22)	-2.69 (-2.93,-2.49)
Spain	male	171.78 (56.89,377.67)	42.84 (11.22,109.19)	-75.06%	1.38 (0.47,2.93)	0.38 (0.10,0.91)	-4.03 (-4.48,-3.54)
Sri Lanka	male	3150.94 (1719.44,5042.09)	459.49 (253.72,775.31)	-85.42%	32.37 (18.07,51.74)	4.92 (2.71,8.34)	-6.88 (-7.16,-6.67)
Sudan	male	10719.58 (6618.03,16102.61)	5585.30 (3130.75,9132.80)	-47.90%	62.59 (37.74,94.72)	20.62 (11.64,33.31)	-3.70 (-3.94,-3.38)
Suriname	male	38.99 (21.50,65.79)	24.67 (14.11,39.30)	-36.72%	17.46 (9.77,28.84)	9.20 (5.23,14.66)	-2.43 (-2.56,-2.32)
Sweden	male	18.21 (4.92,46.84)	5.61 (1.22,16.31)	-69.22%	0.65 (0.17,1.70)	0.18 (0.04,0.50)	-4.05 (-4.61,-3.41)
Switzerland	male	5.86 (1.24,15.80)	2.45 (0.65,6.73)	-58.15%	0.29 (0.06,0.77)	0.11 (0.03,0.29)	-3.24 (-3.66,-2.78)
Syrian Arab Republic	male	1900.67 (921.61,3542.25)	260.70 (110.47,511.24)	-86.28%	16.75 (8.10,31.14)	4.08 (1.70,8.20)	-4.89 (-5.24,-4.61)
Taiwan (Province of China)	male	198.39 (80.54,398.66)	15.37 (4.02,37.54)	-92.25%	2.10 (0.85,4.22)	0.29 (0.08,0.70)	-6.63 (-7.21,-6.14)
Tajikistan	male	837.28 (488.77,1325.64)	700.14 (407.06,1092.19)	-16.38%	20.61 (12.31,32.80)	12.41 (7.37,19.36)	-1.93 (-2.41,-1.52)
Thailand	male	6162.31 (3771.67,9537.94)	1288.35 (752.04,2175.54)	-79.09%	21.36 (13.00,32.90)	5.29 (2.94,9.06)	-4.52 (-4.75,-4.23)
Timor-Leste	male	481.55 (287.38,741.31)	182.03 (99.77,306.31)	-62.20%	73.73 (43.99,115.06)	20.72 (11.50,34.50)	-5.11 (-5.60,-4.76)

Togo	male	4551.23 (2977.20,6744.22)	3068.03 (1785.28,4789.41)	-32.59%	142.82 (93.16,212.65)	55.47 (32.50,86.19)	-3.08 (-3.16,-2.96)
Tokelau	male	0.46 (0.26,0.77)	0.13 (0.06,0.22)	-72.33%	36.02 (20.34,60.52)	14.83 (7.58,25.81)	-2.95 (-3.08,-2.78)
Tonga	male	18.71 (10.55,30.89)	9.54 (4.89,16.73)	-48.98%	26.92 (15.32,44.00)	15.05 (7.94,26.03)	-1.55 (-1.71,-1.32)
Trinidad and Tobago	male	55.76 (31.09,97.29)	28.25 (17.40,43.75)	-49.34%	8.12 (4.61,13.98)	5.00 (3.01,7.77)	-2.02 (-2.24,-1.86)
Tunisia	male	576.51 (328.17,921.76)	253.78 (153.16,387.51)	-55.98%	10.73 (6.29,17.00)	4.77 (2.86,7.29)	-2.72 (-3.01,-2.45)
Turkey	male	5607.35 (3054.30,9830.02)	457.59 (170.49,1029.35)	-91.84%	14.46 (7.86,25.31)	1.72 (0.65,3.89)	-7.58 (-7.83,-7.40)
Turkmenistan	male	319.29 (191.81,498.25)	214.21 (128.52,328.03)	-32.91%	12.71 (7.78,19.82)	7.84 (4.72,11.97)	-1.74 (-1.87,-1.61)
Tuvalu	male	2.78 (1.67,4.46)	1.39 (0.75,2.32)	-50.15%	44.15 (26.55,70.86)	22.88 (12.54,38.64)	-1.79 (-1.94,-1.58)
Uganda	male	7655.60 (4666.45,11703.95)	6075.59 (3494.74,9777.16)	-20.64%	50.26 (29.87,76.03)	18.77 (10.76,29.67)	-3.55 (-3.73,-3.39)
Ukraine	male	132.87 (46.86,293.51)	29.75 (8.43,72.56)	-77.61%	0.68 (0.24,1.47)	0.25 (0.07,0.59)	-3.76 (-4.00,-3.58)
United Arab Emirates	male	25.91 (10.46,53.23)	8.86 (2.65,20.33)	-65.82%	2.30 (0.94,4.75)	0.44 (0.14,1.02)	-5.02 (-5.66,-4.40)
United Kingdom	male	177.14 (67.99,383.96)	58.71 (18.26,151.44)	-66.86%	0.90 (0.35,1.96)	0.28 (0.09,0.72)	-3.60 (-4.08,-3.02)
United Republic of Tanzania	male	13709.91 (8471.96,20007.41)	11020.27 (6455.50,17225.38)	-19.62%	65.03 (40.11,95.06)	27.50 (16.56,42.67)	-2.80 (-2.91,-2.67)

United States Virgin Islands	male	1.45 (0.60,3.06)	0.23 (0.08,0.48)	-83.98%	2.60 (1.09,5.55)	0.66 (0.24,1.39)	-4.97 (-5.51,-4.53)
United States of America	male	182.82 (79.30,371.41)	101.71 (37.74,216.73)	-44.36%	0.18 (0.08,0.37)	0.10 (0.04,0.21)	-2.47 (-2.63,-2.35)
Uruguay	male	177.31 (77.13,343.52)	57.14 (20.76,121.27)	-67.78%	12.53 (5.43,23.92)	4.72 (1.74,9.94)	-3.35 (-3.47,-3.20)
Uzbekistan	male	3101.85 (1669.05,5178.73)	1594.28 (959.51,2451.64)	-48.60%	20.54 (11.42,33.63)	8.83 (5.33,13.64)	-2.94 (-3.00,-2.88)
Vanuatu	male	59.05 (34.90,91.08)	69.45 (39.66,112.25)	17.61%	46.76 (27.79,71.74)	35.05 (20.15,56.21)	-0.77 (-0.88,-0.59)
Venezuela (Bolivarian Republic of)	male	1098.36 (615.70,1891.36)	672.73 (376.13,1118.24)	-38.75%	9.25 (5.22,15.59)	5.24 (2.87,8.83)	-1.63 (-1.89,-1.33)
Viet Nam	male	6868.02 (3918.55,11058.87)	1309.23 (704.07,2301.75)	-80.94%	14.77 (8.49,23.60)	3.29 (1.72,5.89)	-4.49 (-4.76,-4.08)
Yemen	male	10222.29 (6753.40,14974.91)	9344.93 (5499.93,14632.64)	-8.58%	75.35 (49.69,110.65)	42.26 (25.37,65.58)	-2.50 (-2.84,-2.27)
Zambia	male	5766.89 (3704.75,8456.45)	4425.14 (2631.65,6897.81)	-23.27%	90.25 (58.13,131.99)	35.77 (21.94,54.81)	-3.41 (-3.91,-2.94)
Zimbabwe	male	3633.76 (2199.45,5553.39)	3224.43 (1939.76,5081.53)	-11.26%	45.12 (27.60,68.32)	32.86 (20.17,51.24)	-0.49 (-0.76,-0.13)

Afghanistan	female	3507.88 (2167.23,5164.50)	8196.85 (4977.02,12327.76)	133.67%	40.19 (24.68,59.24)	27.80 (17.09,41.80)	-1.14 (-1.57,-0.64)
Albania	female	417.36 (233.39,673.55)	67.07 (34.16,114.56)	-83.93%	22.14 (12.35,35.73)	8.55 (4.34,14.55)	-3.84 (-4.25,-3.56)
Algeria	female	2516.45 (1482.57,3942.77)	1455.71 (899.56,2244.74)	-42.15%	14.78 (8.84,22.96)	7.08 (4.42,10.93)	-2.31 (-2.45,-2.16)
American Samoa	female	2.23 (1.17,3.97)	0.93 (0.43,1.69)	-58.36%	6.49 (3.39,11.57)	3.55 (1.65,6.42)	-1.73 (-1.90,-1.47)
Andorra	female	0.05 (0.02,0.12)	0.02 (0.01,0.06)	-56.70%	0.38 (0.11,0.82)	0.15 (0.04,0.36)	-2.80 (-3.33,-2.21)
Angola	female	3996.69 (2576.75,5821.74)	5638.12 (3512.49,8654.72)	41.07%	50.81 (33.14,73.41)	25.56 (16.00,38.61)	-2.16 (-2.46,-1.75)
Antigua and Barbuda	female	2.73 (1.61,4.28)	2.01 (1.21,3.09)	-26.54%	8.40 (4.93,13.19)	5.77 (3.39,9.10)	-1.37 (-1.50,-1.27)
Argentina	female	1894.97 (1051.07,3114.95)	822.81 (393.86,1514.44)	-56.58%	10.97 (6.09,17.98)	4.67 (2.23,8.45)	-2.95 (-3.15,-2.74)
Armenia	female	10.89 (4.84,19.82)	3.75 (1.63,7.02)	-65.60%	0.61 (0.27,1.11)	0.38 (0.17,0.71)	-1.20 (-1.62,-0.62)
Australia	female	1.13 (0.44,2.40)	0.67 (0.27,1.40)	-40.90%	0.02 (0.01,0.04)	0.01 (0.00,0.02)	-1.56 (-1.87,-1.10)
Austria	female	15.25 (5.36,32.34)	4.54 (1.17,11.48)	-70.20%	0.68 (0.24,1.40)	0.21 (0.05,0.53)	-3.45 (-3.92,-2.86)
Azerbaijan	female	133.73 (69.37,229.94)	61.42 (28.76,110.47)	-54.07%	3.18 (1.65,5.47)	1.64 (0.78,2.93)	-2.31 (-2.97,-1.50)
Bahamas	female	8.68 (5.21,13.35)	8.58 (5.12,13.05)	-1.14%	6.27 (3.80,9.60)	5.31 (3.18,8.12)	-0.72 (-0.87,-0.58)
Bahrain	female	13.27 (7.15,22.28)	5.11 (2.26,9.48)	-61.48%	4.66 (2.51,7.73)	1.32 (0.59,2.44)	-4.06 (-4.17,-3.94)

Bangladesh	female	24117.71 (15140.26,36322.81)	7747.54 (4557.31,12225.98)	-67.88%	29.47 (18.67,44.32)	10.39 (6.11,16.34)	-3.22 (-3.44,-2.91)
Barbados	female	6.19 (3.61,9.52)	4.29 (2.59,6.52)	-30.68%	5.41 (3.14,8.45)	4.23 (2.53,6.49)	-0.83 (-0.95,-0.73)
Belarus	female	43.13 (18.02,84.62)	8.66 (3.03,18.29)	-79.92%	1.06 (0.45,2.06)	0.31 (0.11,0.66)	-3.95 (-4.22,-3.54)
Belgium	female	19.81 (7.16,41.27)	5.98 (1.78,14.46)	-69.78%	0.66 (0.24,1.39)	0.19 (0.06,0.46)	-3.85 (-4.25,-3.35)
Belize	female	21.01 (11.86,33.47)	17.35 (10.11,26.75)	-17.42%	15.63 (9.04,24.58)	8.26 (4.89,12.80)	-2.06 (-2.25,-1.89)
Benin	female	2768.89 (1789.87,4098.31)	4172.52 (2570.20,6251.66)	50.69%	65.98 (42.25,98.10)	42.15 (26.09,62.62)	-1.29 (-1.64,-0.88)
Bermuda	female	0.39 (0.18,0.68)	0.09 (0.03,0.20)	-76.51%	1.85 (0.87,3.26)	0.62 (0.24,1.33)	-3.99 (-4.22,-3.81)
Bhutan	female	222.68 (137.85,332.98)	54.76 (32.38,84.61)	-75.41%	51.31 (31.91,76.31)	16.35 (9.68,25.30)	-4.10 (-4.30,-3.95)
Bolivia (Plurinational State of)	female	795.85 (492.88,1177.99)	818.74 (496.76,1255.98)	2.87%	17.48 (10.91,26.04)	12.03 (7.34,18.44)	-1.02 (-1.19,-0.81)
Bosnia and Herzegovina	female	286.82 (148.83,479.03)	40.83 (19.53,74.78)	-85.77%	15.49 (8.06,25.78)	5.23 (2.56,9.50)	-4.54 (-5.04,-4.12)
Botswana	female	377.58 (233.23,571.92)	223.53 (133.24,357.19)	-40.80%	39.31 (24.35,59.04)	18.47 (11.07,29.59)	-2.41 (-2.52,-2.26)
Brazil	female	19860.00 (12311.51,30007.36)	8978.22 (5452.52,13534.18)	-54.79%	22.46 (13.97,33.91)	10.23 (6.16,15.64)	-2.63 (-2.70,-2.57)

Brunei Darussalam	female	3.98 (2.07,6.89)	1.48 (0.66,2.74)	-62.88%	2.54 (1.31,4.41)	0.94 (0.43,1.73)	-3.18 (-3.44,-2.87)
Bulgaria	female	247.53 (123.84,426.11)	79.24 (37.64,147.00)	-67.99%	8.69 (4.34,14.93)	4.84 (2.36,8.91)	-1.85 (-2.04,-1.66)
Burkina Faso	female	7218.99 (4536.40,10720.81)	10073.01 (6270.55,14977.49)	39.53%	87.14 (54.51,129.09)	56.36 (35.27,83.80)	-1.61 (-1.84,-1.44)
Burundi	female	1977.55 (1203.16,3048.56)	2628.40 (1572.46,4145.57)	32.91%	42.15 (25.62,64.05)	27.76 (16.73,43.37)	-1.81 (-2.13,-1.61)
Cape Verde	female	83.41 (49.85,129.75)	30.39 (18.75,48.33)	-63.56%	31.21 (18.80,48.28)	11.06 (6.78,17.64)	-3.48 (-3.56,-3.41)
Cambodia	female	4792.00 (3031.36,7243.46)	1600.20 (935.69,2568.31)	-66.61%	56.10 (35.26,84.23)	18.57 (10.87,29.73)	-3.63 (-3.70,-3.52)
Cameroon	female	6029.20 (3873.42,8691.20)	5404.25 (3243.66,8456.45)	-10.37%	72.69 (47.00,104.41)	26.74 (16.17,41.65)	-3.34 (-3.48,-3.18)
Canada	female	56.71 (20.82,116.34)	19.75 (5.92,46.75)	-65.17%	0.58 (0.21,1.19)	0.20 (0.06,0.46)	-3.51 (-4.05,-2.93)
Central African Republic	female	1608.67 (997.26,2384.46)	2101.73 (1342.08,3248.79)	30.65%	72.79 (45.80,108.40)	54.38 (34.70,82.85)	-1.06 (-1.33,-0.82)
Chad	female	4430.99 (2858.26,6587.25)	7017.84 (4394.95,10648.06)	58.38%	85.18 (54.82,126.02)	49.45 (31.32,74.26)	-1.85 (-2.05,-1.59)
Chile	female	120.66 (53.77,247.02)	20.85 (5.76,50.73)	-82.72%	1.75 (0.77,3.61)	0.34 (0.10,0.83)	-5.15 (-5.44,-4.75)

China	female	43147.46 (27248.84,63612.91)	15298.76 (9803.59,23047.88)	-64.54%	7.81 (4.92,11.48)	2.87 (1.79,4.32)	-3.83 (-4.12,-3.61)
Colombia	female	1618.35 (975.61,2585.43)	1019.28 (611.91,1575.36)	-37.02%	8.37 (5.07,13.22)	4.66 (2.78,7.25)	-2.18 (-2.47,-1.92)
Comoros	female	176.08 (108.21,269.18)	85.66 (50.62,132.18)	-51.35%	48.48 (30.30,74.62)	21.92 (13.05,33.62)	-2.85 (-3.02,-2.71)
Congo	female	1295.65 (816.55,1920.23)	1454.54 (875.20,2183.92)	12.26%	70.43 (44.57,105.40)	43.78 (26.48,65.84)	-1.47 (-1.92,-0.97)
Cook Islands	female	1.17 (0.66,1.89)	0.44 (0.25,0.68)	-62.87%	10.95 (6.18,17.54)	5.60 (3.16,8.87)	-2.00 (-2.10,-1.86)
Costa Rica	female	130.26 (77.74,201.30)	92.07 (54.44,144.53)	-29.32%	7.12 (4.30,10.88)	4.38 (2.54,6.83)	-1.79 (-2.03,-1.57)
Cote d'Ivoire	female	7120.08 (4669.81,10226.28)	6622.96 (3908.70,10354.92)	-6.98%	71.18 (46.49,102.87)	36.92 (21.97,56.99)	-2.13 (-2.31,-1.89)
Croatia	female	81.99 (37.77,153.53)	22.51 (9.54,44.03)	-72.55%	5.12 (2.41,9.44)	2.32 (1.00,4.52)	-3.09 (-3.29,-2.95)
Cuba	female	120.18 (57.03,215.86)	39.79 (16.60,74.60)	-66.89%	2.82 (1.33,5.09)	1.33 (0.57,2.42)	-2.43 (-2.53,-2.32)
Cyprus	female	5.06 (2.04,10.09)	0.90 (0.24,2.19)	-82.30%	1.55 (0.62,3.13)	0.25 (0.07,0.61)	-6.10 (-6.74,-5.49)
Czechia	female	183.20 (86.54,337.84)	60.95 (26.22,119.25)	-66.73%	5.16 (2.50,9.51)	2.14 (0.92,4.11)	-2.50 (-2.96,-2.04)
Democratic People's Republic of Korea	female	2028.17 (1152.28,3327.98)	665.96 (362.74,1110.95)	-67.16%	16.77 (9.64,27.35)	7.78 (4.09,13.04)	-2.91 (-3.57,-2.38)

Democratic Republic of the Congo	female	19717.38 (12462.44,28926.89)	30057.05 (18738.04,44979.83)	52.44%	61.86 (39.16,91.00)	47.21 (29.89,70.32)	-0.42 (-0.94,0.29)
Denmark	female	8.85 (3.16,19.09)	2.99 (0.82,7.30)	-66.21%	0.61 (0.22,1.33)	0.19 (0.05,0.48)	-3.76 (-4.18,-3.28)
Djibouti	female	168.29 (101.76,248.92)	145.11 (84.46,225.33)	-13.77%	46.53 (28.44,69.28)	21.21 (12.33,32.72)	-2.80 (-3.03,-2.63)
Dominica	female	3.13 (1.90,5.01)	1.54 (0.94,2.39)	-50.61%	7.77 (4.78,12.37)	5.53 (3.30,8.60)	-1.24 (-1.49,-1.03)
Dominican Republic	female	697.43 (411.56,1076.34)	377.53 (219.11,580.37)	-45.87%	14.90 (8.85,22.87)	7.05 (4.12,10.85)	-2.85 (-3.01,-2.74)
Ecuador	female	813.05 (478.11,1262.47)	489.48 (295.47,767.77)	-39.80%	12.56 (7.41,19.38)	5.56 (3.38,8.73)	-3.19 (-3.41,-3.03)
Egypt	female	3886.77 (2344.43,5827.96)	2147.14 (1185.50,3517.11)	-44.76%	10.50 (6.37,15.79)	4.04 (2.27,6.54)	-2.40 (-2.68,-1.97)
El Salvador	female	587.06 (345.00,930.24)	236.73 (144.11,368.32)	-59.68%	16.87 (10.02,26.57)	7.74 (4.69,12.01)	-2.59 (-2.92,-2.25)
Equatorial Guinea	female	391.83 (268.07,558.45)	136.55 (84.64,206.58)	-65.15%	117.25 (79.70,166.00)	18.55 (11.67,27.36)	-7.31 (-7.87,-6.93)
Eritrea	female	1559.34 (999.52,2318.16)	1204.19 (738.32,1904.80)	-22.78%	65.03 (41.93,96.96)	27.52 (17.04,43.04)	-2.84 (-2.88,-2.78)
Estonia	female	3.68 (1.45,7.51)	0.57 (0.19,1.19)	-84.63%	0.62 (0.25,1.26)	0.16 (0.05,0.34)	-4.59 (-4.79,-4.33)
Eswatini	female	209.94 (127.04,329.92)	98.46 (60.11,148.16)	-53.10%	32.43 (19.88,50.09)	14.97 (9.31,22.33)	-2.48 (-2.58,-2.32)
Ethiopia	female	31753.40 (21496.76,45076.38)	29234.65 (18995.62,43346.04)	-7.93%	74.01 (50.28,105.13)	37.89 (24.65,56.06)	-2.34 (-2.64,-1.99)

Fiji	female	70.88 (41.38,113.99)	34.89 (21.18,54.56)	-50.78%	15.36 (9.01,24.40)	7.73 (4.72,12.07)	-2.18 (-2.29,-2.03)
Finland	female	10.47 (3.63,21.50)	2.62 (0.74,6.51)	-75.00%	0.65 (0.23,1.34)	0.19 (0.06,0.46)	-3.98 (-4.38,-3.53)
France	female	21.16 (6.71,49.21)	9.46 (3.37,21.43)	-55.30%	0.11 (0.03,0.25)	0.05 (0.02,0.12)	-1.92 (-2.20,-1.56)
Gabon	female	252.61 (154.03,389.73)	136.11 (85.62,202.73)	-46.12%	36.46 (22.94,55.89)	14.24 (9.00,21.01)	-3.03 (-3.11,-2.92)
Gambia	female	725.18 (481.29,1062.86)	692.12 (442.63,1047.94)	-4.56%	86.60 (57.19,126.31)	43.84 (28.17,66.12)	-2.33 (-2.41,-2.23)
Georgia	female	42.46 (19.84,76.01)	22.19 (10.63,42.23)	-47.75%	1.82 (0.87,3.26)	1.82 (0.89,3.45)	0.71 (0.16,1.47)
Germany	female	39.13 (13.74,83.21)	17.73 (6.65,37.71)	-54.69%	0.18 (0.06,0.38)	0.09 (0.03,0.19)	-1.98 (-2.15,-1.76)
Ghana	female	7395.71 (4702.72,10934.29)	5119.74 (3008.72,7855.73)	-30.77%	61.77 (39.39,91.82)	26.93 (15.89,41.08)	-2.92 (-3.34,-2.48)
Greece	female	31.17 (11.15,66.88)	8.43 (2.75,19.98)	-72.97%	1.02 (0.36,2.14)	0.36 (0.12,0.85)	-3.13 (-3.81,-2.39)
Greenland	female	0.35 (0.15,0.67)	0.13 (0.04,0.25)	-63.98%	1.40 (0.61,2.64)	0.64 (0.22,1.27)	-2.83 (-2.98,-2.68)
Grenada	female	8.14 (4.80,12.25)	2.97 (1.83,4.64)	-63.50%	15.22 (9.05,22.77)	7.21 (4.37,11.17)	-2.19 (-2.59,-1.75)
Guam	female	3.77 (1.92,6.70)	2.06 (0.94,3.65)	-45.34%	5.06 (2.57,8.93)	2.67 (1.22,4.71)	-2.07 (-2.32,-1.76)
Guatemala	female	1234.23 (741.37,1839.39)	998.50 (592.64,1555.30)	-19.10%	20.12 (12.21,29.81)	10.15 (6.06,15.76)	-2.09 (-2.40,-1.77)
Guinea	female	3272.94 (2099.06,4729.31)	3845.95 (2327.92,5926.91)	17.51%	64.73 (41.37,93.55)	39.47 (24.03,60.38)	-1.53 (-1.71,-1.32)

Guinea-Bissau	female	634.33 (398.75,947.38)	578.14 (355.62,889.01)	-8.86%	76.15 (48.03,114.15)	42.84 (26.57,65.56)	-1.79 (-1.89,-1.64)
Guyana	female	86.24 (49.53,138.55)	33.15 (19.35,51.59)	-61.56%	17.67 (10.19,28.44)	8.94 (5.22,13.69)	-2.21 (-2.32,-2.05)
Haiti	female	1710.02 (1041.41,2534.35)	1832.49 (1150.99,2808.20)	7.16%	36.08 (22.04,53.62)	24.83 (15.63,37.94)	-1.14 (-1.21,-1.05)
Honduras	female	518.55 (309.85,787.45)	403.99 (246.71,628.72)	-22.09%	14.36 (8.66,21.56)	7.35 (4.51,11.42)	-2.23 (-2.40,-2.08)
Hungary	female	234.15 (114.94,416.45)	67.99 (30.09,129.50)	-70.96%	6.83 (3.46,12.25)	3.00 (1.34,5.64)	-2.70 (-2.99,-2.47)
Iceland	female	0.52 (0.17,1.13)	0.17 (0.05,0.40)	-67.30%	0.48 (0.16,1.07)	0.15 (0.04,0.37)	-3.65 (-4.16,-3.08)
India	female	271028.63 (179802.52,385368.03)	135244.56 (86457.52,201168.28)	-50.10%	50.03 (33.58,70.91)	21.55 (13.78,32.11)	-2.97 (-3.33,-2.55)
Indonesia	female	51730.98 (33664.97,75602.36)	21610.81 (13801.36,31799.28)	-58.22%	47.02 (30.94,68.15)	18.09 (11.66,26.40)	-3.34 (-3.49,-3.22)
Iran (Islamic Republic of)	female	3524.79 (1967.42,5596.89)	234.56 (110.07,428.45)	-93.35%	7.82 (4.38,12.41)	0.68 (0.32,1.23)	-8.09 (-8.55,-7.70)
Iraq	female	2052.89 (1243.73,3226.88)	1540.89 (900.76,2330.18)	-24.94%	15.61 (9.62,24.23)	6.90 (4.07,10.34)	-2.94 (-3.09,-2.80)
Ireland	female	8.95 (3.10,19.80)	2.05 (0.53,5.30)	-77.11%	0.60 (0.21,1.31)	0.13 (0.03,0.32)	-4.97 (-5.43,-4.44)
Israel	female	134.91 (58.39,255.09)	82.04 (30.04,175.18)	-39.18%	5.22 (2.28,9.83)	1.82 (0.66,3.87)	-3.34 (-3.64,-2.96)
Italy	female	142.95 (57.60,287.83)	40.45 (13.71,95.84)	-71.70%	0.99 (0.39,1.97)	0.32 (0.11,0.75)	-3.35 (-3.92,-2.70)

Jamaica	female	134.38 (78.37,210.60)	71.84 (44.25,111.01)	-46.54%	9.63 (5.65,15.00)	6.10 (3.73,9.41)	-1.56 (-1.76,-1.38)
Japan	female	339.60 (160.38,658.41)	116.30 (47.78,233.79)	-65.75%	0.90 (0.44,1.76)	0.45 (0.19,0.90)	-2.06 (-2.20,-1.86)
Jordan	female	387.39 (230.15,597.38)	404.19 (235.41,632.42)	4.34%	14.70 (8.86,22.35)	6.81 (4.03,10.55)	-2.69 (-2.92,-2.49)
Kazakhstan	female	1039.00 (605.09,1696.65)	445.79 (228.22,745.31)	-57.09%	11.52 (6.75,18.81)	5.03 (2.59,8.38)	-2.69 (-2.95,-2.30)
Kenya	female	11159.65 (7202.71,16079.99)	9933.98 (6175.59,14874.40)	-10.98%	57.31 (36.97,82.44)	31.26 (19.52,46.57)	-2.15 (-2.45,-1.86)
Kiribati	female	17.94 (10.88,27.23)	20.94 (12.49,32.17)	16.71%	35.29 (21.64,53.17)	29.68 (17.77,45.59)	-0.12 (-0.31,0.14)
Kuwait	female	59.73 (35.47,92.23)	92.62 (52.62,144.37)	55.07%	6.74 (4.06,10.35)	5.00 (2.91,7.82)	-1.05 (-1.08,-1.00)
Kyrgyzstan	female	272.52 (163.84,403.48)	325.09 (199.34,492.91)	19.29%	10.05 (6.03,14.82)	9.24 (5.66,13.99)	0.09 (-0.12,0.37)
Lao People's Democratic Republic	female	1293.15 (806.23,1947.62)	566.88 (320.44,898.97)	-56.16%	40.40 (25.13,60.52)	14.67 (8.30,23.22)	-3.42 (-3.70,-3.09)
Latvia	female	6.00 (2.34,12.14)	1.08 (0.40,2.23)	-81.92%	0.61 (0.24,1.23)	0.21 (0.08,0.44)	-3.40 (-3.50,-3.24)
Lebanon	female	130.76 (69.86,223.92)	26.06 (10.98,52.01)	-80.07%	6.09 (3.25,10.49)	1.09 (0.46,2.16)	-5.70 (-5.93,-5.44)
Lesotho	female	610.46 (383.83,921.37)	271.43 (172.29,401.76)	-55.54%	47.75 (30.56,72.08)	24.34 (15.36,35.78)	-2.13 (-2.26,-1.93)
Liberia	female	554.95 (347.68,837.88)	523.76 (306.15,809.88)	-5.62%	36.13 (22.78,54.14)	17.43 (10.34,26.72)	-3.00 (-3.63,-2.44)
Libya	female	363.68 (218.97,577.33)	194.69 (116.71,293.48)	-46.47%	12.20 (7.40,19.18)	6.77 (4.12,10.29)	-1.71 (-2.07,-1.37)

Lithuania	female	8.24 (3.25,16.71)	1.40 (0.50,2.89)	-83.00%	0.58 (0.23,1.17)	0.20 (0.07,0.41)	-3.42 (-3.59,-3.15)
Luxembourg	female	0.54 (0.19,1.13)	0.24 (0.07,0.54)	-56.24%	0.48 (0.17,1.00)	0.15 (0.04,0.33)	-3.82 (-4.36,-3.22)
Madagascar	female	4726.60 (3075.11,7075.48)	5205.20 (3126.13,8065.81)	10.13%	48.97 (31.65,72.80)	28.30 (17.47,43.99)	-1.92 (-2.24,-1.57)
Malawi	female	5959.07 (3899.28,8709.88)	5750.79 (3542.24,8817.05)	-3.50%	76.92 (50.14,113.03)	43.07 (26.69,65.35)	-2.16 (-2.43,-1.90)
Malaysia	female	361.12 (200.10,590.34)	68.96 (30.40,128.65)	-80.90%	3.16 (1.76,5.13)	0.54 (0.24,1.01)	-5.32 (-5.82,-4.67)
Maldives	female	96.74 (59.23,140.68)	13.97 (7.71,23.89)	-85.56%	51.67 (31.87,75.04)	7.26 (4.01,12.24)	-6.75 (-7.29,-6.25)
Mali	female	6458.34 (4215.71,9145.86)	10822.88 (6699.32,16053.94)	67.58%	90.53 (59.23,128.32)	60.62 (37.62,89.52)	-1.43 (-1.54,-1.34)
Malta	female	2.01 (0.82,4.09)	0.35 (0.11,0.78)	-82.47%	1.40 (0.57,2.86)	0.33 (0.10,0.74)	-4.37 (-4.77,-3.88)
Marshall Islands	female	18.40 (11.34,27.56)	7.10 (4.29,10.98)	-61.42%	49.18 (30.44,73.16)	23.25 (14.07,35.99)	-2.48 (-2.54,-2.43)
Mauritania	female	779.45 (482.93,1134.27)	578.48 (336.19,891.09)	-25.78%	48.48 (30.39,70.88)	22.02 (12.90,33.89)	-2.52 (-2.63,-2.42)
Mauritius	female	68.76 (39.45,110.38)	19.03 (11.63,30.30)	-72.32%	12.54 (7.20,19.96)	4.53 (2.68,7.45)	-3.04 (-3.33,-2.67)
Mexico	female	8769.98 (5602.78,12556.40)	5601.50 (3611.12,8222.87)	-36.13%	16.03 (10.30,22.94)	9.52 (6.15,13.87)	-1.80 (-1.91,-1.69)

Micronesia (Federated States of)	female	38.67 (24.87,56.91)	14.44 (8.18,22.64)	-62.66%	49.30 (31.75,72.42)	28.54 (16.21,44.85)	-1.64 (-1.75,-1.51)
Monaco	female	0.02 (0.01,0.04)	0.01 (0.00,0.02)	-45.97%	0.28 (0.09,0.68)	0.11 (0.04,0.26)	-3.00 (-3.41,-2.51)
Mongolia	female	135.84 (82.72,203.86)	98.50 (59.73,151.27)	-27.48%	9.90 (6.19,14.96)	5.62 (3.42,8.66)	-2.08 (-2.20,-1.98)
Montenegro	female	13.45 (6.28,24.08)	5.61 (2.65,10.53)	-58.29%	4.99 (2.37,8.92)	3.18 (1.49,5.92)	-2.18 (-2.78,-1.72)
Morocco	female	3919.76 (2332.33,6165.92)	1529.20 (891.39,2314.42)	-60.99%	23.56 (14.22,36.88)	9.27 (5.39,14.17)	-3.05 (-3.17,-2.89)
Mozambique	female	8924.68 (5649.96,13326.93)	9735.06 (6041.98,14909.31)	9.08%	82.49 (52.16,123.05)	39.99 (24.88,61.34)	-2.60 (-2.81,-2.40)
Myanmar	female	13334.09 (8322.60,20480.66)	3407.29 (1931.11,5705.56)	-74.45%	48.14 (29.95,74.09)	13.46 (7.63,22.59)	-4.43 (-4.65,-4.21)
Namibia	female	245.53 (148.92,373.03)	188.07 (116.85,290.33)	-23.40%	24.86 (15.38,37.51)	13.44 (8.38,20.59)	-2.26 (-2.65,-1.87)
Nauru	female	1.33 (0.72,2.11)	0.71 (0.41,1.19)	-46.56%	17.15 (9.53,26.81)	10.98 (6.36,18.19)	-0.99 (-1.57,-0.18)
Nepal	female	5382.69 (3195.86,8162.87)	2034.03 (1202.55,3202.69)	-62.21%	36.09 (21.49,54.93)	13.19 (7.87,20.84)	-3.71 (-3.99,-3.50)
Netherlands	female	21.76 (7.64,48.78)	7.43 (2.23,18.02)	-65.85%	0.47 (0.16,1.06)	0.17 (0.05,0.41)	-3.26 (-3.62,-2.83)
New Zealand	female	5.69 (2.30,12.16)	3.50 (1.29,7.32)	-38.47%	0.42 (0.17,0.90)	0.24 (0.09,0.50)	-1.06 (-1.39,-0.60)

Nicaragua	female	232.34 (139.71,357.13)	145.79 (82.68,229.86)	-37.25%	8.24 (5.03,12.40)	4.29 (2.46,6.69)	-2.54 (-2.94,-2.22)
Niger	female	7442.06 (4690.92,10982.85)	16269.42 (10187.22,23722.67)	118.61%	104.57 (65.88,154.44)	77.67 (48.49,113.36)	-0.88 (-0.97,-0.75)
Nigeria	female	25444.58 (16944.13,37440.82)	26939.94 (17288.13,39996.43)	5.88%	35.58 (23.68,51.73)	17.62 (11.43,25.91)	-2.56 (-3.01,-2.07)
Niue	female	0.19 (0.10,0.30)	0.05 (0.03,0.07)	-75.60%	13.58 (7.57,22.11)	6.51 (3.67,10.73)	-2.48 (-2.56,-2.39)
North Macedonia	female	125.54 (72.76,207.12)	32.43 (16.52,55.45)	-74.16%	14.00 (8.17,22.87)	5.66 (2.90,9.66)	-3.36 (-3.53,-3.20)
Northern Mariana Islands	female	1.03 (0.51,1.77)	0.39 (0.18,0.73)	-61.90%	4.51 (2.20,7.84)	3.34 (1.54,6.24)	-0.11 (-0.47,0.42)
Norway	female	5.75 (2.28,11.35)	1.87 (0.58,4.38)	-67.56%	0.43 (0.17,0.85)	0.12 (0.04,0.30)	-3.87 (-4.42,-3.25)
Oman	female	392.47 (240.93,601.14)	211.49 (125.96,323.33)	-46.11%	28.28 (17.59,42.92)	11.85 (7.16,17.82)	-2.61 (-3.06,-2.13)
Pakistan	female	27477.10 (17799.61,41112.49)	17813.22 (11373.82,26042.22)	-35.17%	33.52 (21.81,50.10)	13.74 (8.85,20.02)	-3.06 (-3.32,-2.88)
Palau	female	0.90 (0.52,1.41)	0.39 (0.23,0.60)	-57.02%	11.26 (6.58,17.69)	6.41 (3.68,10.14)	-1.59 (-1.72,-1.41)
Palestine	female	499.57 (306.15,750.62)	270.12 (166.29,419.18)	-45.93%	30.23 (18.48,45.53)	9.27 (5.71,14.23)	-3.64 (-3.90,-3.29)
Panama	female	99.73 (58.37,155.50)	112.33 (68.58,175.51)	12.64%	7.40 (4.41,11.52)	5.60 (3.41,8.71)	-1.03 (-1.15,-0.89)

Papua New Guinea	female	628.10 (385.85,967.10)	987.63 (570.93,1566.54)	57.24%	22.20 (13.61,34.16)	15.84 (9.27,24.90)	-0.57 (-0.81,-0.23)
Paraguay	female	464.39 (285.97,731.40)	322.02 (196.40,502.45)	-30.66%	16.90 (10.42,26.46)	9.49 (5.77,14.73)	-1.88 (-1.95,-1.80)
Peru	female	2718.05 (1749.80,4065.29)	1420.35 (866.07,2186.54)	-47.74%	19.39 (12.52,28.93)	8.89 (5.41,13.75)	-2.95 (-3.18,-2.75)
Philippines	female	8815.29 (5380.05,13758.75)	7031.11 (4007.01,11188.30)	-20.24%	20.66 (12.73,32.18)	11.83 (6.78,18.71)	-1.55 (-1.77,-1.21)
Poland	female	1410.78 (724.55,2524.50)	301.66 (138.90,567.23)	-78.62%	8.86 (4.63,15.87)	3.07 (1.42,5.81)	-3.61 (-3.78,-3.46)
Portugal	female	72.32 (28.35,146.09)	10.09 (2.94,24.08)	-86.04%	2.25 (0.89,4.42)	0.46 (0.14,1.09)	-4.83 (-5.34,-4.22)
Puerto Rico	female	35.28 (16.32,64.53)	5.83 (2.19,11.93)	-83.46%	2.10 (0.98,3.94)	0.70 (0.29,1.43)	-4.11 (-4.32,-3.93)
Qatar	female	6.45 (3.26,11.19)	3.29 (1.26,6.50)	-48.97%	2.96 (1.47,5.09)	0.47 (0.18,0.91)	-6.08 (-6.31,-5.84)
Republic of Korea	female	411.69 (212.77,695.60)	53.74 (23.70,105.10)	-86.95%	2.23 (1.14,3.78)	0.48 (0.22,0.94)	-4.67 (-5.13,-4.12)
Republic of Moldova	female	46.97 (22.40,84.35)	8.01 (3.51,16.75)	-82.96%	2.23 (1.07,3.99)	0.84 (0.38,1.67)	-3.16 (-3.57,-2.58)
Romania	female	962.31 (518.58,1578.72)	218.75 (104.29,387.19)	-77.27%	10.52 (5.71,17.08)	4.49 (2.15,7.96)	-3.06 (-3.30,-2.86)
Russian Federation	female	53.09 (26.29,99.70)	15.83 (6.68,34.17)	-70.19%	0.09 (0.05,0.17)	0.03 (0.02,0.07)	-3.18 (-3.41,-2.87)
Rwanda	female	2183.01 (1381.33,3274.50)	1378.86 (802.56,2166.19)	-36.84%	36.45 (23.36,54.77)	17.02 (10.02,26.44)	-2.81 (-3.41,-2.22)
Saint Kitts and Nevis	female	1.95 (1.19,3.08)	1.30 (0.77,1.99)	-33.11%	8.29 (5.10,12.99)	5.46 (3.26,8.46)	-1.37 (-1.60,-1.15)
Saint Lucia	female	9.47 (5.66,15.21)	4.23 (2.57,6.38)	-55.32%	10.76 (6.48,17.12)	6.58 (3.96,10.02)	-1.51 (-1.76,-1.24)

Saint Vincent and the Grenadines	female	9.61 (5.59,15.09)	3.54 (2.10,5.41)	-63.18%	13.91 (8.17,21.78)	7.71 (4.55,11.91)	-2.00 (-2.26,-1.78)
Samoa	female	20.30 (11.56,32.24)	17.70 (9.39,29.14)	-12.85%	19.57 (11.45,30.73)	15.21 (8.24,24.73)	-0.49 (-0.65,-0.27)
San Marino	female	0.03 (0.01,0.07)	0.01 (0.00,0.03)	-54.00%	0.40 (0.13,0.91)	0.16 (0.04,0.36)	-2.88 (-3.48,-2.23)
Sao Tome and Principe	female	52.84 (30.11,83.64)	22.34 (12.74,35.79)	-57.72%	54.96 (32.01,86.65)	18.61 (10.70,29.43)	-3.76 (-3.91,-3.58)
Saudi Arabia	female	319.11 (159.43,571.06)	14.80 (4.96,32.36)	-95.36%	2.63 (1.30,4.71)	0.13 (0.04,0.29)	-8.56 (-9.44,-7.37)
Senegal	female	3869.67 (2414.94,5728.20)	2012.51 (1229.35,3157.25)	-47.99%	61.31 (38.30,90.10)	20.51 (12.75,31.85)	-3.40 (-3.61,-3.12)
Serbia	female	639.24 (342.41,1077.18)	168.81 (77.80,305.45)	-73.59%	17.20 (9.30,29.03)	7.24 (3.39,12.90)	-3.29 (-3.48,-3.16)
Seychelles	female	3.69 (2.05,6.05)	1.55 (0.93,2.46)	-57.90%	9.16 (5.10,14.99)	3.80 (2.24,6.07)	-2.58 (-2.95,-2.13)
Sierra Leone	female	2084.85 (1274.07,3168.84)	2694.16 (1699.47,4078.05)	29.23%	73.34 (45.76,110.98)	46.49 (29.40,70.21)	-1.42 (-1.63,-1.18)
Singapore	female	18.08 (8.25,33.10)	4.99 (1.81,10.46)	-72.38%	1.67 (0.78,3.06)	0.36 (0.13,0.75)	-5.14 (-5.40,-4.87)
Slovakia	female	152.52 (77.35,271.93)	39.35 (17.31,74.91)	-74.20%	6.97 (3.59,12.40)	2.75 (1.21,5.23)	-2.98 (-3.20,-2.78)
Slovenia	female	26.95 (12.32,50.35)	9.37 (3.84,18.10)	-65.25%	4.02 (1.83,7.43)	1.81 (0.76,3.46)	-2.92 (-3.12,-2.79)
Solomon Islands	female	120.76 (76.87,183.62)	132.99 (78.07,215.77)	10.13%	45.01 (28.56,68.58)	30.44 (17.80,49.36)	-0.97 (-1.17,-0.69)

Somalia	female	4913.63 (3176.52,7175.29)	12266.85 (7819.17,18317.97)	149.65%	87.00 (56.16,126.94)	75.56 (48.43,112.74)	-0.57 (-0.63,-0.53)
South Africa	female	4411.46 (2738.83,6531.00)	2504.65 (1575.26,3756.29)	-43.22%	19.82 (12.56,29.20)	9.24 (5.82,13.88)	-2.72 (-2.87,-2.56)
South Sudan	female	2442.95 (1526.08,3603.11)	2211.10 (1349.19,3345.54)	-9.49%	55.70 (34.95,81.11)	32.23 (19.71,48.30)	-1.95 (-2.05,-1.85)
Spain	female	79.92 (27.90,169.08)	16.91 (5.00,38.77)	-78.84%	0.68 (0.25,1.48)	0.16 (0.05,0.37)	-4.08 (-4.81,-3.23)
Sri Lanka	female	1553.50 (917.09,2414.74)	415.32 (230.79,684.59)	-73.27%	16.63 (9.84,25.87)	4.49 (2.48,7.43)	-4.92 (-5.19,-4.69)
Sudan	female	6617.74 (4092.93,9887.35)	4190.96 (2517.39,6468.42)	-36.67%	42.65 (26.39,63.12)	16.44 (9.91,25.30)	-2.98 (-3.26,-2.61)
Suriname	female	32.37 (19.56,48.73)	24.84 (15.42,37.67)	-23.27%	14.60 (8.85,21.70)	9.49 (5.90,14.52)	-1.65 (-1.74,-1.59)
Sweden	female	14.03 (4.84,30.44)	5.24 (1.64,12.04)	-62.65%	0.53 (0.18,1.13)	0.18 (0.05,0.41)	-3.08 (-3.61,-2.41)
Switzerland	female	5.72 (1.75,12.53)	2.62 (0.68,6.51)	-54.28%	0.29 (0.09,0.64)	0.12 (0.03,0.30)	-2.72 (-3.16,-2.22)
Syrian Arab Republic	female	1324.98 (750.01,2262.64)	199.70 (94.57,364.99)	-84.93%	12.42 (7.08,20.91)	3.19 (1.49,5.89)	-4.63 (-4.99,-4.33)
Taiwan (Province of China)	female	133.86 (61.45,252.11)	17.68 (6.67,38.10)	-86.79%	1.50 (0.71,2.80)	0.36 (0.14,0.77)	-4.67 (-5.00,-4.40)

Tajikistan	female	608.17 (358.56,927.03)	646.04 (401.41,976.40)	6.23%	15.78 (9.54,23.47)	11.97 (7.57,17.97)	-0.86 (-1.20,-0.49)
Thailand	female	5520.90 (3430.01,8135.14)	1215.59 (696.67,2029.31)	-77.98%	19.71 (12.28,29.36)	5.09 (2.81,8.50)	-4.24 (-4.70,-3.71)
Timor-Leste	female	329.17 (197.50,494.51)	140.86 (82.77,227.70)	-57.21%	53.26 (31.52,80.19)	16.81 (9.89,27.08)	-4.67 (-5.13,-4.34)
Togo	female	2098.68 (1257.33,3201.03)	1835.35 (1083.83,2834.96)	-12.55%	67.88 (41.12,102.78)	34.87 (20.83,53.67)	-1.95 (-2.10,-1.75)
Tokelau	female	0.31 (0.17,0.49)	0.08 (0.05,0.13)	-73.39%	24.19 (13.91,39.02)	10.24 (5.81,16.60)	-2.82 (-2.92,-2.67)
Tonga	female	9.80 (5.55,15.95)	4.95 (2.67,8.38)	-49.44%	15.35 (8.80,24.81)	8.52 (4.66,14.25)	-1.56 (-1.72,-1.33)
Trinidad and Tobago	female	53.74 (32.34,82.79)	29.40 (18.29,44.79)	-45.29%	7.92 (4.83,12.07)	5.46 (3.29,8.45)	-1.52 (-1.69,-1.40)
Tunisia	female	463.01 (283.43,700.19)	236.18 (140.66,372.95)	-48.99%	9.11 (5.61,13.62)	4.56 (2.72,7.21)	-2.18 (-2.38,-1.96)
Turkey	female	4611.38 (2736.73,7230.20)	602.65 (280.56,1103.13)	-86.93%	12.50 (7.41,19.61)	2.35 (1.10,4.28)	-5.79 (-6.00,-5.59)
Turkmenistan	female	258.36 (160.08,393.23)	199.52 (122.00,301.84)	-22.78%	10.62 (6.63,15.90)	7.78 (4.74,11.82)	-1.02 (-1.09,-0.91)
Tuvalu	female	1.68 (0.98,2.65)	0.83 (0.48,1.37)	-50.82%	29.54 (17.15,46.02)	14.95 (8.74,24.39)	-1.86 (-2.01,-1.63)
Uganda	female	6043.34 (3829.67,9274.31)	5908.12 (3644.72,9089.11)	-2.24%	40.48 (25.80,61.58)	18.75 (11.66,28.62)	-2.72 (-2.91,-2.52)
Ukraine	female	108.32 (43.27,222.54)	32.47 (11.64,67.47)	-70.02%	0.57 (0.23,1.16)	0.29 (0.11,0.60)	-2.39 (-2.51,-2.25)
United Arab Emirates	female	31.34 (15.71,57.24)	13.87 (5.87,26.19)	-55.74%	3.05 (1.53,5.55)	0.72 (0.31,1.33)	-4.49 (-5.01,-3.99)

United Kingdom	female	186.58 (94.98,324.06)	79.30 (36.45,154.47)	-57.50%	1.00 (0.51,1.73)	0.41 (0.19,0.78)	-2.63 (-2.99,-2.15)
United Republic of Tanzania	female	9541.31 (5957.93,13936.08)	10951.44 (6777.59,16610.37)	14.78%	46.04 (29.16,66.16)	26.84 (16.52,40.18)	-1.58 (-1.79,-1.32)
United States Virgin Islands	female	1.52 (0.72,2.74)	0.29 (0.13,0.55)	-80.89%	2.78 (1.32,5.04)	0.86 (0.37,1.63)	-4.29 (-4.77,-3.91)
United States of America	female	327.78 (156.73,629.26)	196.49 (82.41,399.65)	-40.05%	0.34 (0.16,0.66)	0.19 (0.08,0.39)	-1.66 (-2.03,-1.23)
Uruguay	female	110.10 (53.13,195.95)	41.71 (18.36,82.61)	-62.11%	8.02 (3.84,14.35)	3.61 (1.56,7.22)	-2.54 (-2.69,-2.33)
Uzbekistan	female	2066.31 (1240.85,3115.73)	1584.00 (988.65,2377.87)	-23.34%	14.46 (8.80,21.65)	9.10 (5.68,13.66)	-1.45 (-1.54,-1.29)
Vanuatu	female	35.69 (21.21,54.38)	38.60 (22.63,60.42)	8.15%	31.03 (18.61,47.10)	21.05 (12.41,32.72)	-1.09 (-1.19,-0.94)
Venezuela (Bolivarian Republic of)	female	887.34 (521.39,1378.95)	720.59 (440.34,1117.47)	-18.79%	7.77 (4.66,12.05)	5.61 (3.36,8.65)	-0.97 (-1.15,-0.75)
Viet Nam	female	6810.80 (4050.31,10933.60)	1468.14 (825.07,2379.91)	-78.44%	15.18 (9.09,24.28)	3.93 (2.23,6.44)	-4.38 (-4.65,-4.02)
Yemen	female	7403.09 (4721.61,10837.36)	6223.60 (3781.22,9617.96)	-15.93%	58.56 (37.25,84.96)	30.04 (18.49,46.12)	-2.73 (-3.15,-2.43)
Zambia	female	3911.76 (2567.15,5758.46)	3445.89 (2109.68,5330.31)	-11.91%	60.53 (39.93,88.77)	27.54 (17.00,42.18)	-2.85 (-3.34,-2.33)

Zimbabwe	female	2878.51 (1736.37,4364.30)	3079.09 (1865.07,4718.20)	6.97%	36.24 (22.14,54.29)	30.96 (18.97,47.05)	0.19 (-0.15,0.65)

Abbreviations: CI, confidence interval; DALY, disability adjusted life-years; EAPC, estimated annual percentage change; UI, uncertainty interval; VAD, vitamin A deficiency.

Supplemental Table S5: Age distribution of incidence rate (per 100,000 people) for VAD in different countries in 2019.

country	<5	5 to 9	10 to 14	15 to 19	20 to 24	25 to 29	30 to 34	35 to 39	40 to 44	45 to 49	50 to 54	55 to 59	60 to 64	65 to 69	70 to 74	75 to 79	80 to 84	85 to 89	90 to 94	95+	All Ages
Afghanistan	52066.12	26621.32	20437.21	19189.85	19633.36	19264.63	19394.36	19021.70	18782.03	18582.28	17911.65	17693.68	17220.07	17325.96	17117.18	17095.37	17268.09	17333.39	17488.76	17719.15	26062.49
Albania	16589.96	11515.30	11884.39	12188.03	12705.92	12516.87	12324.59	11829.11	11261.80	10531.70	9783.77	9315.61	9084.51	8675.26	8532.77	8392.80	8458.26	9015.22	9821.96	11316.01	11202.14
Algeria	4617.01	3121.03	2599.69	2451.09	2492.33	2418.78	2332.34	2286.75	2223.62	2225.70	2153.98	2137.30	2144.00	2144.07	2123.60	2128.79	2131.22	2152.85	2273.50	2295.76	2639.37
American Samoa	10199.44	9528.12	8984.45	7965.88	7028.91	6195.56	4978.60	3697.77	2501.81	1751.24	1279.80	1003.53	1009.57	950.93	926.76	903.96	899.35	889.15	904.55	891.99	5733.76
Andorra	1260.43	860.71	599.44	480.62	392.87	327.45	263.48	216.72	188.18	172.49	165.96	168.82	186.39	208.83	230.90	265.88	290.24	313.22	348.60	365.64	319.21
Angola	26385.10	13473.50	12506.86	12664.56	12128.07	11597.59	11443.55	10858.33	10399.94	9735.98	9373.20	8583.20	8289.60	7880.09	7600.19	7474.36	7257.09	7091.24	6699.39	6342.90	14271.05

	2244.	2011.	1997.	2043.	2042.	2053.	2062.	1975.	1888.	1799.	1705.	1604.	1472.	1348.	1267.	1162.	1119.	1092.	1072.	1114.	1866.5
Antigua and Barbuda	35	39	98	46	24	87	66	73	83	37	63	95	58	89	19	13	68	49	95	72	2
	11616	9549.	7910.	7438.	7356.	7422.	7237.	7141.	6931.	6843.	6564.	6345.	5984.	5769.	5575.	5469.	5170.	4859.	4771.	4822.	7476.1
Argentina	.86	27	45	79	80	39	52	98	50	02	10	91	46	85	59	87	91	09	99	57	3
	659.5	523.2	434.9	436.7	453.1	464.2	481.2	492.1	501.1	512.2	500.8	498.1	496.4	504.2	515.6	511.0	520.6	520.6	530.2	531.8	
Armenia	3	8	2	8	3	1	5	7	8	2	5	8	1	4	8	5	6	9	3	5	498.59
									105.7	110.0	110.8	116.9	127.8	141.3	156.2	165.5	181.2	193.2	212.0	224.6	
Australia	36.24	38.76	39.62	46.76	56.80	68.96	82.60	95.46	9	6	8	2	9	5	8	9	4	0	5	6	93.28
	1802.	1226.	864.9	690.9	560.0	458.2	380.7	312.0	271.3	247.3	241.5	238.3	261.8	297.4	327.7	374.3	411.3	441.6	483.3	502.0	
Austria	93	03	1	4	7	5	4	0	1	4	3	5	9	9	4	9	5	0	4	4	492.10
	3840.	2855.	2425.	2418.	2437.	2455.	2520.	2496.	2499.	2381.	2263.	2207.	2153.	2104.	2079.	2053.	1990.	1948.	1957.	1972.	2527.3
Azerbaijan	23	49	77	70	26	21	56	85	07	68	53	23	03	38	33	65	69	89	33	05	3
	1615.	1461.	1457.	1480.	1536.	1519.	1505.	1461.	1409.	1329.	1242.	1170.	1096.	1022.	947.8	900.8	846.8	808.3	825.0	832.4	1384.9
Bahamas	40	80	40	86	43	97	72	63	16	33	52	76	77	09	7	6	0	8	0	0	5
	2267.	1719.	1531.	1433.	1428.	1431.	1405.	1378.	1342.	1311.	1335.	1286.	1310.	1283.	1310.	1285.	1289.	1253.	1279.	1252.	1438.0
Bahrain	92	07	98	02	78	33	81	21	67	83	23	97	87	47	00	67	76	72	97	67	4
	8411.	6114.	3863.	4171.	4158.	4264.	4229.	4207.	4146.	4082.	4027.	4109.	3960.	4057.	4043.	3924.	3965.	3986.	4011.	3994.	4671.4
Bangladesh	02	30	96	98	78	74	34	84	03	78	16	99	88	33	86	25	28	57	79	36	5
	2395.	2160.	2102.	2143.	2170.	2178.	2167.	2109.	2020.	1907.	1835.	1717.	1575.	1452.	1369.	1273.	1198.	1165.	1151.	1169.	1918.5
Barbados	86	24	66	75	89	36	86	20	81	25	00	90	11	08	94	00	98	00	84	97	1

	1158.	1116.	1020.	1131.	1265.	1433.	1529.	1571.	1479.	1365.	1240.	1177.	1142.	1139.	1147.	1155.	1162.	1123.	1158.	1174.	1273.7
Belarus	46	59	90	98	16	38	38	37	01	36	46	61	23	98	67	99	16	82	39	61	6
	1611.	1109.	790.6	610.3	501.9	411.9	341.8	280.5	241.9	226.5	215.4	213.7	235.5	267.0	297.6	330.8	373.5	408.9	437.9	450.7	
Belgium	99	87	2	9	6	8	0	0	7	2	6	2	9	5	2	4	5	8	8	6	463.55
	6048.	4429.	4532.	4658.	4720.	4770.	4785.	4584.	4474.	4383.	4195.	3919.	3721.	3381.	3184.	2969.	2773.	2776.	2677.	2736.	4598.0
Belize	83	18	50	97	83	22	79	11	08	14	96	95	17	28	96	31	30	36	25	11	8
	44250	31666	26141	24316	22294	20768	19189	17385	16248	15226	15666	14959	14946	14533	14230	14104	13327	12425	11863	11129	26617.
Benin	.05	.16	.69	.17	.50	.53	.27	.99	.89	.92	.19	.96	.70	.19	.68	.31	.33	.32	.82	.37	58
	1379.	1283.	1284.	1303.	1336.	1330.	1313.	1286.	1215.	1183.	1081.	1038.	964.5	907.4	827.3	782.8	739.4	717.6	725.5	761.5	1133.7
Bermuda	68	89	94	02	83	12	03	67	38	71	66	55	0	2	8	3	0	7	1	8	8
	9858.	5611.	5550.	5701.	5619.	5577.	5519.	5346.	5187.	5078.	4976.	4970.	4939.	4952.	4890.	4853.	4837.	4962.	4869.	4943.	5787.6
Bhutan	42	29	78	08	79	38	73	91	80	25	33	65	83	92	96	72	04	67	96	32	3
Bolivia (Plurinational	10671	8955.	9043.	8946.	8822.	8932.	8293.	7921.	7119.	6353.	5329.	4519.	3755.	3354.	2849.	2557.	2284.	2154.	2224.	2377.	7997.6
State of)	.28	99	95	88	34	24	25	23	20	42	77	44	94	07	22	76	99	09	32	56	1
	11266	8905.	9247.	9576.	9855.	9822.	9713.	9177.	8546.	7900.	7574.	7313.	7238.	7116.	7196.	7356.	7412.	7426.	7491.	7446.	8528.6
Bosnia and Herzegovina	.27	64	64	70	59	63	86	89	91	22	78	66	41	18	05	03	88	16	21	82	0
	18882	13331	10525	9783.	8828.	8160.	7366.	6314.	5560.	4683.	4038.	3460.	2966.	2605.	2387.	2409.	2388.	2459.	2473.	2541.	8986.2
Botswana	.47	.70	.11	59	55	93	64	37	92	89	83	20	37	03	35	38	67	37	20	93	6
	11775	10718	10435	10371	10153	9995.	9999.	9774.	9651.	9463.	9530.	9169.	9109.	8910.	8739.	8307.	7882.	7693.	7590.	7606.	9928.7
Brazil	.99	.83	.14	.04	.78	54	03	32	61	75	43	92	43	68	93	16	09	00	57	47	4

Brunei Darussalam	2182.02	1800.66	1508.64	1349.55	1201.13	1022.03	866.36	697.87	586.31	489.48	426.75	376.90	378.09	385.94	391.89	401.94	420.26	442.48	467.79	480.67	1015.79
Bulgaria	10835.30	8456.55	8663.55	9007.37	9443.41	9305.05	9032.88	8532.90	7940.75	7264.94	6826.69	6716.91	6785.01	6761.30	6907.75	6809.71	6968.86	6997.78	7045.03	7243.00	7918.86
Burkina Faso	35424.71	30710.48	27927.87	26439.21	25111.76	23888.05	22855.88	22112.94	21141.35	20442.78	20246.86	20128.12	19960.75	19963.78	19388.82	19250.66	19139.39	18532.59	17731.27	16457.18	27125.12
Burundi	31779.01	15327.54	15458.85	18667.32	21147.96	23556.14	25208.73	26797.62	27174.44	27450.22	27008.21	27205.33	26893.11	26725.78	26494.67	26209.66	25695.54	24975.59	24180.02	23047.09	22836.18
Cape Verde	6982.59	6735.36	5842.25	5513.13	5236.71	4867.32	4560.91	4269.79	3977.30	3754.05	3661.40	3573.67	3353.76	3234.73	3235.45	3165.93	3056.07	3056.86	3105.46	2894.14	5000.02
Cambodia	18723.16	11615.93	9359.31	8668.59	7678.03	6255.83	4609.93	3048.00	1780.57	1145.57	786.70	663.25	760.65	886.44	1030.76	1176.26	1358.04	1504.48	1649.69	1737.70	7010.57
Cameroon	27907.95	29105.68	30918.64	30417.26	29017.70	27004.38	25284.83	24078.57	22719.46	21802.71	21498.85	21141.31	20990.03	20651.47	20508.54	19662.20	18629.49	17247.76	16005.99	13674.93	27274.48
Canada	2816.73	2062.98	1525.10	1166.16	873.74	684.35	550.11	456.82	380.27	327.52	280.20	255.95	234.23	221.20	209.59	192.61	182.62	179.90	185.27	197.20	718.82
Central African Republic	53732.78	33185.25	31140.06	30841.07	29468.72	28903.87	28329.00	27724.09	27041.09	27015.70	25560.39	24103.53	22858.05	21991.75	20074.32	18949.95	18170.13	17554.35	17167.34	16684.55	33206.55
Chad	51316.22	33586.10	33503.76	31629.47	29363.24	27444.86	25428.67	23923.91	22854.81	22267.44	22813.38	23023.35	23253.63	22897.53	22737.28	22607.04	21952.02	21127.79	19164.81	18220.86	33844.32

Chile	7419.39	6592.50	5564.39	5124.88	4748.74	4224.75	3610.24	3051.52	2472.30	2031.83	1630.69	1377.23	1290.66	1184.56	1130.09	1074.41	1020.91	1003.77	1022.52	1063.47	3530.95
China	3854.30	3666.75	2970.06	2503.61	2594.18	2488.84	2274.66	1901.75	1572.39	1209.55	905.98	656.22	486.00	364.16	302.72	227.64	233.04	245.91	257.21	257.16	1776.19
Colombia	3631.34	2819.68	2723.33	2718.91	2960.04	2969.35	2997.03	2917.01	2907.83	2892.47	2906.87	2818.73	2798.96	2790.96	2700.63	2650.70	2524.56	2423.73	2325.06	2332.43	2912.02
Comoros	25468.07	15086.66	16200.33	18562.69	19563.66	20932.03	21288.85	21702.22	21163.58	20796.46	20665.46	19921.64	19669.83	19432.48	19367.85	19150.79	19270.51	18762.37	18280.28	17554.11	19836.59
Congo	42347.40	30938.42	28288.88	26721.18	24977.72	23347.57	22008.80	19797.42	18867.24	18217.16	17453.08	16372.34	15030.53	14162.12	13325.27	13505.68	12695.95	12266.85	11725.56	10990.53	26140.66
Cook Islands	7237.18	6375.34	5875.80	5177.94	4443.41	3829.69	3132.46	2335.37	1573.63	1105.43	816.18	642.72	649.49	617.92	610.71	578.84	576.78	567.73	560.94	577.68	3157.06
Costa Rica	3370.46	2898.56	2916.53	2932.29	2923.28	2890.13	2883.18	2833.49	2778.68	2694.68	2622.14	2501.84	2418.38	2410.13	2264.18	2190.38	2044.41	1919.14	1842.20	1811.34	2792.16
Cote d'Ivoire	26187.70	19027.17	14160.13	11938.20	10570.69	9774.70	8963.27	8369.64	7692.09	7337.38	7273.45	7259.49	7319.31	7299.29	7252.21	7327.07	7391.15	7291.41	7308.79	7395.24	13786.62
Croatia	9332.78	7569.78	7761.07	8009.76	8319.36	8144.56	8220.62	7592.15	6957.72	6618.59	6261.40	6101.57	5842.53	5947.08	5924.41	6147.32	6214.29	6378.70	6515.84	6828.66	7068.61
Cuba	2632.74	2222.49	2192.59	2197.72	2235.79	2304.43	2338.79	2377.20	2341.74	2266.71	2260.81	2185.70	2105.11	2024.82	1923.66	1860.88	1790.35	1721.34	1708.33	1714.19	2219.56

Cyprus	2034.31	1376.15	977.50	767.97	610.51	508.41	421.29	338.95	301.99	279.57	262.85	267.53	293.29	331.64	373.69	426.94	464.66	502.46	530.85	556.71	573.71
Czechia	6322.48	5650.56	5698.88	5988.70	6235.01	6284.79	6015.19	5683.11	5330.12	4847.25	4711.56	4459.51	4428.65	4461.64	4424.25	4516.67	4643.70	4810.30	5084.16	5157.70	5268.73
Democratic People's Republic of Korea	17418.66	12106.51	9425.15	9102.66	8598.40	7595.92	6589.94	4888.70	3260.38	2250.24	1675.39	1363.17	1268.89	1159.30	1083.11	898.13	938.53	955.29	959.04	981.80	6015.13
Democratic Republic of the Congo	51431.38	29968.54	26495.28	26734.15	26245.52	25826.74	25790.39	24774.85	23831.11	22958.80	21708.04	20843.44	19452.59	18703.97	17336.52	16780.62	16295.61	15728.17	14883.96	14481.06	29839.62
Denmark	1328.17	917.83	629.43	510.90	415.28	338.86	280.14	230.57	202.98	184.19	180.39	183.17	195.93	223.66	246.55	278.05	307.72	337.73	369.90	379.67	381.71
Djibouti	22902.03	13495.21	13068.64	15217.19	16099.68	17120.46	16881.75	17073.86	17193.10	17496.71	17190.39	17320.13	17326.94	16960.40	16718.85	16196.19	15544.73	15072.89	14705.93	13551.21	16798.20
Dominica	2544.89	2236.89	2173.27	2236.84	2253.54	2199.23	2214.34	2166.35	2110.44	1959.76	1849.22	1741.70	1559.09	1485.63	1400.63	1305.34	1236.97	1225.82	1203.67	1284.11	2026.83
Dominican Republic	5930.03	4865.62	4828.07	4795.16	4802.84	4785.91	4728.83	4716.98	4613.72	4580.89	4418.45	4362.58	4115.99	3997.17	3843.83	3573.87	3422.49	3301.25	3351.54	3182.01	4753.97
Ecuador	6878.71	4803.97	4773.87	4613.82	4142.19	3629.39	2961.76	2309.77	1775.23	1372.05	906.90	517.15	235.41	120.22	72.05	47.61	37.14	33.77	34.06	31.97	3315.65
Egypt	2952.10	1937.88	1755.95	1736.40	1766.40	1811.00	1805.11	1800.17	1782.10	1814.09	1806.17	1791.16	1772.28	1772.68	1803.29	1776.41	1754.23	1750.02	1728.86	1700.45	1927.43

	7853.	6196.	5706.	5737.	5763.	5718.	5530.	5481.	5349.	5208.	5090.	4945.	4753.	4659.	4462.	4256.	4016.	3790.	3584.	3571.	5677.1
El Salvador	69	05	73	07	65	15	32	51	97	65	84	04	55	99	79	37	83	05	39	83	9
	6547.	4703.	4583.	4602.	4488.	4242.	4002.	3731.	3322.	3081.	2861.	2649.	2480.	2379.	2262.	2245.	2211.	2158.	2085.	2012.	4481.6
Equatorial Guinea	77	74	33	06	97	66	87	06	69	36	30	55	47	45	16	81	10	53	47	08	1
	31043	17269	17991	20673	22303	23231	23446	24003	23396	23275	22672	21865	21117	21230	20160	20172	19402	19073	18198	17746	22425.
Eritrea	.97	.77	.83	.49	.14	.87	.03	.06	.45	.48	.45	.18	.88	.78	.67	.82	.28	.66	.48	.09	80
	657.1	595.5	559.6	620.0	714.6	795.3	854.5	877.1	838.2	738.9	693.9	651.8	659.5	643.3	645.6	644.2	642.1	650.4	677.8	702.1	
Estonia	7	7	4	3	7	5	3	9	4	1	7	6	2	1	9	7	0	8	5	2	706.57
	17155	10834	9485.	8518.	7506.	6792.	5902.	5263.	4492.	3843.	3386.	2878.	2553.	2358.	2247.	2264.	2274.	2311.	2381.	2343.	8270.4
Eswatini	.65	.14	68	98	94	09	59	27	93	27	30	01	63	95	08	88	27	86	87	07	3
	35695	21529	18902	21362	22817	24088	25016	25615	26453	26795	26602	26304	26092	26103	26624	26365	26237	25983	25060	24220	24921.
Ethiopia	.39	.00	.74	.80	.90	.58	.91	.16	.75	.16	.47	.93	.50	.50	.77	.54	.68	.80	.17	.05	46
	11551	11365	10306	9431.	8628.	7704.	6461.	4934.	3479.	2547.	1995.	1761.	1730.	1718.	1664.	1706.	1705.	1743.	1806.	1881.	6896.4
Fiji	.43	.77	.57	65	14	34	84	27	17	65	81	61	26	71	15	85	25	99	89	85	5
	1651.	1105.	778.9	604.7	507.6	415.0	340.2	282.2	243.6	226.1	213.1	217.3	234.0	267.9	297.1	342.1	369.5	405.4	442.4	458.6	
Finland	76	83	6	9	5	9	7	7	6	0	3	6	6	8	7	8	6	9	7	3	453.56
	479.0	313.9	219.7	174.1	148.4	132.8	118.8	110.5	103.5					100.2	105.6	112.8	117.2	125.5	129.7	137.0	
France	2	0	6	3	5	8	9	2	1	96.07	95.95	92.56	94.31	5	1	9	8	9	7	4	153.46
	6952.	4993.	4783.	4651.	4493.	4296.	4107.	3899.	3600.	3379.	3231.	2986.	2828.	2684.	2448.	2400.	2264.	2195.	2152.	2125.	4467.9
Gabon	34	69	74	70	04	50	04	20	07	79	77	96	64	46	56	74	39	05	30	97	9

	34154	25872	22326	21319	20099	18967	18085	17076	16460	16171	15639	16006	15715	15527	15055	14387	14061	13660	12870	12137	22262.
Gambia	.17	.59	.03	.66	.15	.32	.32	.29	.38	.60	.56	.83	.34	.55	.04	.54	.05	.61	.48	.34	05
	3376.	2653.	2223.	2202.	2244.	2256.	2301.	2307.	2251.	2203.	2110.	2004.	1962.	1946.	1933.	1880.	1810.	1805.	1780.	1771.	2255.9
Georgia	89	74	48	81	00	13	40	68	99	11	85	57	52	16	15	89	93	74	98	38	0
	782.6	569.4	435.3	384.7	347.1	314.2	299.3	268.7	246.3	232.0	212.8	206.0	204.1	197.0	196.5	186.7	181.2	181.0	179.9	181.1	
Germany	9	3	6	0	6	1	3	4	5	7	6	9	7	3	9	8	3	5	6	6	294.30
	26608	19428	17371	16184	14392	12789	11670	10465	9783.	9054.	8667.	8611.	8520.	8311.	8057.	7780.	7547.	7285.	6659.	6037.	15297.
Ghana	.62	.33	.03	.68	.13	.41	.07	.34	85	06	34	69	93	10	90	22	69	82	69	91	09
	2494.	1701.	1172.	939.2	753.9	627.0	519.6	429.2	368.0	345.0	325.2	324.7	356.1	401.3	457.9	507.1	553.7	606.4	677.9	745.9	
Greece	45	95	30	5	6	0	4	3	2	4	8	9	4	2	3	2	7	5	9	6	658.33
	2293.	1822.	1390.	1122.	914.6	782.9	682.4	603.6	507.5	465.4	423.7	401.3	357.1	348.4	319.1	321.9	314.6	316.4	318.6	319.2	
Greenland	27	14	72	31	3	4	6	6	0	3	9	7	7	1	0	8	8	4	5	2	852.92
	4315.	3706.	3671.	3773.	3844.	3809.	3745.	3589.	3513.	3271.	3154.	2878.	2659.	2515.	2305.	2164.	2105.	2091.	2027.	2089.	3472.7
Grenada	16	62	37	48	22	36	69	05	12	00	69	67	92	29	12	72	29	49	92	96	5
	4996.	4824.	4506.	4061.	3633.	3064.	2457.	1820.	1212.	839.1	613.7	509.2	490.5	481.9	465.9	452.6	452.4	457.3	446.1	470.6	2632.4
Guam	34	27	70	70	03	25	49	83	85	8	6	4	5	5	8	2	4	9	2	6	5
	9245.	5179.	5285.	5322.	5499.	5574.	5632.	5744.	5655.	5713.	5757.	5646.	5641.	5794.	5703.	5686.	5507.	5206.	4885.	4565.	5916.5
Guatemala	14	80	82	58	24	92	39	21	04	37	52	80	06	92	96	59	37	23	67	97	2
	38037	27861	24432	22866	20943	18835	17681	16442	15994	14766	15273	15287	15143	15438	15346	14877	14451	13325	12898	12156	24015.
Guinea	.16	.20	.42	.36	.86	.03	.49	.15	.20	.69	.37	.67	.12	.17	.63	.17	.43	.83	.21	.74	27

	40238	32496	27620	26172	24338	22844	21293	20036	18771	17789	17798	17707	17492	17337	16612	15803	15113	14610	13676	12824	26867.
Guinea-Bissau	.56	.92	.55	.45	.48	.50	.24	.83	.31	.92	.01	.62	.81	.58	.08	.28	.44	.30	.65	.62	45
	6180.	4976.	4956.	5001.	5118.	5079.	4995.	4881.	4711.	4463.	4242.	3899.	3551.	3377.	3068.	2946.	2713.	2662.	2617.	2584.	4813.3
Guyana	53	68	08	65	34	39	89	71	36	80	64	44	81	71	97	08	33	32	60	16	0
	19900	13364	12552	12634	12574	12618	12602	12547	12399	12376	12275	12078	11844	11563	11193	10727	10417	10276	10043	9968.	13454.
Haiti	.66	.42	.76	.00	.90	.80	.70	.09	.23	.36	.49	.15	.39	.77	.94	.85	.26	.48	.16	29	54
	7052.	5528.	5419.	5604.	5840.	5866.	6000.	6073.	6074.	6097.	6104.	6056.	6083.	6222.	6212.	6285.	6120.	5928.	5694.	5833.	5963.8
Honduras	66	62	04	73	04	39	67	56	67	92	06	86	07	75	77	10	56	38	13	54	5
	8351.	6691.	6739.	7191.	7365.	7367.	7238.	6917.	6411.	5848.	5621.	5554.	5461.	5415.	5630.	5791.	5850.	5930.	6131.	6195.	6398.4
Hungary	33	27	94	16	10	40	78	33	71	36	61	51	27	13	97	74	59	80	53	83	8
	1459.	994.4	699.0	538.3	449.5	366.7	308.4	245.5	216.8	199.2	191.7	195.9	213.3	235.6	262.0	301.8	335.1	358.8	397.8	407.6	
Iceland	85	7	6	5	0	5	6	2	9	8	5	2	5	6	7	8	7	1	8	4	439.38
	16816	8069.	7549.	7733.	7445.	7391.	7170.	6899.	6680.	6574.	6560.	6303.	6371.	6384.	6362.	6150.	6086.	6012.	6105.	6018.	7969.7
India	.95	44	09	87	63	40	10	06	50	34	29	48	48	10	31	68	57	04	88	85	3
	16639	8434.	6968.	6456.	5757.	5013.	4066.	3033.	2195.	1702.	1361.	1164.	1184.	1239.	1273.	1313.	1371.	1350.	1365.	1420.	5141.8
Indonesia	.37	21	23	49	71	52	13	88	10	85	46	39	20	66	46	94	07	50	07	11	9
	2246.	1577.	1377.	1279.	1261.	1213.	1201.	1153.	1151.	1130.	1118.	1116.	1113.	1063.	1069.	1093.	1108.	1091.	1104.	1114.	1311.4
Iran (Islamic Republic of)	18	81	99	08	71	62	46	80	47	58	24	95	17	55	87	46	91	21	48	66	8
	5292.	3375.	3111.	2887.	2883.	2923.	2872.	2825.	2740.	2662.	2639.	2639.	2605.	2600.	2583.	2544.	2523.	2476.	2493.	2458.	3175.8
Iraq	08	95	54	74	21	58	08	35	51	16	12	67	57	63	46	90	19	92	35	87	0

Ireland	1365.88	925.96	654.59	527.80	422.99	346.05	291.74	239.34	208.64	191.42	180.27	181.17	199.78	230.08	252.33	281.99	313.51	347.59	381.02	399.59	420.09
Israel	11702.74	8627.86	6419.35	4853.31	3403.68	2314.38	1574.27	1001.44	717.53	552.27	457.16	418.35	408.39	440.80	481.65	518.52	556.48	588.36	633.28	670.66	3667.89
Italy	2563.95	1820.61	1367.91	1164.92	1066.29	993.94	949.69	939.10	971.71	1112.94	1343.13	1656.41	2164.76	2398.96	2540.25	2541.36	2482.75	2376.90	2234.68	2069.87	1611.24
Jamaica	2858.12	2553.62	2556.01	2601.20	2578.25	2640.28	2592.31	2534.08	2433.97	2326.33	2202.25	2009.39	1871.40	1747.39	1578.29	1491.48	1429.29	1397.40	1387.65	1379.07	2406.10
Japan	1621.75	1376.00	1143.40	1035.48	893.38	793.38	657.98	549.51	452.58	376.59	328.73	296.53	289.89	303.33	304.52	303.45	322.55	325.85	350.75	369.27	573.02
Jordan	6865.76	6301.12	4829.88	4635.69	4605.50	4620.33	4523.61	4364.72	4387.98	4284.63	4186.69	4273.20	4166.23	4228.33	4143.04	4176.91	4138.04	4169.62	4243.76	4405.83	4943.23
Kazakhstan	12750.09	9582.37	7042.34	6864.17	6712.54	6512.90	6566.62	6761.28	6413.72	6154.38	6215.14	6062.60	5913.58	5567.22	5284.01	4962.84	4707.63	4494.58	4267.34	4069.86	7348.56
Kenya	46813.61	29877.15	27340.34	28508.39	27411.87	26990.64	26632.90	25444.17	24630.85	24220.35	24334.96	23857.48	24416.18	24126.26	24288.44	24621.73	24690.51	24884.31	25166.29	25301.87	29538.69
Kiribati	43331.75	33558.48	31709.69	29370.95	26431.68	23614.46	19541.09	15563.97	10994.64	7938.21	5949.35	4774.87	4655.00	4595.08	4388.34	4228.77	4184.14	4020.89	4032.57	3995.47	24151.07
Kuwait	1070.51	862.06	765.02	715.94	726.71	721.45	732.45	694.76	692.16	661.72	636.53	645.92	639.46	638.80	638.32	624.36	620.96	623.98	615.23	631.34	735.05

	6549.	4865.	3965.	3913.	3993.	4151.	4223.	4201.	4164.	3974.	3794.	3648.	3562.	3573.	3470.	3383.	3249.	3268.	3193.	3189.	4369.4
Kyrgyzstan	03	44	33	60	80	02	63	78	38	74	29	47	15	49	69	46	29	55	58	11	1
Lao People's Democratic	22042	13309	12091	11633	10779	10296	9564.	8693.	7462.	6578.	5732.	5238.	5314.	5235.	5129.	5059.	5089.	4997.	4824.	4681.	11071.
Republic	.24	.88	.43	.84	.10	.25	20	73	35	75	86	51	50	44	22	90	79	59	09	81	54
	700.4	620.8	586.0	663.4	756.8	823.0	913.0	924.8	888.0	818.5	742.7	703.1	693.3	705.6	690.6	685.1	697.1	696.2	704.4	719.4	
Latvia	3	4	9	5	4	5	4	8	2	4	1	3	6	5	4	7	2	4	7	4	751.21
	2779.	1860.	1608.	1550.	1550.	1528.	1547.	1502.	1473.	1421.	1403.	1393.	1370.	1357.	1370.	1362.	1352.	1333.	1329.	1361.	1648.0
Lebanon	46	47	55	41	02	33	87	27	33	02	43	60	77	48	55	05	11	34	63	25	3
	33636	18739	16998	15582	13872	12263	11106	9680.	8412.	7248.	6283.	5429.	4795.	4302.	4059.	4028.	4031.	4155.	4156.	4129.	14522.
Lesotho	.57	.08	.82	.47	.39	.57	.14	60	79	30	86	42	24	75	09	77	01	84	09	63	76
	21713	14094	12887	12567	11994	11528	11134	11277	11146	11012	10835	10779	10690	10436	10108	9734.	9776.	10294	9858.	9264.	13366.
Liberia	.58	.17	.11	.61	.69	.25	.44	.36	.70	.26	.34	.77	.62	.72	.51	29	78	.76	51	77	13
	3590.	2072.	1778.	1700.	1677.	1678.	1646.	1694.	1598.	1592.	1562.	1495.	1545.	1541.	1497.	1509.	1487.	1478.	1497.	1482.	1797.2
Libya	23	72	46	74	45	76	93	98	30	52	36	83	94	99	96	50	90	01	25	31	2
	627.5	554.0	525.4	588.4	682.4	758.3	809.2	851.0	807.9	735.8	675.4	635.4	627.8	619.2	629.5	627.7	615.4	628.5	633.9	656.4	
Lithuania	4	7	5	5	7	9	3	9	8	1	7	7	4	6	1	4	2	6	6	4	676.69
	1271.	868.6	605.3	494.5	396.6	329.7	269.0	223.4	193.5	176.5	168.4	169.3	187.2	212.5	236.3	264.0	292.9	325.4	351.5	362.9	
Luxembourg	88	5	7	9	0	1	1	3	4	7	0	4	3	6	7	9	4	6	1	5	359.45
	36586	17873	16499	18850	20166	21491	21878	22309	21987	21741	21583	21521	21338	20982	21128	20590	20233	20383	19761	19431	22274.
Madagascar	.33	.96	.45	.49	.22	.05	.93	.85	.32	.54	.79	.59	.21	.85	.03	.89	.85	.43	.50	.92	11

	32571	22813	20941	25540	27279	27743	27861	27995	27888	27513	26528	27429	27045	27247	27156	27426	27555	27716	28258	28557	26386.
Malawi	.36	.46	.42	.49	.88	.62	.99	.76	.56	.15	.72	.22	.91	.35	.16	.26	.76	.38	.62	.86	35
	1291.	624.3	559.9	603.8	612.2	580.0	498.4	375.5	256.7	186.3	148.2	139.2	167.4	186.1	198.0	203.2	205.4	203.5	197.5	206.7	
Malaysia	15	1	5	9	1	7	7	5	6	5	4	1	6	7	9	6	4	0	8	3	499.23
	6904.	4930.	4562.	4178.	3564.	2804.	2044.	1290.	727.2	478.6	334.6	282.9	333.7	380.1	451.3	524.6	605.9	678.2	761.9	799.6	2652.5
Maldives	03	46	77	82	95	17	34	09	0	6	4	7	5	7	9	2	4	8	4	8	9
	46112	33444	29071	27311	25878	23709	22503	21260	20336	20032	19982	19514	19641	19501	19075	18916	19297	19188	18667	18179	29889.
Mali	.49	.33	.00	.64	.28	.92	.54	.73	.04	.47	.56	.91	.88	.68	.10	.74	.14	.97	.09	.93	14
	2447.	1641.	1164.	924.1	739.6	625.5	496.3	420.0	362.5	332.6	317.7	326.2	350.7	397.0	447.9	505.5	546.0	593.9	644.2	662.2	
Malta	95	59	85	1	7	7	2	9	6	1	9	2	6	7	4	8	6	9	0	4	658.90
	33073	26885	25869	25089	23910	22343	20434	17946	14153	11876	9905.	8587.	8129.	7882.	7441.	6996.	6662.	6376.	6291.	5972.	21270.
Marshall Islands	.47	.93	.39	.98	.58	.49	.82	.18	.65	.16	16	24	61	17	45	17	63	78	33	20	27
	17922	12661	10318	9554.	8893.	8127.	7655.	6939.	6330.	6112.	5954.	5985.	5925.	5971.	6033.	5792.	5828.	5989.	5847.	5791.	10065.
Mauritania	.52	.52	.76	91	70	76	60	65	76	49	22	41	58	46	93	42	54	58	84	63	11
	3637.	2678.	2154.	1960.	1673.	1366.	1012.	658.8	377.8	235.5	163.7	131.5	153.6	176.9	209.5	237.9	277.7	307.6	328.7	361.9	1056.7
Mauritius	90	39	54	51	56	06	60	9	3	2	3	3	1	9	6	8	4	4	7	7	6
	11960	9717.	7650.	7173.	6902.	6545.	6374.	6033.	5812.	5294.	4722.	4078.	3324.	2868.	2464.	2181.	1960.	1728.	1603.	1562.	6638.3
Mexico	.81	42	49	82	33	27	93	06	25	32	76	56	30	17	89	71	34	76	71	31	1
Micronesia (Federated	43101	36492	35428	35832	34848	34894	33894	33046	31009	29830	28259	27269	27030	25980	24862	23458	22473	21989	22173	21527	33980.
States of)	.96	.44	.20	.25	.64	.06	.11	.46	.69	.06	.73	.54	.86	.73	.60	.21	.68	.79	.27	.85	80

Monaco	954.00	651.93	485.59	374.36	306.19	255.99	207.67	178.91	152.74	137.70	132.26	130.23	144.53	164.02	184.53	209.46	232.69	249.49	282.96	293.28	258.47
Mongolia	2008.08	1249.27	1091.04	1100.32	1098.76	1111.39	1146.04	1137.31	1145.10	1123.69	1140.81	1122.16	1107.59	1094.84	1098.96	1109.84	1090.21	1089.77	1043.95	1053.13	1236.76
Montenegro	7901.04	6202.53	6460.73	6600.56	6900.91	6960.88	6801.32	6571.74	6117.05	5696.11	5383.65	5144.06	4984.05	5024.03	5146.85	5135.39	5239.72	5171.33	5304.09	5241.95	6097.73
Morocco	9554.27	7104.67	6840.27	5781.14	5705.46	5417.91	5404.77	5225.87	5162.14	5029.29	4984.84	5055.83	5058.37	4928.62	4873.89	4777.42	4745.14	4734.39	4973.99	5050.18	5953.99
Mozambique	41320.84	26932.13	23446.53	25045.04	25608.36	26841.62	28465.50	29061.47	29568.50	29126.13	29074.44	29009.55	28947.84	28515.41	27906.50	26936.22	25665.56	24622.04	23625.52	22412.01	29120.03
Myanmar	14644.74	8559.25	6590.03	6103.58	5187.85	4084.19	3016.32	1989.13	1129.87	722.21	497.84	410.90	470.79	548.43	642.19	740.62	841.98	943.29	1035.35	1075.10	4639.95
Namibia	11604.55	8104.23	7195.05	6586.01	6151.09	5507.76	4862.39	4293.57	3803.45	3221.13	2816.95	2465.59	2151.45	1947.37	1787.53	1853.76	1835.00	1883.83	1906.90	1977.70	6182.69
Nauru	17204.95	15029.08	13768.49	12952.41	11334.37	9976.28	7967.84	6114.08	4044.37	2862.81	2078.28	1684.07	1644.97	1608.29	1561.23	1501.89	1441.92	1427.03	1430.67	1434.69	10591.73
Nepal	9626.03	5991.09	4873.03	5269.42	5405.84	5429.57	5227.27	5008.32	4876.98	4704.48	4718.41	4728.51	4736.50	4740.81	4777.11	4708.85	4777.86	4711.99	4877.88	5017.67	5600.14
Netherlands	1494.72	995.86	715.07	569.90	461.06	372.82	315.69	252.06	225.16	202.37	195.96	199.26	215.15	242.14	273.84	305.77	341.16	366.62	410.30	422.80	416.55

	745.4	755.0	676.4	659.6	634.0	594.1	544.5	469.6	407.9	354.8	305.2	282.4	273.3	279.2	287.4	293.4	299.3	313.9	339.6	354.0	
New Zealand	2	6	3	3	8	0	1	9	7	1	6	8	2	8	3	8	2	6	2	4	479.51
	1310.	1556.	1616.	1659.	1728.	1826.	1882.	1919.	2069.	2163.	2166.	2258.	2372.	2515.	2672.	2906.	2900.	2947.	2918.	2854.	1830.3
Nicaragua	44	61	84	99	59	75	77	38	54	55	13	33	55	59	31	44	43	13	76	73	4
	64666	45683	41027	38676	36726	34779	33285	31130	28961	27468	27355	28278	27974	27048	27217	27189	26639	26762	26474	25252	43159.
Niger	.94	.93	.19	.04	.48	.79	.33	.39	.10	.18	.94	.06	.39	.25	.97	.45	.52	.10	.07	.24	09
	8058.	4301.	3869.	4945.	5078.	5276.	5343.	5263.	5121.	4527.	4683.	4667.	4577.	4615.	4468.	4527.	4558.	4536.	4630.	4695.	5232.5
Nigeria	83	72	28	83	45	79	92	80	39	89	64	64	28	09	18	81	91	68	74	81	4
	10221	9106.	8590.	7728.	6634.	5809.	4652.	3461.	2355.	1595.	1177.	933.3	942.2	907.3	895.6	852.9	852.8	828.3	835.4	825.3	4715.5
Niue	.64	79	42	42	78	45	40	48	93	73	44	0	3	4	3	1	9	4	2	6	6
	16489	13708	13402	13344	13476	13303	12928	12691	12187	11722	11271	10984	10543	10344	10202	10010	9746.	9541.	9686.	9250.	12339.
North Macedonia	.51	.61	.59	.66	.33	.55	.33	.55	.68	.45	.95	.93	.32	.31	.56	.63	05	95	90	03	39
	7574.	7311.	6775.	6029.	5341.	4476.	3734.	2827.	1783.	1272.	945.1	722.4	753.4	706.6	699.7	659.0	671.1	664.4	655.6	656.5	3354.6
Northern Mariana Islands	24	80	51	93	29	32	07	82	08	42	9	8	2	2	0	5	3	5	4	3	1
	1201.	815.4	574.8	457.7	365.7	307.2	250.9	211.4	180.8	165.6	158.6	159.0	174.3	197.5	225.7	248.1	277.1	301.5	326.7	346.5	
Norway	09	4	2	1	8	3	4	5	7	5	8	3	1	3	6	6	8	4	8	9	348.54
	2356.	1419.	1258.	1187.	1201.	1204.	1168.	1168.	1138.	1126.	1110.	1072.	1082.	1077.	1068.	1075.	1055.	1083.	1048.	1062.	1292.2
Oman	05	53	17	72	49	85	25	80	09	62	66	63	25	73	58	53	96	68	92	43	0
	6898.	3778.	2847.	2992.	2921.	2974.	2844.	2817.	2800.	2728.	2758.	2699.	2699.	2711.	2741.	2765.	2749.	2784.	2747.	2737.	3530.5
Pakistan	52	41	97	92	66	17	57	38	42	16	26	86	28	00	44	32	26	27	34	15	8

	10331	8871.	8278.	7604.	6901.	6244.	5021.	3637.	2358.	1610.	1161.	939.8	938.3	915.4	842.9	852.4	831.6	820.4	812.7	807.6	4299.4
Palau	.79	29	74	29	09	39	44	94	20	96	60	7	2	9	1	0	4	3	8	6	4
	8307.	6057.	5113.	4808.	4822.	4879.	4810.	4742.	4632.	4461.	4464.	4420.	4459.	4422.	4259.	4164.	4108.	4042.	3869.	3585.	5381.0
Palestine	11	33	47	76	76	16	22	06	30	97	40	60	90	53	05	27	68	32	61	88	2
	3739.	2751.	2769.	2776.	2752.	2778.	2767.	2742.	2654.	2641.	2537.	2501.	2445.	2353.	2205.	2121.	1997.	1888.	1832.	1808.	2753.4
Panama	72	57	56	48	55	16	07	76	37	71	46	84	90	14	62	51	05	01	27	07	0
	18961	12066	11895	11770	11447	11162	10357	9368.	7837.	6534.	5475.	4770.	4788.	4660.	4701.	4591.	4552.	4693.	4534.	4598.	11319.
Papua New Guinea	.59	.59	.55	.84	.57	.87	.20	84	29	10	01	06	20	29	15	47	15	60	48	61	47
	7630.	6981.	6936.	6771.	6722.	6376.	6321.	5864.	5491.	5108.	4757.	4371.	3873.	3447.	3214.	2834.	2647.	2495.	2485.	2474.	6074.7
Paraguay	75	68	01	75	26	45	47	46	11	96	76	72	52	80	23	95	10	91	20	96	3
	6660.	6582.	6486.	6505.	6222.	6189.	5980.	5810.	5518.	5387.	5259.	5082.	4733.	4384.	4021.	3816.	3641.	3702.	3701.	3786.	5871.6
Peru	93	82	59	34	80	92	43	23	34	05	56	96	83	31	92	87	12	66	87	47	6
	23552	12906	10285	9534.	8540.	7244.	5716.	3976.	2383.	1507.	1012.	817.1	871.5	924.4	1009.	1070.	1146.	1197.	1265.	1342.	8391.6
Philippines	.55	.16	.41	85	81	88	77	94	71	06	08	0	1	0	25	20	37	74	19	36	3
	7110.	5817.	6102.	6254.	6618.	6682.	6521.	6013.	5642.	5261.	5079.	4845.	4897.	4881.	4862.	5051.	5183.	5299.	5498.	5843.	5720.2
Poland	53	57	03	38	46	99	71	97	37	36	69	52	17	81	18	00	68	39	86	66	4
	3373.	2254.	1652.	1274.	1057.	848.7	713.0	581.4	504.4	469.4	442.9	448.8	486.8	561.5	611.9	703.2	765.1	829.3	886.1	942.9	
Portugal	50	49	67	19	57	1	7	6	6	4	4	0	0	9	9	9	7	5	6	1	879.13
	1385.	1252.	1241.	1292.	1324.	1305.	1279.	1270.	1219.	1157.	1080.	1022.	944.1	896.6	829.9	770.6	733.6	694.4	693.8	703.2	1125.1
Puerto Rico	99	40	45	47	57	95	79	07	67	66	99	49	6	7	2	7	5	4	5	8	4

Qatar	1273.92	894.35	783.02	721.54	685.03	671.68	678.78	678.29	672.32	657.85	651.00	629.36	626.73	628.69	621.66	642.45	627.01	607.21	618.10	606.61	718.64
Republic of Korea	1323.42	1202.07	1016.27	892.89	789.22	667.98	576.74	471.06	390.79	330.12	287.40	260.23	250.37	261.38	266.07	275.98	287.23	293.22	312.24	320.51	527.46
Republic of Moldova	1789.74	1630.80	1465.07	1614.42	1857.41	2037.43	2207.48	2243.06	2109.14	1893.66	1781.73	1638.26	1627.72	1587.94	1571.95	1531.33	1512.29	1495.03	1542.00	1566.47	1826.55
Romania	9200.48	7140.07	7299.82	7657.32	7931.05	7820.98	7618.28	7403.13	6679.18	6282.49	6062.18	5892.55	6032.49	5917.51	5897.25	6065.39	6085.88	6109.43	6088.02	6218.30	6850.84
Russian Federation	252.64	173.34	153.02	162.83	169.02	181.71	195.75	209.88	223.30	223.93	218.42	221.24	220.24	229.31	233.58	240.95	249.30	248.37	249.38	256.95	207.61
Rwanda	20784.07	12042.90	11719.89	13279.17	14497.86	15278.68	15626.71	15463.16	15559.59	14696.10	14385.96	14339.70	14199.68	14030.39	13864.48	13843.40	13397.06	13148.30	12864.52	12625.41	14755.90
Saint Kitts and Nevis	2215.02	1966.69	1962.10	2028.34	2005.54	2036.98	2009.82	1969.79	1871.11	1776.92	1665.57	1561.94	1479.95	1342.90	1259.00	1168.83	1125.47	1136.82	1143.84	1181.30	1844.31
Saint Lucia	3313.34	3136.51	3107.50	3238.36	3185.92	3174.35	3150.63	3073.79	2950.16	2810.36	2628.54	2503.40	2248.40	2110.39	1971.66	1822.88	1745.30	1695.86	1682.72	1772.18	2876.06
Saint Vincent and the Grenadines	4859.53	4114.53	4011.67	4095.31	4241.11	4212.99	4111.29	4035.86	3889.04	3672.27	3444.99	3231.68	3006.55	2794.07	2543.81	2385.24	2249.77	2200.43	2173.18	2251.58	3812.77
Samoa	25403.73	24056.42	22585.92	20734.78	19054.38	16585.34	13714.70	10550.93	7404.82	5113.27	3669.97	2943.92	2886.22	2808.35	2741.09	2596.78	2569.76	2541.16	2461.85	2546.44	16127.38

San Marino	1359.11	931.54	661.76	517.35	429.17	350.56	289.92	236.96	208.61	188.47	177.49	184.00	199.63	228.79	253.07	282.65	309.81	339.68	371.76	400.63	385.49
Sao Tome and Principe	18185.11	15127.38	15060.64	13736.99	12654.59	11941.89	10923.52	10068.60	9348.52	8790.68	8349.23	8574.80	8409.93	8077.65	7800.94	7601.12	7596.46	7603.23	7541.12	7053.49	12793.12
Saudi Arabia	469.28	333.78	219.80	120.90	128.41	140.09	145.14	152.90	164.75	168.92	171.61	173.79	170.71	167.80	164.72	167.02	178.58	188.89	194.39	191.86	187.22
Senegal	15496.47	16461.48	15441.71	14894.44	14231.69	13449.83	12871.68	12197.89	11534.28	11009.71	11034.07	10867.41	10795.40	10730.18	10695.44	10330.77	10630.88	10220.28	9725.27	9262.75	14057.89
Serbia	21598.51	17087.43	16845.69	16561.82	16522.38	16278.67	15853.64	15579.61	14643.06	14511.03	13849.66	13687.16	13388.66	13267.01	13076.25	12718.98	12744.70	12507.64	12288.54	12048.29	15248.17
Seychelles	3397.34	2585.88	2160.47	1981.28	1664.98	1289.93	928.30	607.05	341.55	220.77	153.57	128.54	145.82	169.54	196.39	232.89	278.83	310.29	342.52	361.12	1145.25
Sierra Leone	39163.66	27041.54	22802.32	21291.24	19656.98	18587.49	16983.96	16207.03	15324.38	14670.66	15040.53	14287.51	14359.78	13722.87	13576.95	13189.65	12925.00	12852.53	12466.11	11923.40	22747.09
Singapore	1369.50	1246.94	1068.15	943.15	848.18	731.36	621.48	491.96	423.68	340.48	308.51	264.17	272.86	270.95	275.27	293.07	292.22	306.20	320.55	341.14	579.48
Slovakia	7493.61	6035.86	6244.39	6528.18	6755.19	6800.85	6606.93	6183.97	5721.66	5341.06	5126.59	4890.90	4840.17	4891.27	5013.10	5072.07	5187.74	5359.93	5570.53	5668.76	5841.53
Slovenia	5975.40	4899.87	5071.13	5324.27	5489.95	5521.17	5356.49	4983.97	4631.40	4316.50	4242.51	3993.73	3890.87	3881.74	3897.77	4022.70	4130.68	4425.16	4923.08	5182.16	4659.84

	43697	34875	32413	30315	27548	24110	20682	16298	11596	8388.	6079.	4950.	4869.	4782.	4626.	4407.	4406.	4320.	4286.	4312.	26167.
Solomon Islands	.18	.29	.35	.40	.39	.60	.59	.85	.63	00	87	42	96	52	30	72	09	74	54	24	45
	73725	59273	58706	61417	63721	64669	64774	64709	64576	63747	62761	61528	61424	60500	59775	58706	58172	57181	56159	55206	64015.
Somalia	.30	.00	.04	.63	.80	.11	.32	.19	.95	.25	.52	.59	.61	.63	.04	.75	.89	.33	.93	.41	04
	10841	7622.	5542.	3870.	3064.	2476.	2030.	1698.	1435.	1246.	1072.	920.4	838.4	772.1	732.6	737.3	759.9	765.3	788.1	793.0	3622.3
South Africa	.94	46	14	55	17	42	16	73	38	41	16	9	1	5	2	6	2	8	1	1	8
	27721	16083	16811	19486	20455	21047	21066	21329	21177	21467	21254	21541	21492	21384	21162	21593	21209	20736	19962	18806	20651.
South Sudan	.37	.02	.57	.56	.87	.06	.90	.35	.08	.86	.55	.17	.41	.29	.31	.76	.48	.04	.56	.69	15
	1208.	1009.	853.6	794.9	777.3	704.1	650.1	578.6	531.1	481.0	451.4	426.3	397.7	398.6	393.5	401.7	410.0	418.6	412.7	424.6	
Spain	94	50	8	9	2	6	6	8	0	0	2	1	5	3	4	4	4	6	3	9	595.59
	7851.	5522.	4374.	4458.	3925.	3379.	2813.	2213.	1633.	1135.	881.4	750.3	776.5	797.6	824.0	862.7	888.9	913.0	949.3	974.7	2999.9
Sri Lanka	86	13	85	24	65	15	18	67	74	77	8	6	7	0	9	6	2	9	8	0	7
	19510	9366.	7561.	7174.	7092.	7185.	6856.	6858.	6793.	6693.	6539.	6714.	6559.	6392.	6562.	6526.	6419.	6325.	6226.	5992.	9021.6
Sudan	.36	18	66	27	53	08	74	23	42	42	86	83	43	33	59	82	90	09	86	76	5
	5100.	4459.	4382.	4518.	4557.	4543.	4392.	4289.	4168.	3887.	3715.	3490.	3149.	2992.	2738.	2583.	2407.	2319.	2341.	2310.	4148.9
Suriname	97	09	31	46	39	40	35	28	49	57	49	48	98	16	74	36	05	20	83	68	7
	1502.	1032.	712.6	578.4	461.0	381.3	320.6	256.6	229.9	211.9	195.6	203.2	218.0	251.8	278.8	318.4	347.5	382.2	408.8	428.5	
Sweden	40	17	8	7	1	5	9	2	3	1	2	9	7	8	5	3	7	9	2	7	440.44
	1021.	693.6	488.3	387.1	319.9	264.0	213.9	178.4	154.2	139.5	135.9	136.3	147.7	169.4	189.3	214.0	235.1	259.9	288.9	292.8	
Switzerland	10	4	1	2	9	1	3	0	2	1	6	2	7	3	9	7	6	9	3	4	281.59

	7471.	3949.	3560.	3341.	3309.	3322.	3287.	3220.	3202.	3214.	3131.	3126.	3021.	3039.	3038.	3034.	2986.	3042.	3161.	3337.	3656.1
Syrian Arab Republic	90	80	26	22	02	61	27	18	95	24	92	75	87	98	51	53	61	37	98	41	4
Taiwan (Province of	1553.	1337.	1008.	963.2	898.2	804.5	665.1	504.0	348.6	248.0	179.9	145.8	138.6	125.4	113.7		101.3	104.3	104.3	108.3	
China)	54	77	77	4	6	8	7	6	5	6	3	5	3	8	3	97.15	3	0	2	2	516.59
	13887	9164.	6948.	6969.	7015.	7060.	6992.	7026.	6928.	6822.	6743.	6802.	6688.	6636.	6647.	6546.	6408.	6234.	6222.	5828.	8075.1
Tajikistan	.12	24	04	54	19	84	97	98	63	70	64	95	37	14	93	34	37	98	06	96	2
	5742.	2194.	1791.	1705.	1619.	1504.	1278.	1098.	874.9	774.2	755.8	824.3	1170.	1421.	1566.	1635.	1719.	1715.	1669.	1668.	1484.1
Thailand	86	25	75	51	62	89	51	72	2	7	5	8	02	47	55	51	97	55	28	15	3
	22039	9598.	8188.	7311.	6392.	5173.	3735.	2374.	1349.	838.8	575.9	475.9	549.5	658.4	755.6	883.6	1025.	1177.	1277.	1371.	7518.8
Timor-Leste	.99	46	13	28	66	53	13	60	01	3	3	3	5	6	1	9	59	02	42	24	7
	27468	22747	20035	18812	17393	15481	13971	13153	12700	11982	12070	11584	11078	10747	9723.	9678.	9702.	9048.	8627.	8001.	18220.
Togo	.35	.71	.58	.92	.09	.85	.11	.86	.01	.45	.16	.16	.67	.73	67	73	25	17	39	01	76
	16350	14398	13463	12407	10393	9769.	7048.	6097.	3688.	2724.	1926.	1566.	1524.	1493.	1423.	1395.	1355.	1347.	1323.	1345.	8874.7
Tokelau	.17	.14	.89	.40	.54	01	34	80	86	50	45	95	28	26	00	54	91	86	29	75	5
	18215	17097	16172	14685	13123	11066	9000.	7053.	4711.	3302.	2465.	1955.	1928.	1860.	1826.	1732.	1709.	1682.	1632.	1682.	11134.
Tonga	.58	.48	.30	.80	.00	.73	10	79	18	60	55	53	43	75	09	99	51	14	91	09	41
	2217.	2017.	2067.	2117.	2114.	2124.	2088.	2056.	1941.	1835.	1752.	1632.	1549.	1427.	1284.	1212.	1152.	1132.	1101.	1116.	1891.6
Trinidad and Tobago	93	61	89	76	54	66	17	17	68	81	82	45	18	61	06	31	30	28	60	92	3
	2569.	1769.	1651.	1663.	1672.	1707.	1708.	1705.	1676.	1648.	1640.	1666.	1645.	1641.	1635.	1648.	1621.	1664.	1641.	1591.	1746.0
Tunisia	45	64	21	82	84	38	75	18	38	25	17	36	22	81	13	53	21	41	31	25	2

Turkey	4777.53	3048.34	2776.25	3080.27	3165.06	3273.65	3205.09	3185.82	3084.33	2970.78	2910.78	2934.07	2941.74	3004.69	2947.87	3086.20	2985.39	3017.06	3060.46	3113.75	3164.80
Turkmenistan	3169.18	2480.53	2097.64	2085.52	2111.50	2204.61	2206.32	2194.13	2139.01	2058.49	1970.18	1866.29	1888.04	1827.77	1849.99	1756.50	1749.42	1717.62	1713.22	1698.80	2238.98
Tuvalu	24011.60	21095.69	19280.27	18282.67	16711.31	14489.12	12018.80	9040.46	6152.00	4254.76	3121.68	2490.89	2454.23	2322.76	2304.64	2183.15	2161.31	2110.43	2090.88	2122.60	13193.66
Uganda	21576.80	9134.73	8575.85	9498.96	10122.66	10513.25	10801.78	11178.73	11134.28	11243.20	11270.24	11358.89	11252.97	11018.42	10666.88	10754.08	10611.07	10115.99	9655.82	9208.06	11925.49
Ukraine	1494.02	1148.41	1037.73	1170.62	1334.99	1467.91	1598.10	1615.83	1542.75	1402.92	1288.79	1229.75	1206.54	1218.85	1179.72	1188.46	1162.82	1144.17	1199.14	1196.24	1339.53
United Arab Emirates	1207.90	844.17	790.09	804.25	822.04	752.75	670.80	722.51	653.27	652.38	630.71	653.10	659.70	668.24	691.63	739.42	694.31	644.22	620.05	622.31	719.68
United Kingdom	1815.54	1207.34	870.50	680.02	544.35	457.25	376.19	306.94	264.08	242.50	234.73	241.82	256.08	295.06	327.96	360.77	409.81	441.19	481.01	513.72	520.72
United Republic of Tanzania	21557.90	11762.58	10017.85	11252.71	12614.60	13563.00	14377.50	14757.36	15040.38	15202.85	15005.33	15179.65	15165.32	15050.34	14684.43	14770.16	14597.95	14339.06	14106.01	13794.02	14216.74
United States Virgin Islands	1210.11	1114.08	1101.33	1119.62	1149.78	1157.25	1171.64	1135.92	1084.47	1025.75	977.02	912.62	850.92	788.42	732.21	693.25	652.50	647.78	634.84	669.35	1010.38
United States of America	1125.27	831.39	556.75	434.65	393.94	355.96	325.19	298.34	280.44	257.48	246.31	232.21	219.18	211.21	195.75	184.69	174.82	167.42	168.19	177.24	382.79

Uruguay	10023.53	8015.49	6839.89	6162.89	5701.20	5184.21	4458.29	3738.44	3019.04	2491.82	2062.44	1690.51	1563.51	1450.67	1376.15	1340.33	1274.34	1247.32	1262.11	1277.63	4329.82
Uzbekistan	4671.41	3348.07	2645.68	2611.17	2729.39	2742.27	2798.13	2782.23	2770.44	2580.46	2502.98	2461.20	2443.26	2433.08	2363.07	2317.90	2247.32	2240.37	2204.51	2195.67	2947.55
Vanuatu	44733.26	34881.15	33083.28	30672.33	27697.04	24788.70	21240.99	16920.81	12142.96	8669.26	6319.31	5089.80	4935.13	4764.28	4594.41	4471.12	4437.17	4351.96	4408.07	4368.56	25875.86
Venezuela (Bolivarian Republic of)	7956.99	8254.06	4391.19	4790.14	4740.14	4756.18	4698.80	4774.98	4735.69	4829.65	4958.10	5025.78	5045.85	5341.52	5100.00	5068.78	4642.29	4470.96	4389.61	4271.76	5344.92
Viet Nam	8559.85	4834.89	2183.97	1453.81	799.20	386.59	163.06	72.89	34.67	23.52	18.53	16.98	17.65	18.65	19.60	20.81	22.21	24.23	26.47	27.38	1369.66
Yemen	28193.53	14527.61	13039.01	12711.94	12443.49	12761.68	12672.16	12443.51	12028.96	12162.78	11938.87	11677.31	11466.88	11640.23	11545.90	11570.36	11418.87	11318.47	11115.45	10412.34	15006.81
Zambia	22306.96	11657.75	10812.53	11599.77	12389.70	13054.83	13495.89	13699.10	14063.78	14014.36	14105.61	13553.75	13134.27	13173.75	12722.81	12653.50	12528.33	12424.88	11911.41	11232.56	13947.08
Zimbabwe	31933.59	20716.38	18381.41	20000.62	18992.36	17931.22	16752.63	15328.62	13852.29	12102.65	11158.08	10658.39	9945.94	9526.65	9059.32	9210.09	9176.34	9617.79	9957.01	10569.23	19203.11

Abbreviations: VAD, vitamin A deficiency.

Supplemental Table S6: Age distribution of DALY rate (per 100,000 people) for VAD in different countries in 2019.

**country**	**<5**	**5 to 9**	**10 to 14**	**15 to 19**	**20 to 24**	**25 to 29**	**30 to 34**	**35 to 39**	**40 to 44**	**45 to 49**	**50 to 54**	**55 to 59**	**60 to 64**	**65 to 69**	**70 to 74**	**75 to 79**	**80 to 84**	**85 to 89**	**90 to 94**	**95 +**	**All Ages**
Afghanistan	159.14	79.04	34.24	4.51	4.00	3.55	3.15	2.70	2.38	2.31	2.45	2.63	2.70	2.74	2.54	2.43	2.15	1.90	1.57	1.27	45.69
Albania	39.66	26.19	15.92	0.00	0.00	0.00	0.00	0.00	0.00	0.00	0.00	0.00	0.00	0.00	0.00	0.00	0.00	0.00	0.00	0.00	4.77
Algeria	23.22	16.73	9.24	5.32	4.70	4.41	3.81	3.35	2.83	2.71	2.70	2.90	3.00	3.11	3.00	2.67	2.28	1.89	1.50	1.17	7.35
American Samoa	30.18	14.84	4.43	0.00	0.00	0.00	0.00	0.00	0.00	0.00	0.00	0.00	0.00	0.00	0.00	0.00	0.00	0.00	0.00	0.00	4.75
Andorra	0.77	0.57	0.10	0.00	0.00	0.00	0.00	0.00	0.00	0.00	0.00	0.00	0.00	0.00	0.00	0.00	0.00	0.00	0.00	0.00	0.06
Angola	132.87	64.23	39.88	8.44	7.87	7.87	7.96	8.33	8.62	9.25	9.78	10.12	10.28	10.17	9.92	9.72	9.22	8.38	6.78	5.53	42.87
Antigua and Barbuda	15.71	14.03	8.49	4.11	3.29	2.94	2.60	2.34	2.08	1.95	1.82	1.72	1.56	1.51	1.59	1.66	1.52	1.27	0.96	0.76	4.33
Argentina	35.58	16.08	4.76	0.00	0.00	0.00	0.00	0.00	0.00	0.00	0.00	0.00	0.00	0.00	0.00	0.00	0.00	0.00	0.00	0.00	4.39
Armenia	1.78	1.09	0.44	0.00	0.00	0.00	0.00	0.00	0.00	0.00	0.00	0.00	0.00	0.00	0.00	0.00	0.00	0.00	0.00	0.00	0.23
Australia	0.05	0.03	0.01	0.00	0.00	0.00	0.00	0.00	0.00	0.00	0.00	0.00	0.00	0.00	0.00	0.00	0.00	0.00	0.00	0.00	0.01
Austria	1.19	0.87	0.13	0.00	0.00	0.00	0.00	0.00	0.00	0.00	0.00	0.00	0.00	0.00	0.00	0.00	0.00	0.00	0.00	0.00	0.11
Azerbaijan	9.15	6.39	2.94	0.00	0.00	0.00	0.00	0.00	0.00	0.00	0.00	0.00	0.00	0.00	0.00	0.00	0.00	0.00	0.00	0.00	1.40
Bahamas	13.14	12.49	8.06	4.27	3.36	3.04	2.65	2.42	2.18	2.03	1.90	1.80	1.62	1.58	1.65	1.72	1.59	1.33	1.01	0.79	4.30
Bahrain	6.12	4.48	1.77	0.00	0.00	0.00	0.00	0.00	0.00	0.00	0.00	0.00	0.00	0.00	0.00	0.00	0.00	0.00	0.00	0.00	0.65
Bangladesh	41.01	27.66	9.21	2.64	2.43	2.46	2.48	2.62	2.71	2.86	2.95	2.97	2.80	2.71	2.67	2.58	2.47	2.31	1.97	1.65	8.93
Barbados	9.96	12.31	7.53	2.81	2.16	1.85	1.56	1.30	1.09	0.97	0.88	0.82	0.74	0.73	0.76	0.82	0.77	0.65	0.50	0.40	2.70
Belarus	1.66	0.81	0.39	0.00	0.00	0.00	0.00	0.00	0.00	0.00	0.00	0.00	0.00	0.00	0.00	0.00	0.00	0.00	0.00	0.00	0.17
Belgium	1.11	0.73	0.11	0.00	0.00	0.00	0.00	0.00	0.00	0.00	0.00	0.00	0.00	0.00	0.00	0.00	0.00	0.00	0.00	0.00	0.11
Belize	29.09	24.28	14.68	4.00	3.20	2.86	2.53	2.29	2.08	1.96	1.84	1.73	1.55	1.51	1.58	1.68	1.55	1.30	1.00	0.78	8.48
Benin	226.52	203.35	101.36	5.59	4.93	4.59	4.19	3.95	3.78	3.91	4.13	4.40	4.57	4.53	4.39	4.02	3.62	3.29	2.67	2.13	86.15
Bermuda	2.66	2.50	0.99	0.00	0.00	0.00	0.00	0.00	0.00	0.00	0.00	0.00	0.00	0.00	0.00	0.00	0.00	0.00	0.00	0.00	0.27

Bhutan	81.22	45.22	29.86	1.76	1.56	1.58	1.60	1.66	1.70	1.86	2.03	2.21	2.26	2.32	2.34	2.29	2.15	1.99	1.59	1.21	14.75
Bolivia (Plurinational State of)	51.19	36.02	20.26	4.73	3.73	3.37	3.01	2.76	2.56	2.54	2.51	2.43	2.24	2.08	2.00	1.95	1.79	1.58	1.30	1.05	14.31
Bosnia and Herzegovina	23.02	15.83	8.39	0.00	0.00	0.00	0.00	0.00	0.00	0.00	0.00	0.00	0.00	0.00	0.00	0.00	0.00	0.00	0.00	0.00	2.23
Botswana	68.79	65.70	30.50	6.11	5.46	5.36	5.29	5.27	5.18	5.20	5.17	5.34	5.25	5.11	4.74	4.21	3.72	3.35	2.85	2.79	20.42
Brazil	35.61	32.80	18.44	4.50	3.42	2.97	2.57	2.32	2.15	2.03	1.96	1.91	1.81	1.80	1.87	1.90	1.79	1.60	1.36	1.16	8.34
Brunei Darussalam	4.30	2.64	1.66	0.00	0.00	0.00	0.00	0.00	0.00	0.00	0.00	0.00	0.00	0.00	0.00	0.00	0.00	0.00	0.00	0.00	0.62
Bulgaria	22.69	15.12	7.95	0.00	0.00	0.00	0.00	0.00	0.00	0.00	0.00	0.00	0.00	0.00	0.00	0.00	0.00	0.00	0.00	0.00	2.15
Burkina Faso	269.11	237.22	157.32	5.25	5.04	4.76	4.10	3.73	3.56	3.78	3.99	4.21	4.26	4.15	3.90	3.53	3.18	2.97	2.45	1.98	106.53
Burundi	157.52	86.92	46.62	3.14	2.81	2.82	2.74	2.78	2.87	3.06	3.23	3.22	3.15	3.17	3.14	3.09	2.82	2.49	1.71	1.21	48.07
Cape Verde	40.61	42.38	19.90	3.78	3.30	2.99	2.71	2.42	2.30	2.47	2.66	2.83	2.80	2.74	2.61	2.37	2.11	1.88	1.57	1.38	11.75
Cambodia	97.11	67.36	25.93	1.29	1.03	0.97	0.96	1.03	1.12	1.26	1.32	1.34	1.29	1.25	1.19	1.09	0.98	0.90	0.78	0.67	20.42
Cameroon	136.74	158.94	94.36	4.28	3.73	3.27	2.77	2.36	2.08	2.13	2.26	2.51	2.70	2.84	2.88	2.73	2.50	2.18	1.76	1.40	56.02
Canada	1.03	0.61	0.29	0.00	0.00	0.00	0.00	0.00	0.00	0.00	0.00	0.00	0.00	0.00	0.00	0.00	0.00	0.00	0.00	0.00	0.11
Central African Republic	358.44	215.04	119.46	11.87	11.06	9.53	7.45	5.92	4.71	4.17	4.38	4.71	5.58	6.30	7.15	7.95	8.56	7.90	6.77	5.20	106.24
Chad	302.21	208.96	135.99	9.46	8.61	7.57	6.14	5.18	4.93	5.05	5.51	5.96	6.52	6.89	6.76	6.29	5.94	5.13	4.01	3.10	118.50
Chile	2.01	1.78	0.59	0.00	0.00	0.00	0.00	0.00	0.00	0.00	0.00	0.00	0.00	0.00	0.00	0.00	0.00	0.00	0.00	0.00	0.29
China	12.16	7.39	4.57	2.66	2.13	1.91	1.82	1.83	1.84	1.73	1.68	1.57	1.55	1.75	1.81	1.64	1.41	1.18	0.95	0.84	2.83
Colombia	12.64	8.20	5.10	3.37	2.87	2.85	2.67	2.49	2.30	2.24	2.20	2.14	2.02	2.00	2.04	2.12	2.03	1.79	1.37	1.08	3.94
Comoros	118.31	73.10	55.56	4.01	3.73	3.76	3.83	4.10	4.30	4.61	4.70	4.78	4.69	4.63	4.32	4.04	3.56	3.11	2.26	1.63	29.65
Congo	220.01	181.17	98.71	9.06	8.27	8.21	8.30	8.89	9.49	10.63	11.54	12.10	11.89	11.82	11.64	11.63	10.95	10.06	7.97	6.26	69.76
Cook Islands	24.41	16.11	6.46	2.37	2.27	2.30	2.31	2.34	2.39	2.48	2.55	2.48	2.27	2.10	1.90	1.69	1.43	1.24	1.10	1.06	5.34
Costa Rica	11.20	9.92	5.19	3.02	2.58	2.48	2.29	2.14	1.96	1.87	1.84	1.78	1.67	1.60	1.57	1.55	1.46	1.33	1.12	0.94	3.62
Cote d'Ivoire	170.85	132.11	65.28	5.44	4.93	4.58	4.17	3.97	3.94	4.16	4.42	4.68	4.87	4.72	4.48	4.11	3.63	3.32	2.66	2.11	54.69
Croatia	13.85	6.40	2.16	0.00	0.00	0.00	0.00	0.00	0.00	0.00	0.00	0.00	0.00	0.00	0.00	0.00	0.00	0.00	0.00	0.00	1.03

Cuba	6.50	5.93	2.49	0.00	0.00	0.00	0.00	0.00	0.00	0.00	0.00	0.00	0.00	0.00	0.00	0.00	0.00	0.00	0.00	0.00	0.79
Cyprus	1.39	1.06	0.17	0.00	0.00	0.00	0.00	0.00	0.00	0.00	0.00	0.00	0.00	0.00	0.00	0.00	0.00	0.00	0.00	0.00	0.14
Czechia	10.11	6.61	3.22	0.00	0.00	0.00	0.00	0.00	0.00	0.00	0.00	0.00	0.00	0.00	0.00	0.00	0.00	0.00	0.00	0.00	1.06
Democratic People's Republic of Korea	40.85	34.69	12.39	0.55	0.37	0.31	0.30	0.32	0.35	0.43	0.51	0.59	0.65	0.69	0.75	0.76	0.70	0.65	0.54	0.44	5.84
Democratic Republic of the Congo	257.55	169.16	105.69	7.55	6.73	6.26	5.56	5.21	5.24	5.53	5.93	6.14	6.07	6.44	6.87	7.47	7.68	6.82	5.81	4.22	81.87
Denmark	0.96	0.77	0.14	0.00	0.00	0.00	0.00	0.00	0.00	0.00	0.00	0.00	0.00	0.00	0.00	0.00	0.00	0.00	0.00	0.00	0.10
Djibouti	105.40	63.62	42.55	3.63	3.41	3.36	3.47	3.55	3.72	4.00	4.16	4.19	4.07	4.03	3.79	3.39	2.94	2.57	1.85	1.33	28.00
Dominica	14.15	11.94	8.36	4.08	3.29	2.92	2.58	2.33	2.13	2.02	1.89	1.79	1.65	1.57	1.64	1.73	1.60	1.34	1.03	0.79	4.28
Dominican Republic	21.71	16.98	11.47	4.24	3.37	3.06	2.75	2.58	2.40	2.34	2.23	2.12	1.94	1.89	2.01	2.22	2.08	1.76	1.30	0.98	6.75
Ecuador	15.36	12.48	7.78	4.22	3.36	3.09	2.74	2.47	2.21	2.10	2.07	2.01	1.81	1.64	1.58	1.53	1.39	1.19	0.96	0.77	5.33
Egypt	11.97	11.67	5.39	2.12	1.85	1.74	1.55	1.35	1.15	1.08	1.07	1.18	1.24	1.27	1.24	1.10	0.94	0.79	0.62	0.47	4.24
El Salvador	23.71	25.09	9.57	3.89	3.39	3.30	3.15	3.00	2.81	2.75	2.65	2.50	2.30	2.21	2.30	2.48	2.41	2.16	1.61	1.22	7.54
Equatorial Guinea	44.85	41.39	30.70	14.94	14.26	13.31	12.39	12.30	12.10	11.43	11.92	13.04	13.69	13.98	13.73	13.00	12.15	10.92	9.20	7.07	23.54
Eritrea	146.98	85.44	63.77	4.23	4.06	4.21	4.30	4.60	4.75	5.02	5.16	5.21	4.93	4.71	4.50	4.27	3.88	3.49	2.44	1.58	42.17
Estonia	0.86	0.37	0.18	0.00	0.00	0.00	0.00	0.00	0.00	0.00	0.00	0.00	0.00	0.00	0.00	0.00	0.00	0.00	0.00	0.00	0.08
Eswatini	68.39	43.47	23.10	6.40	5.74	5.52	5.43	5.49	5.33	5.47	5.63	5.89	5.72	5.50	5.17	4.74	4.27	3.85	3.49	2.96	19.94
Ethiopia	193.70	115.65	60.30	13.23	10.30	6.55	4.82	3.96	3.61	3.55	3.58	3.41	3.25	3.06	2.73	2.49	2.14	1.90	1.64	1.40	58.49
Fiji	46.22	44.89	23.62	2.45	2.36	2.39	2.42	2.45	2.54	2.69	2.75	2.69	2.54	2.40	2.28	2.02	1.69	1.36	1.14	1.01	12.98
Finland	1.23	0.71	0.07	0.00	0.00	0.00	0.00	0.00	0.00	0.00	0.00	0.00	0.00	0.00	0.00	0.00	0.00	0.00	0.00	0.00	0.10
France	0.32	0.19	0.03	0.00	0.00	0.00	0.00	0.00	0.00	0.00	0.00	0.00	0.00	0.00	0.00	0.00	0.00	0.00	0.00	0.00	0.03
Gabon	44.43	40.96	27.16	6.81	6.22	6.37	6.38	6.83	7.10	7.86	8.45	8.98	9.14	9.25	8.92	8.84	8.38	7.74	6.46	5.38	17.33
Gambia	223.07	185.43	114.32	4.99	4.46	3.90	3.31	3.10	2.97	3.18	3.49	3.74	3.82	3.75	3.56	3.22	2.84	2.52	2.11	1.79	75.05
Georgia	9.44	6.50	2.82	0.00	0.00	0.00	0.00	0.00	0.00	0.00	0.00	0.00	0.00	0.00	0.00	0.00	0.00	0.00	0.00	0.00	1.26
Germany	0.38	0.44	0.16	0.00	0.00	0.00	0.00	0.00	0.00	0.00	0.00	0.00	0.00	0.00	0.00	0.00	0.00	0.00	0.00	0.00	0.04

Ghana	156.32	124.30	59.36	5.09	4.50	4.26	3.91	3.72	3.56	3.84	4.18	4.54	4.64	4.63	4.43	4.02	3.51	3.09	2.48	1.97	43.99
Greece	1.98	1.54	0.26	0.00	0.00	0.00	0.00	0.00	0.00	0.00	0.00	0.00	0.00	0.00	0.00	0.00	0.00	0.00	0.00	0.00	0.17
Greenland	2.19	2.12	0.99	0.00	0.00	0.00	0.00	0.00	0.00	0.00	0.00	0.00	0.00	0.00	0.00	0.00	0.00	0.00	0.00	0.00	0.37
Grenada	24.68	21.14	10.59	3.99	3.22	2.90	2.55	2.31	2.06	1.96	1.83	1.72	1.56	1.51	1.59	1.66	1.51	1.25	0.96	0.74	5.90
Guam	16.28	11.67	3.85	0.00	0.00	0.00	0.00	0.00	0.00	0.00	0.00	0.00	0.00	0.00	0.00	0.00	0.00	0.00	0.00	0.00	2.88
Guatemala	40.07	21.82	11.30	4.18	3.61	3.54	3.38	3.15	2.86	2.74	2.54	2.34	2.04	1.90	1.99	2.21	2.18	1.99	1.52	1.11	10.25
Guinea	233.65	172.26	93.12	5.89	5.29	4.77	4.33	4.05	4.02	4.18	4.46	4.74	4.84	4.74	4.50	4.06	3.65	3.11	2.77	2.16	80.65
Guinea-Bissau	235.31	200.10	108.58	5.66	5.18	4.94	4.67	4.51	4.45	4.79	4.95	5.18	5.06	4.90	4.82	4.51	4.36	3.89	3.14	2.34	80.41
Guyana	29.16	30.56	18.37	4.06	3.28	2.95	2.62	2.42	2.20	2.11	1.98	1.85	1.65	1.59	1.67	1.77	1.63	1.38	1.08	0.87	8.98
Haiti	115.33	83.48	49.48	4.73	3.87	3.48	3.20	3.04	2.88	2.80	2.63	2.38	2.07	1.93	2.04	2.15	1.98	1.69	1.33	1.03	31.20
Honduras	26.45	20.05	9.24	2.98	2.61	2.54	2.39	2.26	2.14	2.11	2.04	1.97	1.81	1.76	1.87	2.03	1.96	1.79	1.35	1.07	7.88
Hungary	14.23	9.44	4.37	0.00	0.00	0.00	0.00	0.00	0.00	0.00	0.00	0.00	0.00	0.00	0.00	0.00	0.00	0.00	0.00	0.00	1.31
Iceland	0.85	0.58	0.10	0.00	0.00	0.00	0.00	0.00	0.00	0.00	0.00	0.00	0.00	0.00	0.00	0.00	0.00	0.00	0.00	0.00	0.10
India	98.84	53.68	35.36	6.58	6.05	6.29	6.40	6.50	6.54	6.90	7.30	7.66	7.47	7.19	6.89	6.41	5.70	4.89	4.00	3.24	21.39
Indonesia	51.57	36.78	23.13	12.23	11.15	10.93	10.20	9.36	8.83	8.88	8.89	8.53	7.80	7.43	7.19	6.57	5.80	5.20	4.19	3.36	16.39
Iran (Islamic Republic of)	4.46	2.56	0.73	0.00	0.00	0.00	0.00	0.00	0.00	0.00	0.00	0.00	0.00	0.00	0.00	0.00	0.00	0.00	0.00	0.00	0.64
Iraq	24.97	16.34	7.65	5.13	4.52	4.30	3.78	3.26	2.90	2.79	2.78	3.03	3.14	3.27	3.19	2.84	2.42	1.98	1.53	1.22	7.96
Ireland	0.86	0.44	0.06	0.00	0.00	0.00	0.00	0.00	0.00	0.00	0.00	0.00	0.00	0.00	0.00	0.00	0.00	0.00	0.00	0.00	0.09
Israel	11.10	10.05	1.56	0.00	0.00	0.00	0.00	0.00	0.00	0.00	0.00	0.00	0.00	0.00	0.00	0.00	0.00	0.00	0.00	0.00	2.19
Italy	1.91	1.58	0.28	0.00	0.00	0.00	0.00	0.00	0.00	0.00	0.00	0.00	0.00	0.00	0.00	0.00	0.00	0.00	0.00	0.00	0.16
Jamaica	18.95	14.92	8.56	3.97	3.15	2.81	2.45	2.22	1.95	1.85	1.71	1.64	1.49	1.46	1.54	1.64	1.49	1.25	0.96	0.75	4.89
Japan	1.69	1.28	0.79	0.00	0.00	0.00	0.00	0.00	0.00	0.00	0.00	0.00	0.00	0.00	0.00	0.00	0.00	0.00	0.00	0.00	0.15
Jordan	22.67	17.81	10.35	4.10	3.67	3.51	3.24	2.89	2.58	2.45	2.51	2.76	2.91	3.20	3.18	3.08	2.76	2.25	1.65	1.19	7.58
Kazakhstan	36.96	23.41	9.46	0.00	0.00	0.00	0.00	0.00	0.00	0.00	0.00	0.00	0.00	0.00	0.00	0.00	0.00	0.00	0.00	0.00	6.70

Kenya	170.08	106.13	52.09	5.66	5.18	4.73	4.42	4.32	4.27	4.31	4.36	4.18	3.96	3.73	3.43	3.04	2.69	2.49	2.00	1.50	44.77
Kiribati	175.74	113.77	53.83	2.51	2.43	2.38	2.31	2.25	2.20	2.27	2.31	2.26	2.11	1.94	1.76	1.54	1.33	1.12	0.99	0.94	42.75
Kuwait	10.43	9.71	6.68	4.91	4.33	4.00	3.59	3.08	2.77	2.60	2.63	2.87	2.98	3.11	3.06	2.69	2.27	1.78	1.35	1.04	4.45
Kyrgyzstan	31.28	24.64	12.74	4.99	4.07	3.99	3.68	3.59	3.40	3.62	3.60	3.45	3.15	2.96	2.89	2.84	2.51	2.17	1.76	1.43	10.12
Lao People's Democratic Republic	80.20	46.70	24.06	1.32	1.06	1.01	1.00	1.08	1.17	1.34	1.39	1.37	1.27	1.25	1.31	1.33	1.23	1.08	0.80	0.54	16.98
Latvia	1.07	0.50	0.25	0.00	0.00	0.00	0.00	0.00	0.00	0.00	0.00	0.00	0.00	0.00	0.00	0.00	0.00	0.00	0.00	0.00	0.10
Lebanon	5.77	3.94	1.07	0.00	0.00	0.00	0.00	0.00	0.00	0.00	0.00	0.00	0.00	0.00	0.00	0.00	0.00	0.00	0.00	0.00	1.01
Lesotho	135.38	87.87	42.46	7.08	6.37	6.21	6.16	6.16	6.04	6.14	6.26	6.39	6.35	6.10	5.70	5.29	4.77	4.23	3.61	3.00	32.18
Liberia	125.68	84.23	49.00	5.26	4.70	4.33	3.78	3.58	3.31	3.25	3.28	3.63	3.87	4.03	3.97	3.74	3.50	3.12	2.52	2.00	36.38
Libya	19.88	14.55	9.05	5.80	5.19	4.90	4.33	3.65	3.14	2.94	3.01	3.21	3.28	3.33	3.16	2.82	2.45	2.04	1.63	1.37	6.21
Lithuania	0.99	0.46	0.24	0.00	0.00	0.00	0.00	0.00	0.00	0.00	0.00	0.00	0.00	0.00	0.00	0.00	0.00	0.00	0.00	0.00	0.09
Luxembourg	0.78	0.57	0.09	0.00	0.00	0.00	0.00	0.00	0.00	0.00	0.00	0.00	0.00	0.00	0.00	0.00	0.00	0.00	0.00	0.00	0.08
Madagascar	146.44	89.72	50.53	3.51	3.30	3.30	3.45	3.60	3.77	4.02	4.09	4.08	3.95	3.96	3.86	3.63	3.46	3.10	2.17	1.50	42.34
Malawi	183.03	124.84	87.06	4.10	3.81	3.92	4.07	4.33	4.54	4.68	4.86	4.93	4.71	4.56	4.30	4.16	3.81	3.30	2.27	1.55	58.53
Malaysia	3.03	1.28	0.58	0.00	0.00	0.00	0.00	0.00	0.00	0.00	0.00	0.00	0.00	0.00	0.00	0.00	0.00	0.00	0.00	0.00	0.40
Maldives	36.58	20.04	8.18	1.08	0.81	0.71	0.69	0.77	0.87	1.05	1.13	1.14	1.08	1.08	1.16	1.19	1.11	1.00	0.71	0.47	5.71
Mali	317.75	245.85	157.68	7.71	6.67	5.60	4.58	4.21	4.07	4.37	4.74	5.05	5.09	4.74	4.39	3.87	3.43	3.07	2.39	1.84	120.60
Malta	1.88	1.43	0.24	0.00	0.00	0.00	0.00	0.00	0.00	0.00	0.00	0.00	0.00	0.00	0.00	0.00	0.00	0.00	0.00	0.00	0.17
Marshall Islands	155.14	93.84	32.99	2.23	2.10	2.12	2.13	2.13	2.14	2.23	2.31	2.28	2.12	1.95	1.78	1.54	1.34	1.11	0.97	0.92	31.95
Mauritania	113.22	83.27	43.16	5.15	4.57	4.31	3.96	3.63	3.58	3.86	4.19	4.36	4.54	4.47	4.27	3.88	3.46	3.06	2.47	1.96	34.73
Mauritius	20.02	11.46	4.11	1.45	1.10	1.01	0.98	1.06	1.15	1.31	1.38	1.39	1.33	1.30	1.24	1.14	0.99	0.88	0.70	0.52	2.90
Mexico	30.93	24.11	12.19	6.23	5.17	4.92	4.49	4.05	3.60	3.36	3.23	3.20	3.04	2.98	2.94	2.91	2.66	2.32	1.88	1.54	8.86
Micronesia (Federated States of)	179.38	109.68	41.11	2.06	1.95	1.98	1.98	1.99	1.99	2.11	2.19	2.23	2.08	1.93	1.75	1.52	1.29	1.08	0.95	0.89	34.25
Monaco	0.57	0.40	0.07	0.00	0.00	0.00	0.00	0.00	0.00	0.00	0.00	0.00	0.00	0.00	0.00	0.00	0.00	0.00	0.00	0.00	0.05

Mongolia	14.32	11.37	7.50	4.67	3.92	3.81	3.78	3.91	3.93	4.22	4.24	4.09	3.82	3.60	3.66	3.86	3.55	3.06	2.11	1.56	6.24
Montenegro	14.58	9.06	4.71	0.00	0.00	0.00	0.00	0.00	0.00	0.00	0.00	0.00	0.00	0.00	0.00	0.00	0.00	0.00	0.00	0.00	1.63
Morocco	38.32	28.85	14.89	4.47	3.94	3.68	3.22	2.65	2.31	2.10	2.11	2.26	2.28	2.34	2.23	1.94	1.64	1.32	1.07	0.88	9.33
Mozambique	239.82	149.67	80.96	2.49	2.26	2.22	2.19	2.24	2.24	2.33	2.36	2.38	2.28	2.19	2.09	1.90	1.69	1.52	1.08	0.72	77.41
Myanmar	81.47	47.89	16.75	0.30	0.25	0.24	0.24	0.26	0.27	0.30	0.31	0.31	0.29	0.29	0.28	0.27	0.25	0.23	0.21	0.19	13.55
Namibia	54.24	40.66	21.44	5.59	4.89	4.86	4.76	4.81	4.74	4.91	5.11	5.42	5.37	5.19	4.88	4.57	4.11	3.61	3.12	2.70	17.03
Nauru	65.00	44.02	17.74	1.83	1.70	1.72	1.73	1.72	1.72	1.82	1.93	1.97	1.85	1.71	1.55	1.37	1.21	0.99	0.83	0.77	17.21
Nepal	50.22	28.29	15.20	2.11	1.91	1.96	2.02	2.19	2.35	2.57	2.72	2.75	2.65	2.63	2.61	2.47	2.29	2.08	1.54	1.07	10.92
Netherlands	0.96	0.44	0.05	0.00	0.00	0.00	0.00	0.00	0.00	0.00	0.00	0.00	0.00	0.00	0.00	0.00	0.00	0.00	0.00	0.00	0.08
New Zealand	1.63	0.99	0.17	0.00	0.00	0.00	0.00	0.00	0.00	0.00	0.00	0.00	0.00	0.00	0.00	0.00	0.00	0.00	0.00	0.00	0.18
Nicaragua	8.53	7.34	4.95	3.31	2.88	2.79	2.62	2.44	2.30	2.22	2.14	2.09	1.94	1.87	1.93	2.02	1.94	1.71	1.31	1.03	3.91
Niger	375.41	326.51	189.49	5.91	5.29	4.64	3.97	3.63	3.50	3.62	3.86	4.11	4.29	4.26	4.12	3.83	3.35	2.97	2.71	2.16	162.14
Nigeria	68.70	43.06	29.77	10.01	8.29	6.76	5.17	4.00	3.53	3.48	3.68	3.85	3.89	3.76	3.53	3.15	2.80	2.49	2.14	1.88	24.53
Niue	35.28	23.49	8.93	1.66	1.52	1.54	1.54	1.53	1.52	1.65	1.78	1.86	1.79	1.68	1.51	1.30	1.10	0.90	0.77	0.70	6.53
North Macedonia	33.82	21.66	10.31	0.00	0.00	0.00	0.00	0.00	0.00	0.00	0.00	0.00	0.00	0.00	0.00	0.00	0.00	0.00	0.00	0.00	3.51
Northern Mariana Islands	22.66	14.85	4.63	0.00	0.00	0.00	0.00	0.00	0.00	0.00	0.00	0.00	0.00	0.00	0.00	0.00	0.00	0.00	0.00	0.00	2.32
Norway	0.90	0.61	0.09	0.00	0.00	0.00	0.00	0.00	0.00	0.00	0.00	0.00	0.00	0.00	0.00	0.00	0.00	0.00	0.00	0.00	0.09
Oman	37.74	23.46	13.86	10.46	9.45	8.78	7.80	6.47	5.33	4.73	4.77	4.93	5.01	4.88	4.63	3.90	3.29	2.59	1.89	1.24	11.61
Pakistan	49.54	32.84	21.81	10.16	9.22	8.70	8.28	8.18	8.26	8.56	8.81	8.31	7.55	7.12	6.80	6.41	5.93	5.30	4.25	3.44	19.03
Palau	35.20	23.20	8.39	1.66	1.52	1.55	1.56	1.53	1.55	1.65	1.77	1.85	1.78	1.66	1.52	1.30	1.10	0.91	0.77	0.71	5.30
Palestine	30.83	26.22	13.38	6.66	5.94	5.43	4.66	3.67	2.86	2.71	2.69	2.89	2.95	3.03	2.85	2.47	2.06	1.69	1.35	1.14	11.75
Panama	14.78	10.51	6.85	3.80	3.17	3.02	2.82	2.65	2.44	2.37	2.36	2.29	2.12	2.00	1.97	1.95	1.86	1.70	1.42	1.16	4.92
Papua New Guinea	84.58	48.45	28.98	2.39	2.22	2.21	2.06	1.85	1.72	1.81	1.94	1.98	1.88	1.72	1.49	1.25	1.04	0.88	0.74	0.66	22.61
Paraguay	27.78	25.27	15.19	6.27	4.72	3.88	3.38	3.04	2.75	2.60	2.49	2.46	2.37	2.41	2.51	2.59	2.41	2.04	1.53	1.19	9.03

Peru	31.28	30.76	14.02	5.51	4.22	3.73	3.39	3.00	2.80	2.74	2.76	2.75	2.56	2.45	2.42	2.36	2.17	1.87	1.49	1.23	9.34
Philippines	66.69	35.83	11.15	3.39	2.68	2.54	2.53	2.68	2.82	3.08	3.29	3.45	3.37	3.31	3.17	2.92	2.57	2.25	1.89	1.60	14.40
Poland	13.62	8.97	4.87	0.00	0.00	0.00	0.00	0.00	0.00	0.00	0.00	0.00	0.00	0.00	0.00	0.00	0.00	0.00	0.00	0.00	1.39
Portugal	2.66	2.12	0.34	0.00	0.00	0.00	0.00	0.00	0.00	0.00	0.00	0.00	0.00	0.00	0.00	0.00	0.00	0.00	0.00	0.00	0.21
Puerto Rico	2.80	2.54	1.13	0.00	0.00	0.00	0.00	0.00	0.00	0.00	0.00	0.00	0.00	0.00	0.00	0.00	0.00	0.00	0.00	0.00	0.30
Qatar	2.15	1.18	0.61	0.00	0.00	0.00	0.00	0.00	0.00	0.00	0.00	0.00	0.00	0.00	0.00	0.00	0.00	0.00	0.00	0.00	0.19
Republic of Korea	1.72	1.20	0.49	0.00	0.00	0.00	0.00	0.00	0.00	0.00	0.00	0.00	0.00	0.00	0.00	0.00	0.00	0.00	0.00	0.00	0.14
Republic of Moldova	3.40	2.72	1.33	0.00	0.00	0.00	0.00	0.00	0.00	0.00	0.00	0.00	0.00	0.00	0.00	0.00	0.00	0.00	0.00	0.00	0.38
Romania	23.39	12.28	5.32	0.00	0.00	0.00	0.00	0.00	0.00	0.00	0.00	0.00	0.00	0.00	0.00	0.00	0.00	0.00	0.00	0.00	2.08
Russian Federation	0.25	0.11	0.04	0.00	0.00	0.00	0.00	0.00	0.00	0.00	0.00	0.00	0.00	0.00	0.00	0.00	0.00	0.00	0.00	0.00	0.03
Rwanda	74.50	48.77	23.93	3.06	2.69	2.81	2.79	2.96	3.15	3.31	3.47	3.55	3.48	3.47	3.41	3.24	3.08	2.73	1.87	1.28	20.76
Saint Kitts and Nevis	14.23	13.34	8.28	4.15	3.33	2.98	2.59	2.34	2.07	1.95	1.80	1.69	1.53	1.48	1.56	1.64	1.48	1.22	0.92	0.70	4.25
Saint Lucia	18.81	18.22	10.79	4.05	3.23	2.87	2.52	2.26	2.03	1.93	1.80	1.69	1.55	1.48	1.57	1.65	1.51	1.26	0.96	0.74	4.75
Saint Vincent and the Grenadines	24.72	22.55	13.10	4.09	3.26	2.89	2.55	2.31	2.09	1.96	1.82	1.71	1.55	1.51	1.61	1.70	1.56	1.33	1.00	0.77	6.25
Samoa	86.61	70.25	28.95	1.84	1.71	1.74	1.74	1.73	1.73	1.83	1.96	1.99	1.88	1.73	1.57	1.38	1.15	0.98	0.83	0.77	22.09
San Marino	0.84	0.61	0.10	0.00	0.00	0.00	0.00	0.00	0.00	0.00	0.00	0.00	0.00	0.00	0.00	0.00	0.00	0.00	0.00	0.00	0.08
Sao Tome and Principe	106.19	121.42	52.25	5.28	4.70	4.44	4.09	3.83	3.75	4.01	4.31	4.59	4.57	4.59	4.49	4.10	3.60	3.17	2.57	2.04	36.40
Saudi Arabia	0.90	0.42	0.07	0.00	0.00	0.00	0.00	0.00	0.00	0.00	0.00	0.00	0.00	0.00	0.00	0.00	0.00	0.00	0.00	0.00	0.09
Senegal	99.80	106.53	73.65	4.87	4.40	4.24	3.97	3.91	3.96	4.28	4.55	4.85	4.86	4.93	4.84	4.40	3.98	3.62	2.83	1.99	40.47
Serbia	41.02	24.73	13.12	0.00	0.00	0.00	0.00	0.00	0.00	0.00	0.00	0.00	0.00	0.00	0.00	0.00	0.00	0.00	0.00	0.00	4.30
Seychelles	14.64	9.84	3.97	1.53	1.17	1.05	1.02	1.12	1.22	1.40	1.48	1.49	1.42	1.38	1.32	1.20	1.02	0.91	0.72	0.55	3.05
Sierra Leone	255.46	202.61	113.35	5.07	4.59	4.36	3.94	3.52	3.13	2.97	2.93	3.10	3.27	3.68	3.96	4.10	4.15	3.71	2.85	2.11	81.15
Singapore	1.43	0.92	0.58	0.00	0.00	0.00	0.00	0.00	0.00	0.00	0.00	0.00	0.00	0.00	0.00	0.00	0.00	0.00	0.00	0.00	0.14
Slovakia	13.03	8.20	4.22	0.00	0.00	0.00	0.00	0.00	0.00	0.00	0.00	0.00	0.00	0.00	0.00	0.00	0.00	0.00	0.00	0.00	1.33

Slovenia	8.52	5.27	2.50	0.00	0.00	0.00	0.00	0.00	0.00	0.00	0.00	0.00	0.00	0.00	0.00	0.00	0.00	0.00	0.00	0.00	0.81
Solomon Islands	177.76	135.04	64.31	2.03	1.94	1.92	1.88	1.84	1.83	1.88	1.94	1.92	1.81	1.69	1.53	1.33	1.14	0.95	0.84	0.77	52.29
Somalia	344.68	271.28	202.95	4.45	4.08	3.80	3.71	3.73	3.58	3.53	3.61	3.43	3.27	3.22	2.94	2.61	2.42	2.25	1.80	1.37	133.27
South Africa	24.46	25.37	14.87	5.51	4.81	4.55	4.44	4.55	4.64	4.84	5.13	5.44	5.49	5.44	5.21	4.88	4.40	3.98	3.49	3.03	9.42
South Sudan	150.75	92.14	72.70	11.55	6.67	3.24	2.37	2.17	2.08	2.15	2.24	2.34	2.58	2.81	2.86	2.78	2.78	2.53	2.09	1.71	51.02
Spain	1.52	1.05	0.20	0.00	0.00	0.00	0.00	0.00	0.00	0.00	0.00	0.00	0.00	0.00	0.00	0.00	0.00	0.00	0.00	0.00	0.13
Sri Lanka	16.60	14.85	6.92	1.77	1.29	1.16	1.12	1.22	1.36	1.54	1.63	1.64	1.54	1.46	1.39	1.25	1.09	0.91	0.71	0.51	4.00
Sudan	95.31	44.27	22.05	5.80	5.07	4.59	4.04	3.65	3.37	3.22	3.22	3.42	3.43	3.54	3.60	3.46	3.11	2.67	1.97	1.39	23.96
Suriname	28.40	26.38	16.04	5.67	4.69	4.25	3.85	3.52	3.21	3.01	2.76	2.47	2.13	1.96	2.00	2.06	1.88	1.57	1.18	0.92	8.60
Sweden	0.97	0.71	0.11	0.00	0.00	0.00	0.00	0.00	0.00	0.00	0.00	0.00	0.00	0.00	0.00	0.00	0.00	0.00	0.00	0.00	0.11
Switzerland	0.66	0.41	0.07	0.00	0.00	0.00	0.00	0.00	0.00	0.00	0.00	0.00	0.00	0.00	0.00	0.00	0.00	0.00	0.00	0.00	0.06
Syrian Arab Republic	21.96	11.30	4.25	0.00	0.00	0.00	0.00	0.00	0.00	0.00	0.00	0.00	0.00	0.00	0.00	0.00	0.00	0.00	0.00	0.00	3.18
Taiwan (Province of China)	1.85	1.15	0.30	0.00	0.00	0.00	0.00	0.00	0.00	0.00	0.00	0.00	0.00	0.00	0.00	0.00	0.00	0.00	0.00	0.00	0.14
Tajikistan	47.73	32.98	17.04	4.95	4.06	3.88	3.82	3.72	3.70	3.88	3.89	3.77	3.47	3.24	3.18	3.10	2.70	2.31	1.77	1.31	14.18
Thailand	22.14	10.32	5.29	3.33	2.38	1.98	1.82	2.10	2.30	2.32	2.19	2.02	1.74	1.49	1.35	1.22	1.03	0.80	0.58	0.44	3.57
Timor-Leste	115.04	45.42	18.19	1.84	1.62	1.62	1.70	1.84	2.01	2.31	2.44	2.48	2.34	2.30	2.33	2.36	2.21	1.93	1.44	0.99	24.19
Togo	181.62	161.84	95.97	5.49	5.02	4.60	4.21	3.99	3.90	4.14	4.43	4.59	4.82	4.77	4.56	4.17	3.71	3.26	2.64	2.10	61.90
Tokelau	60.37	41.07	16.45	1.78	1.64	1.68	1.68	1.67	1.65	1.81	1.89	1.96	1.88	1.73	1.59	1.36	1.16	0.96	0.83	0.77	14.82
Tonga	60.89	38.64	13.21	1.37	1.25	1.27	1.27	1.29	1.31	1.39	1.46	1.48	1.43	1.37	1.30	1.17	1.02	0.88	0.79	0.76	14.16
Trinidad and Tobago	14.79	14.13	8.63	3.76	2.96	2.65	2.34	2.13	1.92	1.85	1.74	1.66	1.49	1.43	1.50	1.61	1.49	1.25	0.97	0.77	4.16
Tunisia	12.60	8.56	5.81	4.15	3.67	3.36	3.01	2.71	2.34	2.20	2.23	2.38	2.45	2.58	2.49	2.24	1.94	1.63	1.31	1.06	4.23
Turkey	11.57	6.50	2.79	0.00	0.00	0.00	0.00	0.00	0.00	0.00	0.00	0.00	0.00	0.00	0.00	0.00	0.00	0.00	0.00	0.00	1.30
Turkmenistan	24.09	17.66	10.87	6.30	5.07	4.70	4.17	3.55	3.22	3.16	3.09	2.89	2.57	2.25	2.02	1.82	1.56	1.32	1.04	0.84	8.14
Tuvalu	91.78	64.52	27.54	1.91	1.80	1.82	1.82	1.80	1.81	1.91	2.02	2.02	1.90	1.77	1.60	1.39	1.19	0.99	0.85	0.80	18.75

Uganda	94.69	47.02	28.23	3.22	3.11	3.12	3.15	3.18	3.19	3.33	3.38	3.37	3.19	3.13	3.03	2.82	2.58	2.35	1.69	1.23	29.14
Ukraine	1.65	0.79	0.32	0.00	0.00	0.00	0.00	0.00	0.00	0.00	0.00	0.00	0.00	0.00	0.00	0.00	0.00	0.00	0.00	0.00	0.14
United Arab Emirates	2.90	1.96	1.10	0.00	0.00	0.00	0.00	0.00	0.00	0.00	0.00	0.00	0.00	0.00	0.00	0.00	0.00	0.00	0.00	0.00	0.25
United Kingdom	1.86	1.37	0.28	0.00	0.00	0.00	0.00	0.00	0.00	0.00	0.00	0.00	0.00	0.00	0.00	0.00	0.00	0.00	0.00	0.00	0.21
United Republic of Tanzania	113.98	70.96	49.59	6.11	6.05	6.20	6.44	6.56	6.84	7.17	7.09	6.85	6.39	6.09	5.86	5.45	5.02	4.55	3.15	2.36	38.73
United States Virgin Islands	3.30	3.04	1.56	0.00	0.00	0.00	0.00	0.00	0.00	0.00	0.00	0.00	0.00	0.00	0.00	0.00	0.00	0.00	0.00	0.00	0.50
United States of America	0.56	0.71	0.25	0.00	0.00	0.00	0.00	0.00	0.00	0.00	0.00	0.00	0.00	0.00	0.00	0.00	0.00	0.00	0.00	0.00	0.09
Uruguay	28.88	10.56	3.19	0.00	0.00	0.00	0.00	0.00	0.00	0.00	0.00	0.00	0.00	0.00	0.00	0.00	0.00	0.00	0.00	0.00	2.88
Uzbekistan	31.67	21.86	12.73	4.82	3.90	3.75	3.50	3.46	3.36	3.52	3.48	3.37	3.11	2.92	2.84	2.75	2.40	2.08	1.62	1.26	9.44
Vanuatu	157.24	83.74	40.32	1.52	1.38	1.39	1.37	1.35	1.33	1.43	1.54	1.60	1.55	1.45	1.31	1.15	0.97	0.78	0.67	0.61	36.68
Venezuela (Bolivarian Republic of)	19.86	12.41	6.12	3.11	2.72	2.61	2.46	2.33	2.21	2.17	2.14	2.08	1.94	1.90	2.03	2.27	2.26	2.05	1.51	1.14	4.96
Viet Nam	19.12	8.91	3.18	1.08	0.79	0.69	0.65	0.71	0.77	0.84	0.83	0.78	0.68	0.65	0.67	0.69	0.65	0.58	0.44	0.31	2.88
Yemen	220.20	78.02	27.00	8.07	7.15	6.87	6.48	5.98	5.48	5.37	5.54	5.79	5.94	6.14	6.04	5.57	4.91	4.28	3.33	2.55	49.42
Zambia	127.08	71.53	67.54	7.73	7.36	7.75	8.05	8.54	8.97	9.47	10.00	10.11	9.87	9.34	8.86	8.02	6.95	5.92	4.45	3.27	43.16
Zimbabwe	136.23	91.77	52.48	7.45	6.67	6.50	6.39	6.57	6.53	6.75	7.05	7.50	7.30	7.11	6.66	6.07	5.37	4.60	3.94	3.12	41.99

Abbreviations: DALY, disability adjusted life year; VAD, vitamin A deficiency.
